# Streamlined One‐Pot Synthesis of Fused Pyrroles: A Three‐Component Approach

**DOI:** 10.1002/open.70181

**Published:** 2026-03-26

**Authors:** Kateryna I. Marchenko, Vladislava I. Grinko, Roman M. Gutzul, Oleksandr S. Kharchenko, Henning J. Jessen, Nadiia N. Kolos

**Affiliations:** ^1^ Faculty of Chemistry and Pharmacy University of Freiburg Freiburg Germany; ^2^ Enamine Ltd. Kyiv Ukraine; ^3^ Faculty of Chemistry V. N. Karazin Kharkiv National University Kharkiv Ukraine

**Keywords:** 4,5,6,7‐tetrahydroindoles, arylglyoxals, Beckmann rearrangement, cyclic enaminones, functionalization, malononitrile, Meldrum's acid, multicomponent reaction, Passerini reaction, pyrrolo[2,3‐*d*]pyrimidines

## Abstract

New modified pyrrole derivatives, namely tetrahydroindol‐4‐ones and pyrrolo[2,3‐*d*]pyrimidines, functionalized at position 3, were synthesized through a one‐pot multicomponent condensation. Stabilized dimedone enaminones derived from α‐amino acids and their esters (or 6‐amino‐1,3‐dimethyluracil), arylglyoxals, and methylene‐active compounds (malononitrile, Meldrum's acid) were selected as key reagents for the reaction. Functionalization of 3‐cyanoacetamides within the 4,5,6,7‐tetrahydroindol‐4‐one framework was performed, involving transformations of the cyanoamide fragment, carbonyl group, and acetate residue at position 1 of the bicyclic system. The reactions proceeded under mild conditions, without the use of hazardous organic solvents or catalysts. Multicomponent condensation approach is particularly attractive for such syntheses, as it offers high atom economy, operational efficiency, and enables the rapid construction of structurally diverse and complex molecules in a single synthetic operation, thereby affording good yields with straightforward purification.

## Graphical abstract

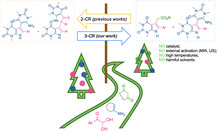

## 1 Introduction

The diversity of accessible chemical structures is fundamental to the discovery of novel collections of biologically active compounds [1]. One of the particularly effective strategies for achieving such diversity is the application of multicomponent reactions(MCRs), which enable the rapid generation of molecular libraries through systematic variation of the individual reaction components [2].

The methodology of MCRs involves the use of more than two starting compounds to rapidly obtain complex and diverse molecules. This provides the possibility to generate multiple analogs from simple starting materials [3]. MCRs can be considered a subclass of domino processes, as these reactions are carried out using all substrates in a single reactor under similar experimental conditions, with transformation steps occurring sequentially over time [4]. These reactions efficiently address synthetic challenges such as difficult purification processes, protection–deprotection steps, and they are often environmentally friendly. Well‐known strategies in the discovery of new MCRs include single reagent replacement (SRR), modular reaction sequences, combination of MCRs, post‐cycling strategies, and others [5, 6, 7].

The SRR strategy enables the optimization of reaction products for drug discovery by selecting building blocks that meet the fundamental criteria of drug similarity [8], including novel factors [9] such as the presence of a chiral center [10] and an increased Fsp^3^ parameterа descriptor used in drug development and cheminformatics to predict drug‐likeness, lipophilicity, solubility, bioavailability, etc [11]. This promotes the incorporation of pharmacophore or bioisosteric fragments into the final molecule and enables the rapid generation of diversity. The approach may also involve atypical reaction pathways, leading to the synthesis of new types of products, thereby exploring chemical space and expanding the synthetic potential of organic chemistry.

In particular, asymmetric poly‐substituted pyrroles and their condensed analogues can be synthesized through three‐ or four‐component condensation methods based on methylene‐active compounds, stabilized enamines [12], and substituted arylglyoxals. Specifically, the three‐component condensation of enaminoketones, arylglyoxal hydrates, and methylene‐active compounds (such as cyclic 1,3‐diketones, barbituric acid, and malononitrile) leads to the formation of 4,5,6,7‐tetrahydroindole derivatives functionalized at position 3 [13, 14, 15]. Various modifications of the four‐component condensations involving 1,3‐dicarbonyl compounds (e.g., dimedone and acetylacetone), arylglyoxals, methylene‐active compounds (e.g., malononitrile, *N*,*N*‐dimethylbarbituric acid and 4‐hydroxycoumarin), and primary amines are also well‐established. They lead to the formation of poly‐substituted pyrroles or tetrahydroindoles known in the literature. In some cases, the intermediate enamine is generated in situ from the 1,3‐diketone and amine [16, 17, 18, 19].

Indole and its derivatives hold a prominent position among heterocyclic compounds due to their significance as privileged pharmacological structures in natural bioactive products and pharmaceuticals [20, 21, 22, 23, 24, 25, 26, 27]. The most well‐known method for obtaining the indole ring is the Fischer synthesis, which has retained its significance to this day. In addition, the Reissert, Madelung, Bischler, Nenitzescu indole syntheses [28], and their numerous modifications also remain of practical importance. As a result, there is a sustained and growing interest in the development of novel and more efficient synthetic methodologies for the preparation of indole derivatives.

**FIGURE 1:**
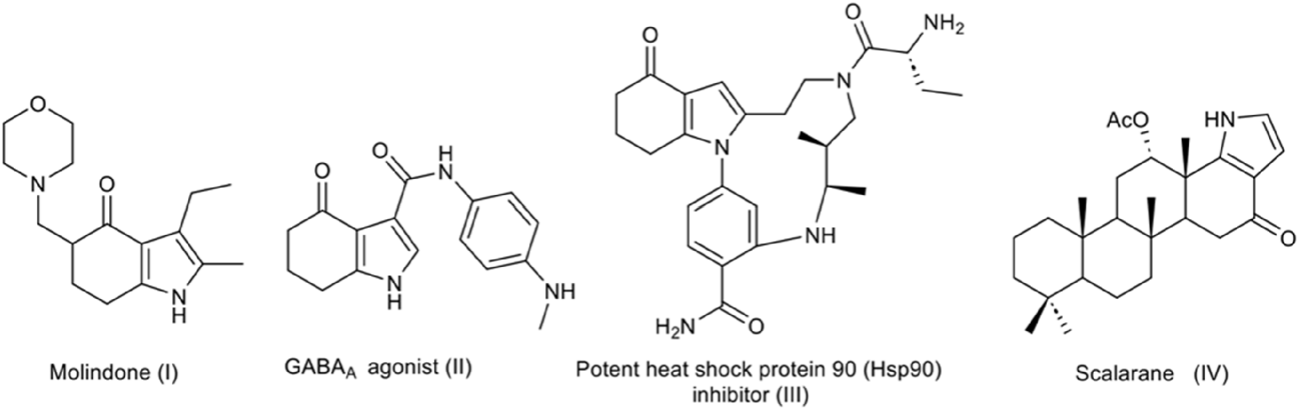
Pharmaceuticals containing the 4*H*‐indol‐4‐one moiety.

4,5,6,7‐Tetrahydroindole‐4‐ones constitute a key class of indole derivatives, owing to their structural versatility and biological activity. These compounds serve as core structures in a variety of drugs, such as the antipsychotic molindone (**I**) [29], used in the treatment of schizophrenia, the GABA_A_ agonist CP‐409092 for the treatmeant of anxiety (**II**) [30], and the potent heat shock protein 90 (Hsp90) inhibitor **III** for cancer treatment [31]. The indolone fragment is also found in natural products, e.g., as part of the structure of the sesterterpenoid scalarane **IV**, which was isolated from a marine sponge [32] (Figure 1).

## 2 Results and Discussion

Considering the significance of tetrahydroindole derivatives in medicinal and natural products chemistry, we report herein a one‐pot synthesis of indole‐3‐(1‐cyano)acetamides and indole‐3‐yl acetates utilizing enaminones of dimedone, arylglyoxals, and methylene‐active compounds. In comparison to earlier MCRs employing arylglyoxals and cyclic diketones, the present strategy offers enhanced structural diversity and streamlined reaction conditions, proceeding under mild and catalyst‐free environments, thus representing a significant advancement in MCR approach.

Dimedone enaminones **1** were synthesized via well‐established methods, employing readily available amino acids such as glycine, alanine, their methyl esters, as well as aniline and 4‐(aminomethyl)benzoic acid [33]. Arylglyoxals were synthesized via mild oxidation of the methyl group from the corresponding acetophenones, following a previously reported method [34].

**SCHEME 1:**
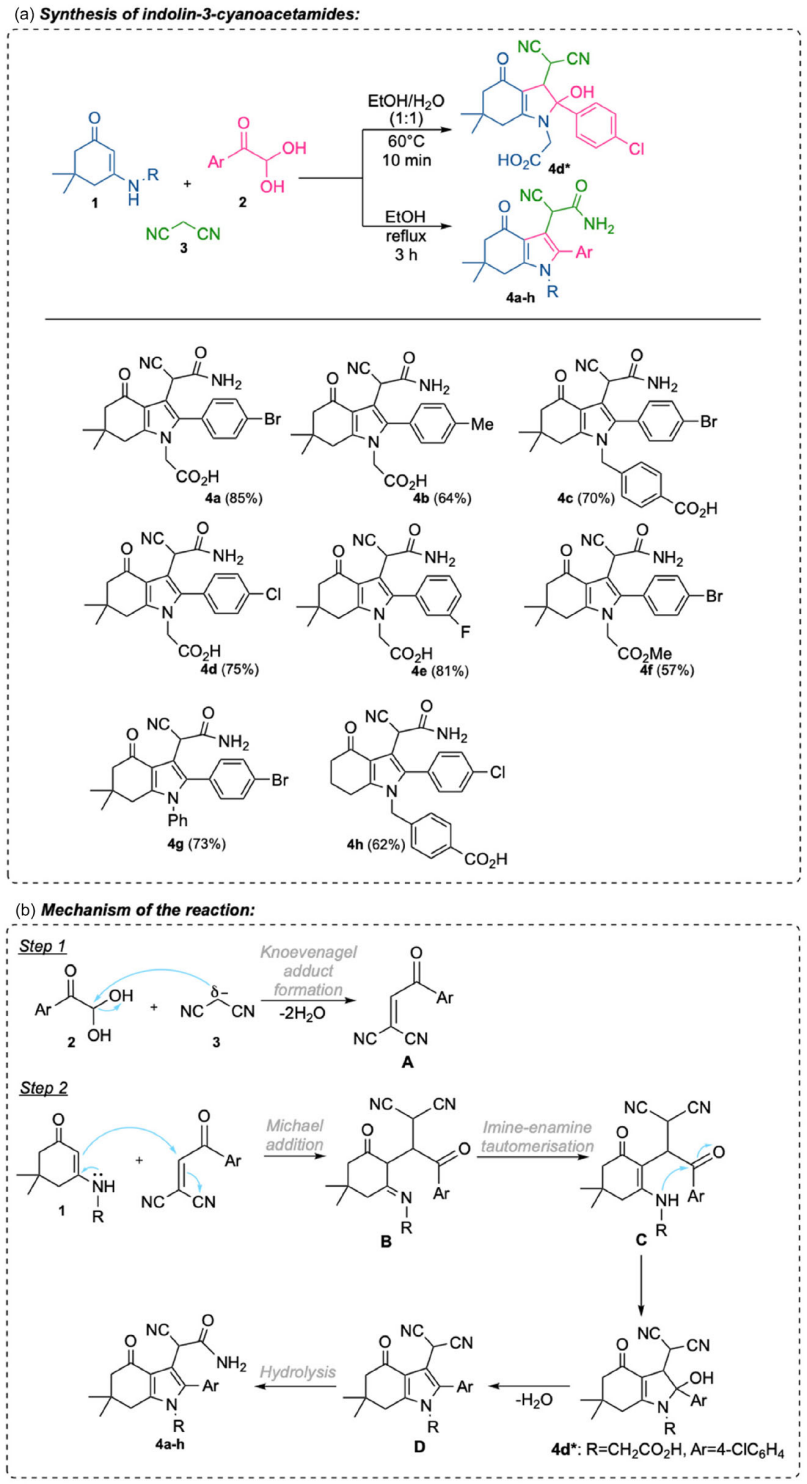
(a) Synthesis of indolin‐3‐cyanoacetamides via three‐component condensation. (b) Mechanism of the multicomponent reaction.

Heating enaminones **1**, arylglyoxals **2**, and malononitrile **3** in ethanol directly leads to cyanoacetamides **4a–h** in 62‐85% yields (Scheme 1a). In this study, our approach was based on the optimization results of a related three‐component reaction previously reported by one of us [35], in which 1,3‐dimethylbarbituric acid was used as the methylene component instead of nitrile **3**. In particular, we demonstrated that the use of aprotic solvents (Dimethylformamide (DMF), toluene) resulted in low yields of the target compounds, whereas performing the reaction in acetic acid led to the formation of a resinous by‐product, rendering purification impossible. In contrast, ethanol proved to be the optimal solvent for this transformation, allowing the condensation products to be obtained in yields ranging from 55% to 90%.

It was established (using *p*‐chloroglyoxal as an example) that the rapid reaction under mild conditions in aqueous ethanol (60°C, 10 min) results in the formation of the 2,3,4,5,6,7‐hexahydroindole derivative 4d*, which bears a hydroxyl group at position 2, thereby supporting the proposed reaction sequence. Compound 4d* was obtained as a mixture of diastereomers in an approximately 1:1 ratio, as evidenced by the ^1^H NMR spectrum, which exhibits a duplicated set of signals in the aromatic region. This ratio is further supported by the relative intensities of the methyl proton signals observed in the 1.05–1.10 ppm region.

Further heating of this compound induces dehydration and hydrolysis of a CN group, leading to the formation of indolone **4**. However, compounds **4**, derived from dimedone‐based enamino ketones and racemic alanine or its methyl ester, were not obtained, possibly due to steric hindrance from the α‐methyl group during the cyclization step. Stepwise addition of the reagents, specifically, heating *p*‐bromophenylglyoxal with malononitrile **3** in ethanol for 1 h, followed by refluxing of the resulting reaction mixture with the enaminone derived from alanine or its methyl ester and dimedone, did not result in the formation of the desired product as well. In contrast, the enaminone of dimedone and β‐alanine readily affords tetrahydroindol‐4‐one derivatives under similar MCR conditions [35, 36].

Our approach to cyanoacetamides **4** is based on the use of enaminoketones synthesized from dimedone and unsubstituted at the α‐position amino acids. The mechanism of the cyclization involves the initial formation of a Knoevenagel adduct between malononitrile and an arylglyoxal, followed by a Michael addition of the enaminone, an *exo*‐trig cyclization, and subsequent hydrolysis of one of the CN groups (Scheme 1b). The presence of the carboxyl group at position 1 of the pyrrole ring allows for various further modifications at this site, enhancing the solubility and bioavailability of the compounds in contrast to previously reported analogs with an aromatic core at this position [13, 18, 37, 38].

**SCHEME 2:**
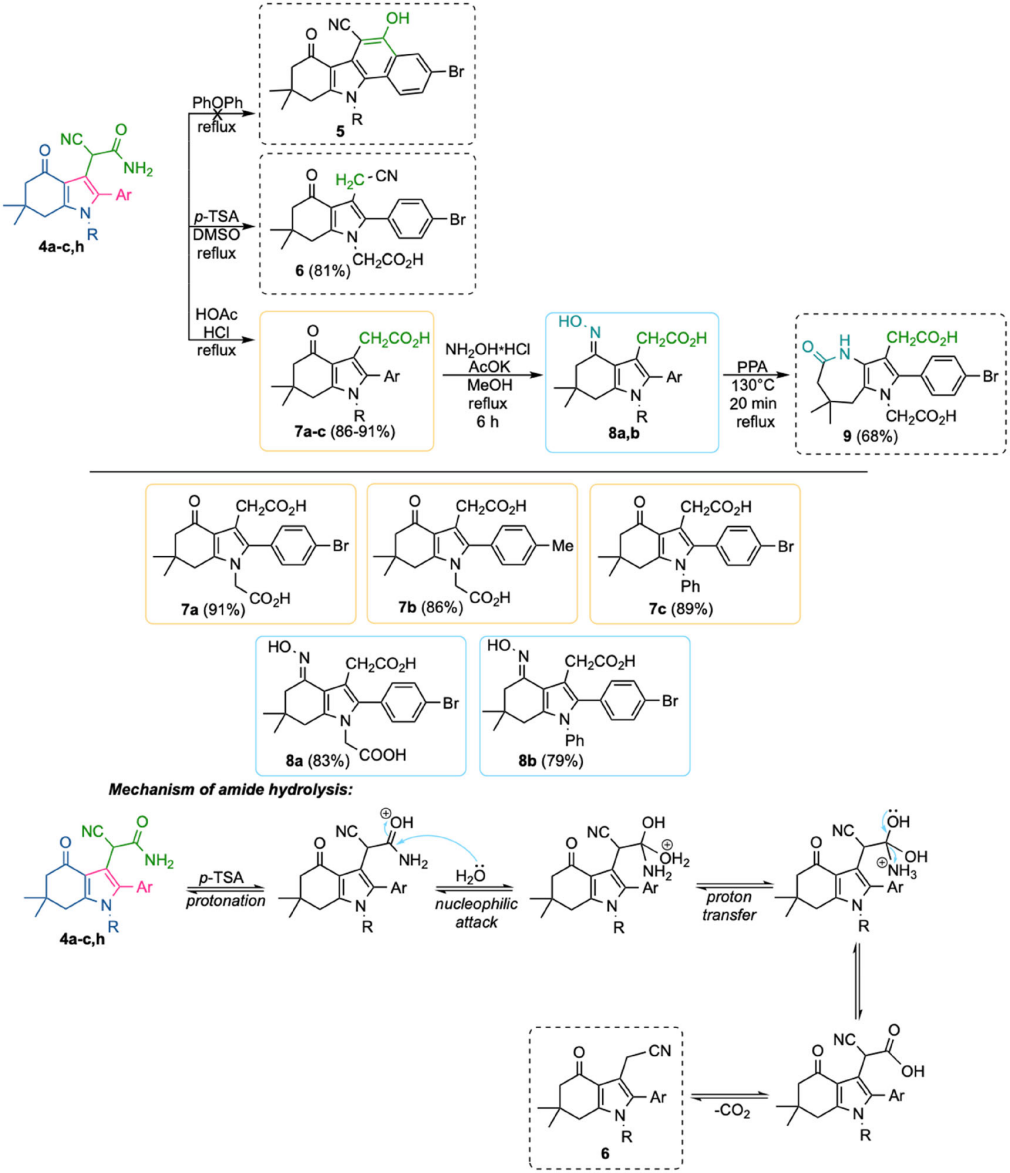
Functionalization of 3‐cyanoacetamide derivatives of tetrahydroindolin‐4‐one.

This synthetic sequence provides opportunities for subsequent molecular modifications via transformations of the available functional groups (Scheme 2). However, when compound **4a** was subjected to heating in diphenyl ether, product **5** was not isolated. Notably, heating compound **4a** in DMSO in the presence of *p‐*TSA resulted in the formation of indolone **6** with an acetonitrile moiety at position 3 of the bicyclic scaffold. Hydrolysis of the amide fragment in derivatives **4** proceeds via a classical acid‐catalyzed mechanism, resulting in the formation of an ammonium salt and a carboxylic acid. Upon heating, the latter undergoes decarboxylation facilitated by the presence of an electron‐withdrawing cyano group, ultimately leading to the formation of derivative **6**. Moreover, refluxing compounds **4** in a HCl/AcOH mixture (1:1) facilitated hydrolysis of the nitrile group and minimized the formation of polymeric by‐products and resulted in the formation of indolonacetic acids **7a–c** through decarboxylation with 86%–91% yield. It is known that a thermally induced cyclization of a structurally related cyanoacetamide at reflux in diphenylether leads to 5‐hydroxy‐6‐cyanobenzo[*a*]carbazole derivatives [13]. This transformation would be of interest as a potential route to polyheterocyclic systems to explore with our compounds.

The presence of a carbonyl group in tetrahydroindolones **4** enables nucleophilic addition reactions, particularly the synthesis of oximes **8a, b.** The *E*‐configured oximes **8a, b** were obtained in 79%–83% yields and high purity by heating the corresponding substrates in MeOH in the presence of KOAc. The Beckmann rearrangement of compound **8a**, carried out in polyphosphoric acid (PPA), led to the formation of pyrolodiazepinone **9** in 68% yield. In the context of this synthesis, the Beckmann rearrangement can be considered as a form of skeletal *N*‐editing of tetrahydroindoles [39].

**SCHEME 3:**
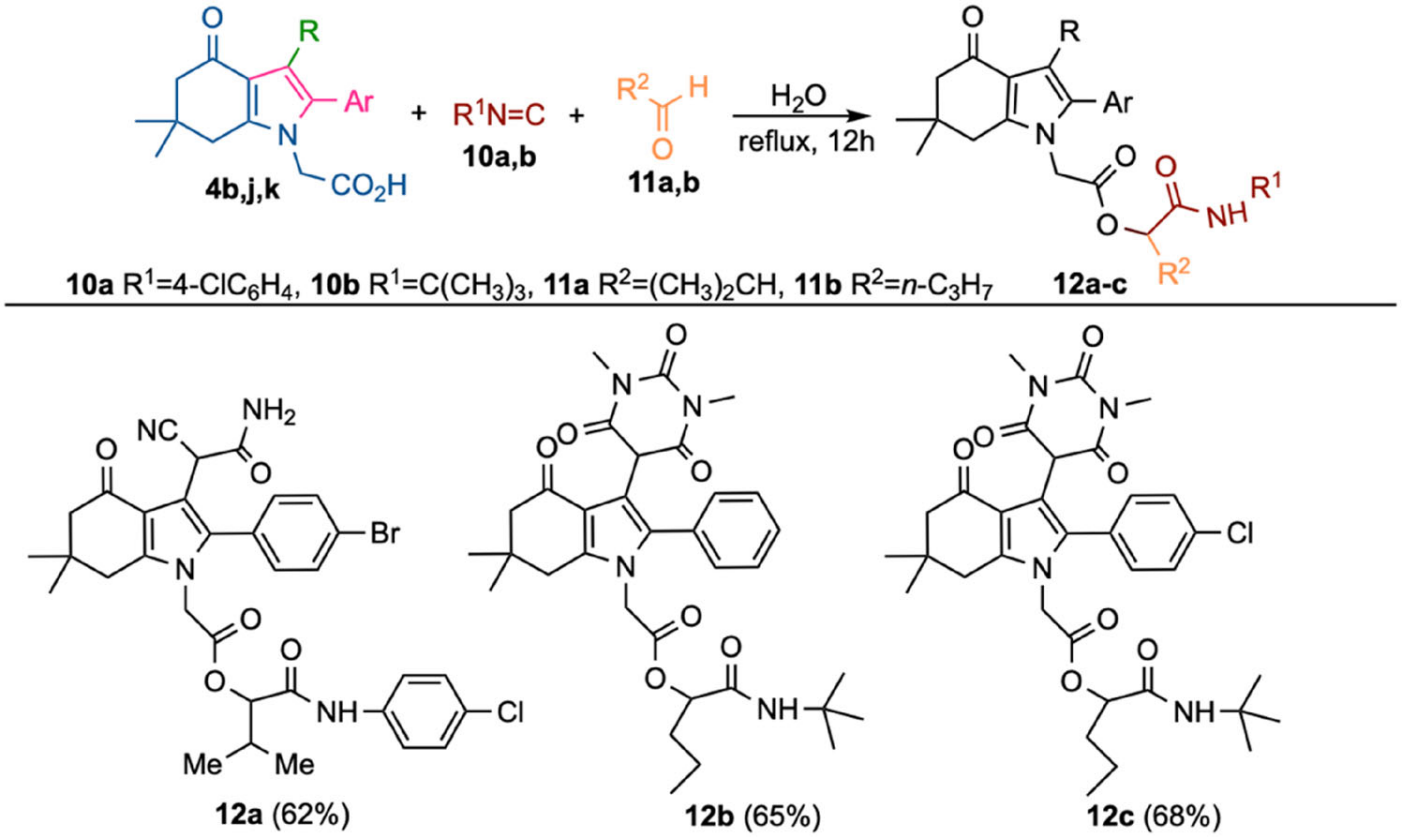
Passerini reaction of tetrahydroindol‐4‐one derivatives.

4,5,6,7‐Tetrahydro‐1*H*‐indole derivatives of type **4**, containing either a cyanoacetamide moiety or an *N*,*N*‐dimethylbarbituric acid residue at position 3 of the bicyclic core, were subjected to a Passerini MCR using isocyanides **10a,b** (**a** R^1^ = 4‐ClC_6_H_4_; **b** R^1^ = C(CH_3_)_3_) and aliphatic aldehydes **11a**,**b** (**a** R^2^ = (CH_3_)_2_CH; **b** R^2^ = C_3_H_7_). This allowed the synthesis of α‐acyloxycarboxamide derivatives **12a–c** (Scheme 3). Analysis of the ^1^H NMR spectrum of compound **12a**, which displays additional satellite signals with similar chemical shifts and comparable intensities alongside the principal resonances, indicates the formation of a diastereomeric mixture in an approximately 1:1 ratio. The starting indolin‐4‐ones **4j, k** were prepared via a three‐component condensation of dimedone‐derived enamino ketones **1** (R = CH_2_CO_2_H), arylglyoxals, and *N*, *N*‐dimethylbarbituric acid, following a procedure previously described [35].

**SCHEME 4:**
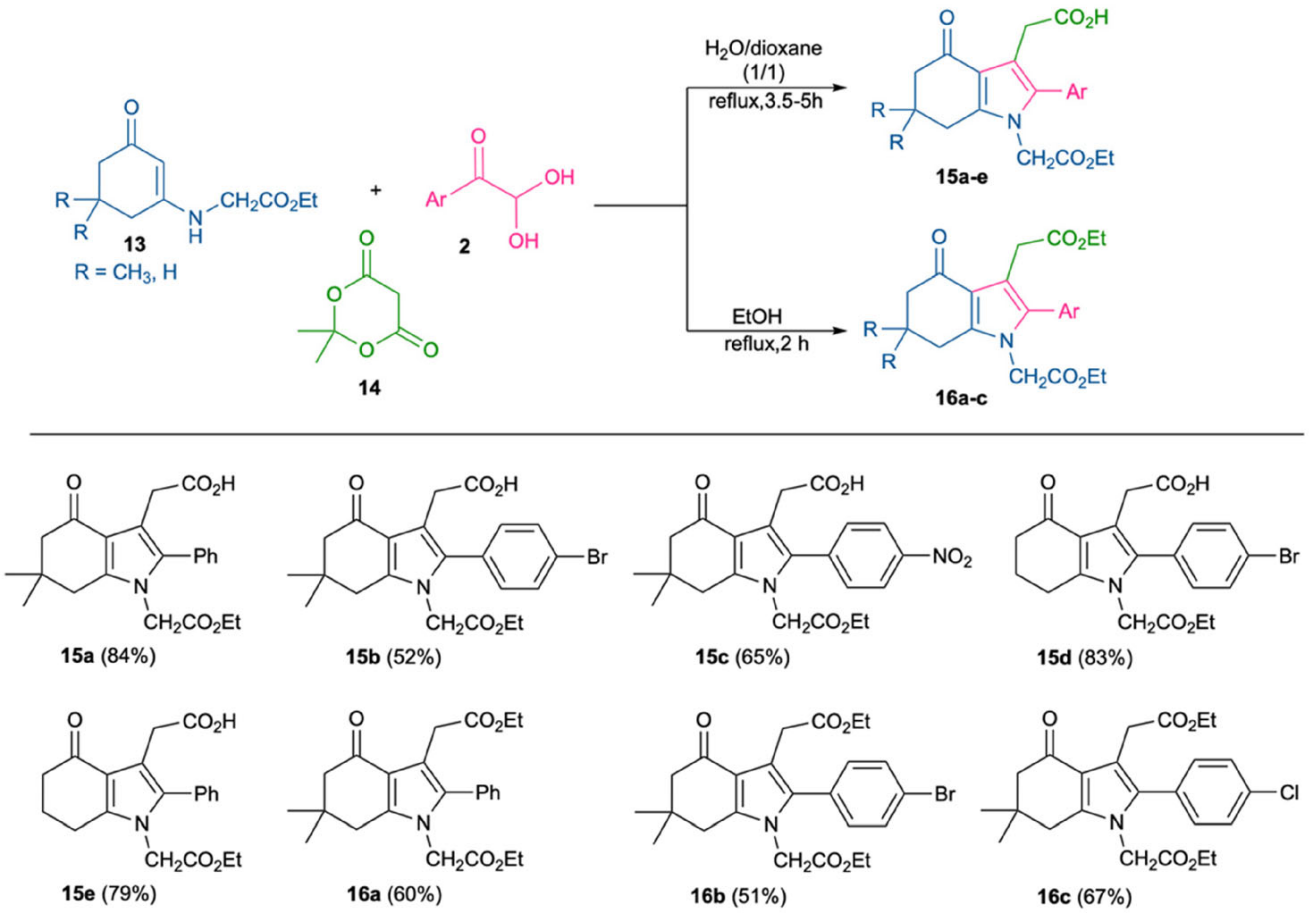
Three‐component approach to derivatives **15a–e** and **16a–c** from the same starting compounds.

Additionally, enaminoesters **13** were employed in a one‐pot condensation with arylglyoxal hydrates **2** and Meldrum's acid **14**, as shown in Scheme 4. It is well known that substituted derivatives of Meldrum's acid offer a broad scope for application in MCRs due to the tendency of Meldrum's acid to undergo decomposition with the formation of a ketene via the loss of acetone and CO_2_. This behavior can be controlled, making it valuable for numerous chemical processes [40, 41]. A variety of natural and synthetic bioactive scaffolds have been synthesized based on Meldrum's acid derivatives.

**FIGURE 2:**
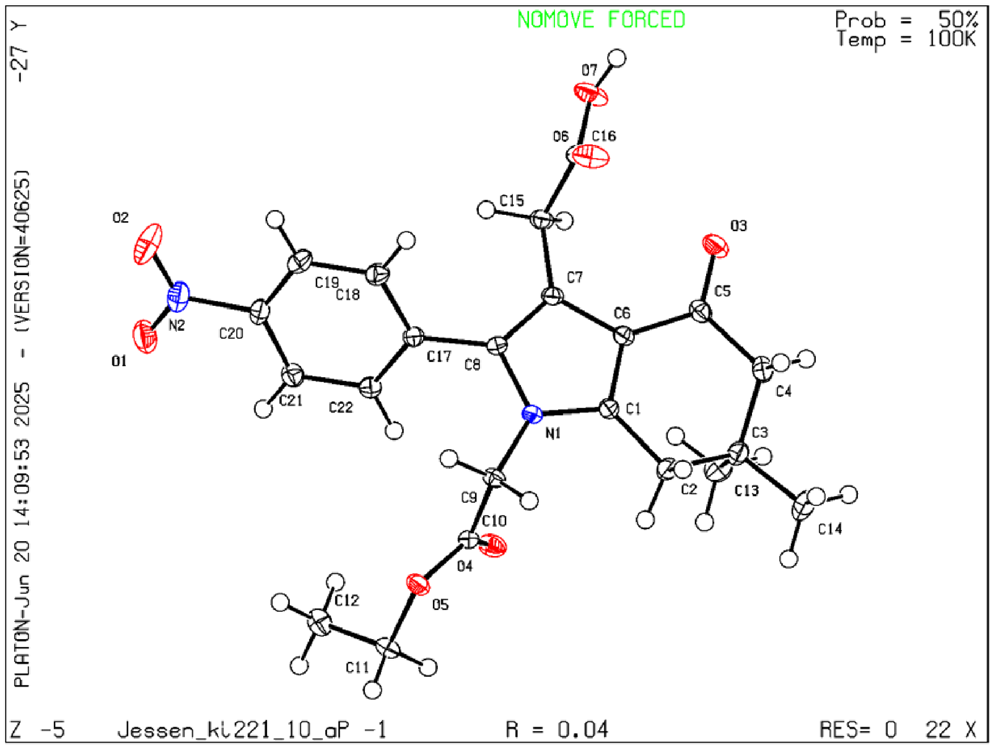
Crystal structure of derivative **15c** (CCDC: 2465978) determined by single‐crystal X‐ray diffraction.

As an extension of the reaction scope, we have optimized the conditions for the synthesis of carboxylic acids **15** and their corresponding esters **16**, which can serve as valuable substrates for further functionalization at positions 1 and 3 of the bicyclic system. The highest yields of analytically pure acids **15a–e** were achieved by heating the starting materials in a mixture of water/1,4‐dioxane (3:1) at 85°C in the absence of any catalyst. The reaction time under these conditions ranged from 3.5 to 5 h, depending on the substituents in the arylglyoxal moiety. The synthesis of esters **16a–c** was accomplished by heating the starting compounds in ethanol for 2 h, followed by purification via column chromatography, affording the target products in 51%–67% yield. The structure of compound **15с** was confirmed by single crystal X‐ray structural analysis (Figure 2).

**SCHEME 5:**
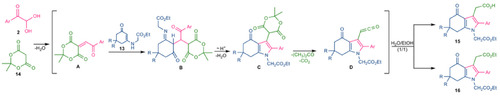
The reaction mechanism of the synthesis of сompounds **15** or **16**.

A plausible reaction mechanism for the formation of products 15 and 16 is shown in Scheme 5. This mechanism is consistent with the previously reported mechanism for the three‐component condensation of arylglyoxals, enaminones, and Meldrum's acid [40].

It is noteworthy that the reaction of enaminone **1** (R = CH_2_CO_**2**_H), *p*‐chlorophenylglyoxal, and Meldrum's acid **14** led to the isolation of two products in 31%–37% yields. Spectroscopic analysis indicated that both products were functionalized at position 7 of the bicyclic system and did not contain any structural fragments derived from Meldrum's acid.

**SCHEME 6:**
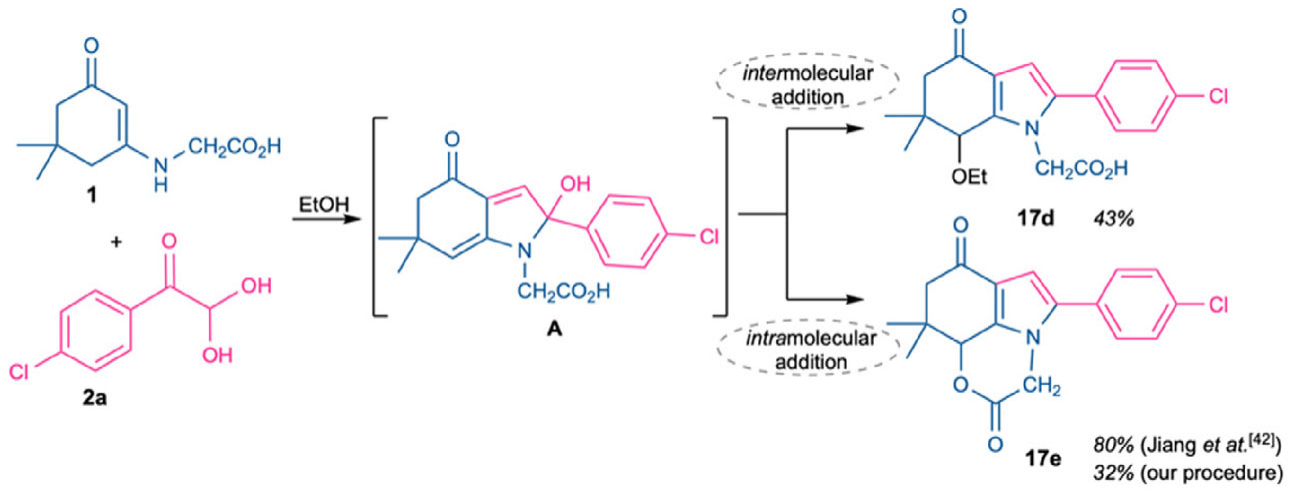
Synthesis of allylic addition products **17d** and **17e**.

The formation of compounds **17d** and **17e** is proposed to proceed via an allylic addition through the diene system (intermediate A) during the pyrrole ring‐formation step (Scheme 6). Formation of the novel 7‐ethoxytetrahydroindole derivative **17d** likely occurs through an intermolecular reaction with ethanol. In contrast, formation of derivative **17e** is proposed to involve an intramolecular addition of the α‐amino acid residue, corresponding to a typical CH‐activation domino process. Therefore, it is assumed that competition between external and internal nucleophiles occurs at the allylic addition stage, which had not been explored previously, leading to a decreased yield of **17e** compared with the previously reported value [42]. Nevertheless, the physicochemical characteristics of compound **17e** are consistent with Jiang et al. [42] data.

**SCHEME 7:**
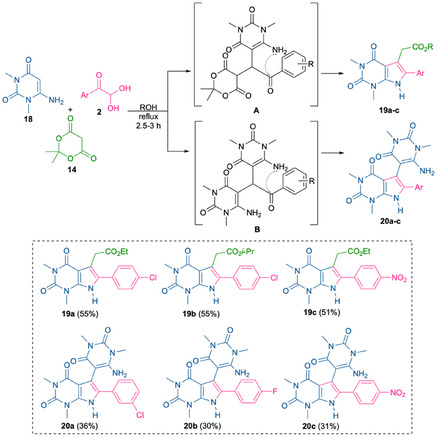
Synthesis of two different types of pyrrolo[2,3‐*d*]pyrimidine derivatives via the three‐component condensation of arylglyoxal, Meldrum's acid, and 6‐aminouracil.

Sequential addition of reagents in the reaction of arylglyoxals, Meldrum's acid, and 1,3‐dimethyl‐6‐aminouracil **18**, followed by refluxing the reaction mixture for 2.5–3 h, afforded pyrrolo[2,3‐*d*]pyrimidines **19a–c** in **51%–55%** yields (Scheme 7). Some of the products, namely **19a** and **19c**, precipitated directly from the reaction mixture upon reflux, allowing for isolation without further purification, which significantly reduces the time and effort required for their synthesis. However, compound **19b** required purification by column chromatography.

Interestingly, when *p*‐nitrophenylglyoxal was used as the aryl component, compound **20c** was also isolated from the reaction mixture. The spectral data differed significantly from those of pyrrolo[2,3‐*d*]pyrimidine compounds **19**. In the NMR ^1^H spectrum of this compound, a singlet corresponding to the NH proton of the pyrrole ring was observed, along with multiplets of aromatic protons, a broadened two‐proton singlet in the region of 6.2 ppm, and singlets of *N*‐methyl groups of the uracil fragment. In the cases of *m*‐chlorophenylglyoxal and *p*‐fluorophenylglyoxal, only compounds **20** were isolated from the reaction mixtures with low yields. The spectral characteristics of products **20a** and **20b** were similar to those of compound **20c**. These data, together with high‐resolution mass spectrometry results, suggest that a pseudo‐three‐component condensation occurs without the involvement of acid **14**. 1,3‐Dimethyl‐6‐aminouracil **18** competes with Meldrum's acid at the Knoevenagel product formation stage, despite the higher acidity of the latter (pKa values are 7.3 and 5.0, respectively). However, the greater electron‐donating capacity of the nitrogen atom significantly enhances the nucleophilicity at the position 5 of this amine. The involvement of a second molecule of 6‐aminouracil leads to the formation of products **20**.

The yields of derivatives **19c**/**20c** indicate a higher reactivity of Meldrum's acid in the formation of Knoevenagel products in comparison to 1,3‐dimethyl‐6‐aminouracil, which is entirely logical. However, the successful isolation of both types of products was still achievable in the case of *p*‐nitrophenylglyoxal, whereas reactions involving other arylglyoxals afforded exclusively either compounds of **19** or **20**. It should be pointed out that compounds **20**, synthesized via a two‐component (pseudo‐three‐component) reaction, were reported in 2023 by Panday et al. [43] In that study, the highest yield of the target product was achieved by heating 1,3‐dimethyl‐6‐aminouracil **18** with arylglyoxal **2a** in ethanol in the presence of *p*‐TSA (Table 1, entry 2). Conducting the reaction under identical conditions in the absence of *p*‐TSA resulted in a significant decrease in product yield (Table 1, entry 1). In addition, it is stated that the product yield increases substantially when a 2:1 ratio of derivative **18** to **2a** was employed, even in nonpolar aprotic solvents such as toluene.

**TABLE 1:** Ratios of the starting compounds employed by Panday et al*.* [43] (entries 1 and 2) and in the present study (entries 3 and 4).

Entry	Derivative **2a/2d**	Derivative 18	Derivative 14	*p*‐TSA	Yield of derivative 20a/20d
1	1 eq.	2 eq.	—	—	35%
2	1 eq.	2 eq.	—	10.0 mol%	80%
3	1 eq.	1 eq.	1 eq.	—	36%
4	1 eq.	2 eq.	1 eq.	—	32%

To examine this observation under our noncatalyzed conditions, we performed a model three‐component condensation of aminouracil derivative **18**, Meldrum's acid **14**, and *m*‐chlorophenylglyoxal **2d** in ethanol using 2:1:1 and 1:1:1 molar ratios of the starting materials (Table 1, entries 3 and 4). However, increasing the amount of aminouracil derivative **18** did not lead to an improvement in the yield of compound **20a**. This observation provides evidence that two competing pathways operate under these conditions, involving either Meldrum's acid or two equivalents of 6‐aminouracil.

**SCHEME 8:**
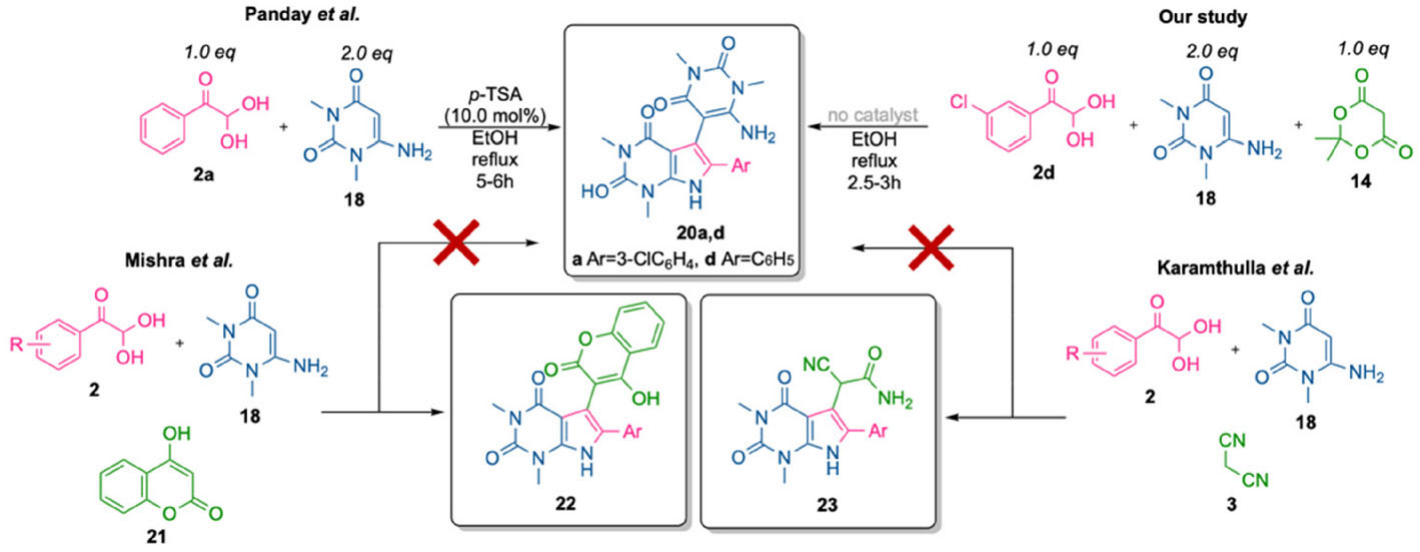
Synthesis of pyrrolo[2,3‐*d*]pyrimidine derivatives reported by Panday et al. [43], Karamthulla et al*.* [44], Mishra et al*.* [45] and the present three‐component approach.

Another study by Karamthulla et al. [44] reported the condensation of 1,3‐dimethyl‐6‐aminouracil **18**, arylglyoxals **2**, and malononitrile **3**. The authors reported two synthetic methods: a conventional heating approach and a microwave‐assisted procedure, the latter significantly reducing the reaction time and improving the yield. However, none of these methods yielded derivatives **20** rather pyrrolo[2,3‐*d*]pyrimidine derivatives **23** (Scheme 8).

Similarly, the three‐component condensation of 1,3‐dimethyl‐6‐aminouracil **18**, arylglyoxals **2**, and 4‐hydroxycoumarin **21** reported by Mishra et al. [45] proceeds via a similar Michael‐type addition of 5‐CH nucleophilic center of derivative **18** to the Knoevenagel intermediate followed by cyclization, providing a straightforward route to pyrrolo[2,3‐*d*]pyrimidine derivatives **22** bearing a 4‐hydroxycoumarin moiety at the 5‐position. In a similar manner, the formation of derivatives **20** was not mentioned.

**FIGURE 3:**
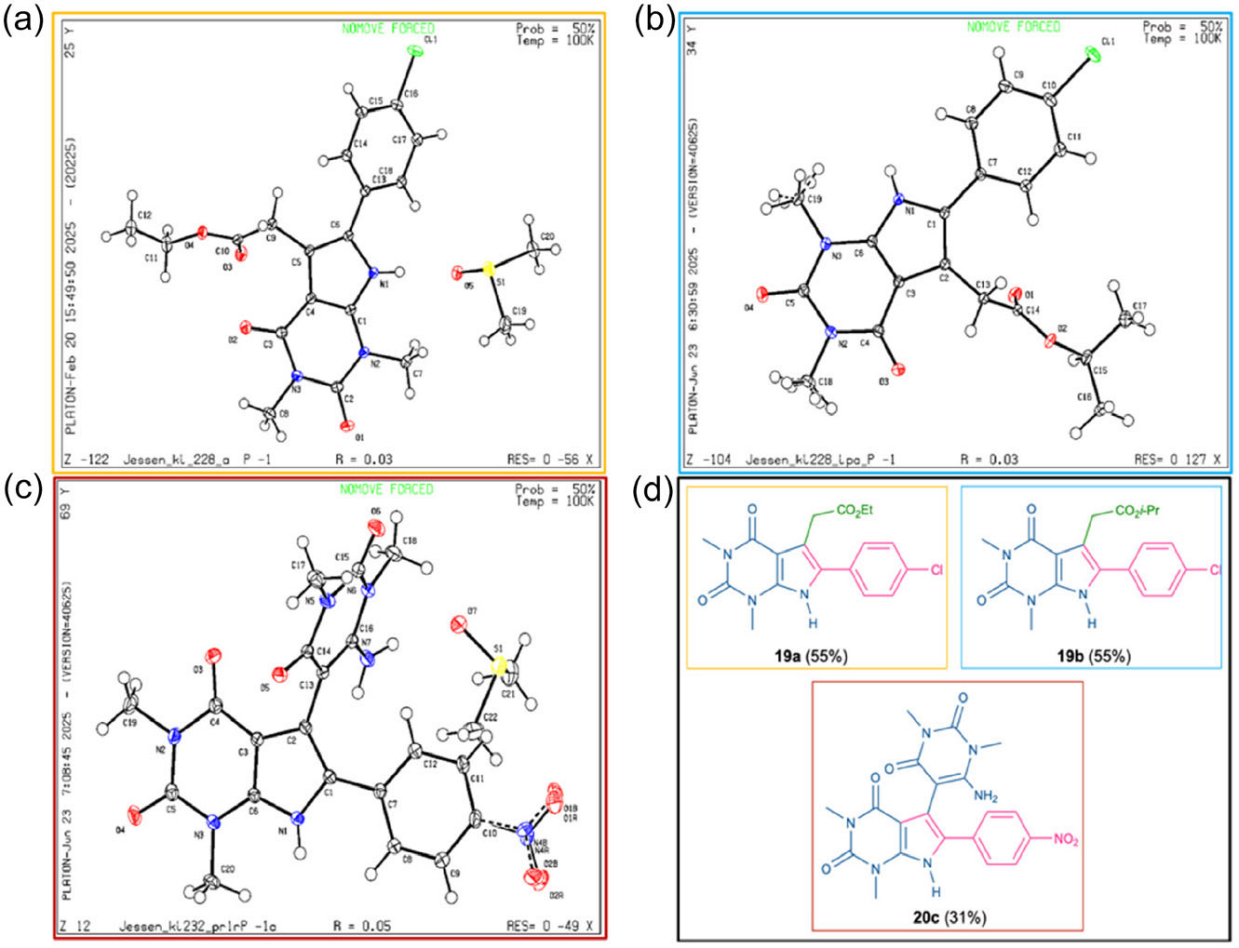
(a) Crystal structures of derivative **19a** (CCDC: 2425672) showing DMSO solvate determined by single‐crystal X‐ray diffraction. (b) Crystal structures of derivative **19b** (CCDC: 2466312) determined by single‐crystal X‐ray diffraction. (c) Crystal structures of derivative **20c** (CCDC: 2491896) showing DMSO solvate determined by single‐crystal X‐ray diffraction. (d) Structural formulas of derivatives **19a**, **19b** and **20c**.

The structures of compounds **19a**, **19b**, and **20c** were unambiguously established by single‐crystal X‐ray diffraction analysis. Notably, the crystals of the **19b** and **20c** derivatives include a DMSO solvate (Figure 3).

## 3 Conclusion

In this study, it was delineated that the combination of enaminoketones based on dimedone and glycine or its esters, arylglyoxals, and methylene‐active compounds (malononitrile, Meldrum's acid) provides a route to the synthesis of indolin‐4‐one‐3‐cyanoacetamides, indolin‐4‐one‐3‐acetic acids, and indolin‐4‐one‐3‐acetate esters. Such a cascade reaction can be conveniently carried out without prior activation or the use of an additional catalyst. Despite the structural sensitivity and limitations of the reaction, when the presence of an alkyl or aryl substituent at the α‐position of the enaminone derived from α‐amino acids hinders the synthesis of indole derivatives, this approach opens ready access to a novel “non‐planar” chemical space relevant to medicinal chemistry. Functional transformations of the tetrahydroindolone scaffold were successfully achieved via oxime formation and Beckmann rearrangement, as well as through the synthesis of Passerini adducts. Furthermore, a series of pyrrolo[2,3‐*d*]pyrimidine derivatives was synthesized in alcohols via a MCR involving 6‐amino‐1,3‐dimethyluracil, arylglyoxals, and Meldrum's acid. It was shown that in the presence of electron‐withdrawing groups in the arylglyoxal molecules, a competing pseudo‐three‐component reaction occurs, leading to the formation of pyrrolo[2,3‐*d*]pyrimidine derivatives bearing a 6‐amino‐1,3‐dimethyluracil fragment at position 3 of the bicyclic system. Meanwhile, the pyrrolo[2,3‐*d*]pyrimidine derivatives resembling purine‐like scaffolds may find a place as valuable platforms for biological studies. The obtained tetrahydroindole derivatives can be further functionalized at positions 1 and 3 of the bicyclic system, thereby expanding the scope of accessible analogs.

## Supporting Information

Additional supporting information can be found online in the Supporting Information section. **Supporting Table S1**: Crystal data and structure refinement for Jessen_ki_228_a. **Supporting Table S2**: Atomic coordinates and *U*_*eq*_ [Å^2^] for Jessen_ki_228_a. **Supporting Table S**3: Anisotropic displacement parameters [Å^2^] for Jessen_ki_228_a. The anisotropic displacement factor exponent takes the form: −2π^2^[ *h* ^2^(a^*^)^2^ *U*_11_ *+ k* ^2^(b^*^)^2^ *U*_22_ + … + 2hka*b^*^U_12_]. **Supporting Table S**4: Bond lengths and angles for Jessen_ki_228_a. **Supporting Table S**5: Torsion angles for Jessen_ki_228_a.

## Conflicts of Interest

The authors declare no conflicts of interest.

## Supporting information

Supplementary Material

## Data Availability

The data supporting this article have been included as part of the ESI.
