# Supplementary material for: Streamlined One‐Pot Synthesis of Fused Pyrroles: A Three‐Component Approach
Source: ChemistryOpen. 2026 Mar 26;15(4):e70181. doi: 10.1002/open.70181 (PMC13140558; doi:10.1002/open.70181)
Supplement: Supplementary file 1 — Supplementary Material [file OPEN-15-e70181-s001.pdf]

Supporting Information

Streamlined One-Pot Synthesis of Fused Pyrroles: A Three-Component Approach

Kateryna I. Marchenko,[a] Vladislava I. Grinko,[b] Roman M. Gutzul,[b] Oleksandr S. Kharchenko,[b] Henning J. Jessen,[a] Nadiia N. Kolos*[c]

[a] Faculty of Chemistry and Pharmacy, University of Freiburg, Albertstr. 21, 79104 Freiburg, Germany

[b] Enamine Ltd., Winston Churchill st. 78, 02094 Kyiv, Ukraine

[c] Faculty of Chemistry, V. N. Karazin Kharkiv National University, Svobody Sq. 4, 61022 Kharkiv, Ukraine

*Correspondence: Prof. Dr. Nadiia N. Kolos, email: nadiia.kolos@karazin.ua

Table of Contents

General Information..3

General procedure for the Synthesis of Compounds **4-20** and Characterization........4

1. Preparation of the Starting Compounds........4
2. General Procedure for the Synthesis of Compounds **4a-h**........4
3. General Procedure for the Synthesis of Compound **4d*** 6
4. General Procedure for the Synthesis of Compound **6**........7
5. General Procedure for the Synthesis of Compounds **7a-c**........7
6. General Procedure for the Synthesis of Compounds **8a,b**........8
7. General Procedure for the Synthesis of Compound **9**........9
8. General Procedure for the Synthesis of Compounds **12a-c**........9
9. General Procedure for the Synthesis of Compounds **15a-e**........11
10. General Procedure for the Synthesis of Compounds **16a-c**........13
11. General Procedure for the Synthesis of Compounds **17d,e**........14
12. General Procedure for the Synthesis of Compounds **19a-c**........15
13. General Procedure for the Synthesis of Compounds **20a-c**........16

X-Ray Diffraction Data........17

^{1}H, ^{13}C NMR, HRMS and LCMS spectra of all compounds........48

General Information

All commercially available reagents and solvents were purchased from commercial vendors and used without purification.

The progress of reactions was monitored by TLC on *Merck* Silica gel 60 F_{254} plates (0.25 mm layer thickness, fluorescence indicator). Visualization was achieved by UV-light (λ = 254 nm).

Flash chromatography was carried out using silica gel 60 (0.04-0.063 nm) from *Macherery-Nagel.*

Normal-Phase-Medium Pressure Liquid Chromatography (NP-MPLC) was performed with the Flash Chromatography System PuriFlash® 430 from Interchim®. PuriFlash® Silica HP Flash Columns were purchased from Interchim®.

Nuclear magnetic resonance spectra (^{1}H NMR, ^{13}C NMR) were recorded on a *Bruker Avance III HD 300 MHz, Bruker Avance II 400 MHz,* and *Bruker Avance II 500 MHz* spectrometers in DMSO-d_6, $CDCl_3$, CD_3OD or CD_3CN solutions with TMS as an internal reference. The spectra were analyzed with the software *MestReNova 12.0.1*. The chemical shift δ was given in part per million (ppm) and the coupling constant *J* in Hertz (Hz). The common abbreviations for characterization of signal multiplicity were used (s, singlet; d, doublet; t, triplet; q, quartet; m, multiplet).

Elemental analyses were carried out on an EA 3000 Eurovector elemental analyzer.

High resolution mass spectra (HRMS, electrospray ionization) were recorded by analytical department of the university of Freiburg, institute of organic chemistry using a *Thermo LCQ Advantage* (spray voltage: 2.5-4.5 kV, spray current: 5 µA, ion transfer tube: 250 (150)°C, evaporation temp.: 50-400 °C).

Melting points were determined on a Melting Point Meter KSP1D.

Single-crystal X-ray crystallographic data were obtained from a shock-cooled single crystal at 100(2)°K on a Bruker APEX2 QUAZAR three-circle diffractometer with a microfocus sealed X-ray tube using a mirror optics as monochromator and a Bruker APEXII detector. The diffractometer was equipped with an Oxford Cryostream 800 low temperature device and used MoK_α radiation (λ = 0.71073 Å). All data were integrated with SAINT V8.41 and a multi-scan absorption correction using SADABS 2016/2 was applied.[1,2] The structure was solved by direct methods with SHELXT and refined by full-matrix least-squares methods against F^2 using SHELXL-2019/2.[3,4] All non-hydrogen atoms were refined with anisotropic displacement parameters. All hydrogen atoms were refined with isotropic displacement parameters. Some of their coordinates were refined freely and some on calculated positions using a riding model with their U_{iso} values constrained to 1.5 times the U_{eq} of their pivot atoms for terminal sp^3 carbon atoms and 1.2 times for all other carbon atoms. Disordered moieties were refined using bond lengths restraints and displacement parameter restraints.

General procedure for the Synthesis of Compounds 4-20 and Characterization

1. Preparation of the Starting Compounds

β-Enaminoketones[5,6] **1** and **13** and arylglyoxal hydrates[7] **2** were synthesized according to previously reported procedures.

2. General Procedure for the Synthesis of Compounds 4a-h

A mixture of β-enaminoketone **1** (1.0 mmol), arylglyoxal monohydrate **2** (1.0 mmol), and malononitrile **3** (1.1 mmol) in ethanol (7 mL) was heated under reflux in a 10 mL round-bottom flask for 3 h. Upon completion of the reaction (monitored by TLC), the mixture was cooled to room temperature, and the resulting precipitate was collected by filtration. The crude product was then recrystallized from ethanol to afford pure compounds **4a-h** as white powders.

2-(3-(2-Amino-1-cyano-2-oxoethyl)-2-(4-bromophenyl)-6,6-dimethyl-4-oxo-4,5,6,7-tetrahydro-1H-indol-1-yl)acetic acid (**4a**)

R_f=0.68 (DCM/MeOH 10/1); ^{1}H NMR (400 MHz, DMSO-d_6): δ = 7.67 (d, ^2J = 8.4 Hz, 2H, Ar-H), 7.38 (s, 1H, NH), 7.29-7.22 (m, 3H, Ar-H+NH), 5.12 (s, 1H, CH), 4.47 (s, 2H, CH_2), 2.65 (s, 2H, CH_2), 2.32 (s, 2H, CH_2), 1.06 (s, 6H, CH_3) ppm; ^{13}C NMR (101 MHz, DMSO-d_6): δ = 193.4, 169.2, 165.5, 154.7, 144.5, 132.7 (2C), 131.6 (2C), 128.0, 127.3, 122.8, 115.3, 108.8, 56.0, 51.6, 46.0, 35.0, 34.8, 28.2, 28.0 ppm; HRMS (ESI): *m/z* calcd for $C_{21}H_{20}BrN_3O_4$-H^+: 456.0564 [*M*-H]$^+$; found: 456.0562.

2-(3-(2-Amino-1-cyano-2-oxoethyl)-6,6-dimethyl-4-oxo-2-(p-tolyl)-4,5,6,7-tetrahydro-1H-indol-1-yl)acetic acid (**4b**)

R_f=0.43 (DCM/MeOH 2/1); ^{1}H NMR (400 MHz, DMSO-d_6): δ = 7.42 (s, 1H, NH), 7.39-7.23 (m, 3H, Ar-H+NH), 7.18 (d, ^3J = 7.7 Hz, 2H, Ar-H), 4.97 (s, 1H, CH), 4.48 (s, 2H, CH_2), 2.66 (s, 2H, CH_2), 2.41 (s, 3H, CH_3), 2.32-2.39 (m, 2H, CH_2), 1.07 (s, 6H, CH_3) ppm; ^{13}C NMR (101 MHz, DMSO-d_6): δ = 193.4, 172.0, 166.2, 143.9, 138.9, 134.8, 131.1 (2C), 129.8 (2C), 126.5, 117.4, 115.6, 109.1, 52.1, 35.9, 35.3, 34.8, 28.8, 28.5, 28.4, 21.4 ppm; HRMS (ESI): *m/z* calcd for $C_{22}H_{23}N_3O_4$+Na^+: 416.1586 [*M*+Na]$^+$, found: 416.1589 [*M*+Na]$^+$; *m/z* calcd for $C_{22}H_{23}N_3O_4$-H^+: 392.1615 [*M*-H]$^+$; found: 392.1617.

4-((3-(2-amino-1-cyano-2-oxoethyl)-2-(4-bromophenyl)-6,6-dimethyl-4-oxo-4,5,6,7-tetrahydro-1H-indol-1-yl)methyl)benzoic acid (**4c**)

R_f=0.47 (CH/EE 1/1); m.p. 214-216°C; ^{1}H NMR (400 MHz, DMSO-d_6): δ = 7.82 (d, ^3J = 8.4 Hz, 2H, Ar-H), 7.47-7.42 (m, 3H, Ar-H+NH), 7.32 (s,1H, NH), 7.25 (d, ^3J = 8.0 Hz, 2H, Ar-

H), 7.12 (d, ^3J = 8.4 Hz, 2H, Ar-H), 5.20 (s, 1H, CH), 5.12 (s, 2H, CH_2), 2.69 (t, ^3J = 6.2 Hz, 2H, CH_2), 2.41 (t, ^3J = 6.2 Hz, 2H, CH_2), 1.07 (s. 6H, CH_3) ppm; ^{13}C NMR (101 MHz, DMSO-d_6): δ = 193.9, 169.8, 166.0, 145.0 (2C), 133.7, 133.2 (2C), 132.1 (2C), 128.5 (2C), 123.3 (2C), 120.2, 117.2, 115.7, 114.1, 109.2, 52.1, 46.5, 35.5 (2C), 35.3 (2C), 28.4, 27.9 ppm; HRMS (ESI): *m/z* calcd for $C_{27}H_{24}BrN_3O_4$+H^+: 534.1023 [*M*+*H*]$^+$; found: 534.1032 [*M*+*H*]$^+$.

2-(3-(2-amino-1-cyano-2-oxoethyl)-2-(4-chlorophenyl)-6,6-dimethyl-4-oxo-4,5,6,7-tetrahydro-1H-indol-1-yl)acetic acid (**4d**)

R_f=0.56 (DCM/MeOH 2/1); m.p. 198-199°C; ^{1}H NMR (400 MHz, DMSO-d_6): δ = 7.56 (d, ^3J = 8.4 Hz, 2H, Ar-H), 7.43 (s, 1H, NH), 7.34-7.29 (m, 3H, Ar-H+NH), 5.12 (s, 1H, CH), 4.56-4.44 (m, 2H, CH_2), 2.66 (s, 2H, CH_2), 2.32 (d, ^4J = 3.1 Hz, 2H, CH_2), 1.07 (s, 6H, CH_3) ppm; ^{13}C NMR (101 MHz, DMSO-d_6): δ = 193.9, 169.8, 166.1, 145.0, 134.5, 133.7, 133.0 (2C), 129.2 (2C), 128.1, 117.2, 115.7, 109.3, 52.1, 46.4, 35.5, 35.3 (2C), 28.8, 28.6 ppm; HRMS (ESI): *m/z* calcd for $C_{21}H_{20}ClN_3O_4$+H^+: 414.1216 [*M*+H]$^+$; found: 414.1207 [*M*+H]$^+$.

2-(3-(2-amino-1-cyano-2-oxoethyl)-2-(3-fluorophenyl)-6,6-dimethyl-4-oxo-4,5,6,7-tetrahydro-1H-indol-1-yl)acetic acid solvate with EtOH (**4e**)

R_f=0.50 (DCM/MeOH 2/1); m.p. =195-197°C; ^{1}H NMR (400 MHz, DMSO-d_6): δ = 7.56-7.49 (m, 1H, Ar-H), 7.46 (s, 1H, NH), 7.35-7.28 (m, 2H, Ar-H+NH), 7.17-7.11 (m, 2H, Ar-H), 5.15 (s, 1H, CH), 4.58-4.46 (m, 2H, CH_2), 2.66 (s, 2H, CH_2), 2.32 (s, 2H, CH_2), 1.07 (s, 6H, CH_3) ppm; ^{13}C NMR (101 MHz, DMSO-d_6): δ = 194.0, 169.9 (2C), 166.1 (2C), 149.2, 145.1, 133.6, 131.4, 117.2, 115.7, 115.5, 109.4, 52.1, 35.3 (2C), 32.8 (2C), 28.7, 28.5, 28.4 ppm; HRMS (ESI): *m/z* calcd for $C_{21}H_{20}FN_3O_4$+H^+: 398.1511 [*M*+H]$^+$, found: 398.1512 [*M*+H]$^+$.

methyl 2-(3-(2-amino-1-cyano-2-oxoethyl)-2-(4-bromophenyl)-6,6-dimethyl-4-oxo-4,5,6,7-tetrahydro-1H-indol-1-yl)acetate (**4f**)

R_f=0.52 (DCM/MeOH 2/1); ^{1}H NMR (400 MHz, DMSO-d_6): 7.68 (d, ^3J = 7.7 Hz, 2H, Ar-H), 7.44 (s, 1H, NH), 7.32 (s, 1H, NH), 7.22 (d, ^3J = 7.7 Hz, 2H, Ar-H), 5.13 (s, 1H, CH), 4.63 (q, ^2J=7.0 Hz, 2H, CH_2), 3.65 (s, 3H, CH_3), 2.66 (s, 2H, CH_2), 2.33 (s, 2H, CH_2), 1.06 (s, 6H, CH_3) ppm; ^{13}C NMR (101 MHz, DMSO-d_6): δ = 194.0, 180.0, 169.0, 145.1, 134.0, 133.3 (2C), 132.2 (2C), 128.3, 123.4, 117.2, 116.0, 109.6, 71.1, 53.1, 52.2, 46.3, 35.5, 35.4, 28.7, 28.5 ppm; elemental analysis calcd (%) for $C_{22}H_{22}BrN_3O_4$: C 55.94, H 4.69, N 8.90, found: C 55.93, H 4.70, N 8.91.

2-(2-(4-Bromophenyl)-6,6-dimethyl-4-oxo-1-phenyl-4,5,6,7-tetrahydro-1H-indol-3-yl)-2-cyanoacetamide (**4g**)

R_f=0.43 (CH/EE 1/1); ^{1}H NMR (400 MHz, CD_3OD): δ = 7.77-7.57 (m, 6H, Ph+NH), 7.54-7.28 (m, 5H, Ar+NH), 5.46 (s, 1H, CH), 2.83 (s, 2H, CH_2), 2.71 (s, 2H, CH_2), 1.36 (s, 6H, CH_3) ppm; ^{13}C NMR (101 MHz, DMSO-d_6): δ = 193.3, 165.6, 144.3, 136.2, 133.8, 132.7 (2C), 131.1 (2C), 129.2 (2C), 128.6 (2C), 128.4, 127.9, 121.9, 116.9, 115.8, 109.4, 51.8, 36.0, 35.4, 35.0, 28.2, 27.8 ppm; HRMS (ESI): *m/z* calcd for $C_{25}H_{22}BrN_3O_2$+Na^+: 498.0788 [M+Na]$^+$; found: 498.0781 [M+Na]$^+$.

4-((3-(2-amino-1-cyano-2-oxoethyl)-2-(4-chlorophenyl)-4-oxo-4,5,6,7-tetrahydro-1H-indol-1-yl)methyl)benzoic acid (**4h**)

R_f=0.45 (CH/EE 1/1); m.p. 208-209°C; ^{1}H NMR (400 MHz, DMSO-d_6): δ = 7.85 (d, ^3J = 8.1 Hz, 2H, Ar-H), 7.40-7.45 (m, 3H, Ar-H+NH), 7.30 (s, 1H, NH), 7.26 (d, ^3J = 8.2 Hz, 2H, Ar-H), 7.02 (d, ^3J = 8.1 Hz, 2H, Ar-H), 5.19 (s, 1H, CH), 5.11 (s, 2H, CH_2), 2.69 (t, ^3J = 6.2 Hz, 2H, CH_2), 2.41 (t, ^3J = 6.4 Hz, 2H, CH_2), 2.05 (t, ^3J = 6.2 Hz, 2H, CH_2) ppm; ^{13}C NMR (101 MHz, DMSO-d_6): δ = 194.5, 182.1, 181.9, 167.4 (2C), 166.1 (2C), 145.5, 142.3, 134.5, 133.7, 133.1, 130.3, 130.2 (2C), 129.1 (2C), 128.2, 126.6 (2C), 117.4, 117.2, 110.0, 47.6, 38.2, 35.6, 23.3 ppm; HRMS (ESI): *m/z* calcd for $C_{25}H_{20}ClN_3O_4$+H^+: 462.1216 [M+H]$^+$; found: 462.1208 [M+H]$^+$.

3. General Procedure for the Synthesis of Compound 4d*

A mixture of β-enaminoketone **1** (1.0 mmol), *p*-chlorophenylglyoxal monohydrate **2a** (1.0 mmol), and malononitrile **3** (1.1 mmol) in ethanol (7 mL) was heated under reflux for 0.5 h. After completion of the reaction (monitored by TLC), the reaction mixture was poured into a saturated brine solution. The resulting white precipitate was collected by filtration, washed with water, and dried under vacuum. Recrystallization from ethanol afforded compound **4d*** as a white powder.

2-(2-(4-chlorophenyl)-3-(dicyanomethyl)-2-hydroxy-6,6-dimethyl-4-oxo-2,3,4,5,6,7-hexahydro-1H-indol-1-yl)acetic acid (**4d***)

R_f=0.46 (DCM/MeOH 1/4); m.p. 194-197°C;^{1}H NMR (400 MHz, DMSO-d_6): δ = 13.28 (s, 1H, OH), 7.57 (d, ^3J = 8.5 Hz, 2H, Ar-H), 7.33 (d, ^3J = 8.5 Hz, 2H, Ar-H), 5.14 (s, 1H, OH), 4.52 (d, ^2J = 7.2 Hz, 2H, CH_2), 3.32 (s, 2H, 2CH), 2.68 (s, 2H, CH_2), 2.34 (d, ^2J = 1.9 Hz, 2H, CH_2), 1.09 (s, 6H, CH_3) ppm; ^{13}C NMR (101 MHz, DMSO-d_6): 194.0, 169.9, 166.1,

145.0, 134.6, 133.7, 133.0 (2C), 129.2 (2C), 128.1, 117.3, 115.8, 109.4, 52.2, 46.4, 35.5, 35.4, 35.3, 28.7, 28.6 ppm; HRMS (ESI): *m/z* calcd for $C_{21}H_{20}ClN_3O_4$-H^+: 412.1069 [*M*-H]$^+$; found: 412.1073 [*M*-H]$^+$; elemental analysis calcd (%) for $C_{21}H_{20}ClN_3O_4$: C 60.95, H 4.87, N 10.15, found: C 60.96, H 4.88, N 10.14.

4. General Procedure for the Synthesis of Compound 6

In a three-neck flask equipped with a thermometer and a reflux condenser, compound **4a** (0.5 g, 1.0 mmol) was dissolved in DMSO (6 mL), followed by the addition of *p*-toluenesulfonic acid (10 mg). The reaction mixture was heated at 170 °C for 2 h under stirring. After cooling to room temperature, water (15 mL) was added, resulting in the formation of a precipitate. The solid was collected by filtration, washed with water, and dried to afford compound **6** as a light yellow powder (0.42 g, 81%).

2-(2-(4-bromophenyl)-3-(cyanomethyl)-6,6-dimethyl-4-oxo-4,5,6,7-tetrahydro-1H-indol-1-yl)acetic acid (**6**)

R_f=0.53 (CH/EE 1/1); ^{1}H NMR (400 MHz, CD_3OD): δ =7.73 (d, ^3J=7.7 Hz, 2H, Ar-H), 7.23 (d, ^3J = 7.7 Hz, 2H, Ar-H), 4.52 (s, 2H, CH_2), 3.73 (s, 2H, CH_2), 2.64 (s, 2H, CH_2), 2.30 (s, 2H, CH_2), 1.07 (s, 6H, CH_3) ppm; ^{13}C NMR (101 MHz, DMSO-d_6): δ = 193.7, 169.9, 160.6, 157.8, 144.8, 132.8, 132.7 (2C), 132.5 (2C), 128.8, 122.9, 119.1, 108.6, 52.3, 46.6, 35.4, 35.3, 28.7 (2C) ppm; LCMS (ESI): *m/z* calcd for $C_{20}H_{19}BrN_2O_3$+H^+: 415.0 [*M*+H]$^+$; found: 415.0 [*M*+H]$^+$; elemental analysis calcd (%) for $C_{20}H_{19}BrN_2O_3$: C 57.84, H 4.61, N 6.75, found: C 57.84, H 4.62, N 6.77.

5. General Procedure for the Synthesis of Compounds 7a-c

A corresponding tetrahydroindole derivative **4a-c** (1.0 mmol) was refluxed in a 1:1 mixture of concentrated HCl and AcOH (20 mL) for 6 h. Upon completion of the reaction, the mixture was poured into water (50 mL) and left to stand for 1 h. The resulting precipitate was collected by filtration, washed with water, and dried under vacuum to afford compounds **7a-c** as white powders.

2,2'-(2-(4-Bromophenyl)-6,6-dimethyl-4-oxo-4,5,6,7-tetrahydro-1H-indole-1,3-diyl)diacetic acid (**7a**)

R_f=0.31 (DCM/MeOH 2/1); m.p. >300°C; ^{1}H NMR (400 MHz, CD_3OD): δ = 7.88 (d, ^3J = 8.1 Hz, 2H, Ar-H), 7.47 (d, ^3J = 8.1 Hz, 2H, Ar-H), 4.77 (s, 2H, CH_2), 3.77 (s, 2H, CH_2), 2.93 (s, 2H, CH_2), 2.61 (s, 2H, CH_2), 1.40 (s, 6H, CH_3) ppm; ^{13}C NMR (101 MHz, DMSO-

d$_6$): δ = 193.1, 172.4, 169.6, 147.4, 143.6, 132.3 (2C), 131.7 (2C), 129.3 121.8, 116.7, 112.8, 51.9, 45.8, 35.0, 34.7, 30.7, 28.2 (2C) ppm; HRMS (ESI): *m/z* calcd for $C_{20}H_{20}BrNO_5+H^+$: 434.0598 [*M*+H]$^+$; found: 434.0595 [*M*+H]$^+$.

2,2'-(6,6-Dimethyl-4-oxo-2-(p-tolyl)-4,5,6,7-tetrahydro-1H-indole-1,3-diyl)diacetic acid (**7b**)

R$_f$=0.34 (DCM/MeOH 2/1); ^{1}H NMR (400 MHz, CD_3OD): δ = 7.54 (d, ^3J = 7.7 Hz, 2H, Ar-H), 7.42 (d, ^3J = 7.7 Hz, 2H, Ar-H), 4.76 (s, 2H, CH_2), 3.76 (s, 2H, CH), 2.93 (s, 2H, CH_2), 2.61-2.64 (m, 5H, CH_2+CH_3), 1.41 (s, 6H, CH_3) ppm; ^{13}C NMR (101 MHz, DMSO-*d*$_6$): δ = 192.3, 172.5, 169.7, 147.2, 143.2, 137.7, 133.3, 129.9 (2C), 129.3 (2C), 125.1, 116.4, 112.1, 51.9, 45.8, 34.8, 30.8, 28.3 (2C), 20.8 ppm; HRMS (ESI): *m/z* calcd for $C_{21}H_{23}NO_5+Na^+$: 392.1576 [*M*+Na]$^+$, found: 392.1570 [*M*+Na]$^+$; *m/z* calcd for $C_{21}H_{23}NO_5$-H^+: 368.1503 [*M*-H]$^+$; found: 368.1500 [*M*-H]$^+$.

2-(2-(4-Bromophenyl)-6,6-dimethyl-4-oxo-1-phenyl-4,5,6,7-tetrahydro-1H-indol-3-yl)acetic acid (**7c**)

R$_f$=0.57 (DCM/MeOH 10/1); m.p. >300°C; ^{1}H NMR (400 MHz, CD_3CN): δ = 7.58-7.35 (m, 5H, Ar-H), 7.20 (d, ^3J = 7.3 Hz, 2H, Ar-H), 7.13 (d, ^3J = 7.3 Hz, 2H, Ar-H), 3.57 (s, 2H, CH_2), 2.60 (s, 2H, CH_2), 2.52 (s, 2H, CH_2), 1.11 (s, 6H, CH_3) ppm; ^{13}C NMR (101 MHz, DMSO-*d*$_{6}$,): δ = 193.4, 172.3, 169.4, 150.1, 136.6, 131.9 (2C), 131.1 (2C), 129.6, 129.3 (2C), 128.2, 127.7 (2C), 120.9, 117.4, 113.7, 52.0, 36.1, 34.9, 30.8, 28.0 (2C) ppm; HRMS (ESI): *m/z* calcd for $C_{24}H_{22}BrNO_3+Na^+$: 474.0676 [*M*+Na]$^+$; found: 474.0671 [*M*+Na]$^+$.

6. General Procedure for the Synthesis of Compounds 8a,b

A solution of the appropriate tetrahydroindole derivative **7a-c** (1.0 mmol), anhydrous potassium acetate (2.5 mmol), and hydroxylamine hydrochloride (2.5 mmol) in dry methanol (20 mL) was stirred under reflux in a three-necked round-bottom flask equipped with a thermometer and a reflux condenser for 6 h. The reaction progress was monitored by thin-layer chromatography (TLC). After completion, the reaction mixture was allowed to cool to room temperature. The resulting precipitate was collected by vacuum filtration, washed with cold methanol, and dried under reduced pressure to afford compounds **8a,b** as white powders.

(E)-2,2'-(2-(4-Bromophenyl)-4-(hydroxyimino)-6,6-dimethyl-4,5,6,7-tetrahydro-1H-indole-1,3-diyl)diacetic acid (**8a**)

R$_f$=0.69 (DCM/MeOH 2/1); m.p. 245-246°C; ^{1}H NMR (400 MHz, CD_3OD): δ = 10.35 (s,1H, OH), 7.59 (d, ^3J = 8.6 Hz, 2H, Ar), 7.23 (d, ^3J = 8.5, 2H, Ar), 4.45 (s, 2H, CH_2), 3.41 (s, 2H,

CH_2), 2.60 (s, 2H, CH_2), 2.48 (s, 2H, CH_2), 1.09 (s, 6H, CH_3) ppm; ^{13}CNMR (101 MHz, DMSO-d_6): δ = 172.7, 170.1, 152.1, 134.1, 131.9 (2C), 131.5 (2C), 131.3, 130.1, 121.2, 112.4, 110.5, 45.6, 36.0, 34.9, 32.2, 31.8, 28.6 (2C) ppm; HRMS (ESI): *m/z* calcd for $C_{20}H_{21}BrN_2O_5$+H^+: 449.0707 [*M*+H]$^+$; found: 449.0712 [*M*+H]$^+$;

(E)-2-(2-(4-Bromophenyl)-4-(hydroxyimino)-6,6-dimethyl-1-phenyl-4,5,6,7-tetrahydro-1H-indol-3-yl)acetic acid (**8b**)

R_f=0.60 (CH/EE 2/1); m.p. >300°C;^{1}H NMR (400 MHz, CD_3OD): δ = 10.40 (s, 1H, OH), 7.49-7.18 (m, 5H, Ph), 7.06 (d, ^3J=7.7 Hz, 2H, Ar-H), 7.10 (d, ^3J=7.7 Hz, 2H, Ar-H), 3.54 (s, 2H, CH_2), 2.62 (s, 2H, CH_2), 2.39 (s, 2H, CH_2), 1.03 (s, 6H, CH_3) ppm; ^{13}C NMR (101 MHz, DMSO-d_6): δ=173.2, 152.4, 137.8, 134.5, 132.3 (2C), 131.8, 131.5 (2C), 131.0, 129.6 (2C), 128.2 (2C), 128.0, 120.7, 114.0, 112.4, 36.6, 36.5, 32.7, 32.4, 28.9 (2C) ppm; HRMS (ESI): *m/z* calcd for $C_{24}H_{23}BrN_2O_5$+H^+: 467.0965 [*M*+H]$^+$; found: 467.0971 [*M*+H]$^+$.

7. General Procedure for the Synthesis of Compound 9

Oxime derivative **8a** (1.0 mmol) was added to polyphosphoric acid (PPA, 10 mL) and the mixture was stirred at 130 °C for 20 minutes. Upon completion (monitored by TLC), the reaction mixture was allowed to cool to room temperature and then poured into a mixture of ice and water. The suspension was left to stand overnight to ensure complete precipitation. The resulting solid was collected by vacuum filtration and recrystallised from ethanol to afford compound **9** as a transparent oil.

2,2'-(2-(4-Bromophenyl)-7,7-dimethyl-5-oxo-5,6,7,8-tetrahydropyrrolo[3,2-b]azepine-1,3(4H)-diyl)diacetic acid (**9**)

R_f=0.64 (CH/EE 2/1); m.p. >300°C;^{1}H NMR (400 MHz, CD_3OD): δ = 8.28 (s, 1H, NH), 7.30 (d, ^3J = 7.7 Hz, 2H, Ar-H), 6.91 (d, ^3J = 7.7 Hz, 2H, Ar-H), 4.20 (s, 2H, CH_2), 3.11 (s, 2H, CH_2), 2.67 (s, 2H, CH_2), 2.31 (s, 2H, CH_2), 0.77 (s, 6H, CH_3) ppm; ^{13}C NMR (101 MHz, DMSO-d_6): 186.0, 171.1, 160.9, 151.8, 136.5, 132.9 (2C), 132.0 (2C), 129.9, 122.3, 118.9, 114.9, 114.1, 52.5, 46.5, 38.1, 33.4, 26.9 (2C) ppm; LCMS (ESI): *m/z* calcd for $C_{20}H_{21}BrN_2O_5$+H^+: 449.0 [*M*+H]$^+$; found: 449.0 [*M*+H]$^+$; elemental analysis calcd (%) for $C_{20}H_{21}BrN_2O_5$: C 53.47, H 4.71, N 6.24, found: C 53.55, H 4.80, N 6.17.

8. General Procedure for the Synthesis of Compounds 12a-c

A mixture of the appropriate tetrahydroindole derivative **4b**, **4j**, or **4k** (1.0 equiv), aldehyde **11a** or **11b** (1.1 equiv) and isocyanide **10a** or **10b** (1.1 equiv) was stirred at 35 °C in chloro-

form for 3 h. Upon completion (monitored by TLC), the reaction mixture was diluted with ethyl acetate (30 mL). The organic layer was separated, dried over anhydrous Na_2SO_4, filtered, and concentrated under reduced pressure to afford the crude products **12a-c** as light yellow powders.

1-((4-chlorophenyl)amino)-3-methyl-1-oxobutan-2-yl 2-(3-(2-amino-1-cyano-2-oxoethyl)-2-(4-bromophenyl)-6,6-dimethyl-4-oxo-4,5,6,7-tetrahydro-1H-indol-1-yl)acetate (**12a**)

R_f=0.60 (CH/EE 2/1); ^{1}H NMR (400 MHz, DMSO-d$_6$): δ = 10.24 (s, 1H, NH), 7.65-7.38 (m, 8H, Ar-H+NH_2), 5.15 (d, ^2J = 7.2 Hz, 1H, CH), 4.69-4.72 (m, 3H, CH_2+CH), 2.72-2.68 (m, 2H, CH_2), 2.31 (s, 2H, CH_2), 2,16-2.10 (m, 1H, CH), 1.05 (s, 3H, CH_3), 1.09 (s, 3H, CH_3), 0.88 (d, ^2J = 6.8 Hz, 6H, 2CH_3) ppm; ^{13}C NMR (101 MHz, DMSO-d_6): 193.1, 186.0, 168.5, 167.4, 166.0, 152.2, 145.9, 139.4, 137.7, 134.6, 132.1 (2C), 129.2 (2C), 121.6 (2C), 127.5, 123.4, 121.5 (2C), 104.5, 79.6 (2C), 75.2, 52.2, 35.3 (2C), 30.3, 28.8, 25.8, 18.9, 17.1 ppm; LCMS (ESI): *m/z* calcd for $C_{32}H_{32}BrClN_4O_5$+H^+: 667.1 [*M*+H]$^+$; found: 667.2 [*M*+H]$^+$; *m/z* calcd for $C_{32}H_{32}BrClN_4O_5$-H^+: 665.1 [*M*+H]$^+$; found: 665.2 [*M*+H]$^+$; elemental analysis calcd (%) for $C_{32}H_{32}BrClN_4O_5$: C 57.54, H 4.83, N 8.39, found: C 57.60, H 4.98, N 8.52.

1-(Tert-butylamino)-1-oxopentan-2-yl-2-(3-(1,3-dimethyl-2,4,6-trioxohexahydropyrimidin-5-yl)-6,6-dimethyl-4-oxo-2-phenyl-4,5,6,7-tetrahydro-1H-indol-1-yl)acetate (**12b**)

R_f=0.49 (DCM/MeOH 12/1); m.p. 109-112°C; ^{1}H NMR (400 MHz, DMSO-d_6): δ = 7.63 (s, 1H, NH), 7.42-7.48 (m, 3H, Ar-H+NH), 7.36 (d, ^3J = 7.0 Hz, 2H, Ar-H), 4.86 (t, ^3J = 6.4 Hz, 1H,CH), 4.75 (s, 2H, CH_2), 4.68 (s, 1H, CH), 3.14 (s, 3H, CH_3), 3.12 (s, 3H, CH_3), 2.68 (s, 2H, CH_2), 2.18 (s, 2H, CH_2), 1.63 (q, ^3J = 7.3 Hz, 2H, CH_2), 1.44-1.37 (m, 2H, CH_2), 1.27 (s, 9H, CH_3), 1.06 (s. 3H, CH_3), 1.02 (s. 3H, CH_3), 0.87 (t, ^3J = 7.3 Hz, 3H, CH_3) ppm; ^{13}C NMR (101 MHz, DMSO-d_6): δ=193.7, 168.5 (2C), 168.1 (2C), 152.4, 144.6, 136.7, 130.6 (2C), 129.4 (2C), 129.1, 115.6, 111.7, 79.7, 75.1, 51.4 (2C), 47.7, 46.4, 35.7, 35.3, 33.9, 30.6 (2C), 28.9, 28.4, 18.3 (2C), 14.1 (3C) ppm; HRMS (ESI): *m/z* calcd for $C_{33}H_{42}N_4O_7$+H^+: 607.3127 [*M*+H]$^+$; found: 607.3136; elemental analysis calcd (%) for $C_{33}H_{42}N_4O_7$: C 65.33, H 6.98, N 9.23, found: C 65.35, H 6.97, N 9.23.

1-(Tert-butylamino)-1-oxopentan-2-yl 2-(2-(4-chlorophenyl)-3-(1,3-dimethyl-2,4,6-trioxohexahydropyrimidin-5-yl)-6,6-dimethyl-4-oxo-4,5,6,7-tetrahydro-1H-indol-1-yl)acetate (**12c**)

R_f=0.51 (DCM/MeOH 12/1); m.p. 118-121°C; ^{1}H NMR (400 MHz, DMSO-d_6): δ = 7.63 (s, 1H, NH), 7.54 (d,^3J = 8.3 Hz, 2H, Ar-H), 7.37 (d, ^3J = 8.3 Hz, 2H, Ar-H), 4.86-4.76 (m, 3H,

CH_2+CH), 4.70 (s, 1H, CH), 3.14 (s, 3H, CH_3), 3.12 (s, 3H, CH_3), 2.68 (s, 2H, CH_2), 2.17 (s, 2H, CH_2), 1.66-1.60 (m, 2H, CH_2), 1.41-1.39 (m, 2H, CH_2), 1.27 (s, 9H, CH_3), 1.05 (s. 3H, CH_3), 1.05 (s. 3H, CH_3), 0.87 (t, ^3J = 7.3 Hz, 3H, CH_3) ppm; ^{13}C NMR (101 MHz, DMSO-d_6): δ = 193.7, 168.4 (2C), 168.0 (2C), 152.4, 144.9, 135.4, 134.1, 132.4, 129.4, 128.3, 115.6, 112.2, 79.7, 75.1, 51.3 (2C), 50.8, 47.7, 46.5, 35.7, 35.2, 33.9, 30.6 (2C), 28.9 (3C), 28.4, 18.3, 14.1 (2C) ppm; HRMS (ESI): *m/z* calcd for $C_{33}H_{41}ClN_4O_7+Na^+$: 663.2556 [*M*+Na]$^+$; found: 663.2554; *m/z* calcd for $C_{33}H_{41}ClN_4O_7-H^+$: 639.2591 [*M-H*]$^+$; found: 639.2586; elemental analysis calcd (%) for $C_{33}H_{41}ClN_4O_7$: C 61.82, H 6.45, N 8.74, found: C 61.83, H 6.45, N 8.75.

9. General Procedure for the Synthesis of Compounds 15a-e

Arylglyoxal monohydrate **2** (1.0 mmol) and Meldrum's acid **14** (1.1 mmol) were dissolved in a mixture of H_2O/1,4-dioxane (7 mL, v/v ratio) and heated to reflux. β-Enaminoketone **13** (1.0 mmol) was then added, and the resulting mixture was stirred under reflux overnight. After completion (monitored by TLC), the solvent was removed under reduced pressure. The resulting oily residue was dissolved in ethyl acetate and washed with a saturated aqueous brine solution. The organic layer was dried over anhydrous Na_2SO_4, filtered, and concentrated under reduced pressure. The crude product was purified by flash column chromatography on silica gel using EtOAc/EtOH (4:1) as eluent to afford the target compounds **15a-e** as white powders.

2-(1-(2-Ethoxy-2-oxoethyl)-6,6-dimethyl-4-oxo-2-phenyl-4,5,6,7-tetrahydro-1H-indol-3-yl)acetic acid (**15a**)

R_f=0.60 (DCM/MeOH 12/1); ^{1}H NMR (500 MHz, $CDCl_3$): δ = 11.91 (s, 1H, OH), 7.47-7.38 (m, 2H, Ar-H), 7.32-7.24 (m, 1H, Ar-H), 7.21-7.15 (m, 2H, Ar-H), 4.59 (s, 2H, CH_2), 4.10 (q, ^3J = 7.0 Hz, 2H, CH_2), 3.39 (s, 2H, CH_2), 2.62 (s, 2H, CH_2), 2.24 (s, 2H, CH_2), 1.12 (t, ^3J = 7.1 Hz, 3H, CH_3), 1.05 (s, 6H, CH_3), ppm; ^{13}C NMR (125 MHz, $CDCl_3$): δ = 193.7, 172.9, 168.7, 143.9, 133.8, 130.6 (2C), 130.3, 129.2 (2C), 128.8, 117.3, 113.0, 61.7, 52.4, 46.4, 35.5, 35.3, 31.3, 28.8 (2C), 14.4 ppm; HRMS (ESI): *m/z* calcd for $C_{22}H_{25}NO_5+Na^+$: 406.1733 [*M*+Na]$^+$; found: 406.1624 [*M*+Na]$^+$; elemental analysis calcd (%) for $C_{22}H_{25}NO_5$: C, 68.91; H, 6.57; N, 3.65; found: %: C, 69.83; H, 6.60; N, 3.57.

2-(2-(4-Bromophenyl)-1-(2-ethoxy-2-oxoethyl)-6,6-dimethyl-4-oxo-4,5,6,7-tetrahydro-1H-indol-3-yl)acetic acid (**15b**)

R_f=0.57 (DCM/EE 10/1); ^{1}H NMR (400 MHz, $CDCl_3$): δ = 12.33 (s, 1H, OH), 7.54 (d, ^3J=8.6 Hz, 2H, Ar-H), 7.15 (d, ^3J = 8.5 Hz, 2H, Ar-H), 4.36 (s, 2H, CH_2), 4.18 (q, ^3J = 7.1 Hz, 2H, CH_2), 3.39 (s, 2H, CH_2), 2.53 (s, 2H, CH_2), 2.44 (s, 2H, CH_2), 1.21 (t, ^3J = 7.1 Hz, 3H, CH_3), 1.12 (s, 6H, CH_3) ppm; ^{13}C NMR (101 MHz, $CDCl_3$): δ = 198.1, 171.6, 167.6, 146.0, 134.4, 132.5 (2C), 132.2 (2C), 127.5, 123.7, 117.5, 112.5, 62.4, 51.3, 46.4, 36.0, 35.7, 34.0, 28.6 (2C), 14.1 ppm; HRMS (ESI): *m/z* calcd for $C_{22}H_{24}BrNO_5$-H^+: 460.0765 [*M*-H]$^+$; found: 460.0760; elemental analysis calcd (%) for $C_{22}H_{24}BrNO_5$: C, 57.15; H, 5.23; N, 3.03; found: %: C, 57.15; H, 5.22; N, 3.01.

2-(1-(2-Ethoxy-2-oxoethyl)-6,6-dimethyl-2-(4-nitrophenyl)-4-oxo-4,5,6,7-tetrahydro-1H-indol-3-yl)acetic acid (**15c**)

R_f=0.46 (DCM/MeOH 12/1); m.p. 152-155°C; ^{1}H NMR (400 MHz, $CDCl_3$): δ = 12.23 (s, 1H, OH), 8.27 (d, ^3J = 8.9 Hz, 2H, Ar-H), 7.50 (d, ^3J = 8.9 Hz, 2H, Ar-H), 4.41 (s, 2H, CH_2), 4.21 (q, ^3J = 7.1 Hz, 2H, CH_2CH_3), 3.42 (s, 2H, CH_2), 2.56 (s, 2H, CH_2), 2.46 (s, 2H, CH_2), 1.23 (t, ^3J = 7.1 Hz, 3H, CH_3), 1.13 (s, 6H, CH_3) ppm; ^{13}C NMR (101 MHz, $CDCl_3$): 198.2, 171.4, 167.4, 148.0, 146.8, 135.2, 133.2, 131.6 (2C), 124.1 (2C), 117.8, 113.9, 62.6, 51.4, 46.6, 36.0, 35.7, 34.0, 28.6 (2C), 14.2 ppm; HRMS (ESI): *m/z* calcd for $C_{22}H_{24}N_2O_7$+Na^+: 451.1476 [*M*+Na]$^+$; found: 451.1483; elemental analysis calcd (%) for $C_{22}H_{24}N_2O_7$: C, 61.68; H, 5.65; N, 6.54; found: %: C, 61.66; H, 5.65; N, 6.55.

2-(2-(4-Bromophenyl)-1-(2-ethoxy-2-oxoethyl)-4-oxo-4,5,6,7-tetrahydro-1H-indol-3-yl)acetic acid (**15d**)

R_f=0.60 (DCM/MeOH 12/1); ^{1}H NMR (500 MHz, $CDCl_3$): δ = 11.96 (s, 1H, OH), 7.65 (d, ^3J = 8.1 Hz, 2H, Ar-H), 7.14 (d, ^3J = 8.1 Hz, 2H, Ar-H), 4.63 (s, 2H, CH_2), 4.09 (q, ^3J = 7.1 Hz, 2H, CH_2), 3.40 (s, 2H, CH_2), 2.71 (d, ^3J = 6.2 Hz, 2H, CH_2), 2.32 (d, ^3J = 6.5 Hz, 2H, CH_2), 2.10-1.97 (m, 2H, CH_2), 1.12 (t, J = 7.1 Hz, 3H, CH_3) ppm; ^{13}C NMR (125 MHz, $CDCl_3$): δ = 194.2, 172.9, 168.7, 145.2, 132.6 (2C), 132.2 (2C), 129.6, 122.4, 118.5, 113.7, 61.8, 46.4, 38.3, 31.2, 23.4, 21.7, 14.4 (2C) ppm; HRMS (ESI): *m/z* calcd for $C_{20}H_{20}BrNO_5$+Na^+: 458.0525 [*M*+Na]$^+$; found: 458.0380 [*M*+Na]$^+$; elemental analysis calcd (%) for $C_{20}H_{20}BrNO_5$: C, 55.31; H, 4.64; N, 3.23; found: %: C, 55.36; H, 4.58; N, 3.29.

2-(1-(2-Ethoxy-2-oxoethyl)-4-oxo-2-phenyl-4,5,6,7-tetrahydro-1H-indol-3-yl)acetic acid (**15e**)

R_f=0.56 (DCM/MeOH 12/1); ^{1}H NMR (500 MHz, $CDCl_3$): δ = 11.93 (s, 1H, OH), 7.49-7.35 (m, 3H, Ar-H), 7.19 (d, ^3J = 6.7 Hz, 2H, Ar-H), 4.61 (s, 2H, CH_2), 4.09 (q, ^3J = 7.1 Hz, 2H, CH_2), 3.39 (s, 2H, CH_2), 2.72 (d, ^3J = 6.2 Hz, 2H, CH_2), 2.33 (t, ^3J = 6.3 Hz, 2H, CH_2), 2.04 (t, ^3J = 6.4 Hz, 2H, CH_2), 1.12 (t, J = 7.1 Hz, 3H, CH_3) ppm; ^{13}C NMR (125 MHz, $CDCl_3$): δ = 194.2, 173.0, 168.8, 145.0, 133.7, 130.6 (2C), 130.3, 129.2 (2C), 128.9, 118.5, 113.2, 61.7, 46.4, 38.3, 31.3, 23.5, 21.7, 14.4 ppm; HRMS (ESI): *m/z* calcd for $C_{20}H_{21}NO_5$+Na^+: 378.1420 [M+Na]$^+$; found: 378.1309 [M+Na]$^+$; elemental analysis calcd (%) for $C_{20}H_{21}NO_5$: C, 67.59; H, 5.96; N, 3.94; found: %: C, 67.60; H, 5.90; N, 3.91.

10. General Procedure for the Synthesis of Compounds 16a-c

Arylglyoxal monohydrate **2** (1.0 mmol), Meldrum's acid **14** (1.0 mmol), and the corresponding enamine **13** (1.0 mmol) were dissolved in ethanol (5 mL) and the reaction mixture was stirred under reflux for 2 h. Reaction progress was monitored by thin-layer chromatography (TLC). Upon completion, the mixture was cooled to room temperature and the solvent was removed under reduced pressure. The crude residue was purified by flash column chromatography on silica gel using a mixture of cyclohexane/ethyl acetate (10:4, v/v) as the eluent to afford the desired compounds **16a-c** as white powders.

Diethyl 2,2'-(6,6-dimethyl-4-oxo-2-phenyl-4,5,6,7-tetrahydro-1H-indole-1,3-diyl)diacetate (**16a**)

R_f=0.56 (DCM/MeOH 12/1); ^{1}H NMR (400 MHz, $CDCl_3$): δ = 7.37-7.30 (m, 3H, Ar-H), 7.22-7.15 (m, 2H, Ar-H), 4.33 (s, 2H, CH_2), 4.15 (q, ^3J = 7.1 Hz, 2H, CH_2), 4.05 (q, ^3J = 7.1 Hz, 2H, CH_2), 3.54 (s, 2H, CH_2), 2.49 (s, 2H, CH_2), 2.29 (s, 2H, CH_2), 1.21-1.16 (m, 3H, CH_3), 1.18-1.13 (m, 3H, CH_3),1.08 (s, 6H, CH_3) ppm; ^{13}C NMR (101 MHz, $CDCl_3$): δ = 194.0, 172.3, 168.2, 142.8, 134.2, 130.7 (2C), 130.1, 128.7 (2C), 128.6, 117.9, 113.2, 61.9, 60.4, 52.3, 46.2, 36.1, 35.4, 31.2, 28.8 (2C), 14.3, 14.1 ppm; HRMS (ESI): *m/z* calcd for $C_{24}H_{29}NO_5$+H^+: 412.2119 [M+H]$^+$; found: 412.2119.

Diethyl 2,2'-(2-(4-bromophenyl)-6,6-dimethyl-4-oxo-4,5,6,7-tetrahydro-1H-indole-1,3-diyl)diacetate (**16b**)

R_f=0.24 (petroleum ether/EE 1/1); ^{1}H NMR (400 MHz, $CDCl_3$): δ = 7.48 (d, ^3J=8.6 Hz, 2H, Ar-H), 7.08 (d, ^3J = 8.6 Hz, 2H, Ar-H), 4.31 (s, 2H, CH_2), 4.16 (q, ^3J = 7.1 Hz, 2H, CH_2), 4.05 (q, ^3J = 7.1 Hz, 2H, CH_2), 3.52 (s, 2H, CH_2), 2.48 (s, 2H, CH_2), 2.29 (s, 2H, CH_2), 1.22-1.18 (m, 3H, CH_3), 1.18-1.15 (m, 3H, CH_3), 1.08 (s, 6H, CH_3) ppm; ^{13}C NMR (101 MHz, $CDCl_3$): δ = 193.9, 172.1, 168.1, 143.1, 132.9, 132.3 (2C), 132.0 (2C), 129.0, 123.0, 117.9,

113.6, 62.0, 60.5, 52.3, 46.1, 36.0, 35.3, 31.1, 28.8 (2C), 14.3, 14.2 ppm; HRMS (ESI): *m/z* calcd for $C_{24}H_{28}BrNO_5+H^+$: 490.1224 [*M*+H]$^+$; found: 490.1230.

Diethyl 2,2'-(2-(4-chlorophenyl)-6,6-dimethyl-4-oxo-4,5,6,7-tetrahydro-1H-indole-1,3-diyl)diacetate (**16c**)

R_f=0.63 (DCM/MeOH 12/1); ^{1}H NMR (400 MHz, $CDCl_3$): δ = 7.32 (d, ^3J = 8.7 Hz, 2H, Ar-H), 7.14 (d, ^3J = 8.7 Hz, 2H, Ar-H), 4.31 (s, 2H, CH_2), 4.15 (q, ^3J = 7.1 Hz, 2H, CH_2), 4.05 (q, ^3J = 7.1 Hz, 2H, CH_2), 3.52 (s, 2H, CH_2), 2.48 (s, 2H, CH_2), 2.29 (s, 2H, CH_2), 1.23-1.19 (m, 3H, CH_3), 1.18-1.13 (m, 3H, CH_3), 1.08 (s, 6H, CH_3) ppm; ^{13}C NMR (101 MHz, $CDCl_3$): δ = 194.3, 172.2, 168.1, 143.3, 134.9, 133.0, 132.0 (2C), 129.0 (2C), 128.5, 117.8, 113.6, 62.0, 60.6, 52.1, 46.1, 36.0, 35.4, 31.1, 28.8 (2C), 14.2, 14.1 ppm; HRMS (ESI): *m/z* calcd for $C_{24}H_{28}ClNO_5+H^+$: 446.1729 [*M*+H]$^+$; found: 446.1725; *m/z* calcd for $C_{24}H_{28}ClNO_5-H^+$: 444.1583[*M*-H]$^+$; found: 444.1578.

11. General Procedure for the Synthesis of Compounds 17d,e

p-Chlorophenylglyoxal monohydrate (1.0 mmol), β-enaminoketone **1** (1.0 mmol) and acetic acid (20 μL) were dissolved in ethanol and stirred at reflux for 2 hours. Reaction progress was monitored by thin-layer chromatography (TLC). After completion, the reaction mixture was cooled to room temperature, and the resulting precipitate was collected by filtration, washed with cold ethanol, and dried in air to afford compound **17d** as a light yellow powder.

2-(2-(4-chlorophenyl)-7-ethoxy-6,6-dimethyl-4-oxo-4,5,6,7-tetrahydro-1H-indol-1-yl)acetic acid (**17d**)

R_f=0.56 (DCM/MeOH 12/1); ^{1}H NMR (300 MHz, $CDCl_3$): δ = 7.33 (d, ^3J = 8.5 Hz, 2H, Ar-H), 7.15 (d, ^3J = 8.5 Hz, 2H, Ar-H), 6.50 (s, 1H, CH), 4.62 (d, ^2J = 7.0 Hz, 2H, CH_2), 4.35 (d, ^4J = 5.1 Hz, 1H, CH), 4.19 (q, ^3J = 7.2 Hz, 2H, CH_2), 2.68 (d, ^2J = 16.4 Hz, 1H, CH), 2.20 (d, ^2J = 16.4 Hz, 1H, CH), 1.24-1.19 (m, 6H, CH_3), 1.14 (t, 3H, ^3J = 7.2 Hz, CH_3) ppm; HRMS (ESI): *m/z* calcd for $C_{20}H_{22}ClNO_4+H^+$: 376.1237 [*M*+H]$^+$; found: 376.1312; *m/z* calcd for $C_{20}H_{22}ClNO_4-H^+$: 376.1237 [*M*-H]$^+$; found: 374.1152; elemental analysis calcd (%) for $C_{20}H_{22}ClNO_4$: C, 63.91; H, 5.90; N, 3.73; found: %: C, 63.92; H, 5.90; N, 3.72.

The synthesis of compound **17e** was previously reported in the literature, and its physico-chemical characteristics correspond to those reported.[8]

12. General Procedure for the Synthesis of Compounds 19a-c

An equimolar mixture of arylglyoxal monohydrate (1.0 mmol) and Meldrum's acid (1.0 mmol) were taken in a 25-mL round-bottomed flask containing 10 mL of ethanol. The contents were stirred magnetically in an oil-bath maintained at 80°C for 1 h. The 6-amino-1,3-dimethyluracil (1.0 mmol) was added, and the progress of the reaction was monitored by TLC (eluent: DCM/MeOH=12/1 or petroleum ether/EE 1/1). After the completion of reaction, reaction mixture was cooled to room temperature and evaporated. The residue was purified by *Flash Chromatography System PuriFlash® 430* to obtain derivatives **19a-c** as white powders.

Ethyl 2-(6-(4-chlorophenyl)-1,3-dimethyl-2,4-dioxo-2,3,4,7-tetrahydro-1H-pyrrolo[2,3-d]pyrimidin-5-yl)acetate (**19a**)

R_f=0.25 (petroleum ether/EE 1/1); m.p. 252-254°C; ^{1}H NMR (400 MHz, DMSO-d_6): δ = 11.70 (s, 1H, NH), 7.53 (d, ^3J = 8.6 Hz, 2H, Ar-H), 7.46 (d, ^3J = 8.6 Hz, 2H, Ar-H), 4.04 (q, ^3J = 7.1 Hz, 2H, CH_2), 3.78 (s, 2H, CH_2), 3.48 (s, 3H, CH_3), 3.19 (s, 3H, CH_3), 1.14 (t, ^3J = 7.1 Hz, 3H, CH_3) ppm; ^{13}C NMR (101 MHz, DMSO-d_6): δ = 171.0, 158.7, 150.6, 139.3, 131.9, 129.9, 129.2 (2C), 128.7 (2C), 126.9, 110.2, 98.7, 60.1, 30.6, 30.3, 27.3, 14.1 ppm; HRMS (ESI): *m/z* calcd for $C_{18}H_{18}ClN_3O_4$+Na^+: 398.0879 [M+Na]$^+$; found: 398.0871 [M+Na]$^+$.

Isopropyl 2-(6-(4-chlorophenyl)-1,3-dimethyl-2,4-dioxo-2,3,4,7-tetrahydro-1H-pyrrolo[2,3-d]pyrimidin-5-yl)acetate (**19b**)

R_f=0.53 (DCM/MeOH 12/1); m.p. >300°C; ^{1}H NMR (400 MHz, DMSO-d_6): δ = 11.69 (s, 1H, NH), 7.52 (d, ^3J = 8.6 Hz, 2H, Ar-H), 7.46 (d, ^3J = 8.6 Hz, 2H, Ar-H), 4.86 (p, ^3J = 6.3 Hz, 1H, *CH*(CH_3)$_2$), 3.76 (s, 2H, CH_2), 3.49 (s, 3H, CH_3), 3.19 (s, 3H, CH_3), 1.14 (d, ^3J = 6.3 Hz, 6H, (CH_3)$_2$) ppm; ^{13}C NMR (101 MHz, DMSO-d_6): δ = 170.4, 158.7, 150.6, 139.3, 131.8, 130.0, 129.2 (2C), 128.6 (2C), 126.8, 110.3, 98.7, 67.4, 30.6, 30.5, 27.3, 21.6 (2C) ppm; HRMS (ESI): *m/z* calcd for $C_{19}H_{20}ClN_3O_4$+Na^+: 412.1040 [M+Na]$^+$; found: 412.1041 [M+Na]$^+$.

Ethyl 2-(1,3-dimethyl-6-(4-nitrophenyl)-2,4-dioxo-2,3,4,7-tetrahydro-1H-pyrrolo[2,3-d]pyrimidin-5-yl)acetate (**19c**)

R_f=0.31 (DCM/MeOH 12/1); m.p. >300°C; ^{1}H NMR (400 MHz, DMSO-d_6): δ = 11.88 (s, 1H, NH), 8.30 (d, ^3J = 8.8 Hz, 2H, Ar-H), 7.72 (d, ^3J = 8.8 Hz, 2H, Ar-H), 4.06 (q, ^3J = 7.1

Hz, 2H, CH_2), 3.92 (s, 2H, CH_2), 3.51 (s, 3H, CH_3), 3.20 (s, 3H, CH_3), 1.15 (t, ^3J = 7.1 Hz, 3H, CH_3) ppm; ^{13}C NMR (101 MHz, DMSO-d_6): δ = 170.6, 158.7, 150.6, 145.6, 140.2, 137.6, 127.7 (2C), 126.0, 123.9 (2C), 113.2, 99.4, 60.3, 30.7, 30.5, 27.4, 14.0 ppm; HRMS (ESI): *m/z* calcd for $C_{18}H_{18}N_4O_6$+Na^+: 409.1124 [*M*+Na]$^+$; found: 409.1120 [*M*+Na]$^+$.

13. General Procedure for the Synthesis of Compounds 20a-c

An equimolar mixture of 6-amino-1,3-dimethyluracil (1.0 mmol), arylglyoxal monohydrate (1.0 mmol) and Meldrum's acid (1.0 mmol) were taken in a 25-mL round-bottomed flask containing 10 mL of ethanol. The contents were stirred magnetically in an oil-bath maintained at 80°C and the progress of the reaction was monitored by TLC (eluent: DCM/MeOH=12/1). After 2.5-3h of refluxing, precipitate appeaded in the solution. The flask was cooled, and the precipitate was filtrated, washed with EtOH, and dried to obtain compounds **20a-c** as transparent oils.

5-(6-Amino-1,3-dimethyl-2,4-dioxo-1,2,3,4-tetrahydropyrimidin-5-yl)-6-(3-chlorophenyl)-1,3-dimethyl-1,7-dihydro-2H-pyrrolo[2,3-d]pyrimidine-2,4(3H)-dione (**20a**)

R_f=0.24 (DCM/MeOH 12/1); m.p. >300°C; ^{1}H NMR (400 MHz, DMSO-d_6): δ = 11.66 (s, 1H, NH), 7.59 (s, 1H, Ar-H), 7.44 (d, ^3J = 7.9 Hz, 1H, Ar-H), 7.36 (td, ^3J = 7.9 Hz, ^2J = 0.4 Hz, 1H, Ar-H), 7.27 (d, ^3J = 7.9 Hz, 1H, Ar-H), 6.18 (s, 2H, NH_2), 3.55 (s, 3H, CH_3), 3.32 (s, 3H, CH_3), 3.19 (s, 3H, CH_3), 3.17 (s, 3H, CH_3) ppm; ^{13}C NMR (101 MHz, DMSO-d_6): δ = 161.1 (2C), 158.2, 151.7, 140.6, 134.4, 133.4, 130.6, 127.9 (2C), 126.7, 126.5, 125.3, 110.2, 80.9, 30.5, 28.1 (2C), 27.8 (2C) ppm; HRMS (ESI): *m/z* calcd for $C_{20}H_{19}ClN_6O_4$+H^+: 443.1230 [*M*+H]$^+$; found: 443.1232, *m/z* calcd for $C_{20}H_{19}ClN_6O_4$-H^+: 441.1083 [*M*-H]$^+$; found: 441.1071 [*M*-H]$^+$.

5-(6-Amino-1,3-dimethyl-2,4-dioxo-1,2,3,4-tetrahydropyrimidin-5-yl)-6-(4-fluorophenyl)-1,3-dimethyl-1,7-dihydro-2H-pyrrolo[2,3-d]pyrimidine-2,4(3H)-dione (**20b**)

R_f=0.31 (DCM/MeOH 12/1); m.p. >300°C; ^{1}H NMR (400 MHz, DMSO-d_6): δ = 11.62 (s, 1H, NH), 7.53 (dd, ^3J = 8.9 Hz, ^2J = 5.6 Hz, 2H, Ar-H), 7.22 (t, ^3J = 9.0 Hz, 2H, Ar-H), 6.14 (s, 2H, NH_2), 3.55 (s, 3H, CH_3), 3.30 (s, 3H, CH_3), 3.17 (s, 3H, CH_3), 3.14 (s, 3H, CH_3), ppm; ^{13}C NMR (101 MHz, DMSO-d_6): δ = 161.2, 158.3, 151.7, 151.3, 140.2, 129.1 (2C), 129.0, 115.7 (2C), 115.5, 108.8, 99.6, 81.0, 31.0, 30.4, 28.1 (2C), 27.8 (2C) ppm; HRMS (ESI): *m/z* calcd for $C_{20}H_{19}FN_6O_4$-H^+: 425.1379 [*M*-H]$^+$; found: 425.1393 [*M*-H]$^+$.

5-(6-Amino-1,3-dimethyl-2,4-dioxo-1,2,3,4-tetrahydropyrimidin-5-yl)-6-(4-nitrophenyl)-1,3-dimethyl-1,7-dihydro-2H-pyrrolo[2,3-d]pyrimidine-2,4(3H)-dione (**20c**)

R_f=0.46 (DCM/MeOH 12/1); m.p. >300°C; ^{1}H NMR (400 MHz, DMSO-d_6): δ = 11.79 (s, 1H, NH), 8.17 (d, ^3J = 9.0 Hz, 2H, Ar-H), 7.75 (d, ^3J = 9.0 Hz, 2H, Ar-H), 6.25 (s, 2H, NH_2), 3.54 (s, 3H, CH_3), 3.30 (s, 3H, CH_3) 3.16 (s, 3H, CH_3), 3.14 (s, 3H, CH_3) ppm; ^{13}C NMR (101 MHz, DMSO-d_6): δ = 160.4, 157.6, 152.7, 151.2, 150.7, 144.8, 126.2 (2C), 125.4, 123.7 (2C), 112.8, 100.2, 81.4, 30.6, 30.0, 27.6 (2C), 27.3 (2C) ppm; HRMS (ESI): *m/z* calcd for $C_{20}H_{19}N_7O_6$-H^+: 452.1324 [*M-H*]$^+$; found: 452.1318.

X-Ray Diffraction Data

Crystal Data for **19a** derivative

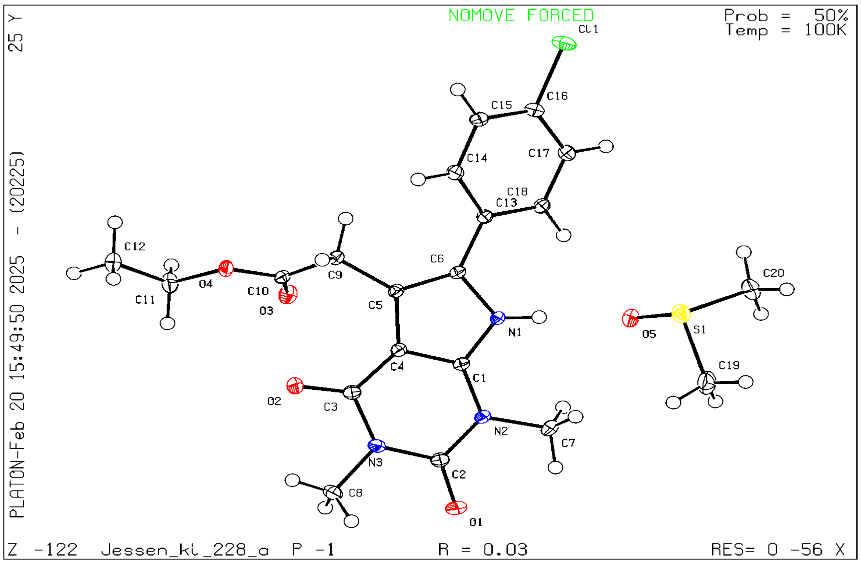

checkCIF/PLATON report

Structure factors have been supplied for datablock(s) Jessen_ki_228_a

THIS REPORT IS FOR GUIDANCE ONLY. IF USED AS PART OF A REVIEW PROCEDURE FOR PUBLICATION, IT SHOULD NOT REPLACE THE EXPERTISE OF AN EXPERIENCED CRYSTALLOGRAPHIC REFEREE.

No syntax errors found. CIF dictionary Interpreting this report

Datablock: Jessen_ki_228_a

```
Bond precision:    C-C = 0.0017 A            Wavelength=0.71073

Cell:          a=9.114(4)          b=10.280(4)         c=11.832(5)
               alpha=104.161(11)   beta=100.249(13)    gamma=96.655(18)
Temperature:   100 K

                  Calculated                   Reported
Volume            1042.9(8)                    1042.9(7)
Space group       P -1                         P -1
Hall group        -P 1                         -P 1
Moiety formula    C18 H18 Cl N3 O4, C2 H6 O S  C18 H18 Cl N3 O4, C2 H6 O S
Sum formula       C20 H24 Cl N3 O5 S           C20 H24 Cl N3 O5 S
Mr                453.93                       453.93
Dx,g cm-3         1.446                        1.446
Z                 2                            2
Mu (mm-1)         0.321                        0.321
F000              476.0                        476.0
F000'             476.75
h,k,lmax          13,14,16                     13,14,16
Nref              6393                         6373
Tmin,Tmax         0.924,0.973                  0.687,0.746
Tmin'             0.825

Correction method= # Reported T Limits: Tmin=0.687 Tmax=0.746
AbsCorr = MULTI-SCAN

Data completeness= 0.997             Theta(max)= 30.563

R(reflections)= 0.0327( 5560)                       wR2(reflections)=
                                                    0.0879( 6373)
S = 1.029                  Npar= 276
```

Table 1. Crystal data and structure refinement for Jessen_ki_228_a.

CCDC number	2425672
Empirical formula	$C_{20}H_{24}ClN_3O_5S$
Formula weight	453.93
Temperature [K]	100(2)
Crystal system	triclinic
Space group (number)	$P\bar{1}$ (2)
a [Å]	9.114(4)
b [Å]	10.280(4)
c [Å]	11.832(5)
α [°]	104.161(11)
β [°]	100.249(13)
γ [°]	96.655(18)
Volume [Å^3]	1042.9(7)
Z	2
ρ_{calc} [gcm^{-3}]	1.446
μ [mm^{-1}]	0.321
F(000)	476

Crystal size [mm^3]	0.084×0.204×0.601
Crystal colour	colourless
Crystal shape	block
Radiation	MoK_α (λ=0.71073 Å)
2θ range [°]	3.63 to 61.13 (0.70 Å)
Index ranges	−13 ≤ h ≤ 13<br>−14 ≤ k ≤ 14<br>−16 ≤ l ≤ 16
Reflections collected	48061
Independent reflections	6373<br>R_{int} = 0.0357<br>R_{sigma} = 0.0214
Completeness to<br>θ = 25.242°	100.0 %
Data / Restraints / Parameters	6373 / 0 / 276
Absorption correction T_{min}/T_{max}<br>(method)	0.6870 / 0.7461<br>(multi-scan)
Goodness-of-fit on F^2	1.029
Final *R* indexes<br>[I≥2σ(I)]	R_1 = 0.0327<br>wR_2 = 0.0841
Final *R* indexes<br>[all data]	R_1 = 0.0389<br>wR_2 = 0.0879
Largest peak/hole [eÅ^{-3}]	0.48/−0.28

Table 2. Atomic coordinates and U_{eq} [Å^2] for Jessen_ki_228_a.

**Atom**	***x***	***y***	***z***	U_{eq}
Cl1	0.94894(3)	0.39821(3)	0.77780(3)	0.02076(7)
S1	0.36940(3)	0.78836(3)	0.85501(3)	0.01900(7)
O1	0.09546(9)	0.98637(9)	0.36915(8)	0.01909(17)
O2	0.52056(10)	0.92693(9)	0.22009(7)	0.01852(17)
O3	0.56273(9)	0.62691(9)	0.15850(7)	0.01832(16)
O4	0.78018(9)	0.75288(9)	0.15449(7)	0.01528(15)
O5	0.32568(10)	0.70662(9)	0.72600(8)	0.02137(18)
N1	0.45768(9)	0.76170(9)	0.53995(8)	0.01138(16)
H1	0.419442	0.744215	0.598945	0.014
N2	0.26324(10)	0.87628(9)	0.46062(8)	0.01246(17)
N3	0.30820(10)	0.95414(9)	0.29624(8)	0.01318(17)
C1	0.39634(11)	0.82699(10)	0.46222(9)	0.01098(18)
C2	0.21418(12)	0.94150(11)	0.37542(9)	0.01344(19)
C3	0.44701(12)	0.90654(11)	0.29380(9)	0.01286(19)
C4	0.48826(11)	0.83741(11)	0.38262(9)	0.01158(18)

C5	0.61416(11)	0.77097(11)	0.41252(9)	0.01154(18)
C6	0.59349(11)	0.72628(11)	0.51052(9)	0.01121(18)
C7	0.17081(13)	0.85808(13)	0.54613(11)	0.0200(2)
H7A	0.076426	0.893089	0.527716	0.030
H7B	0.226269	0.907829	0.626954	0.030
H7C	0.147631	0.761086	0.541165	0.030
C8	0.25478(13)	1.02637(12)	0.20862(10)	0.0172(2)
H8A	0.336826	1.049380	0.169803	0.026
H8B	0.222612	1.109992	0.248975	0.026
H8C	0.169147	0.968070	0.148431	0.026
C9	0.73726(12)	0.75077(12)	0.34399(9)	0.0147(2)
H9A	0.794207	0.682638	0.369165	0.018
H9B	0.808401	0.837588	0.364322	0.018
C10	0.67900(12)	0.70380(11)	0.21007(10)	0.01372(19)
C11	0.74191(13)	0.71082(13)	0.02529(10)	0.0195(2)
H11A	0.654561	0.751150	−0.004783	0.023
H11B	0.715100	0.610501	−0.004082	0.023
C12	0.87928(14)	0.76016(13)	−0.01695(11)	0.0208(2)
H12A	0.856036	0.737910	−0.104295	0.031
H12B	0.963267	0.715687	0.009735	0.031
H12C	0.907803	0.858936	0.016246	0.031
C13	0.68150(11)	0.64968(10)	0.57898(9)	0.01145(18)
C14	0.83755(12)	0.65606(12)	0.58563(10)	0.0150(2)
H14	0.887649	0.714375	0.548194	0.018
C15	0.92043(12)	0.57872(12)	0.64594(10)	0.0159(2)
H15	1.025397	0.581685	0.647677	0.019
C16	0.84691(12)	0.49710(11)	0.70356(10)	0.01454(19)
C17	0.69433(12)	0.49389(11)	0.70438(9)	0.01356(19)
H17	0.646743	0.440982	0.747410	0.016
C18	0.61226(11)	0.56910(11)	0.64143(9)	0.01240(18)
H18	0.507482	0.566014	0.640586	0.015
C19	0.23394(19)	0.90020(14)	0.87442(13)	0.0306(3)
H19A	0.252989	0.971626	0.834585	0.046
H19B	0.242804	0.941762	0.959753	0.046
H19C	0.131825	0.848486	0.839562	0.046
C20	0.30762(15)	0.67701(13)	0.93805(11)	0.0223(2)
H20A	0.199736	0.640795	0.906865	0.033
H20B	0.323980	0.727436	1.022271	0.033
H20C	0.365257	0.601700	0.930586	0.033

U_{eq} is defined as 1/3 of the trace of the orthogonalized U_{ij} tensor.

Table 3. Anisotropic displacement parameters [Å^2] for Jessen_ki_228_a. The anisotropic displacement factor exponent takes the form: $-2\pi^2[\, h^2(a^*)^2U_{11} + k^2(b^*)^2U_{22} + \ldots + 2hka^*b^*U_{12}\,]$

Atom	U_{11}	U_{22}	U_{33}	U_{23}	U_{13}	U_{12}
Cl1	0.01792(13)	0.01984(14)	0.02657(15)	0.01219(11)	0.00009(10)	0.00587(10)

S1	0.01726(13)	0.02258(15)	0.01876(14)	0.00917(11)	0.00457(10)	0.00086(10)
O1	0.0142(4)	0.0226(4)	0.0229(4)	0.0081(3)	0.0042(3)	0.0083(3)
O2	0.0213(4)	0.0211(4)	0.0192(4)	0.0107(3)	0.0097(3)	0.0077(3)
O3	0.0175(4)	0.0189(4)	0.0182(4)	0.0040(3)	0.0059(3)	0.0006(3)
O4	0.0137(3)	0.0212(4)	0.0133(3)	0.0068(3)	0.0057(3)	0.0033(3)
O5	0.0255(4)	0.0262(5)	0.0183(4)	0.0088(3)	0.0118(3)	0.0106(4)
N1	0.0095(4)	0.0135(4)	0.0127(4)	0.0049(3)	0.0042(3)	0.0028(3)
N2	0.0095(4)	0.0147(4)	0.0147(4)	0.0050(3)	0.0042(3)	0.0036(3)
N3	0.0131(4)	0.0135(4)	0.0145(4)	0.0059(3)	0.0026(3)	0.0042(3)
C1	0.0093(4)	0.0103(4)	0.0132(4)	0.0027(4)	0.0023(3)	0.0019(3)
C2	0.0120(4)	0.0127(5)	0.0147(5)	0.0027(4)	0.0018(3)	0.0021(4)
C3	0.0131(4)	0.0110(4)	0.0141(4)	0.0028(4)	0.0026(3)	0.0024(4)
C4	0.0109(4)	0.0121(4)	0.0125(4)	0.0037(4)	0.0033(3)	0.0026(3)
C5	0.0100(4)	0.0125(5)	0.0126(4)	0.0035(4)	0.0033(3)	0.0023(3)
C6	0.0091(4)	0.0116(4)	0.0132(4)	0.0031(4)	0.0032(3)	0.0023(3)
C7	0.0143(5)	0.0292(6)	0.0235(6)	0.0130(5)	0.0106(4)	0.0087(4)
C8	0.0193(5)	0.0188(5)	0.0150(5)	0.0071(4)	0.0018(4)	0.0068(4)
C9	0.0114(4)	0.0215(5)	0.0140(5)	0.0070(4)	0.0051(4)	0.0054(4)
C10	0.0141(4)	0.0147(5)	0.0162(5)	0.0066(4)	0.0070(4)	0.0066(4)
C11	0.0209(5)	0.0242(6)	0.0132(5)	0.0054(4)	0.0050(4)	0.0007(4)
C12	0.0228(5)	0.0259(6)	0.0181(5)	0.0092(5)	0.0101(4)	0.0059(5)
C13	0.0114(4)	0.0112(4)	0.0116(4)	0.0028(4)	0.0026(3)	0.0019(3)
C14	0.0118(4)	0.0181(5)	0.0172(5)	0.0083(4)	0.0041(4)	0.0023(4)
C15	0.0110(4)	0.0205(5)	0.0178(5)	0.0075(4)	0.0029(4)	0.0042(4)
C16	0.0148(5)	0.0134(5)	0.0153(5)	0.0048(4)	0.0007(4)	0.0039(4)
C17	0.0143(5)	0.0114(5)	0.0147(5)	0.0041(4)	0.0027(4)	0.0004(4)
C18	0.0108(4)	0.0121(5)	0.0139(4)	0.0027(4)	0.0032(3)	0.0010(3)
C19	0.0514(9)	0.0242(6)	0.0272(7)	0.0114(5)	0.0247(6)	0.0166(6)
C20	0.0277(6)	0.0228(6)	0.0187(5)	0.0097(5)	0.0062(4)	0.0022(5)

Table 4. Bond lengths and angles for Jessen_ki_228_a.

Atom–Atom	Length [Å]
Cl1–C16	1.7422(12)
S1–O5	1.5077(11)
S1–C20	1.7863(13)
S1–C19	1.7887(15)
O1–C2	1.2219(14)
O2–C3	1.2324(13)
O3–C10	1.2082(14)
O4–C10	1.3445(13)
O4–C11	1.4483(14)
N1–C1	1.3472(13)
N1–C6	1.4078(14)
N1–H1	0.8800
N2–C1	1.3667(14)
N2–C2	1.3805(14)
N2–C7	1.4586(14)
N3–C2	1.3959(14)
N3–C3	1.4108(14)
N3–C8	1.4643(14)
C1–C4	1.3819(14)
C3–C4	1.4274(15)
C4–C5	1.4366(15)

C5–C6	1.3819(15)
C5–C9	1.5015(15)
C6–C13	1.4653(15)
C7–H7A	0.9800
C7–H7B	0.9800
C7–H7C	0.9800
C8–H8A	0.9800
C8–H8B	0.9800
C8–H8C	0.9800
C9–C10	1.5141(16)
C9–H9A	0.9900
C9–H9B	0.9900
C11–C12	1.5080(17)
C11–H11A	0.9900
C11–H11B	0.9900
C12–H12A	0.9800
C12–H12B	0.9800
C12–H12C	0.9800
C13–C14	1.4033(15)
C13–C18	1.4052(15)
C14–C15	1.3918(15)
C14–H14	0.9500
C15–C16	1.3892(16)
C15–H15	0.9500
C16–C17	1.3890(16)
C17–C18	1.3891(15)
C17–H17	0.9500
C18–H18	0.9500
C19–H19A	0.9800
C19–H19B	0.9800
C19–H19C	0.9800
C20–H20A	0.9800
C20–H20B	0.9800
C20–H20C	0.9800
**Atom–Atom–Atom**	**Angle [°]**
O5–S1–C20	105.72(6)
O5–S1–C19	105.80(7)
C20–S1–C19	98.23(7)
C10–O4–C11	116.25(9)
C1–N1–C6	108.21(8)

C1–N1–H1	125.9
C6–N1–H1	125.9
C1–N2–C2	119.56(9)
C1–N2–C7	120.79(9)
C2–N2–C7	119.64(9)
C2–N3–C3	126.85(9)
C2–N3–C8	115.01(9)
C3–N3–C8	118.13(9)
N1–C1–N2	125.89(9)
N1–C1–C4	109.91(9)
N2–C1–C4	124.20(10)
O1–C2–N2	122.32(10)
O1–C2–N3	121.39(10)
N2–C2–N3	116.29(9)
O2–C3–N3	120.20(10)
O2–C3–C4	126.24(10)
N3–C3–C4	113.55(9)
C1–C4–C3	119.51(10)
C1–C4–C5	106.98(9)
C3–C4–C5	133.50(9)
C6–C5–C4	106.46(9)
C6–C5–C9	128.51(10)
C4–C5–C9	124.97(9)
C5–C6–N1	108.42(9)
C5–C6–C13	131.88(9)
N1–C6–C13	119.66(9)
N2–C7–H7A	109.5
N2–C7–H7B	109.5
H7A–C7–H7B	109.5
N2–C7–H7C	109.5
H7A–C7–H7C	109.5
H7B–C7–H7C	109.5
N3–C8–H8A	109.5
N3–C8–H8B	109.5
H8A–C8–H8B	109.5
N3–C8–H8C	109.5
H8A–C8–H8C	109.5
H8B–C8–H8C	109.5
C5–C9–C10	113.40(9)
C5–C9–H9A	108.9
C10–C9–H9A	108.9
C5–C9–H9B	108.9

C10–C9–H9B	108.9
H9A–C9–H9B	107.7
O3–C10–O4	123.81(10)
O3–C10–C9	125.73(10)
O4–C10–C9	110.33(9)
O4–C11–C12	106.98(10)
O4–C11–H11A	110.3
C12–C11–H11A	110.3
O4–C11–H11B	110.3
C12–C11–H11B	110.3
H11A–C11–H11B	108.6
C11–C12–H12A	109.5
C11–C12–H12B	109.5
H12A–C12–H12B	109.5
C11–C12–H12C	109.5
H12A–C12–H12C	109.5
H12B–C12–H12C	109.5
C14–C13–C18	118.02(10)
C14–C13–C6	121.11(9)
C18–C13–C6	120.86(9)
C15–C14–C13	121.35(10)
C15–C14–H14	119.3
C13–C14–H14	119.3
C16–C15–C14	118.81(10)

C16–C15–H15	120.6
C14–C15–H15	120.6
C17–C16–C15	121.41(10)
C17–C16–Cl1	119.35(8)
C15–C16–Cl1	119.24(9)
C18–C17–C16	119.09(10)
C18–C17–H17	120.5
C16–C17–H17	120.5
C17–C18–C13	121.17(10)
C17–C18–H18	119.4
C13–C18–H18	119.4
S1–C19–H19A	109.5
S1–C19–H19B	109.5
H19A–C19–H19B	109.5
S1–C19–H19C	109.5
H19A–C19–H19C	109.5
H19B–C19–H19C	109.5
S1–C20–H20A	109.5
S1–C20–H20B	109.5
H20A–C20–H20B	109.5
S1–C20–H20C	109.5
H20A–C20–H20C	109.5
H20B–C20–H20C	109.5

Table 5. Torsion angles for Jessen_ki_228_a.

Atom–Atom–Atom–Atom	Torsion Angle [°]
C6–N1–C1–N2	179.29(10)
C6–N1–C1–C4	−0.66(12)
C2–N2–C1–N1	179.91(10)
C7–N2–C1–N1	−1.49(16)
C2–N2–C1–C4	−0.15(16)
C7–N2–C1–C4	178.46(11)
C1–N2–C2–O1	179.29(10)
C7–N2–C2–O1	0.67(16)
C1–N2–C2–N3	−0.91(14)
C7–N2–C2–N3	−179.54(10)
C3–N3–C2–O1	−179.73(10)
C8–N3–C2–O1	1.08(15)
C3–N3–C2–N2	0.47(16)
C8–N3–C2–N2	−178.72(9)
C2–N3–C3–O2	−178.58(10)
C8–N3–C3–O2	0.59(15)
C2–N3–C3–C4	0.98(15)
C8–N3–C3–C4	−179.85(9)
N1–C1–C4–C3	−178.33(9)
N2–C1–C4–C3	1.71(16)
N1–C1–C4–C5	1.20(12)
N2–C1–C4–C5	−178.75(10)
O2–C3–C4–C1	177.54(11)
N3–C3–C4–C1	−1.98(14)
O2–C3–C4–C5	−1.8(2)
N3–C3–C4–C5	178.63(11)
C1–C4–C5–C6	−1.27(12)
C3–C4–C5–C6	178.17(11)
C1–C4–C5–C9	176.06(10)
C3–C4–C5–C9	−4.49(19)
C4–C5–C6–N1	0.89(12)
C9–C5–C6–N1	−176.32(10)
C4–C5–C6–C13	178.34(11)
C9–C5–C6–C13	1.13(19)
C1–N1–C6–C5	−0.17(12)
C1–N1–C6–C13	−177.99(9)
C6–C5–C9–C10	132.84(12)
C4–C5–C9–C10	−43.90(15)
C11–O4–C10–O3	1.27(15)
C11–O4–C10–C9	177.35(9)
C5–C9–C10–O3	−36.10(16)
C5–C9–C10–O4	147.92(9)
C10–O4–C11–C12	−171.86(9)
C5–C6–C13–C14	27.23(18)
N1–C6–C13–C14	−155.55(10)
C5–C6–C13–C18	−153.71(11)
N1–C6–C13–C18	23.50(15)
C18–C13–C14–C15	3.91(16)
C6–C13–C14–C15	−177.01(10)
C13–C14–C15–C16	−2.03(17)
C14–C15–C16–C17	−1.54(17)
C14–C15–C16–Cl1	179.21(9)
C15–C16–C17–C18	3.07(17)
Cl1–C16–C17–C18	−177.68(8)
C16–C17–C18–C13	−1.08(16)
C14–C13–C18–C17	−2.33(16)
C6–C13–C18–C17	178.59(10)

Crystal Data for **19b** derivative

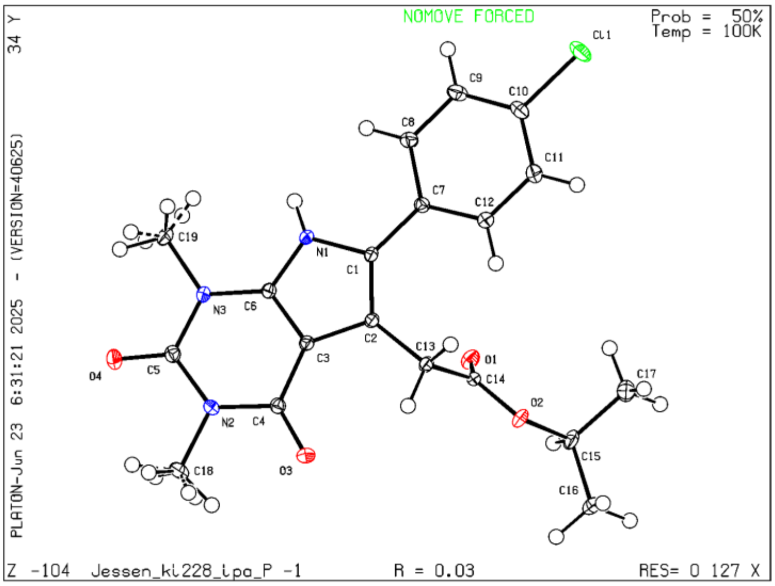

checkCIF/PLATON report

Structure factors have been supplied for datablock(s) Jessen_ki228_ipa_a

THIS REPORT IS FOR GUIDANCE ONLY. IF USED AS PART OF A REVIEW PROCEDURE FOR PUBLICATION, IT SHOULD NOT REPLACE THE EXPERTISE OF AN EXPERIENCED CRYSTALLOGRAPHIC REFEREE.

No syntax errors found. CIF dictionary Interpreting this report

Datablock: Jessen_ki228_ipa_a

```
Bond precision:   C-C = 0.0015 A          Wavelength=0.71073

Cell:           a=8.474(3)         b=8.884(3)        c=12.984(4)
                alpha=108.973(15)  beta=101.850(8)   gamma=100.420(15)
Temperature:    100 K

                 Calculated                  Reported
Volume           871.7(5)                    871.6(5)
Space group      P -1                        P -1
Hall group       -P 1                        -P 1
Moiety formula   C19 H20 Cl N3 O4            C19 H20 Cl N3 O4
Sum formula      C19 H20 Cl N3 O4            C19 H20 Cl N3 O4
Mr               389.83                      389.83
Dx,g cm-3        1.485                       1.485
Z                2                           2
Mu (mm-1)        0.252                       0.252
F000             408.0                       408.0
F000'            408.48
h,k,lmax         12,12,18                    12,12,18
Nref             5371                        5351
Tmin,Tmax        0.935,0.963                 0.705,0.746
Tmin'            0.920

Correction method= # Reported T Limits: Tmin=0.705 Tmax=0.746
AbsCorr = MULTI-SCAN

Data completeness= 0.996          Theta(max)= 30.607

                                              wR2(reflections)=
R(reflections)= 0.0324( 4858)                 0.0902( 5351)

S = 1.045                 Npar= 253
```

Table 1. Crystal data and structure refinement for Jessen_ki228_ipa_a.

CCDC number	2466312
Empirical formula	$C_{19}H_{20}ClN_3O_4$
Formula weight	389.83
Temperature [K]	100(2)
Crystal system	triclinic
Space group (number)	$P\bar{1}$ (2)
a [Å]	8.474(3)
b [Å]	8.884(3)
c [Å]	12.984(4)
α [°]	108.973(15)
β [°]	101.850(8)
γ [°]	100.420(15)
Volume [$Å^3$]	871.6(5)
Z	2
ρ_{calc} [gcm^{-3}]	1.485
μ [mm^{-1}]	0.252
F(000)	408
Crystal size [mm^3]	0.148×0.224×0.333
Crystal colour	colourless
Crystal shape	block
Radiation	MoK_α (λ=0.71073 Å)
2θ range [°]	3.46 to 61.21 (0.70 Å)
Index ranges	−12 ≤ h ≤ 12<br>−12 ≤ k ≤ 12<br>−18 ≤ l ≤ 18
Reflections collected	40656
Independent reflections	5351<br>R_{int} = 0.0217<br>R_{sigma} = 0.0125
Completeness to θ = 25.242°	99.9 %
Data / Restraints / Parameters	5351 / 3 / 253
Absorption correction T_{min}/T_{max} (method)	0.7046 / 0.7461 (multi-scan)
Goodness-of-fit on F^2	1.045
Final R indexes [$I≥2σ(I)$]	R_1 = 0.0324<br>wR_2 = 0.0875
Final R indexes [all data]	R_1 = 0.0357<br>wR_2 = 0.0902
Largest peak/hole [$eÅ^{-3}$]	0.50/−0.24

Table 2. Atomic coordinates and U_{eq} [$Å^2$] for Jessen_ki228_ipa_a.

**Atom**	***x***	***y***	***z***	**U_{eq}**

Cl1	0.81082(3)	0.65999(3)	0.14145(2)	0.02146(7)
O1	0.13686(9)	0.34001(9)	0.30807(6)	0.01487(14)
O2	0.00972(9)	0.10961(9)	0.15144(6)	0.01313(14)
O3	0.13804(9)	−0.07723(9)	0.43586(6)	0.01483(14)
O4	0.49305(9)	0.12420(10)	0.79611(6)	0.01669(15)
N1	0.61116(10)	0.34299(10)	0.52700(7)	0.01041(14)
H1N	0.6935(15)	0.4288(15)	0.5689(11)	0.012
N2	0.31174(10)	0.03224(10)	0.61761(7)	0.01192(15)
N3	0.56664(10)	0.23887(10)	0.67359(7)	0.01150(15)
C1	0.52332(11)	0.30725(11)	0.41339(7)	0.00986(16)
C2	0.37963(11)	0.18160(11)	0.38487(7)	0.00969(16)
C3	0.38100(11)	0.14084(11)	0.48290(8)	0.01001(16)
C4	0.26643(11)	0.02383(11)	0.50500(8)	0.01062(16)
C5	0.45930(12)	0.13138(12)	0.70189(8)	0.01191(17)
C6	0.52377(11)	0.24315(11)	0.56724(8)	0.00990(16)
C7	0.59362(11)	0.39378(11)	0.34758(8)	0.01061(16)
C8	0.74412(12)	0.52004(12)	0.39665(8)	0.01407(17)
H8	0.801448	0.550067	0.474383	0.017
C9	0.81138(13)	0.60228(12)	0.33423(9)	0.01576(18)
H9	0.912897	0.687842	0.369121	0.019
C10	0.72835(13)	0.55776(12)	0.22043(9)	0.01466(18)
C11	0.58050(13)	0.43217(13)	0.16823(9)	0.01701(19)
H11	0.525052	0.401600	0.090101	0.020
C12	0.51479(12)	0.35183(13)	0.23183(8)	0.01549(18)
H12	0.413567	0.265975	0.196056	0.019
C13	0.24136(11)	0.09517(11)	0.27645(8)	0.01110(16)
H13A	0.172927	−0.004627	0.280354	0.013
H13B	0.291275	0.058119	0.213144	0.013
C14	0.12715(11)	0.19879(11)	0.24961(7)	0.01024(16)
C15	−0.11691(12)	0.18558(13)	0.10980(8)	0.01431(18)
H15	−0.140142	0.266673	0.175140	0.017
C16	−0.27209(13)	0.04363(15)	0.04182(9)	0.0206(2)
H16A	−0.357294	0.083505	0.002972	0.031
H16B	−0.315814	−0.001382	0.093063	0.031
H16C	−0.243470	−0.043008	−0.014508	0.031
C17	−0.05192(14)	0.27152(13)	0.03758(9)	0.0196(2)
H17A	−0.032602	0.190355	−0.027279	0.029
H17B	0.053404	0.355717	0.083254	0.029
H17C	−0.134487	0.324145	0.010013	0.029
C18	0.19452(13)	−0.07427(13)	0.65008(9)	0.01718(19)
H18A	0.093735	−0.035116	0.651511	0.026
H18B	0.247642	−0.070860	0.725641	0.026
H18C	0.163853	−0.187993	0.594791	0.026
H18D	0.243086	−0.160863	0.663118	0.026
H18E	0.089178	−0.125119	0.588987	0.026
H18F	0.172967	−0.007986	0.719838	0.026

C19	0.72486(12)	0.34246(12)	0.75755(8)	0.01517(18)
H19A	0.818164	0.313499	0.729019	0.023
H19B	0.731358	0.324491	0.828717	0.023
H19C	0.731211	0.458872	0.771255	0.023
H19D	0.702324	0.417742	0.823642	0.023
H19E	0.789130	0.406750	0.723944	0.023
H19F	0.789277	0.272369	0.781406	0.023

U_{eq} is defined as 1/3 of the trace of the orthogonalized U_{ij} tensor.

Table 3. Anisotropic displacement parameters [Å^2] for Jessen_ki228_ipa_a. The anisotropic displacement factor exponent takes the form: $-2\pi^2[h^2(a^*)^2U_{11} + k^2(b^*)^2U_{22} + \ldots + 2hka^*b^*U_{12}]$

Atom	U_{11}	U_{22}	U_{33}	U_{23}	U_{13}	U_{12}
Cl1	0.02309(13)	0.02443(13)	0.02590(13)	0.01717(10)	0.01350(10)	0.00598(10)
O1	0.0143(3)	0.0129(3)	0.0137(3)	0.0029(3)	0.0000(2)	0.0033(2)
O2	0.0105(3)	0.0148(3)	0.0102(3)	0.0028(2)	−0.0017(2)	0.0031(2)
O3	0.0119(3)	0.0145(3)	0.0153(3)	0.0057(3)	0.0017(2)	−0.0009(2)
O4	0.0174(3)	0.0221(4)	0.0129(3)	0.0098(3)	0.0040(3)	0.0052(3)
N1	0.0092(3)	0.0106(3)	0.0094(3)	0.0039(3)	0.0002(3)	0.0005(3)
N2	0.0112(3)	0.0134(4)	0.0126(3)	0.0071(3)	0.0039(3)	0.0019(3)
N3	0.0108(3)	0.0131(3)	0.0096(3)	0.0052(3)	0.0008(3)	0.0015(3)
C1	0.0092(4)	0.0106(4)	0.0090(4)	0.0041(3)	0.0007(3)	0.0021(3)
C2	0.0089(4)	0.0099(4)	0.0094(4)	0.0036(3)	0.0011(3)	0.0021(3)
C3	0.0094(4)	0.0098(4)	0.0105(4)	0.0042(3)	0.0018(3)	0.0023(3)
C4	0.0105(4)	0.0110(4)	0.0118(4)	0.0054(3)	0.0034(3)	0.0038(3)
C5	0.0117(4)	0.0137(4)	0.0122(4)	0.0060(3)	0.0041(3)	0.0050(3)
C6	0.0096(4)	0.0104(4)	0.0101(4)	0.0045(3)	0.0024(3)	0.0032(3)
C7	0.0104(4)	0.0105(4)	0.0115(4)	0.0049(3)	0.0032(3)	0.0028(3)
C8	0.0134(4)	0.0129(4)	0.0136(4)	0.0047(3)	0.0027(3)	−0.0001(3)
C9	0.0154(4)	0.0132(4)	0.0180(4)	0.0060(3)	0.0062(3)	0.0002(3)
C10	0.0158(4)	0.0154(4)	0.0186(4)	0.0104(4)	0.0093(4)	0.0058(3)
C11	0.0154(4)	0.0231(5)	0.0146(4)	0.0106(4)	0.0043(3)	0.0036(4)
C12	0.0123(4)	0.0194(4)	0.0132(4)	0.0079(4)	0.0014(3)	−0.0003(3)
C13	0.0105(4)	0.0104(4)	0.0103(4)	0.0037(3)	0.0000(3)	0.0016(3)
C14	0.0083(4)	0.0128(4)	0.0090(4)	0.0051(3)	0.0014(3)	0.0005(3)
C15	0.0112(4)	0.0197(4)	0.0116(4)	0.0060(3)	0.0004(3)	0.0062(3)
C16	0.0105(4)	0.0318(6)	0.0162(4)	0.0103(4)	−0.0002(3)	−0.0001(4)
C17	0.0219(5)	0.0185(5)	0.0162(4)	0.0082(4)	0.0008(4)	0.0021(4)
C18	0.0154(4)	0.0195(5)	0.0207(5)	0.0125(4)	0.0075(4)	0.0022(4)
C19	0.0128(4)	0.0160(4)	0.0121(4)	0.0051(3)	−0.0020(3)	−0.0001(3)

Table 4. Bond lengths and angles for Jessen_ki228_ipa_a.

Atom–Atom	Length [Å]
Cl1–C10	1.7422(11)
O1–C14	1.2143(12)
O2–C14	1.3341(11)
O2–C15	1.4698(12)
O3–C4	1.2317(12)
O4–C5	1.2231(12)
N1–C6	1.3530(12)
N1–C1	1.4106(12)
N1–H1N	0.864(11)
N2–C5	1.3963(13)
N2–C4	1.4067(13)
N2–C18	1.4667(13)
N3–C6	1.3687(12)
N3–C5	1.3876(13)
N3–C19	1.4608(13)
C1–C2	1.3857(13)
C1–C7	1.4650(13)
C2–C3	1.4305(13)
C2–C13	1.4961(13)
C3–C6	1.3784(13)
C3–C4	1.4313(13)
C7–C8	1.4052(13)
C7–C12	1.4062(14)
C8–C9	1.3926(14)
C8–H8	0.9500
C9–C10	1.3882(15)
C9–H9	0.9500
C10–C11	1.3884(15)
C11–C12	1.3892(14)
C11–H11	0.9500
C12–H12	0.9500
C13–C14	1.5159(13)
C13–H13A	0.9900
C13–H13B	0.9900
C15–C17	1.5136(15)
C15–C16	1.5159(15)
C15–H15	1.0000
C16–H16A	0.9800
C16–H16B	0.9800
C16–H16C	0.9800
C17–H17A	0.9800
C17–H17B	0.9800
C17–H17C	0.9800
C18–H18A	0.9800
C18–H18B	0.9800
C18–H18C	0.9800
C18–H18D	0.9800
C18–H18E	0.9800
C18–H18F	0.9800
C19–H19A	0.9800
C19–H19B	0.9800
C19–H19C	0.9800
C19–H19D	0.9800
C19–H19E	0.9800
C19–H19F	0.9800
**Atom–Atom–Atom**	**Angle [°]**
C14–O2–C15	118.61(8)
C6–N1–C1	108.82(8)
C6–N1–H1N	124.4(9)
C1–N1–H1N	125.3(9)
C5–N2–C4	126.23(8)
C5–N2–C18	116.41(8)
C4–N2–C18	117.35(8)
C6–N3–C5	119.44(8)
C6–N3–C19	121.51(8)
C5–N3–C19	119.03(8)
C2–C1–N1	107.50(8)
C2–C1–C7	132.19(8)
N1–C1–C7	120.25(8)
C1–C2–C3	106.98(8)
C1–C2–C13	130.81(8)
C3–C2–C13	122.21(8)
C6–C3–C2	107.41(8)
C6–C3–C4	119.98(9)
C2–C3–C4	132.60(8)
O3–C4–N2	120.31(9)
O3–C4–C3	125.96(9)
N2–C4–C3	113.74(8)
O4–C5–N3	121.37(9)
O4–C5–N2	121.92(9)
N3–C5–N2	116.71(8)

N1–C6–N3	127.02(8)	C15–C17–H17A	109.5
N1–C6–C3	109.29(8)	C15–C17–H17B	109.5
N3–C6–C3	123.69(9)	H17A–C17–H17B	109.5
C8–C7–C12	117.09(9)	C15–C17–H17C	109.5
C8–C7–C1	121.57(8)	H17A–C17–H17C	109.5
C12–C7–C1	121.34(9)	H17B–C17–H17C	109.5
C9–C8–C7	121.68(9)	N2–C18–H18A	109.5
C9–C8–H8	119.2	N2–C18–H18B	109.5
C7–C8–H8	119.2	H18A–C18–H18B	109.5
C10–C9–C8	119.22(9)	N2–C18–H18C	109.5
C10–C9–H9	120.4	H18A–C18–H18C	109.5
C8–C9–H9	120.4	H18B–C18–H18C	109.5
C9–C10–C11	120.96(9)	N2–C18–H18D	109.5
C9–C10–Cl1	119.70(8)	H18A–C18–H18D	141.1
C11–C10–Cl1	119.34(8)	H18B–C18–H18D	56.3
C10–C11–C12	119.08(9)	H18C–C18–H18D	56.3
C10–C11–H11	120.5	N2–C18–H18E	109.5
C12–C11–H11	120.5	H18A–C18–H18E	56.3
C11–C12–C7	121.96(9)	H18B–C18–H18E	141.1
C11–C12–H12	119.0	H18C–C18–H18E	56.3
C7–C12–H12	119.0	H18D–C18–H18E	109.5
C2–C13–C14	114.74(8)	N2–C18–H18F	109.5
C2–C13–H13A	108.6	H18A–C18–H18F	56.3
C14–C13–H13A	108.6	H18B–C18–H18F	56.3
C2–C13–H13B	108.6	H18C–C18–H18F	141.1
C14–C13–H13B	108.6	H18D–C18–H18F	109.5
H13A–C13–H13B	107.6	H18E–C18–H18F	109.5
O1–C14–O2	124.33(9)	N3–C19–H19A	109.5
O1–C14–C13	126.24(8)	N3–C19–H19B	109.5
O2–C14–C13	109.41(8)	H19A–C19–H19B	109.5
O2–C15–C17	108.52(8)	N3–C19–H19C	109.5
O2–C15–C16	105.21(9)	H19A–C19–H19C	109.5
C17–C15–C16	112.31(9)	H19B–C19–H19C	109.5
O2–C15–H15	110.2	N3–C19–H19D	109.5
C17–C15–H15	110.2	H19A–C19–H19D	141.1
C16–C15–H15	110.2	H19B–C19–H19D	56.3
C15–C16–H16A	109.5	H19C–C19–H19D	56.3
C15–C16–H16B	109.5	N3–C19–H19E	109.5
H16A–C16–H16B	109.5	H19A–C19–H19E	56.3
C15–C16–H16C	109.5	H19B–C19–H19E	141.1
H16A–C16–H16C	109.5	H19C–C19–H19E	56.3
H16B–C16–H16C	109.5	H19D–C19–H19E	109.5

N3–C19–H19F	109.5
H19A–C19–H19F	56.3
H19B–C19–H19F	56.3
H19C–C19–H19F	141.1
H19D–C19–H19F	109.5
H19E–C19–H19F	109.5

Table 5. Torsion angles for Jessen_ki228_ipa_a.

Atom–Atom–Atom–Atom	Torsion Angle [°]
C6–N1–C1–C2	−0.34(10)
C6–N1–C1–C7	−177.70(8)
N1–C1–C2–C3	−0.05(10)
C7–C1–C2–C3	176.87(9)
N1–C1–C2–C13	−179.97(9)
C7–C1–C2–C13	−3.05(17)
C1–C2–C3–C6	0.42(10)
C13–C2–C3–C6	−179.66(8)
C1–C2–C3–C4	179.15(9)
C13–C2–C3–C4	−0.93(15)
C5–N2–C4–O3	174.78(9)
C18–N2–C4–O3	−4.44(13)
C5–N2–C4–C3	−5.49(13)
C18–N2–C4–C3	175.28(8)
C6–C3–C4–O3	−177.58(9)
C2–C3–C4–O3	3.82(17)
C6–C3–C4–N2	2.71(12)
C2–C3–C4–N2	−175.89(9)
C6–N3–C5–O4	−179.86(9)
C19–N3–C5–O4	1.46(14)
C6–N3–C5–N2	0.25(13)
C19–N3–C5–N2	−178.43(8)
C4–N2–C5–O4	−175.79(9)
C18–N2–C5–O4	3.44(14)
C4–N2–C5–N3	4.11(14)
C18–N2–C5–N3	−176.66(8)
C1–N1–C6–N3	179.87(9)
C1–N1–C6–C3	0.62(10)
C5–N3–C6–N1	178.08(9)
C19–N3–C6–N1	−3.27(14)
C5–N3–C6–C3	−2.76(14)
C19–N3–C6–C3	175.89(9)
C2–C3–C6–N1	−0.64(10)
C4–C3–C6–N1	−179.56(8)
C2–C3–C6–N3	−179.93(8)
C4–C3–C6–N3	1.15(14)
C2–C1–C7–C8	179.79(10)
N1–C1–C7–C8	−3.61(14)
C2–C1–C7–C12	−1.41(16)
N1–C1–C7–C12	175.19(9)
C12–C7–C8–C9	1.03(14)
C1–C7–C8–C9	179.88(9)
C7–C8–C9–C10	−0.44(15)
C8–C9–C10–C11	−0.45(15)
C8–C9–C10–Cl1	179.83(8)
C9–C10–C11–C12	0.69(16)
Cl1–C10–C11–C12	−179.59(8)
C10–C11–C12–C7	−0.06(16)
C8–C7–C12–C11	−0.78(15)
C1–C7–C12–C11	−179.63(9)
C1–C2–C13–C14	−71.26(13)
C3–C2–C13–C14	108.83(10)
C15–O2–C14–O1	0.24(14)
C15–O2–C14–C13	179.02(8)
C2–C13–C14–O1	−1.45(14)
C2–C13–C14–O2	179.80(8)
C14–O2–C15–C17	89.27(10)
C14–O2–C15–C16	−150.33(8)

Crystal Data for **20c** derivative

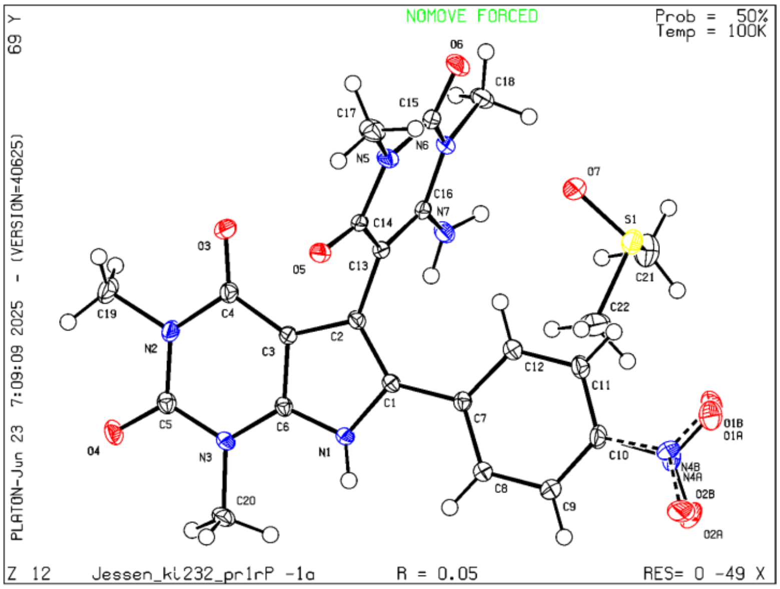

checkCIF/PLATON report

Structure factors have been supplied for datablock(s) Jessen_ki232_pr1recr_a

THIS REPORT IS FOR GUIDANCE ONLY. IF USED AS PART OF A REVIEW PROCEDURE FOR PUBLICATION, IT SHOULD NOT REPLACE THE EXPERTISE OF AN EXPERIENCED CRYSTALLOGRAPHIC REFEREE.

No syntax errors found. CIF dictionary Interpreting this report

Datablock: Jessen_ki232_pr1recr_a

```
Bond precision:   C-C = 0.0021 A          Wavelength=0.71073

Cell:          a=8.482(5)         b=10.226(4)      c=14.258(7)
               alpha=86.108(11)   beta=85.731(17)  gamma=72.10(2)
Temperature:   100 K

                Calculated                   Reported
Volume          1172.3(10)                   1172.3(10)
Space group     P -1                         P -1
Hall group      -P 1                         -P 1
Moiety formula  C20 H19 N7 O6, C2 H6 O S     C20 H19 N7 O6, C2 H6 O S
Sum formula     C22 H25 N7 O7 S              C22 H25 N7 O7 S
Mr              531.55                       531.55
Dx,g cm-3       1.506                        1.506
Z               2                            2
Mu (mm-1)       0.199                        0.199
F000            556.0                        556.0
F000'           556.51
h,k,lmax        12,14,20                     12,14,20
Nref            7203                         7195
Tmin,Tmax       0.989,0.992                  0.707,0.746
Tmin'           0.950

Correction method= # Reported T Limits: Tmin=0.707 Tmax=0.746
AbsCorr = MULTI-SCAN

Data completeness= 0.999          Theta(max)= 30.602

                                                wR2(reflections)=
R(reflections)= 0.0450( 5235)                   0.1174( 7195)

S = 1.034                 Npar= 377
```

Table 1. Crystal data and structure refinement for Jessen_ki232_pr1recr_a.

CCDC number	2491896
Empirical formula	$C_{22}H_{25}N_7O_7S$
Formula weight	531.55
Temperature [K]	100(2)
Crystal system	triclinic
Space group (number)	$P\bar{1}$ (2)
a [Å]	8.482(5)
b [Å]	10.226(4)
c [Å]	14.258(7)
α [°]	86.108(11)
β [°]	85.731(17)
γ [°]	72.10(2)
Volume [$Å^3$]	1172.3(10)
Z	2
ρ_{calc} [gcm^{-3}]	1.506
μ [mm^{-1}]	0.199
F(000)	556
Crystal size [mm^3]	0.042×0.047×0.260
Crystal colour	yellow
Crystal shape	needle
Radiation	MoK_{α} (λ=0.71073 Å)
2θ range [°]	2.87 to 61.20 (0.70 Å)
Index ranges	$-12 \leq h \leq 12$<br>$-14 \leq k \leq 14$<br>$-20 \leq l \leq 20$
Reflections collected	54490
Independent reflections	7195<br>R_{int} = 0.0653<br>R_{sigma} = 0.0424
Completeness to<br>θ = 25.242°	99.9 %
Data / Restraints / Parameters	7195 / 102 / 377
Absorption correction<br>T_{min}/T_{max} (method)	0.7073 / 0.7461<br>(multi-scan)
Goodness-of-fit on F^2	1.034
Final R indexes<br>[$I \geq 2\sigma(I)$]	R_1 = 0.0450<br>wR_2 = 0.1059
Final R indexes<br>[all data]	R_1 = 0.0693<br>wR_2 = 0.1174
Largest peak/hole [$eÅ^{-3}$]	0.47/−0.58

Table 2. Atomic coordinates and U_{eq} [$Å^2$] for Jessen_ki232_pr1recr_a.

**Atom**	***x***	***y***	***z***	U_{eq}
O3	0.02067(15)	0.85601(12)	0.42854(8)	0.0214(3)
O4	−0.09480(14)	0.61072(12)	0.69125(8)	0.0200(2)
O5	0.46931(14)	0.80407(11)	0.40167(8)	0.0168(2)
O6	0.43918(16)	0.94820(12)	0.09364(8)	0.0237(3)
O7	0.22676(17)	0.51327(12)	0.00107(9)	0.0267(3)
N1	0.35348(16)	0.41049(13)	0.48088(9)	0.0135(2)
H1N	0.409(2)	0.3405(16)	0.5166(12)	0.016
N2	−0.03627(17)	0.73135(13)	0.55877(10)	0.0173(3)
N3	0.12535(16)	0.50260(13)	0.59469(9)	0.0145(3)
N5	0.45006(17)	0.87776(13)	0.24799(9)	0.0166(3)
N6	0.31230(17)	0.78769(13)	0.14414(9)	0.0156(3)
N7	0.20956(18)	0.60590(14)	0.19027(10)	0.0178(3)
H7A	0.205(3)	0.589(2)	0.1314(11)	0.027
H7B	0.196(3)	0.5419(18)	0.2351(12)	0.027
N4A	0.9509(9)	0.0895(6)	0.1898(5)	0.0236(11)
O1A	1.0271(10)	0.1337(11)	0.1241(5)	0.0323(13)
O2A	0.9753(5)	−0.0328(3)	0.2103(2)	0.0380(11)
N4B	0.9717(15)	0.1133(10)	0.1922(8)	0.027(2)
O1B	0.9974(19)	0.145(2)	0.1098(9)	0.030(2)
O2B	1.0599(11)	0.0103(8)	0.2336(4)	0.059(3)
C1	0.41690(19)	0.46026(15)	0.39669(10)	0.0132(3)
C2	0.31966(19)	0.59483(15)	0.37895(10)	0.0133(3)
C3	0.19417(19)	0.62656(15)	0.45415(10)	0.0134(3)
C4	0.05900(19)	0.74714(16)	0.47493(11)	0.0155(3)
C5	−0.0077(2)	0.61521(16)	0.61924(11)	0.0157(3)
C6	0.22165(18)	0.51122(15)	0.51426(10)	0.0129(3)
C7	0.55915(19)	0.36914(15)	0.34464(10)	0.0131(3)
C8	0.6433(2)	0.23773(16)	0.38269(11)	0.0170(3)
H8	0.609093	0.209156	0.443531	0.020
C9	0.7751(2)	0.14934(17)	0.33305(12)	0.0211(3)
H9	0.831050	0.060615	0.359132	0.025
C10	0.8237(2)	0.19275(17)	0.24477(11)	0.0189(3)
C11	0.7477(2)	0.32203(17)	0.20517(11)	0.0178(3)
H11	0.785642	0.350418	0.145163	0.021
C12	0.6151(2)	0.40907(16)	0.25497(11)	0.0169(3)
H12	0.560591	0.497658	0.228145	0.020
C13	0.34229(19)	0.69390(15)	0.30285(10)	0.0138(3)
C14	0.41981(19)	0.79207(15)	0.32345(11)	0.0140(3)
C15	0.4023(2)	0.87633(16)	0.15791(11)	0.0168(3)
C16	0.28695(19)	0.69469(15)	0.21367(11)	0.0145(3)
C17	0.5435(2)	0.97304(18)	0.26293(12)	0.0223(3)
H17A	0.649230	0.946482	0.225610	0.034
H17B	0.564788	0.969571	0.329807	0.034

H17C	0.478805	1.066743	0.243347	0.034
C18	0.2357(2)	0.80249(18)	0.05329(11)	0.0215(3)
H18A	0.248757	0.884471	0.017944	0.032
H18B	0.117365	0.811910	0.064272	0.032
H18C	0.289903	0.720968	0.017082	0.032
C19	−0.1760(2)	0.85183(18)	0.58499(14)	0.0269(4)
H19A	−0.139586	0.934320	0.578665	0.040
H19B	−0.214829	0.838621	0.650365	0.040
H19C	−0.266577	0.862865	0.543409	0.040
C20	0.1620(2)	0.37867(17)	0.65717(11)	0.0190(3)
H20A	0.215442	0.297755	0.620243	0.029
H20B	0.058530	0.369970	0.688372	0.029
H20C	0.236527	0.385299	0.704720	0.029
C21	0.1707(3)	0.2911(2)	−0.05411(14)	0.0315(4)
H21A	0.136615	0.329007	−0.117008	0.047
H21B	0.073556	0.312440	−0.009540	0.047
H21C	0.220068	0.191118	−0.055856	0.047
C22	0.3526(3)	0.2765(2)	0.09337(14)	0.0340(5)
H22A	0.395861	0.177257	0.084781	0.051
H22B	0.247584	0.296901	0.131422	0.051
H22C	0.433028	0.306179	0.125495	0.051
S1	0.31886(6)	0.36501(4)	−0.01774(3)	0.02227(10)

U_{eq} is defined as 1/3 of the trace of the orthogonalized U_{ij} tensor.

Table 3. Anisotropic displacement parameters [Å^2] for Jessen_ki232_pr1recr_a. The anisotropic displacement factor exponent takes the form: $-2\pi^2[\, h^2(a^*)^2U_{11} + k^2(b^*)^2U_{22} + \ldots + 2hka^*b^*U_{12}\,]$

Atom	U_{11}	U_{22}	U_{33}	U_{23}	U_{13}	U_{12}
O3	0.0228(6)	0.0142(5)	0.0230(6)	0.0023(4)	0.0045(5)	−0.0012(5)
O4	0.0203(6)	0.0232(6)	0.0164(5)	−0.0031(5)	0.0074(4)	−0.0082(5)
O5	0.0202(6)	0.0149(5)	0.0153(5)	0.0005(4)	−0.0012(4)	−0.0055(4)
O6	0.0353(7)	0.0203(6)	0.0166(6)	0.0022(5)	0.0049(5)	−0.0121(5)
O7	0.0398(8)	0.0161(6)	0.0203(6)	−0.0011(5)	0.0009(5)	−0.0034(5)
N1	0.0139(6)	0.0124(6)	0.0130(6)	0.0012(5)	0.0015(5)	−0.0034(5)
N2	0.0165(6)	0.0133(6)	0.0196(6)	−0.0023(5)	0.0061(5)	−0.0025(5)
N3	0.0151(6)	0.0150(6)	0.0127(6)	0.0016(5)	0.0021(5)	−0.0048(5)
N5	0.0216(7)	0.0144(6)	0.0153(6)	0.0018(5)	0.0014(5)	−0.0088(5)
N6	0.0199(7)	0.0139(6)	0.0117(6)	0.0008(5)	0.0016(5)	−0.0043(5)
N7	0.0225(7)	0.0186(7)	0.0134(6)	−0.0003(5)	0.0014(5)	−0.0085(6)
N4A	0.0205(17)	0.0226(18)	0.0217(15)	−0.0046(13)	0.0064(12)	0.0014(14)
O1A	0.025(3)	0.033(2)	0.031(3)	−0.0010(19)	0.014(2)	−0.002(2)
O2A	0.042(2)	0.0204(11)	0.0359(14)	−0.0005(10)	0.0166(13)	0.0084(11)
N4B	0.028(4)	0.018(3)	0.022(3)	0.004(2)	0.001(2)	0.009(3)
O1B	0.023(4)	0.039(3)	0.015(2)	0.001(2)	0.001(2)	0.009(3)

O2B	0.055(4)	0.046(4)	0.035(3)	0.022(2)	0.019(3)	0.037(3)
C1	0.0133(7)	0.0138(7)	0.0124(7)	0.0006(5)	0.0013(5)	−0.0048(6)
C2	0.0155(7)	0.0135(7)	0.0114(6)	−0.0006(5)	0.0016(5)	−0.0056(6)
C3	0.0147(7)	0.0118(6)	0.0133(7)	−0.0004(5)	0.0019(5)	−0.0040(5)
C4	0.0154(7)	0.0149(7)	0.0157(7)	−0.0018(6)	0.0027(6)	−0.0047(6)
C5	0.0159(7)	0.0171(7)	0.0155(7)	−0.0023(6)	0.0021(6)	−0.0072(6)
C6	0.0129(7)	0.0142(7)	0.0124(6)	−0.0012(5)	0.0015(5)	−0.0057(6)
C7	0.0132(7)	0.0132(7)	0.0136(7)	−0.0019(5)	0.0001(5)	−0.0048(5)
C8	0.0184(8)	0.0155(7)	0.0147(7)	0.0003(6)	0.0016(6)	−0.0025(6)
C9	0.0228(8)	0.0173(8)	0.0178(8)	0.0006(6)	0.0002(6)	0.0014(6)
C10	0.0153(7)	0.0208(8)	0.0162(7)	−0.0042(6)	0.0023(6)	0.0012(6)
C11	0.0171(8)	0.0201(8)	0.0154(7)	−0.0026(6)	0.0040(6)	−0.0051(6)
C12	0.0175(8)	0.0164(7)	0.0155(7)	0.0001(6)	0.0021(6)	−0.0042(6)
C13	0.0151(7)	0.0112(6)	0.0133(7)	0.0003(5)	0.0028(5)	−0.0022(5)
C14	0.0138(7)	0.0109(6)	0.0142(7)	0.0009(5)	0.0038(5)	−0.0007(5)
C15	0.0197(8)	0.0127(7)	0.0159(7)	−0.0012(5)	0.0042(6)	−0.0027(6)
C16	0.0137(7)	0.0119(6)	0.0148(7)	−0.0010(5)	0.0040(5)	−0.0006(5)
C17	0.0288(9)	0.0208(8)	0.0212(8)	0.0020(6)	0.0004(7)	−0.0142(7)
C18	0.0304(9)	0.0205(8)	0.0137(7)	0.0028(6)	−0.0021(6)	−0.0085(7)
C19	0.0244(9)	0.0177(8)	0.0329(10)	−0.0034(7)	0.0143(7)	−0.0013(7)
C20	0.0191(8)	0.0204(8)	0.0162(7)	0.0052(6)	0.0019(6)	−0.0057(6)
C21	0.0314(10)	0.0334(10)	0.0314(10)	−0.0108(8)	0.0007(8)	−0.0109(8)
C22	0.0486(13)	0.0205(9)	0.0319(10)	0.0028(8)	−0.0133(9)	−0.0074(9)
S1	0.0238(2)	0.0183(2)	0.0234(2)	−0.00390(15)	0.00503(16)	−0.00526(16)

Table 4. Bond lengths and angles for Jessen_ki232_pr1recr_a.

Atom–Atom	Length [Å]
O3–C4	1.2216(19)
O4–C5	1.2264(19)
O5–C14	1.2470(19)
O6–C15	1.2185(19)
O7–S1	1.5090(14)
N1–C6	1.348(2)
N1–C1	1.4066(19)
N1–H1N	0.879(13)
N2–C5	1.388(2)
N2–C4	1.421(2)
N2–C19	1.470(2)
N3–C6	1.3716(19)
N3–C5	1.385(2)
N3–C20	1.464(2)
N5–C15	1.377(2)
N5–C14	1.4002(19)
N5–C17	1.466(2)
N6–C16	1.3782(19)
N6–C15	1.384(2)
N6–C18	1.470(2)
N7–C16	1.344(2)
N7–H7A	0.877(14)
N7–H7B	0.911(14)
N4A–O2A	1.221(6)
N4A–O1A	1.237(7)
N4A–C10	1.477(6)
N4B–O1B	1.219(11)
N4B–O2B	1.231(10)
N4B–C10	1.453(10)

C1–C2	1.387(2)
C1–C7	1.464(2)
C2–C3	1.433(2)
C2–C13	1.476(2)
C3–C6	1.380(2)
C3–C4	1.433(2)
C7–C8	1.407(2)
C7–C12	1.409(2)
C8–C9	1.384(2)
C8–H8	0.9500
C9–C10	1.382(2)
C9–H9	0.9500
C10–C11	1.383(2)
C11–C12	1.383(2)
C11–H11	0.9500
C12–H12	0.9500
C13–C16	1.386(2)
C13–C14	1.415(2)
C17–H17A	0.9800
C17–H17B	0.9800
C17–H17C	0.9800
C18–H18A	0.9800
C18–H18B	0.9800
C18–H18C	0.9800
C19–H19A	0.9800
C19–H19B	0.9800
C19–H19C	0.9800
C20–H20A	0.9800
C20–H20B	0.9800
C20–H20C	0.9800
C21–S1	1.776(2)
C21–H21A	0.9800
C21–H21B	0.9800
C21–H21C	0.9800
C22–S1	1.768(2)
C22–H22A	0.9800
C22–H22B	0.9800
C22–H22C	0.9800
**Atom–Atom–Atom**	**Angle [°]**
C6–N1–C1	108.49(12)
C6–N1–H1N	122.7(12)
C1–N1–H1N	125.2(12)
C5–N2–C4	126.50(13)
C5–N2–C19	117.15(13)
C4–N2–C19	116.33(13)
C6–N3–C5	119.41(13)
C6–N3–C20	121.79(13)
C5–N3–C20	118.78(13)
C15–N5–C14	124.33(14)
C15–N5–C17	116.48(13)
C14–N5–C17	119.15(13)
C16–N6–C15	122.09(14)
C16–N6–C18	120.95(14)
C15–N6–C18	116.87(13)
C16–N7–H7A	121.4(13)
C16–N7–H7B	117.7(13)
H7A–N7–H7B	117.4(18)
O2A–N4A–O1A	123.6(7)
O2A–N4A–C10	119.5(4)
O1A–N4A–C10	116.8(6)
O1B–N4B–O2B	123.3(11)
O1B–N4B–C10	120.2(11)
O2B–N4B–C10	116.3(8)
C2–C1–N1	107.96(13)
C2–C1–C7	132.56(14)
N1–C1–C7	119.46(13)
C1–C2–C3	106.66(13)
C1–C2–C13	128.64(14)
C3–C2–C13	124.56(14)
C6–C3–C4	119.85(14)
C6–C3–C2	107.08(13)
C4–C3–C2	133.07(14)
O3–C4–N2	119.70(14)
O3–C4–C3	126.88(14)
N2–C4–C3	113.42(13)
O4–C5–N3	120.74(14)
O4–C5–N2	122.40(15)
N3–C5–N2	116.86(13)
N1–C6–N3	126.24(14)
N1–C6–C3	109.80(13)
N3–C6–C3	123.95(14)
C8–C7–C12	117.90(14)

C8–C7–C1	120.68(14)
C12–C7–C1	121.42(14)
C9–C8–C7	121.17(15)
C9–C8–H8	119.4
C7–C8–H8	119.4
C10–C9–C8	118.67(15)
C10–C9–H9	120.7
C8–C9–H9	120.7
C9–C10–C11	122.42(15)
C9–C10–N4B	122.3(4)
C11–C10–N4B	114.8(4)
C9–C10–N4A	116.7(3)
C11–C10–N4A	120.6(3)
C10–C11–C12	118.43(15)
C10–C11–H11	120.8
C12–C11–H11	120.8
C11–C12–C7	121.39(15)
C11–C12–H12	119.3
C7–C12–H12	119.3
C16–C13–C14	119.85(14)
C16–C13–C2	121.97(14)
C14–C13–C2	118.17(14)
O5–C14–N5	117.89(14)
O5–C14–C13	125.47(14)
N5–C14–C13	116.56(14)
O6–C15–N5	121.67(15)
O6–C15–N6	121.88(15)
N5–C15–N6	116.44(13)
N7–C16–N6	117.07(14)
N7–C16–C13	122.68(14)
N6–C16–C13	120.24(14)
N5–C17–H17A	109.5
N5–C17–H17B	109.5
H17A–C17–H17B	109.5
N5–C17–H17C	109.5
H17A–C17–H17C	109.5

H17B–C17–H17C	109.5
N6–C18–H18A	109.5
N6–C18–H18B	109.5
H18A–C18–H18B	109.5
N6–C18–H18C	109.5
H18A–C18–H18C	109.5
H18B–C18–H18C	109.5
N2–C19–H19A	109.5
N2–C19–H19B	109.5
H19A–C19–H19B	109.5
N2–C19–H19C	109.5
H19A–C19–H19C	109.5
H19B–C19–H19C	109.5
N3–C20–H20A	109.5
N3–C20–H20B	109.5
H20A–C20–H20B	109.5
N3–C20–H20C	109.5
H20A–C20–H20C	109.5
H20B–C20–H20C	109.5
S1–C21–H21A	109.5
S1–C21–H21B	109.5
H21A–C21–H21B	109.5
S1–C21–H21C	109.5
H21A–C21–H21C	109.5
H21B–C21–H21C	109.5
S1–C22–H22A	109.5
S1–C22–H22B	109.5
H22A–C22–H22B	109.5
S1–C22–H22C	109.5
H22A–C22–H22C	109.5
H22B–C22–H22C	109.5
O7–S1–C22	106.57(9)
O7–S1–C21	106.68(10)
C22–S1–C21	97.73(10)

Table 5. Torsion angles for Jessen_ki232_pr1recr_a.

Atom–Atom–Atom–Atom	Torsion Angle [°]
C6–N1–C1–C2	−0.21(17)
C6–N1–C1–C7	−178.54(13)
N1–C1–C2–C3	−0.25(17)
C7–C1–C2–C3	177.77(16)
N1–C1–C2–C13	175.57(15)
C7–C1–C2–C13	−6.4(3)
C1–C2–C3–C6	0.61(17)
C13–C2–C3–C6	−175.43(14)
C1–C2–C3–C4	−179.76(16)
C13–C2–C3–C4	4.2(3)
C5–N2–C4–O3	−179.30(15)
C19–N2–C4–O3	−1.2(2)
C5–N2–C4–C3	1.2(2)
C19–N2–C4–C3	179.29(15)
C6–C3–C4–O3	179.70(16)
C2–C3–C4–O3	0.1(3)
C6–C3–C4–N2	−0.8(2)
C2–C3–C4–N2	179.61(16)
C6–N3–C5–O4	−179.30(14)
C20–N3–C5–O4	−1.1(2)
C6–N3–C5–N2	0.7(2)
C20–N3–C5–N2	178.96(14)
C4–N2–C5–O4	178.88(15)
C19–N2–C5–O4	0.8(2)
C4–N2–C5–N3	−1.1(2)
C19–N2–C5–N3	−179.26(15)
C1–N1–C6–N3	179.58(14)
C1–N1–C6–C3	0.61(17)
C5–N3–C6–N1	−179.33(14)
C20–N3–C6–N1	2.5(2)
C5–N3–C6–C3	−0.5(2)
C20–N3–C6–C3	−178.68(15)
C4–C3–C6–N1	179.56(14)
C2–C3–C6–N1	−0.76(17)
C4–C3–C6–N3	0.6(2)
C2–C3–C6–N3	−179.76(14)
C2–C1–C7–C8	175.54(16)
N1–C1–C7–C8	−6.6(2)
C2–C1–C7–C12	−5.1(3)
N1–C1–C7–C12	172.74(14)
C12–C7–C8–C9	−1.1(2)
C1–C7–C8–C9	178.26(15)
C7–C8–C9–C10	0.2(3)
C8–C9–C10–C11	1.3(3)
C8–C9–C10–N4B	173.0(7)
C8–C9–C10–N4A	−172.8(4)
O1B–N4B–C10–C9	169.4(13)
O2B–N4B–C10–C9	−5.8(14)
O1B–N4B–C10–C11	−18.4(18)
O2B–N4B–C10–C11	166.4(9)
O2A–N4A–C10–C9	19.4(9)
O1A–N4A–C10–C9	−160.9(7)
O2A–N4A–C10–C11	−154.8(6)
O1A–N4A–C10–C11	24.9(10)
C9–C10–C11–C12	−1.9(3)
N4B–C10–C11–C12	−174.2(7)
N4A–C10–C11–C12	172.0(4)
C10–C11–C12–C7	1.0(2)
C8–C7–C12–C11	0.5(2)
C1–C7–C12–C11	−178.89(15)
C1–C2–C13–C16	83.3(2)
C3–C2–C13–C16	−101.56(19)
C1–C2–C13–C14	−98.08(19)
C3–C2–C13–C14	77.1(2)
C15–N5–C14–O5	−179.81(14)
C17–N5–C14–O5	2.4(2)
C15–N5–C14–C13	3.3(2)
C17–N5–C14–C13	−174.51(14)
C16–C13–C14–O5	177.48(15)
C2–C13–C14–O5	−1.2(2)
C16–C13–C14–N5	−5.9(2)
C2–C13–C14–N5	175.50(13)
C14–N5–C15–O6	−176.18(15)
C17–N5–C15–O6	1.6(2)
C14–N5–C15–N6	3.0(2)
C17–N5–C15–N6	−179.18(14)
C16–N6–C15–O6	172.22(15)
C18–N6–C15–O6	−11.3(2)
C16–N6–C15–N5	−7.0(2)
C18–N6–C15–N5	169.51(14)

C15–N6–C16–N7	−174.64(14)
C18–N6–C16–N7	9.0(2)
C15–N6–C16–C13	4.5(2)
C18–N6–C16–C13	−171.84(14)

C14–C13–C16–N7	−178.69(14)
C2–C13–C16–N7	−0.1(2)
C14–C13–C16–N6	2.2(2)
C2–C13–C16–N6	−179.17(14)

Crystal Data for **15c** derivative

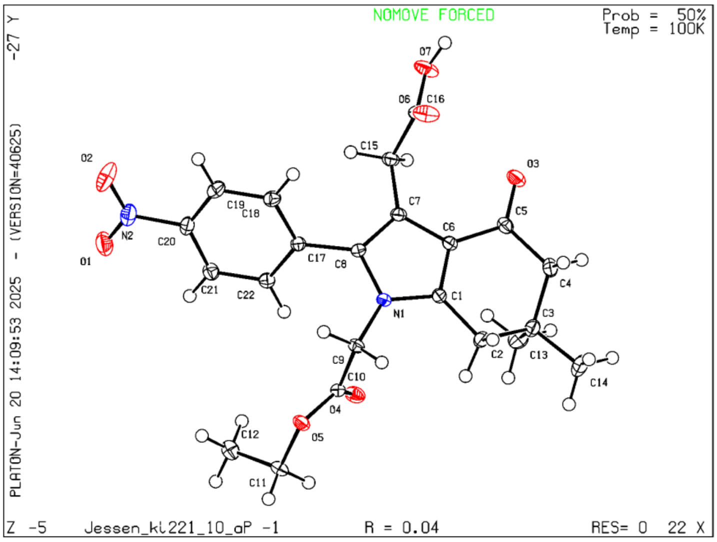

checkCIF/PLATON report

Structure factors have been supplied for datablock(s) Jessen_ki221_10_a

THIS REPORT IS FOR GUIDANCE ONLY. IF USED AS PART OF A REVIEW PROCEDURE FOR PUBLICATION, IT SHOULD NOT REPLACE THE EXPERTISE OF AN EXPERIENCED CRYSTALLOGRAPHIC REFEREE.

No syntax errors found. CIF dictionary Interpreting this report

Datablock: Jessen_ki221_10_a

Bond precision: C-C = 0.0016 A Wavelength=0.71073

Cell:	a=9.305(3)	b=9.730(3)	c=13.155(4)
	alpha=82.906(13)	beta=70.40(2)	gamma=68.37(2)
Temperature:	100 K		

	Calculated	Reported
Volume	1043.0(6)	1043.1(6)
Space group	P -1	P -1
Hall group	-P 1	-P 1
Moiety formula	C22 H24 N2 O7	C22 H24 N2 O7
Sum formula	C22 H24 N2 O7	C22 H24 N2 O7
Mr	428.43	428.43
Dx,g cm-3	1.364	1.364
Z	2	2
Mu (mm-1)	0.103	0.103
F000	452.0	452.0
F000'	452.25	
h,k,lmax	13,13,18	13,13,18
Nref	6406	6391
Tmin,Tmax	0.974,0.979	0.721,0.746
Tmin'	0.964	

Correction method= # Reported T Limits: Tmin=0.721 Tmax=0.746
AbsCorr = MULTI-SCAN

Data completeness= 0.998 Theta(max)= 30.598

R(reflections)= 0.0409(5561) wR2(reflections)= 0.1132(6391)

S = 1.024 Npar= 285

Table 1. Crystal data and structure refinement for Jessen_ki221_10_a.

CCDC number	2465978
Empirical formula	$C_{22}H_{24}N_2O_7$
Formula weight	428.43
Temperature [K]	100(2)
Crystal system	triclinic
Space group (number)	$P\bar{1}$ (2)
a [Å]	9.305(3)
b [Å]	9.730(3)
c [Å]	13.155(4)
α [°]	82.906(13)
β [°]	70.40(2)
γ [°]	68.37(2)
Volume [$Å^3$]	1043.1(6)
Z	2
ρ_{calc} [gcm^{-3}]	1.364
μ [mm^{-1}]	0.103
F(000)	452
Crystal size [mm^3]	0.210×0.216×0.356
Crystal colour	colourless
Crystal shape	block
Radiation	MoK_α<br>(λ=0.71073 Å)
2θ range [°]	3.29 to 61.20<br>(0.70 Å)
Index ranges	−13 ≤ h ≤ 13<br>−13 ≤ k ≤ 13<br>−18 ≤ l ≤ 18
Reflections collected	45563
Independent reflec-tions	6391<br>R_{int} = 0.0315<br>R_{sigma} = 0.0190
Completeness to θ = 25.242°	99.9 %
Data / Restraints / Parameters	6391 / 0 / 285
Absorption correction T_{min}/T_{max} (method)	0.7206 / 0.7461<br>(multi-scan)
Goodness-of-fit on F^2	1.024
Final *R* indexes [$I\geq2\sigma(I)$]	R_1 = 0.0409<br>wR_2 = 0.1077
Final *R* indexes [all data]	R_1 = 0.0472<br>wR_2 = 0.1132
Largest peak/hole [$eÅ^{-3}$]	0.48/−0.26

Table 2. Atomic coordinates and U_{eq} [$Å^2$] for Jessen_ki221_10_a.

Atom	*x*	*y*	*z*	U_{eq}
O1	−0.13916(12)	0.91728(10)	0.50295(8)	0.0297(2)
O2	−0.30457(11)	1.02400(12)	0.65474(9)	0.0379(2)
O3	0.66367(10)	0.78375(9)	0.90262(7)	0.02170(16)
O4	0.55888(9)	0.36782(9)	0.59836(6)	0.01985(16)
O5	0.35974(9)	0.26923(8)	0.65025(6)	0.01696(15)
O6	0.26959(11)	0.93016(9)	1.01135(7)	0.02567(18)
O7	0.34588(12)	1.11285(9)	0.91800(7)	0.02583(19)
H7	0.326(2)	1.1461(12)	0.9854(14)	0.039
N1	0.42082(10)	0.52424(9)	0.79401(7)	0.01271(15)
N2	−0.17226(12)	0.94074(11)	0.59927(8)	0.02269(19)
C1	0.55163(11)	0.50551(10)	0.82585(7)	0.01329(17)
C2	0.68007(12)	0.36266(11)	0.83698(9)	0.01656(18)
H2A	0.700769	0.292402	0.780944	0.020
H2B	0.643176	0.318726	0.908607	0.020
C3	0.83876(12)	0.39089(11)	0.82443(9)	0.01737(19)
C4	0.79714(13)	0.51935(12)	0.89971(9)	0.0193(2)
H4A	0.760089	0.487288	0.975415	0.023
H4B	0.897702	0.539922	0.889350	0.023
C5	0.66759(12)	0.66140(11)	0.88231(8)	0.01582(18)
C6	0.54732(12)	0.64459(10)	0.84413(8)	0.01410(17)
C7	0.40880(12)	0.75318(10)	0.82074(8)	0.01401(17)
C8	0.33248(11)	0.67631(10)	0.79011(7)	0.01288(17)
C9	0.36956(12)	0.40614(10)	0.77928(8)	0.01414(17)
H9A	0.249354	0.443163	0.798773	0.017
H9B	0.401428	0.324053	0.828992	0.017
C10	0.44275(12)	0.34720(10)	0.66448(8)	0.01384(17)
C11	0.41467(14)	0.20216(12)	0.54416(9)	0.0194(2)
H11A	0.385275	0.112650	0.551405	0.023
H11B	0.534655	0.171938	0.514020	0.023
C12	0.33724(14)	0.31022(13)	0.46841(9)	0.0215(2)
H12A	0.372829	0.262660	0.398372	0.032
H12B	0.370208	0.396921	0.458876	0.032
H12C	0.218442	0.341175	0.498844	0.032
C13	0.91607(13)	0.42933(13)	0.70695(9)	0.0229(2)
H13A	0.945457	0.345771	0.660224	0.034
H13B	1.014064	0.450039	0.700780	0.034
H13C	0.838057	0.516726	0.684820	0.034
C14	0.96043(14)	0.25218(13)	0.85699(11)	0.0266(2)
H14A	0.988026	0.170139	0.809358	0.040
H14B	0.911785	0.226319	0.931785	0.040
H14C	1.059392	0.271288	0.850675	0.040
C15	0.37020(13)	0.91676(11)	0.81705(8)	0.01622(18)
H15A	0.279371	0.964088	0.786682	0.019

H15B	0.466290	0.938591	0.767995	0.019
C16	0.32362(12)	0.98379(11)	0.92653(8)	0.01603(18)
C17	0.19594(11)	0.73415(10)	0.74554(8)	0.01334(17)
C18	0.04751(12)	0.83827(11)	0.80298(8)	0.01716(19)
H18	0.030676	0.863776	0.874548	0.021
C19	−0.07535(12)	0.90461(11)	0.75620(9)	0.01865(19)
H19	−0.176405	0.974891	0.794975	0.022
C20	−0.04658(12)	0.86557(11)	0.65171(9)	0.01712(19)
C21	0.09744(13)	0.76118(11)	0.59255(8)	0.01766(19)
H21	0.112774	0.735802	0.521257	0.021
C22	0.21846(12)	0.69494(11)	0.64073(8)	0.01579(18)
H22	0.317665	0.622227	0.602259	0.019

U_{eq} is defined as 1/3 of the trace of the orthogonalized U_{ij} tensor.

Table 3. Anisotropic displacement parameters [Å^2] for Jessen_ki221_10_a. The anisotropic displacement factor exponent takes the form: $-2\pi^2[h^2(a^*)^2U_{11} + k^2(b^*)^2U_{22} + \ldots + 2hka^*b^*U_{12}]$

**Atom**	U_{11}	U_{22}	U_{33}	U_{23}	U_{13}	U_{12}
O1	0.0345(5)	0.0316(5)	0.0327(5)	0.0057(4)	−0.0228(4)	−0.0133(4)
O2	0.0190(4)	0.0428(6)	0.0410(5)	0.0085(4)	−0.0121(4)	0.0011(4)
O3	0.0228(4)	0.0215(4)	0.0255(4)	−0.0058(3)	−0.0087(3)	−0.0102(3)
O4	0.0191(4)	0.0214(4)	0.0184(3)	−0.0055(3)	−0.0006(3)	−0.0094(3)
O5	0.0188(3)	0.0169(3)	0.0183(3)	−0.0035(3)	−0.0066(3)	−0.0080(3)
O6	0.0345(5)	0.0201(4)	0.0196(4)	−0.0038(3)	0.0005(3)	−0.0132(3)
O7	0.0457(5)	0.0176(4)	0.0217(4)	0.0003(3)	−0.0125(4)	−0.0178(4)
N1	0.0130(3)	0.0114(3)	0.0144(3)	−0.0022(3)	−0.0045(3)	−0.0041(3)
N2	0.0194(4)	0.0221(4)	0.0307(5)	0.0080(4)	−0.0134(4)	−0.0094(4)
C1	0.0123(4)	0.0143(4)	0.0133(4)	−0.0015(3)	−0.0040(3)	−0.0042(3)
C2	0.0141(4)	0.0137(4)	0.0218(5)	0.0004(3)	−0.0071(4)	−0.0036(3)
C3	0.0137(4)	0.0165(4)	0.0215(5)	0.0012(3)	−0.0067(4)	−0.0042(3)
C4	0.0180(4)	0.0222(5)	0.0211(5)	0.0004(4)	−0.0101(4)	−0.0074(4)
C5	0.0153(4)	0.0192(4)	0.0139(4)	−0.0026(3)	−0.0037(3)	−0.0072(3)
C6	0.0139(4)	0.0144(4)	0.0146(4)	−0.0029(3)	−0.0046(3)	−0.0046(3)
C7	0.0145(4)	0.0123(4)	0.0144(4)	−0.0029(3)	−0.0036(3)	−0.0039(3)
C8	0.0120(4)	0.0119(4)	0.0133(4)	−0.0022(3)	−0.0033(3)	−0.0024(3)
C9	0.0152(4)	0.0138(4)	0.0150(4)	−0.0020(3)	−0.0036(3)	−0.0071(3)
C10	0.0143(4)	0.0108(4)	0.0169(4)	−0.0020(3)	−0.0063(3)	−0.0030(3)
C11	0.0231(5)	0.0175(4)	0.0197(5)	−0.0061(4)	−0.0088(4)	−0.0057(4)
C12	0.0231(5)	0.0254(5)	0.0189(5)	−0.0007(4)	−0.0079(4)	−0.0102(4)
C13	0.0175(5)	0.0247(5)	0.0216(5)	−0.0014(4)	−0.0015(4)	−0.0058(4)
C14	0.0181(5)	0.0197(5)	0.0437(7)	0.0069(5)	−0.0160(5)	−0.0052(4)
C15	0.0201(5)	0.0126(4)	0.0168(4)	−0.0013(3)	−0.0068(4)	−0.0054(3)
C16	0.0165(4)	0.0117(4)	0.0201(4)	−0.0023(3)	−0.0065(4)	−0.0038(3)
C17	0.0128(4)	0.0125(4)	0.0145(4)	−0.0008(3)	−0.0038(3)	−0.0043(3)
C18	0.0153(4)	0.0174(4)	0.0158(4)	−0.0030(3)	−0.0030(3)	−0.0031(3)

C19	0.0133(4)	0.0171(4)	0.0214(5)	−0.0007(4)	−0.0031(4)	−0.0025(3)
C20	0.0145(4)	0.0166(4)	0.0221(5)	0.0040(4)	−0.0090(4)	−0.0058(3)
C21	0.0189(5)	0.0188(4)	0.0167(4)	−0.0001(3)	−0.0072(4)	−0.0067(4)
C22	0.0148(4)	0.0161(4)	0.0154(4)	−0.0026(3)	−0.0043(3)	−0.0039(3)

Table 4. Bond lengths and angles for Jessen_ki221_10_a.

**Atom–Atom**	**Length [Å]**
O1–N2	1.2290(14)
O2–N2	1.2299(15)
O3–C5	1.2369(13)
O4–C10	1.2045(13)
O5–C10	1.3309(12)
O5–C11	1.4613(13)
O6–C16	1.2014(14)
O7–C16	1.3318(13)
O7–H7	0.917(19)
N1–C1	1.3601(13)
N1–C8	1.4074(13)
N1–C9	1.4514(13)
N2–C20	1.4712(14)
C1–C6	1.3873(14)
C1–C2	1.4914(14)
C2–C3	1.5515(15)
C2–H2A	0.9900
C2–H2B	0.9900
C3–C14	1.5307(15)
C3–C13	1.5348(16)
C3–C4	1.5443(16)
C4–C5	1.5148(15)
C4–H4A	0.9900
C4–H4B	0.9900
C5–C6	1.4367(14)
C6–C7	1.4333(14)
C7–C8	1.3726(14)
C7–C15	1.4953(14)
C8–C17	1.4717(14)
C9–C10	1.5197(14)
C9–H9A	0.9900
C9–H9B	0.9900
C11–C12	1.5106(16)
C11–H11A	0.9900
C11–H11B	0.9900
C12–H12A	0.9800
C12–H12B	0.9800
C12–H12C	0.9800
C13–H13A	0.9800
C13–H13B	0.9800
C13–H13C	0.9800
C14–H14A	0.9800
C14–H14B	0.9800
C14–H14C	0.9800
C15–C16	1.5141(14)
C15–H15A	0.9900
C15–H15B	0.9900
C17–C18	1.4009(14)
C17–C22	1.4027(14)
C18–C19	1.3901(15)
C18–H18	0.9500
C19–C20	1.3829(16)
C19–H19	0.9500
C20–C21	1.3876(15)
C21–C22	1.3885(14)
C21–H21	0.9500
C22–H22	0.9500
**Atom–Atom–Atom**	**Angle [°]**
C10–O5–C11	116.89(8)
C16–O7–H7	109.5
C1–N1–C8	109.26(8)
C1–N1–C9	125.17(8)
C8–N1–C9	125.18(8)
O1–N2–O2	123.85(10)
O1–N2–C20	118.36(10)
O2–N2–C20	117.78(10)
N1–C1–C6	107.65(8)

N1–C1–C2	126.90(9)
C6–C1–C2	125.43(9)
C1–C2–C3	109.25(8)
C1–C2–H2A	109.8
C3–C2–H2A	109.8
C1–C2–H2B	109.8
C3–C2–H2B	109.8
H2A–C2–H2B	108.3
C14–C3–C13	108.88(9)
C14–C3–C4	108.90(9)
C13–C3–C4	110.16(9)
C14–C3–C2	109.74(9)
C13–C3–C2	110.43(9)
C4–C3–C2	108.72(9)
C5–C4–C3	114.72(9)
C5–C4–H4A	108.6
C3–C4–H4A	108.6
C5–C4–H4B	108.6
C3–C4–H4B	108.6
H4A–C4–H4B	107.6
O3–C5–C6	122.33(10)
O3–C5–C4	121.81(9)
C6–C5–C4	115.83(9)
C1–C6–C7	108.45(9)
C1–C6–C5	120.96(9)
C7–C6–C5	130.58(9)
C8–C7–C6	106.27(9)
C8–C7–C15	127.45(9)
C6–C7–C15	125.84(9)
C7–C8–N1	108.36(8)
C7–C8–C17	128.52(9)
N1–C8–C17	122.62(8)
N1–C9–C10	113.08(8)
N1–C9–H9A	109.0
C10–C9–H9A	109.0
N1–C9–H9B	109.0
C10–C9–H9B	109.0
H9A–C9–H9B	107.8
O4–C10–O5	126.30(9)
O4–C10–C9	124.41(9)
O5–C10–C9	109.28(8)
O5–C11–C12	110.50(9)

O5–C11–H11A	109.5
C12–C11–H11A	109.5
O5–C11–H11B	109.5
C12–C11–H11B	109.5
H11A–C11–H11B	108.1
C11–C12–H12A	109.5
C11–C12–H12B	109.5
H12A–C12–H12B	109.5
C11–C12–H12C	109.5
H12A–C12–H12C	109.5
H12B–C12–H12C	109.5
C3–C13–H13A	109.5
C3–C13–H13B	109.5
H13A–C13–H13B	109.5
C3–C13–H13C	109.5
H13A–C13–H13C	109.5
H13B–C13–H13C	109.5
C3–C14–H14A	109.5
C3–C14–H14B	109.5
H14A–C14–H14B	109.5
C3–C14–H14C	109.5
H14A–C14–H14C	109.5
H14B–C14–H14C	109.5
C7–C15–C16	113.31(8)
C7–C15–H15A	108.9
C16–C15–H15A	108.9
C7–C15–H15B	108.9
C16–C15–H15B	108.9
H15A–C15–H15B	107.7
O6–C16–O7	123.39(10)
O6–C16–C15	124.83(9)
O7–C16–C15	111.76(9)
C18–C17–C22	119.30(9)
C18–C17–C8	120.61(9)
C22–C17–C8	119.85(9)
C19–C18–C17	120.59(10)
C19–C18–H18	119.7
C17–C18–H18	119.7
C20–C19–C18	118.24(9)
C20–C19–H19	120.9
C18–C19–H19	120.9
C19–C20–C21	123.09(9)

C19–C20–N2	118.75(10)
C21–C20–N2	118.14(10)
C20–C21–C22	117.98(10)
C20–C21–H21	121.0
C22–C21–H21	121.0
C21–C22–C17	120.77(9)
C21–C22–H22	119.6
C17–C22–H22	119.6

Table 5. Torsion angles for Jessen_ki221_10_a.

Atom–Atom–Atom–Atom	Torsion Angle [°]
C8–N1–C1–C6	0.84(11)
C9–N1–C1–C6	−172.24(8)
C8–N1–C1–C2	−177.76(9)
C9–N1–C1–C2	9.16(15)
N1–C1–C2–C3	154.87(9)
C6–C1–C2–C3	−23.50(14)
C1–C2–C3–C14	169.16(9)
C1–C2–C3–C13	−70.81(11)
C1–C2–C3–C4	50.16(11)
C14–C3–C4–C5	−175.54(9)
C13–C3–C4–C5	65.12(12)
C2–C3–C4–C5	−56.01(11)
C3–C4–C5–O3	−151.76(10)
C3–C4–C5–C6	30.11(13)
N1–C1–C6–C7	−0.91(11)
C2–C1–C6–C7	177.72(9)
N1–C1–C6–C5	177.97(9)
C2–C1–C6–C5	−3.40(15)
O3–C5–C6–C1	−177.60(10)
C4–C5–C6–C1	0.52(14)
O3–C5–C6–C7	1.00(17)
C4–C5–C6–C7	179.12(10)
C1–C6–C7–C8	0.63(11)
C5–C6–C7–C8	−178.10(10)
C1–C6–C7–C15	−172.19(9)
C5–C6–C7–C15	9.07(17)
C6–C7–C8–N1	−0.12(11)
C15–C7–C8–N1	172.55(9)
C6–C7–C8–C17	−172.11(9)
C15–C7–C8–C17	0.56(17)
C1–N1–C8–C7	−0.45(11)
C9–N1–C8–C7	172.63(9)
C1–N1–C8–C17	172.11(9)
C9–N1–C8–C17	−14.81(14)
C1–N1–C9–C10	−91.92(11)
C8–N1–C9–C10	96.08(11)
C11–O5–C10–O4	−0.03(14)
C11–O5–C10–C9	−179.28(8)
N1–C9–C10–O4	16.80(14)
N1–C9–C10–O5	−163.93(8)
C10–O5–C11–C12	−86.64(11)
C8–C7–C15–C16	120.17(11)
C6–C7–C15–C16	−68.52(13)
C7–C15–C16–O6	−23.70(15)
C7–C15–C16–O7	157.87(9)
C7–C8–C17–C18	−57.84(15)
N1–C8–C17–C18	131.19(10)
C7–C8–C17–C22	116.54(12)
N1–C8–C17–C22	−54.43(13)
C22–C17–C18–C19	−1.30(15)
C8–C17–C18–C19	173.11(9)
C17–C18–C19–C20	−0.33(15)
C18–C19–C20–C21	1.48(16)
C18–C19–C20–N2	−176.80(9)
O1–N2–C20–C19	172.28(10)
O2–N2–C20–C19	−7.51(15)
O1–N2–C20–C21	−6.08(15)
O2–N2–C20–C21	174.13(10)
C19–C20–C21–C22	−0.92(16)
N2–C20–C21–C22	177.36(9)
C20–C21–C22–C17	−0.79(15)
C18–C17–C22–C21	1.87(15)
C8–C17–C22–C21	−172.58(9)

^{1}H, ^{13}C NMR, HRMS and LCMS spectra of all compounds

2-(3-(2-Amino-1-cyano-2-oxoethyl)-2-(4-bromophenyl)-6,6-dimethyl-4-oxo-4,5,6,7-tetrahydro-1H-indol-1-yl)acetic acid (**4a**)

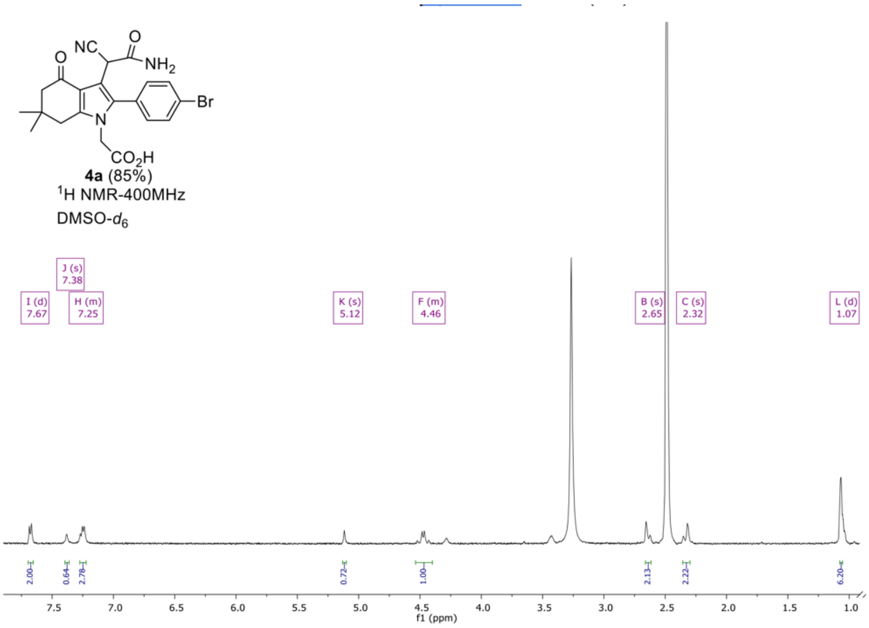

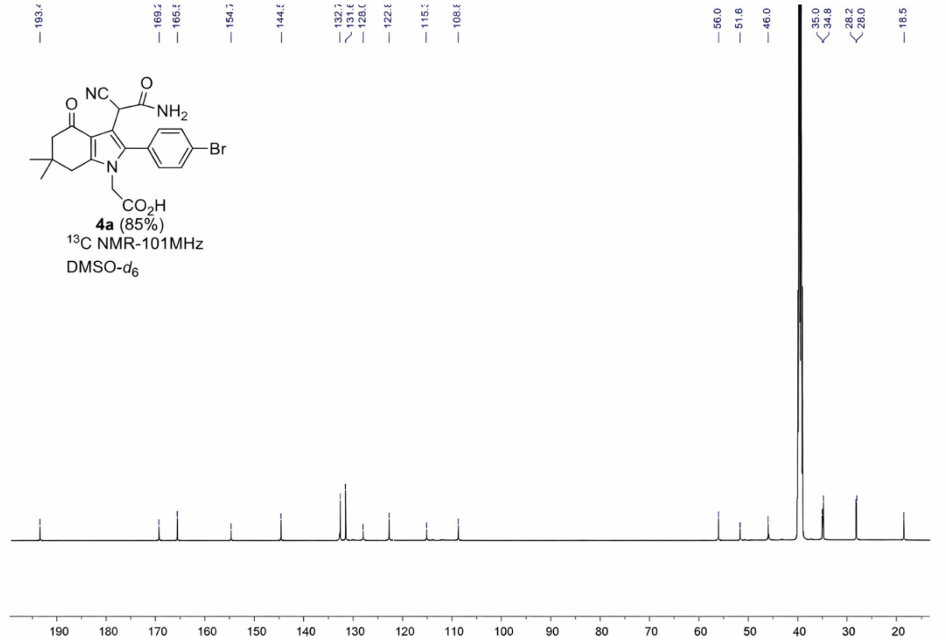

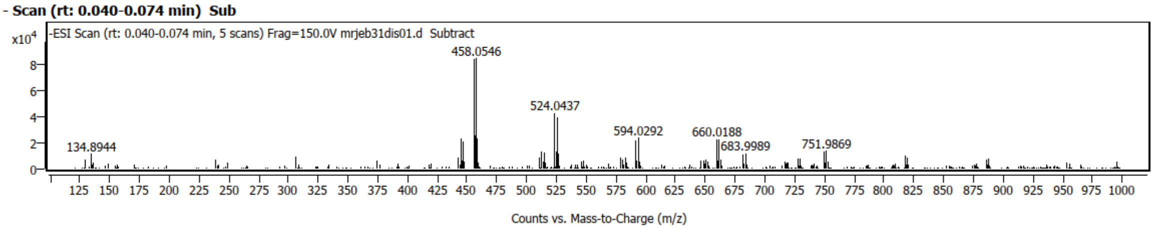

2-(3-(2-Amino-1-cyano-2-oxoethyl)-6,6-dimethyl-4-oxo-2-(p-tolyl)-4,5,6,7-tetrahydro-1H-indol-1-yl)acetic acid (**4b**)

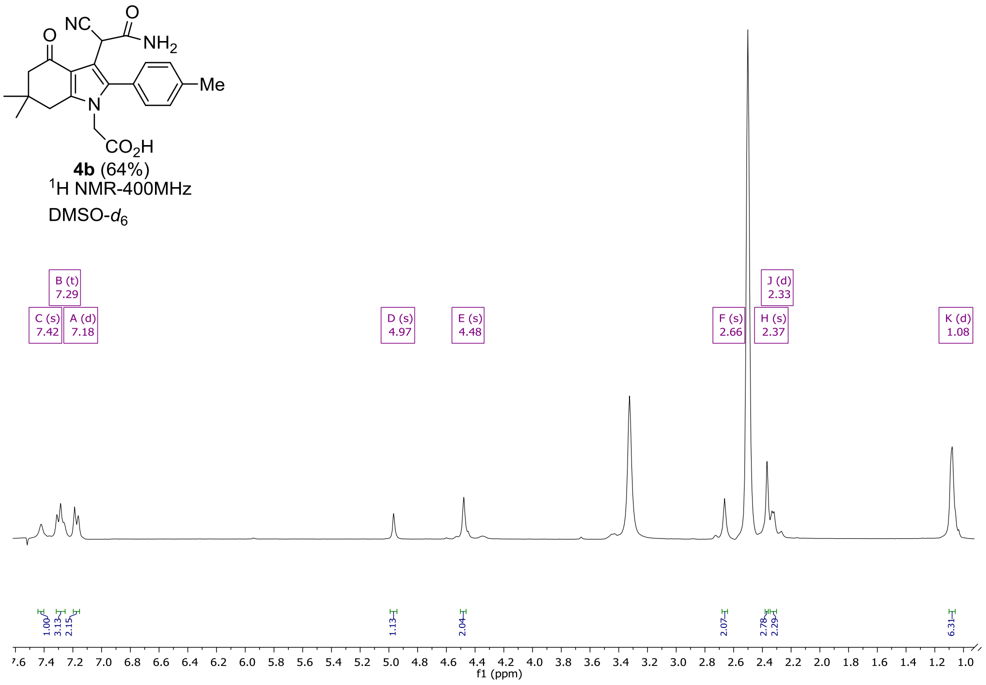

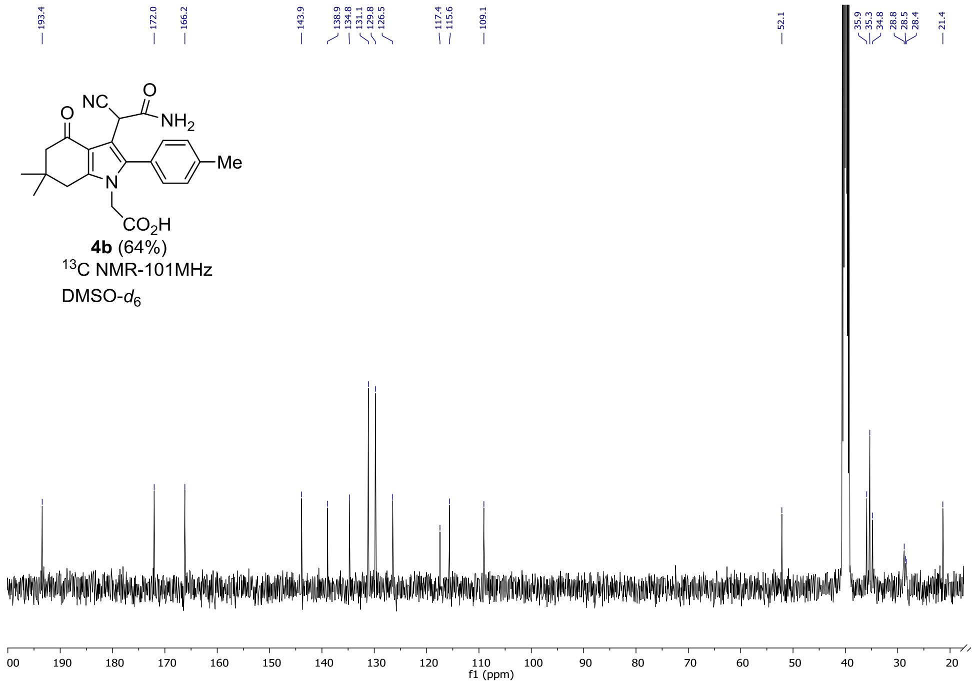

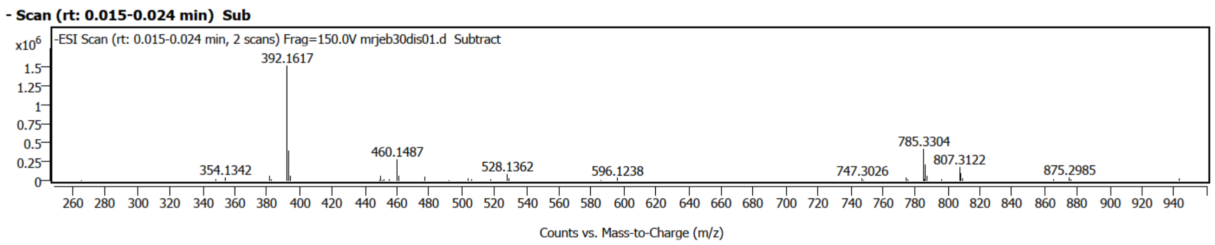

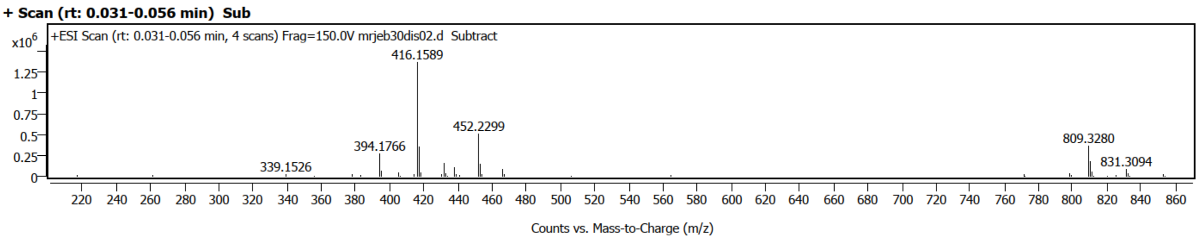

4-((3-(2-amino-1-cyano-2-oxoethyl)-2-(4-bromophenyl)-6,6-dimethyl-4-oxo-4,5,6,7-tetrahydro-1H-indol-1-yl)methyl)benzoic acid (**4c**)

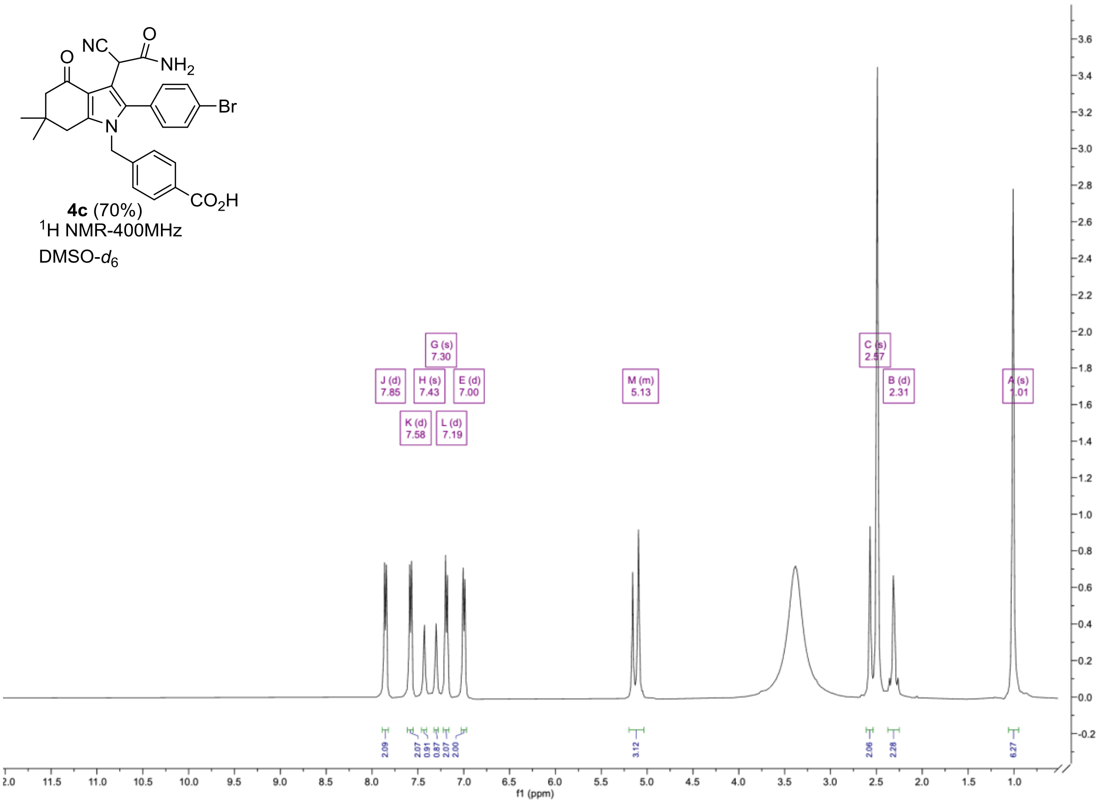

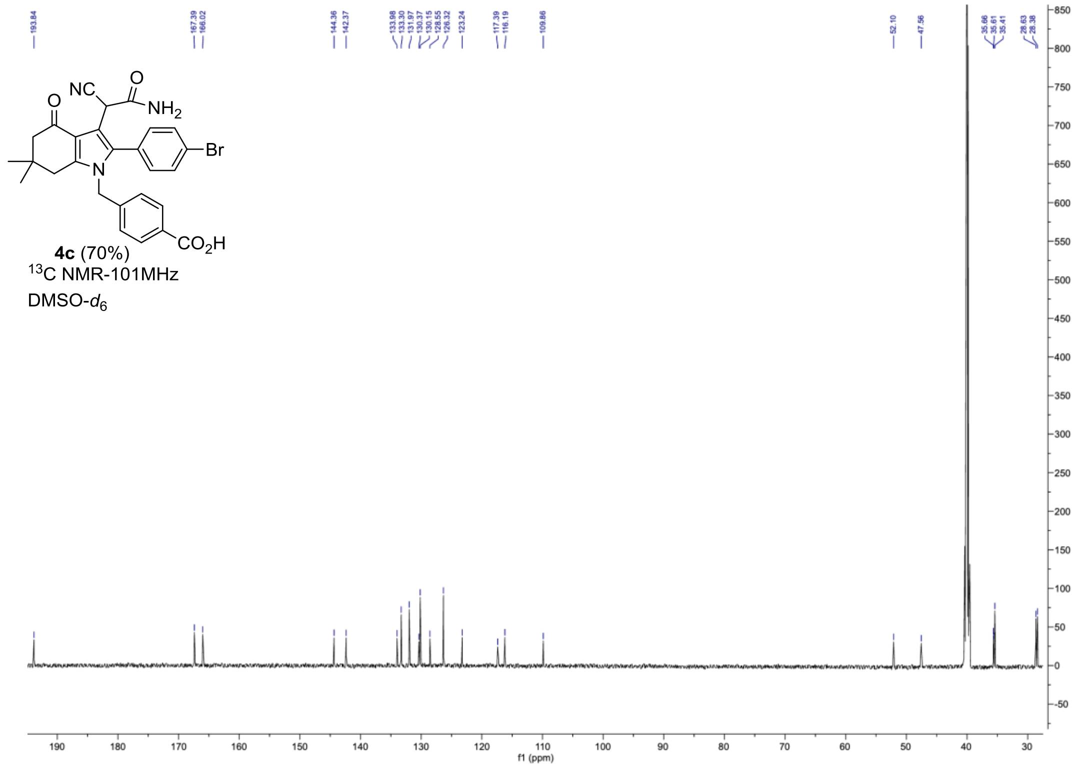
4c (70%)
13C NMR-101MHz
DMSO-d6
f1 (ppm)

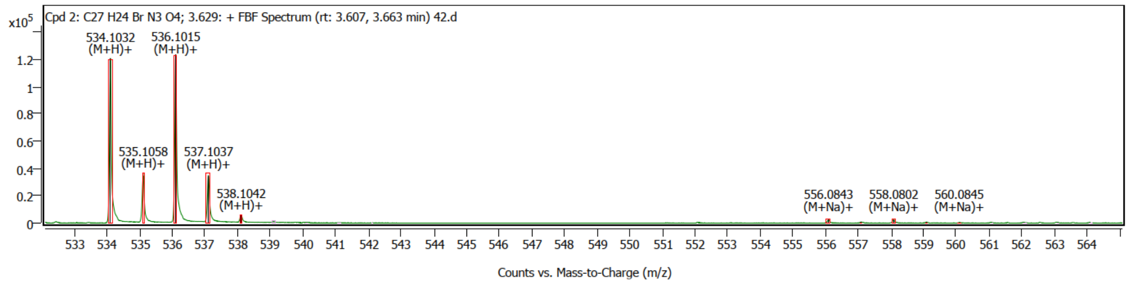
Cpd 2: C27 H24 Br N3 O4; 3.629: + FBF Spectrum (rt: 3.607, 3.663 min) 42.d
534.1032 (M+H)+
536.1015 (M+H)+
535.1058 (M+H)+
537.1037 (M+H)+
538.1042 (M+H)+
556.0843 (M+Na)+
558.0802 (M+Na)+
560.0845 (M+Na)+
Counts vs. Mass-to-Charge (m/z)

2-(3-(2-amino-1-cyano-2-oxoethyl)-2-(4-chlorophenyl)-6,6-dimethyl-4-oxo-4,5,6,7-tetrahydro-1H-indol-1-yl)acetic acid (**4d**)

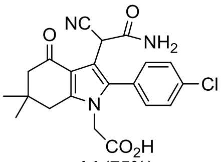

4d (75%)
^{1}H NMR-400MHz
DMSO-d_6

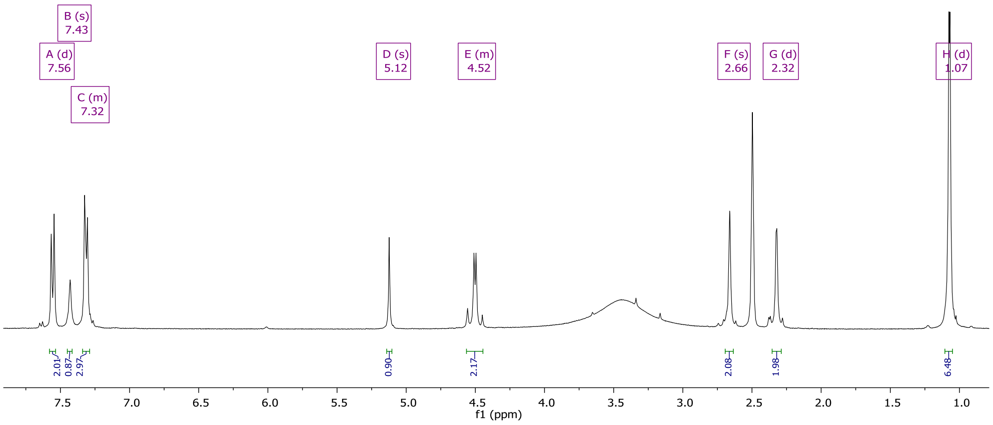

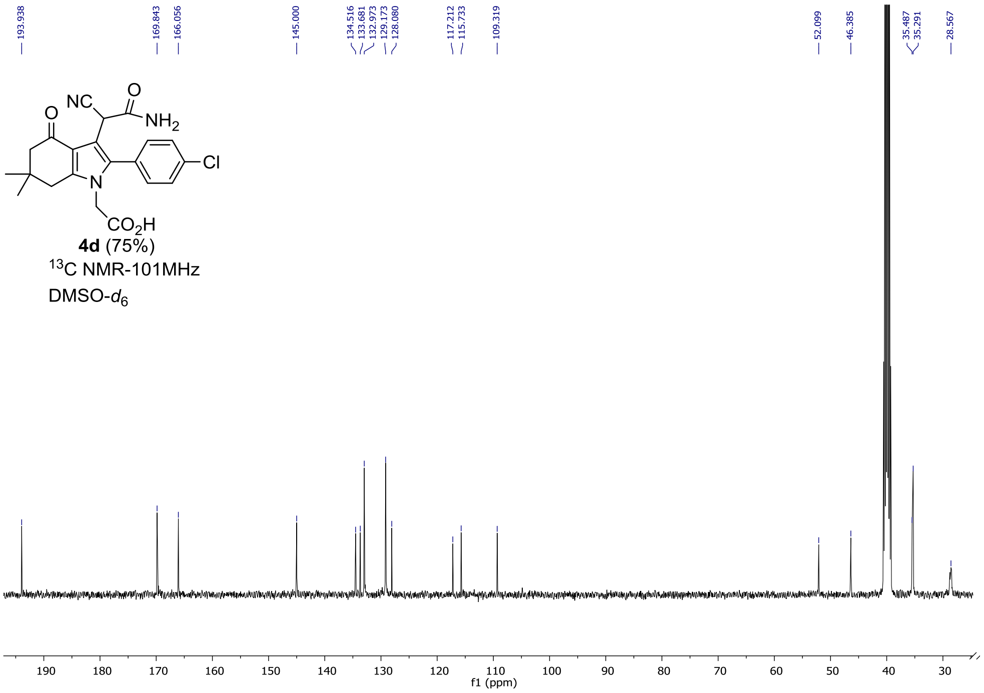

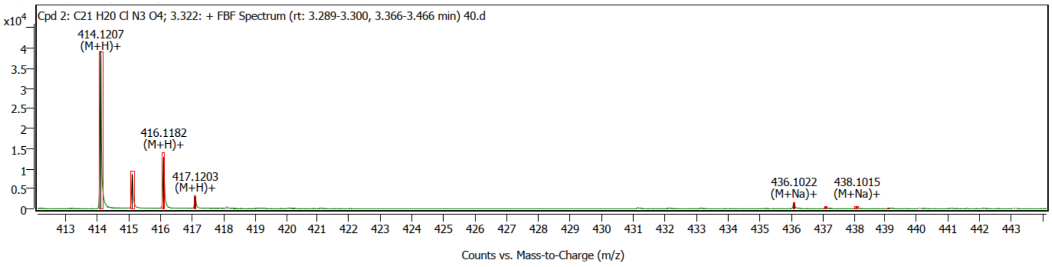

2-(2-(4-chlorophenyl)-3-(dicyanomethyl)-2-hydroxy-6,6-dimethyl-4-oxo-2,3,4,5,6,7-hexahydro-1H-indol-1-yl)acetic acid (**4d***)

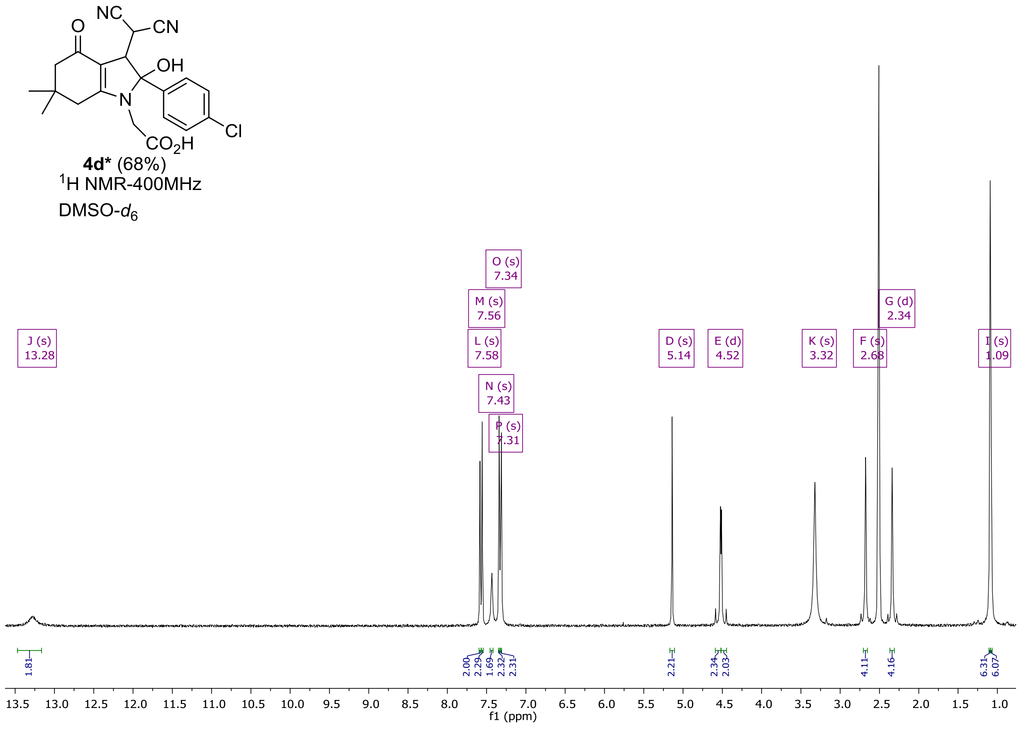

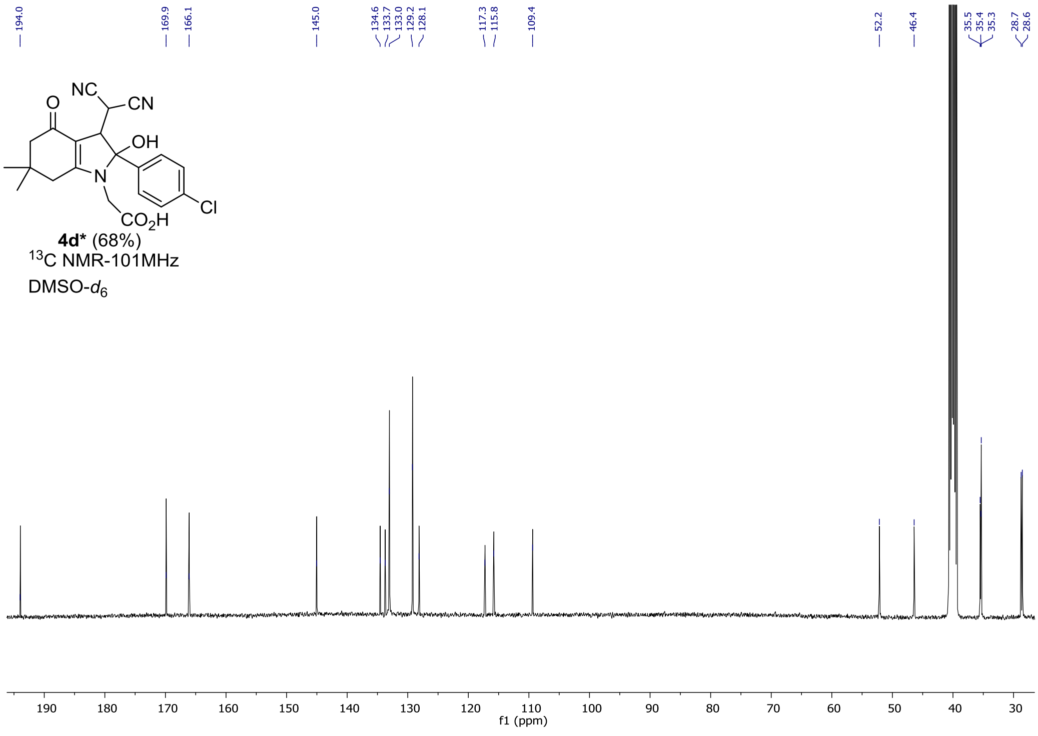

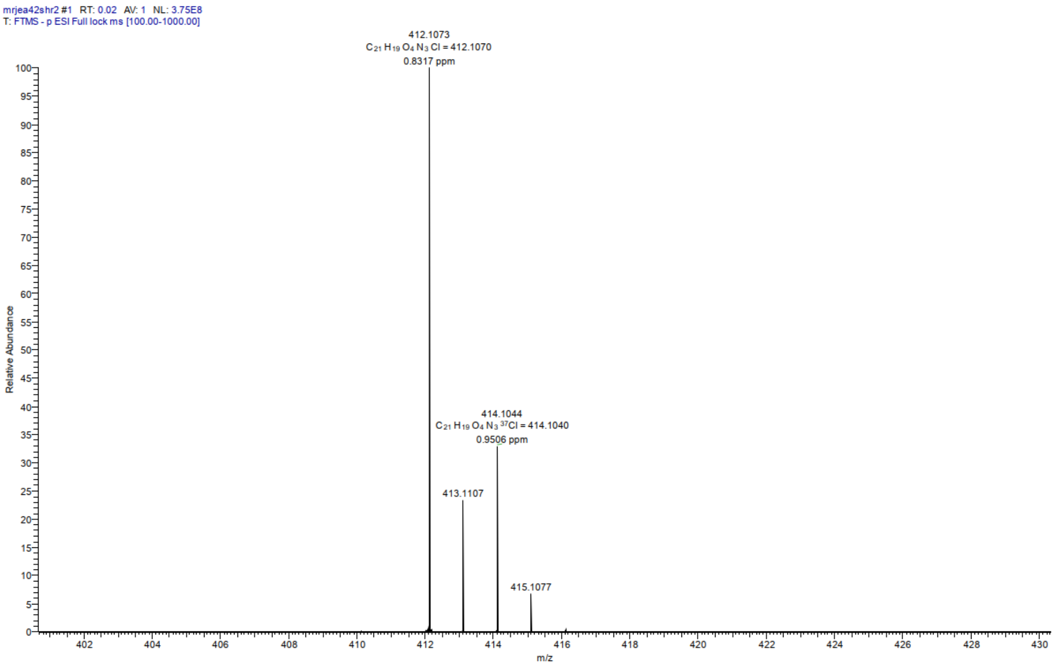

2-(3-(2-amino-1-cyano-2-oxoethyl)-2-(3-fluorophenyl)-6,6-dimethyl-4-oxo-4,5,6,7-tetrahydro-1H-indol-1-yl)acetic acid (**4e**)

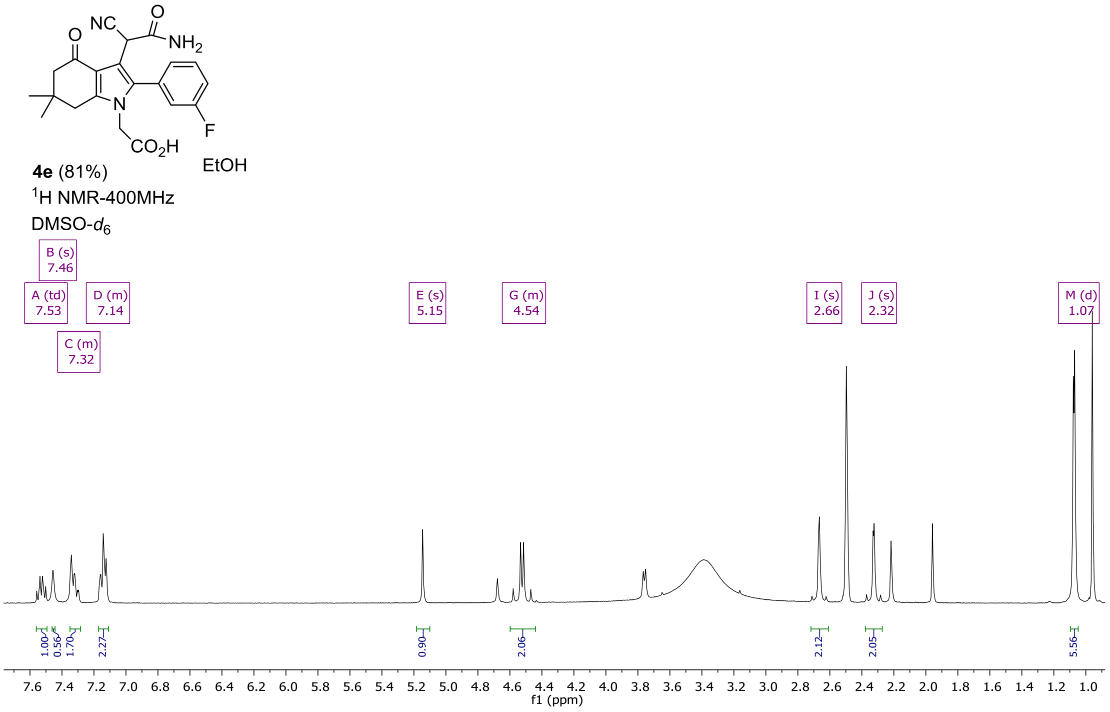

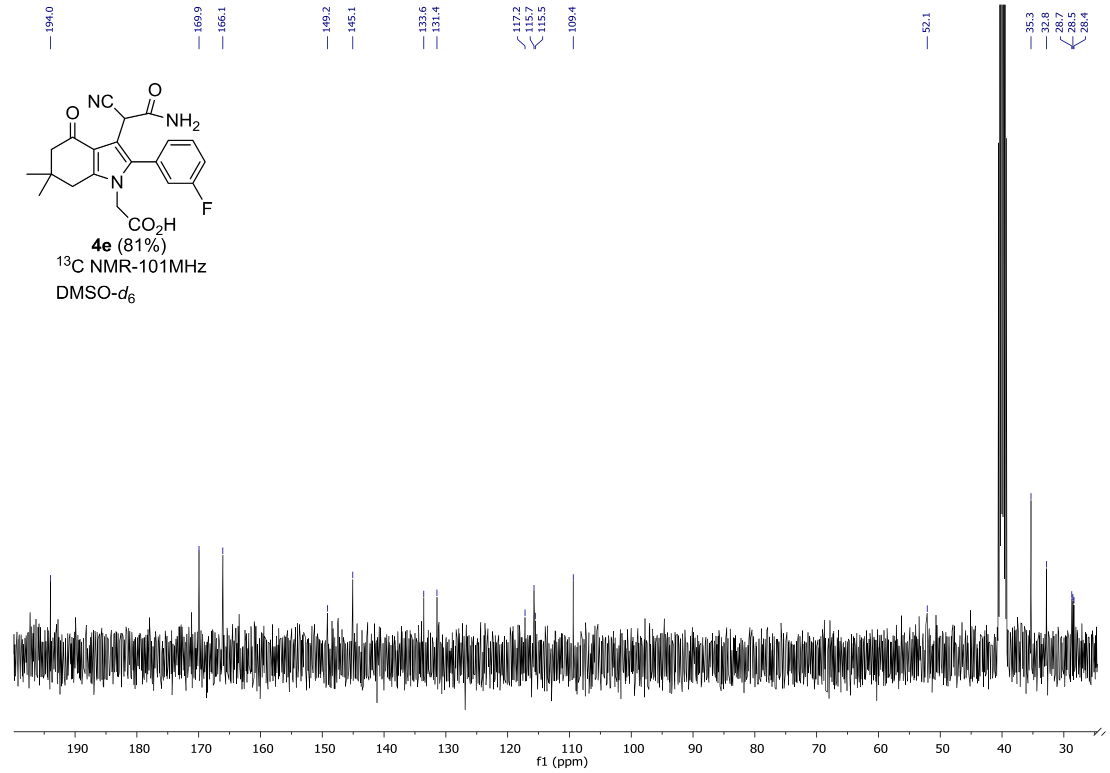
194.0
169.9
166.1
149.2
145.1
133.6
131.4
117.2
115.7
115.5
109.4
52.1
35.3
32.8
28.7
28.5
28.4
NC
O
NH2
O
N
F
CO2H
4e (81%)
13C NMR-101MHz
DMSO-d6
190
180
170
160
150
140
130
120
110
100
90
80
70
60
50
40
30
f1 (ppm)

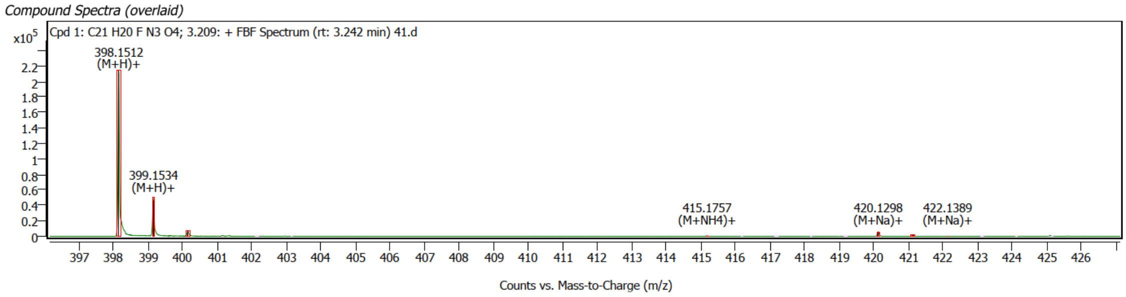
Compound Spectra (overlaid)
x10^5
Cpd 1: C21 H20 F N3 O4; 3.209: + FBF Spectrum (rt: 3.242 min) 41.d
398.1512
(M+H)+
399.1534
(M+H)+
415.1757
(M+NH4)+
420.1298
(M+Na)+
422.1389
(M+Na)+
Counts vs. Mass-to-Charge (m/z)

methyl 2-(3-(2-amino-1-cyano-2-oxoethyl)-2-(4-bromophenyl)-6,6-dimethyl-4-oxo-4,5,6,7-tetrahydro-1H-indol-1-yl)acetate (**4f**)

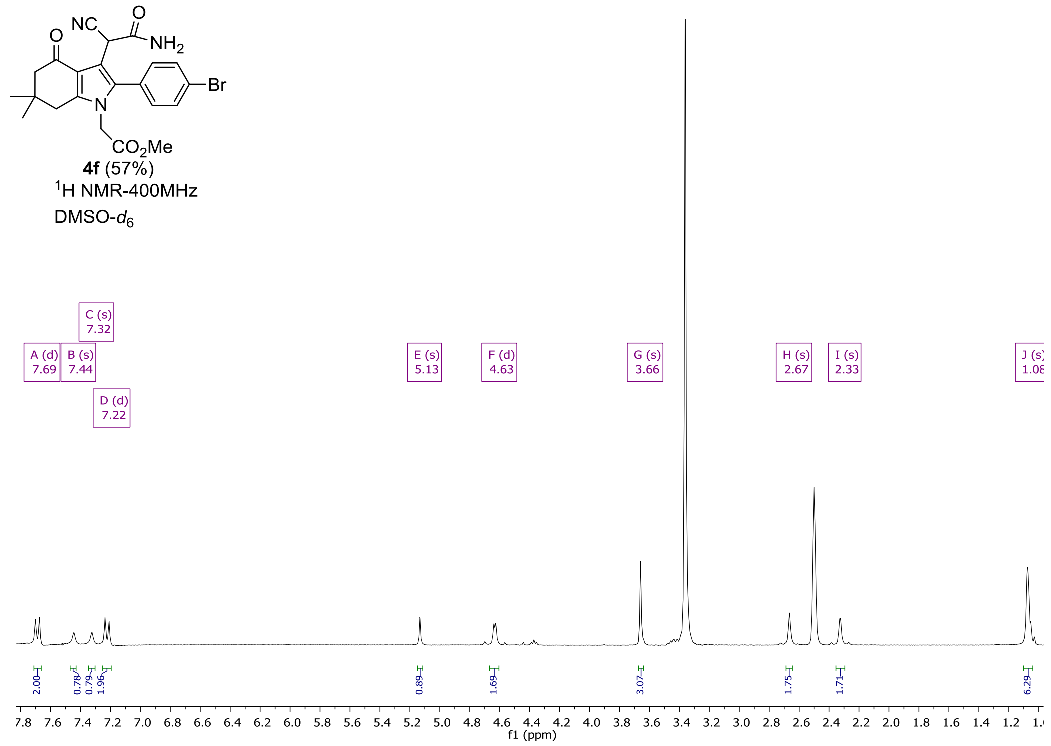

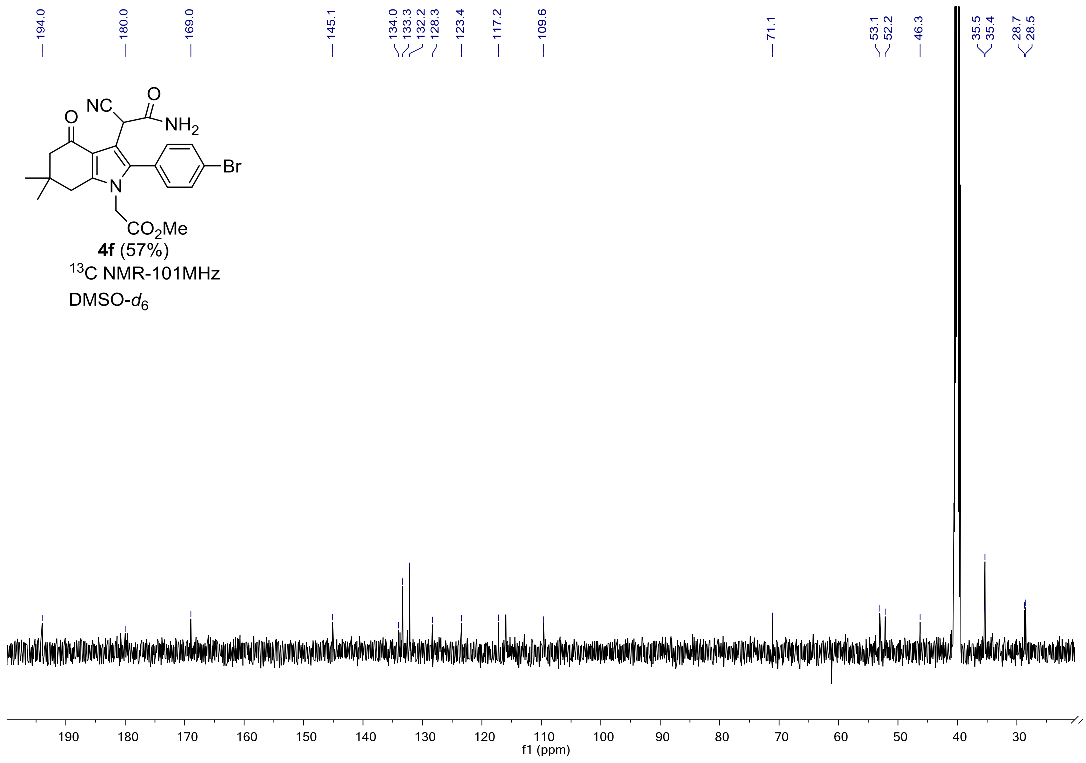

2-(2-(4-Bromophenyl)-6,6-dimethyl-4-oxo-1-phenyl-4,5,6,7-tetrahydro-1H-indol-3-yl)-2-cyanoacetamide (**4g**)

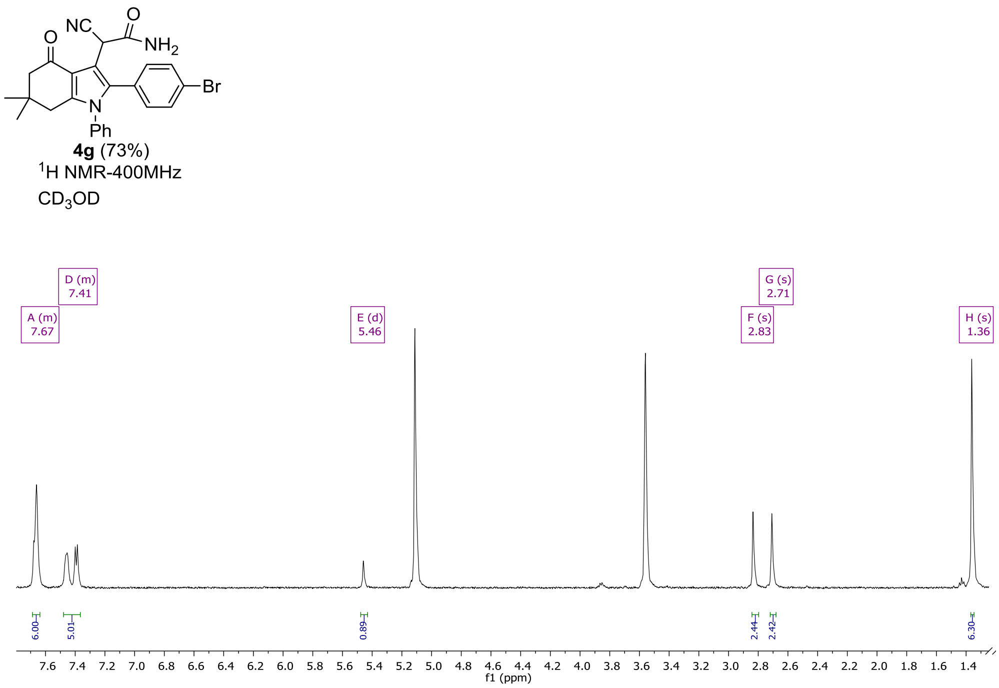

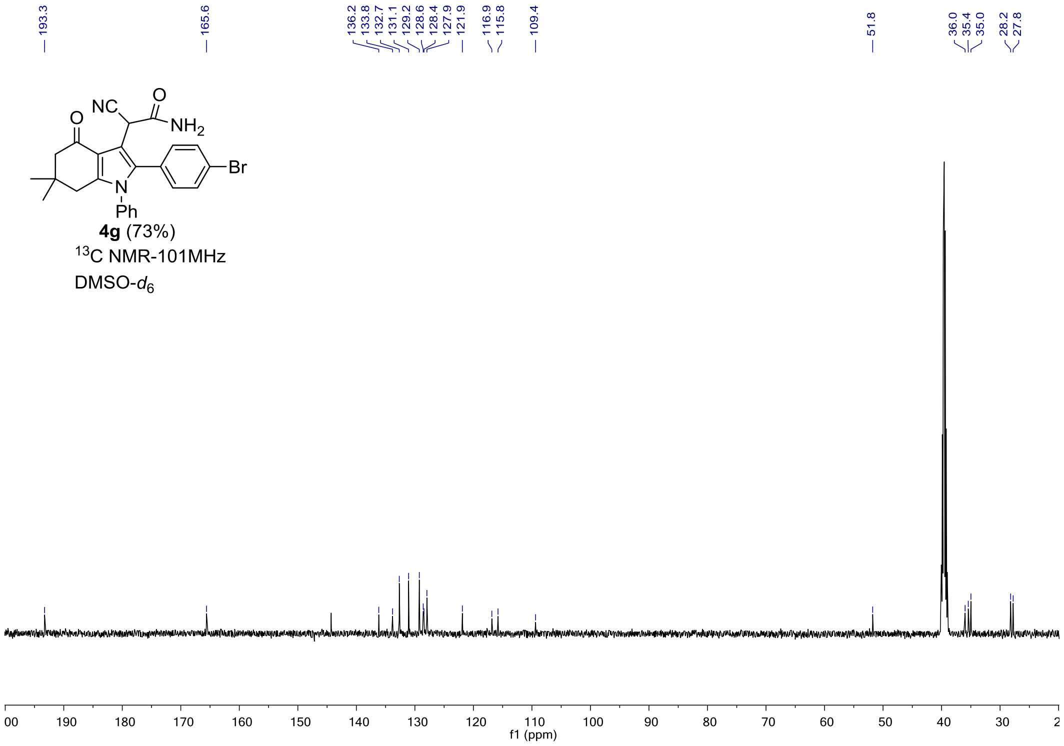

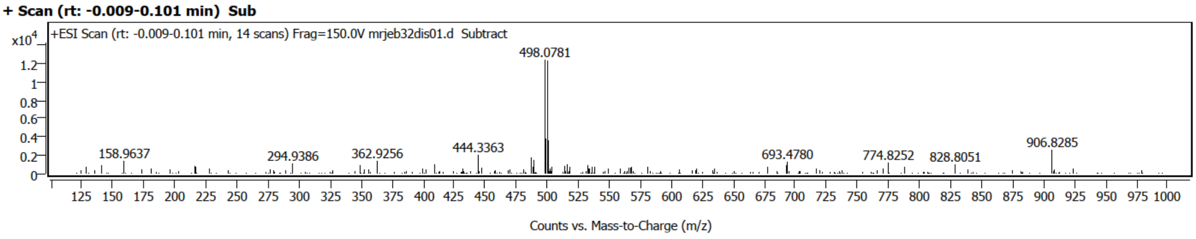

4-((3-(2-amino-1-cyano-2-oxoethyl)-2-(4-chlorophenyl)-6,6-dimethyl-4-oxo-4,5,6,7-tetrahydro-1H-indol-1-yl)methyl)benzoic acid (**4h**)

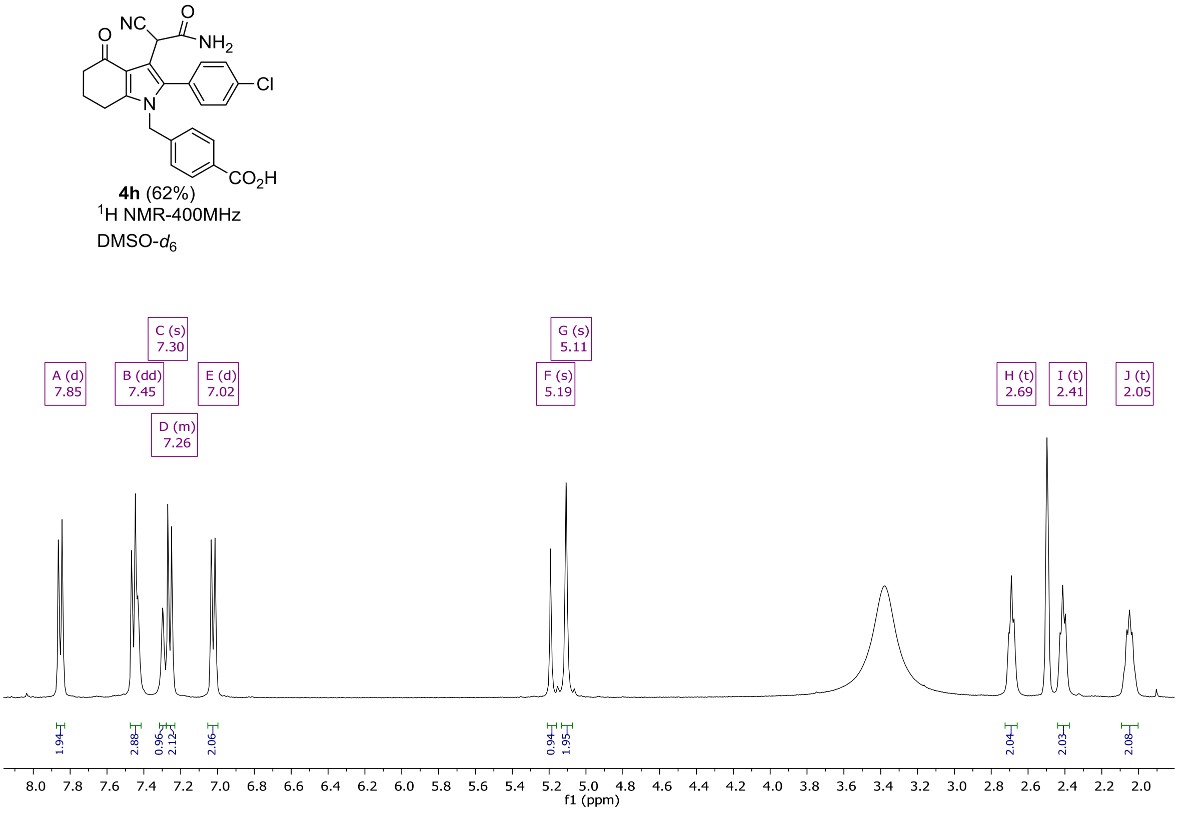

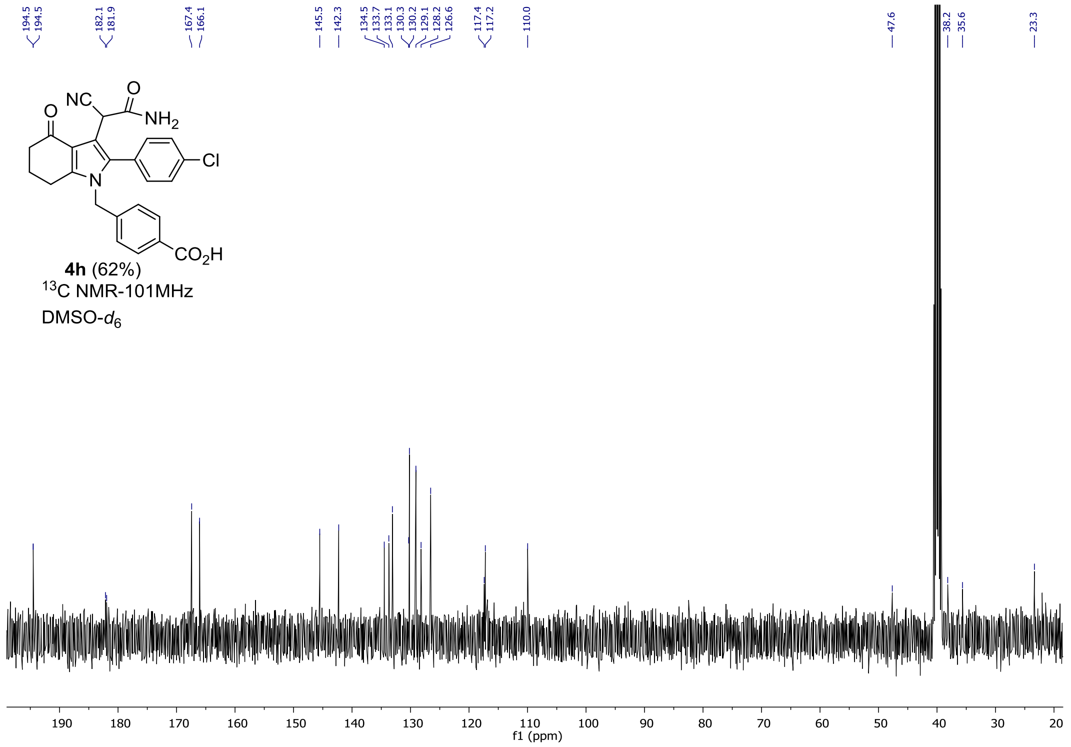

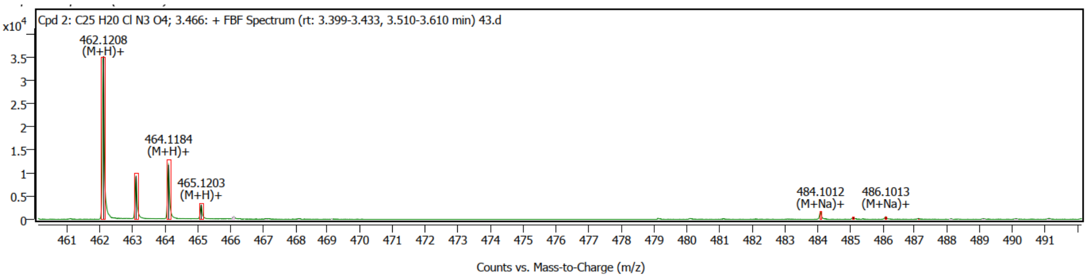

2-(2-(4-bromophenyl)-3-(cyanomethyl)-6,6-dimethyl-4-oxo-4,5,6,7-tetrahydro-1H-indol-1-yl)acetic acid (**6**)

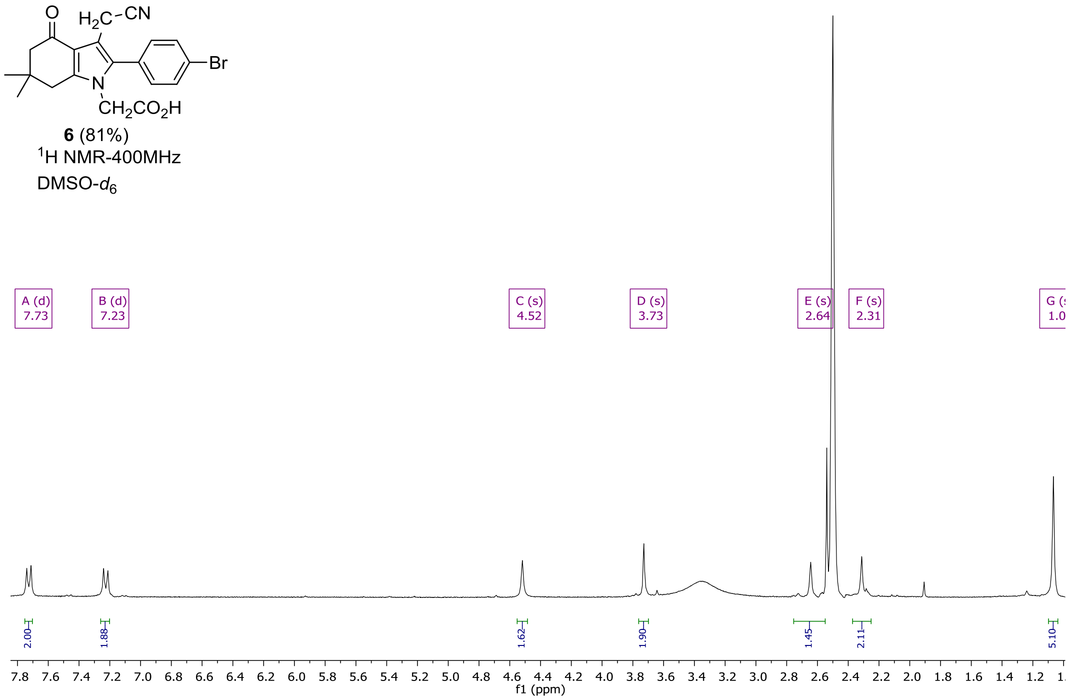

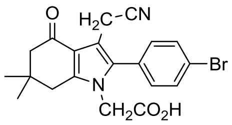

6 (81%)

^{13}C NMR-101MHz

DMSO-d_6

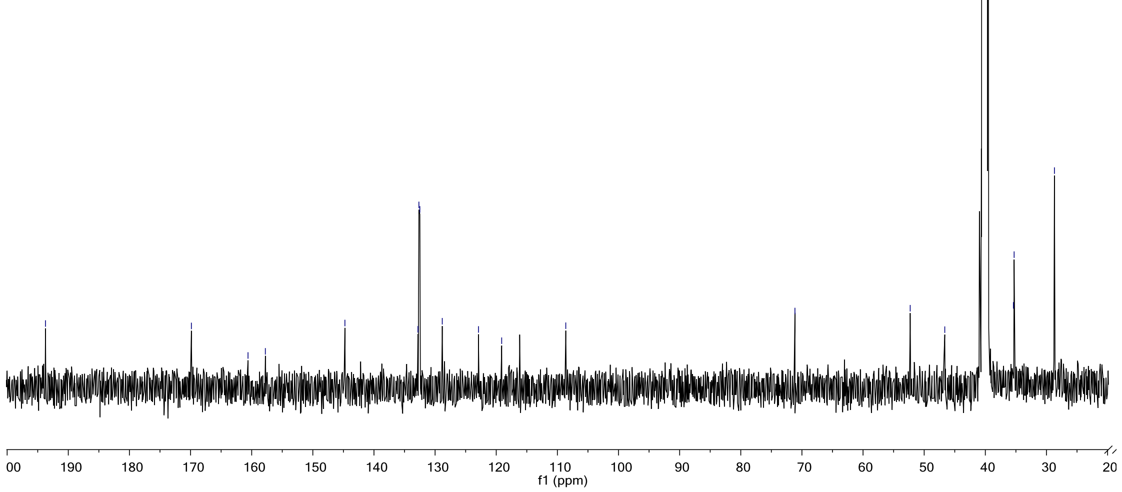

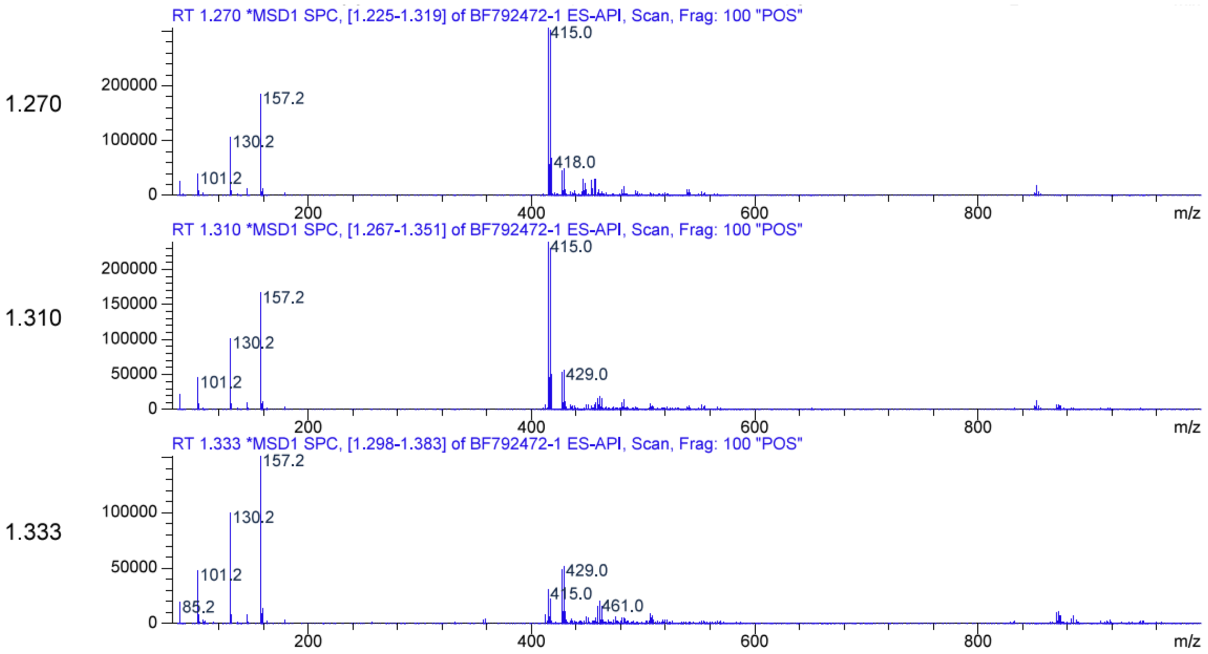

2,2'-(2-(4-Bromophenyl)-6,6-dimethyl-4-oxo-4,5,6,7-tetrahydro-1H-indole-1,3-diyl)diacetic acid (**7a**)

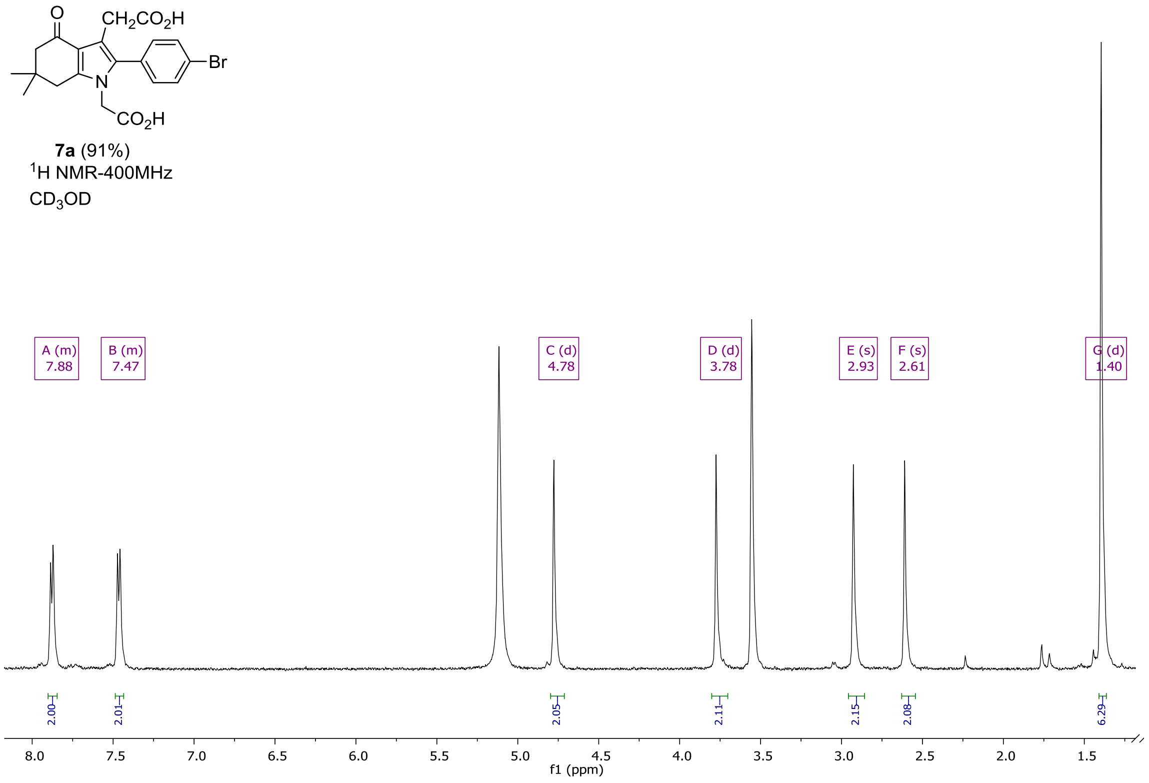

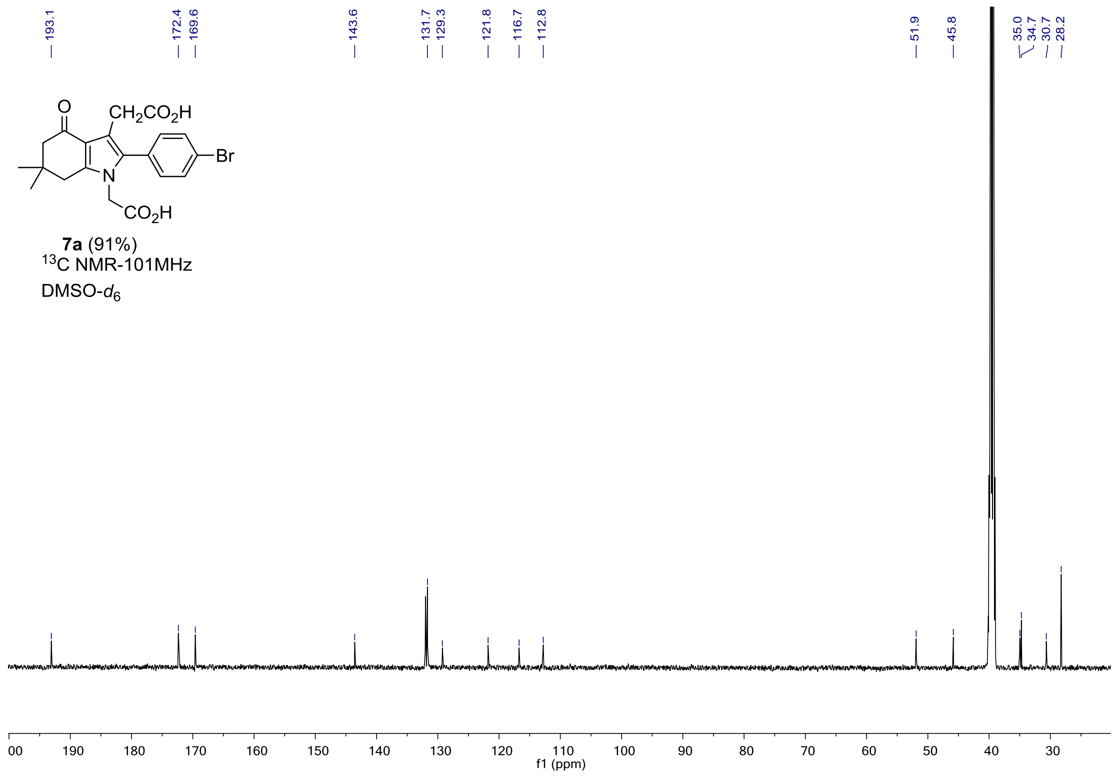

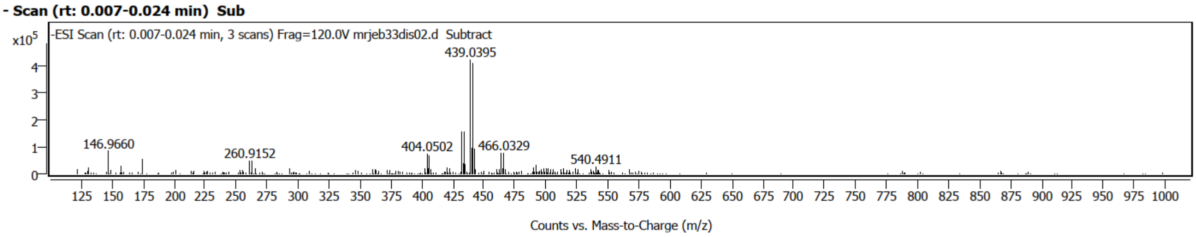

2,2'-(6,6-Dimethyl-4-oxo-2-(p-tolyl)-4,5,6,7-tetrahydro-1H-indole-1,3-diyl)diacetic acid (**7b**)

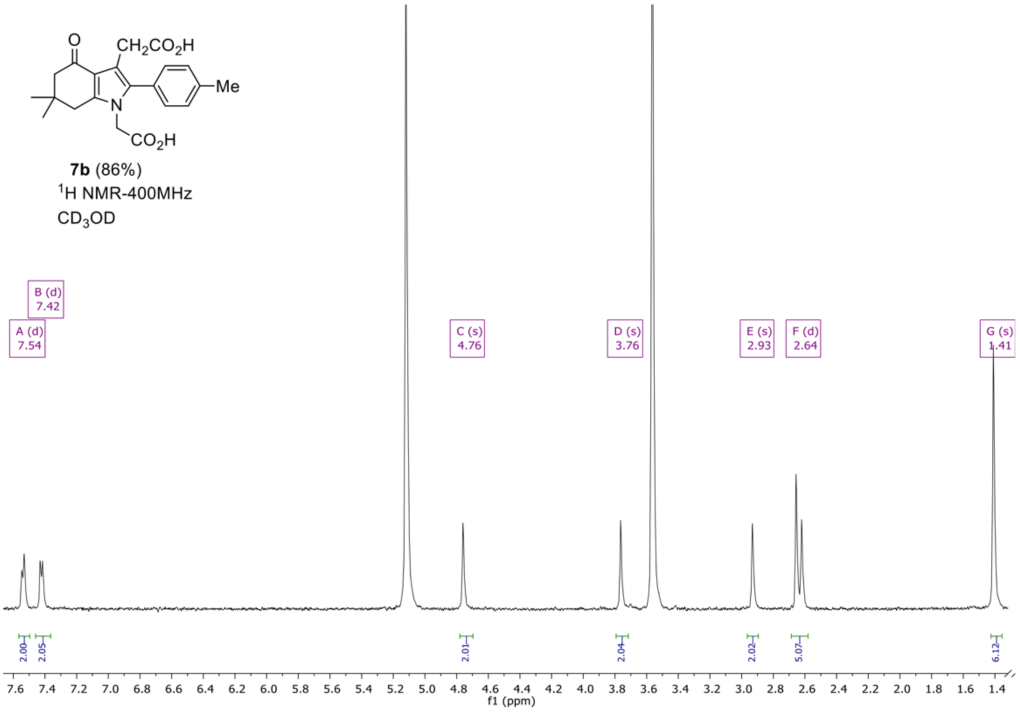

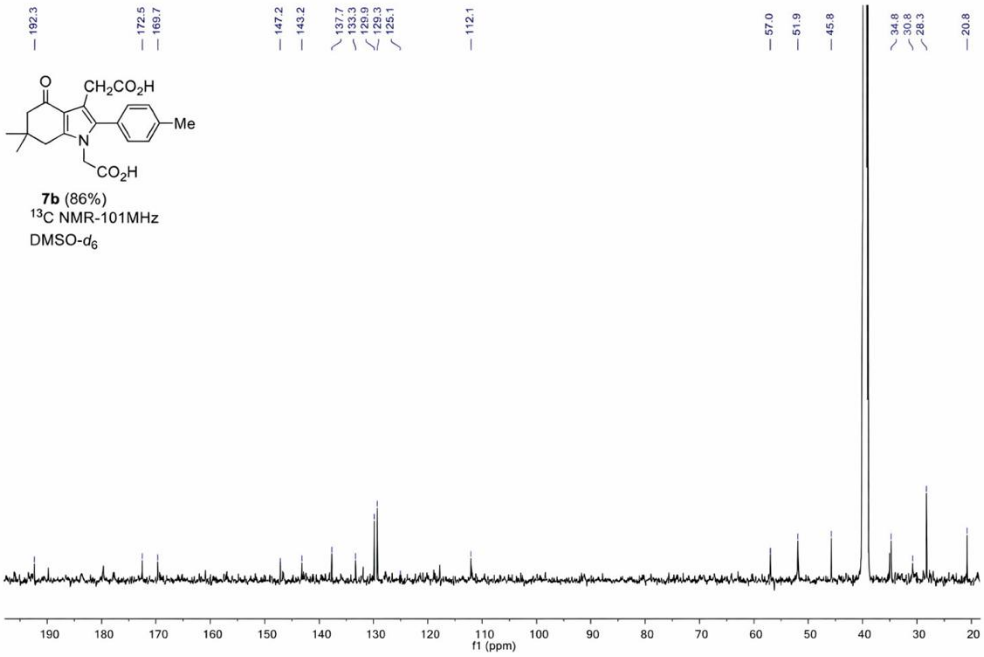

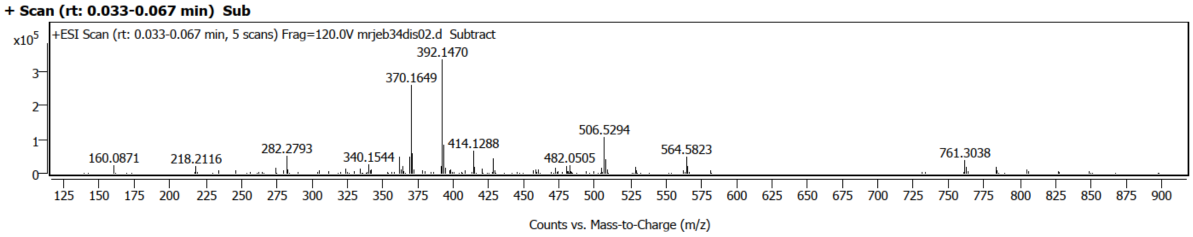

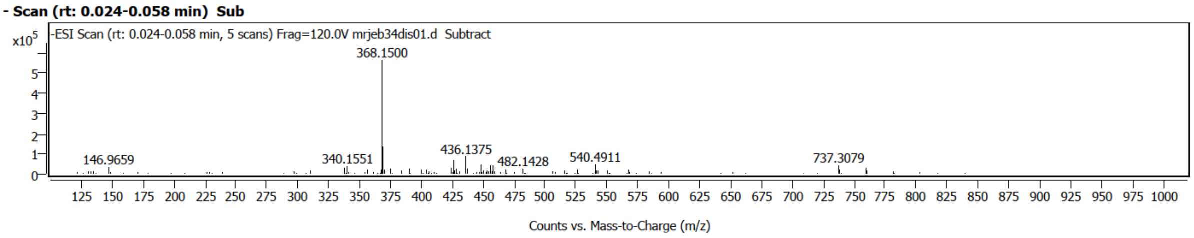

2-(2-(4-Bromophenyl)-6,6-dimethyl-4-oxo-1-phenyl-4,5,6,7-tetrahydro-1H-indol-3-yl)acetic acid (**7c**)

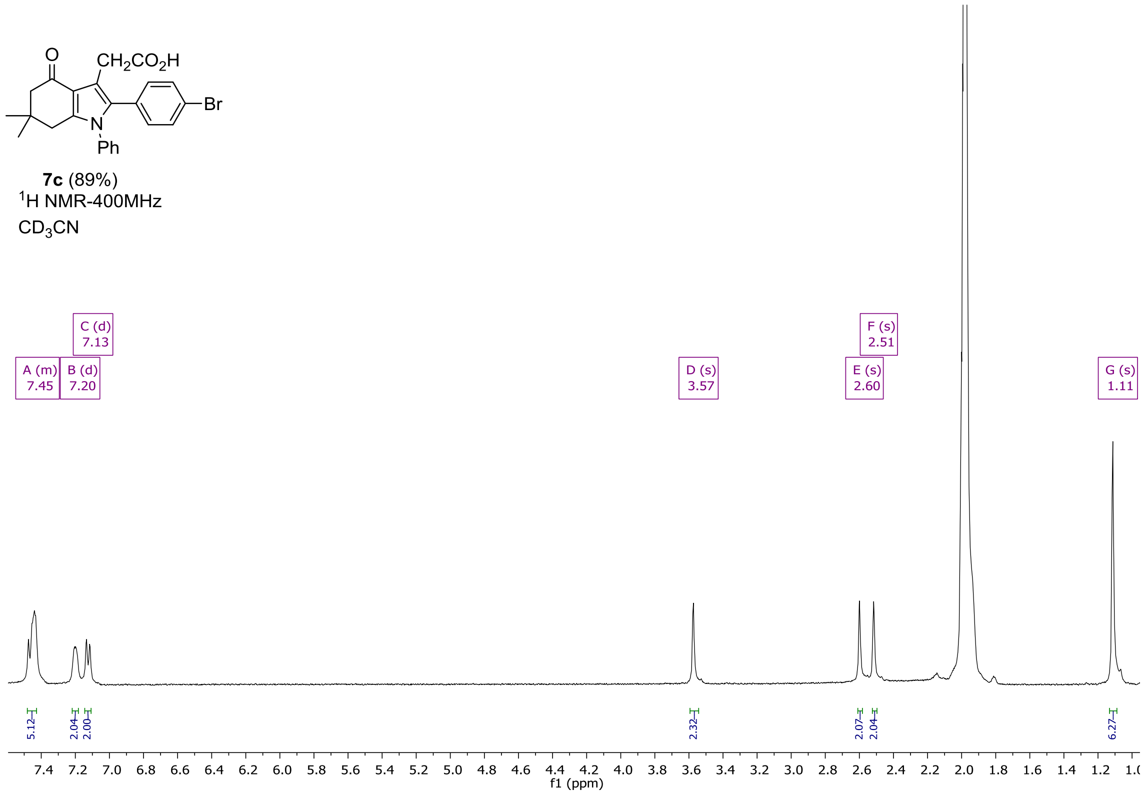

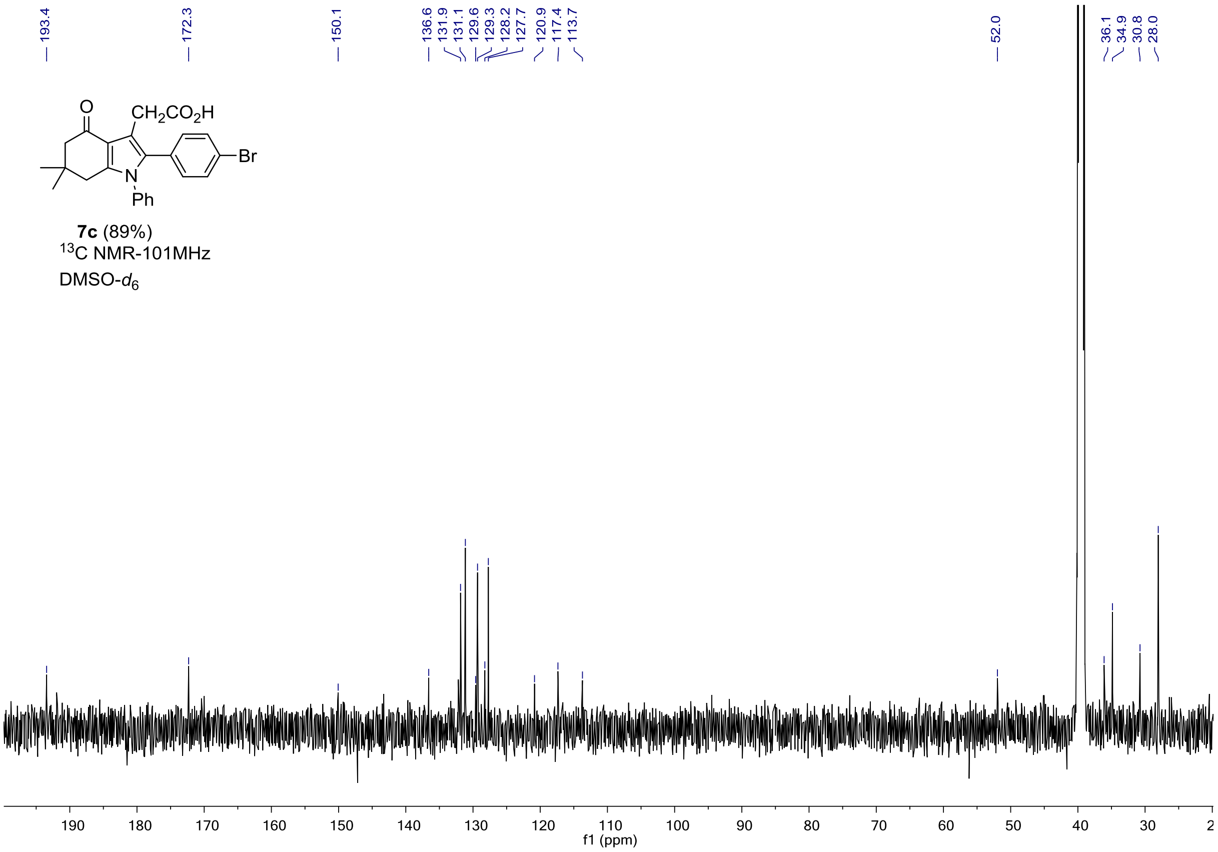
193.4
172.3
150.1
136.6
131.9
131.1
129.6
129.3
128.2
127.7
120.9
117.4
113.7
52.0
36.1
34.9
30.8
28.0
O
CH2CO2H
Br
N
Ph
7c (89%)
13C NMR-101MHz
DMSO-d6
f1 (ppm)

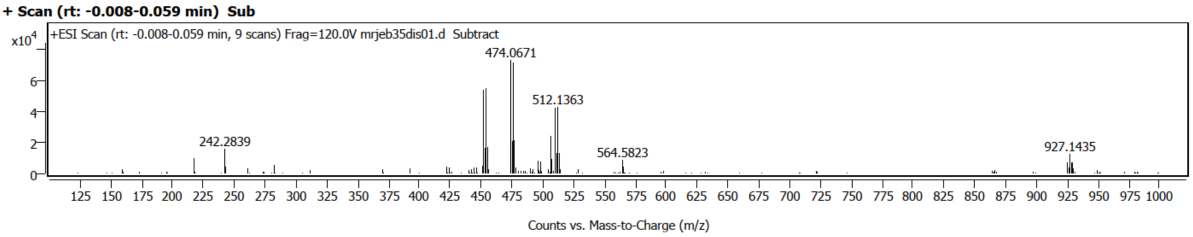
+ Scan (rt: -0.008-0.059 min) Sub
+ESI Scan (rt: -0.008-0.059 min, 9 scans) Frag=120.0V mrjeb35dis01.d Subtract
x10^4
474.0671
512.1363
242.2839
564.5823
927.1435
Counts vs. Mass-to-Charge (m/z)

(E)-2,2'-(2-(4-Bromophenyl)-4-(hydroxyimino)-6,6-dimethyl-4,5,6,7-tetrahydro-1H-indole-1,3-diyl)diacetic acid (**8a**)

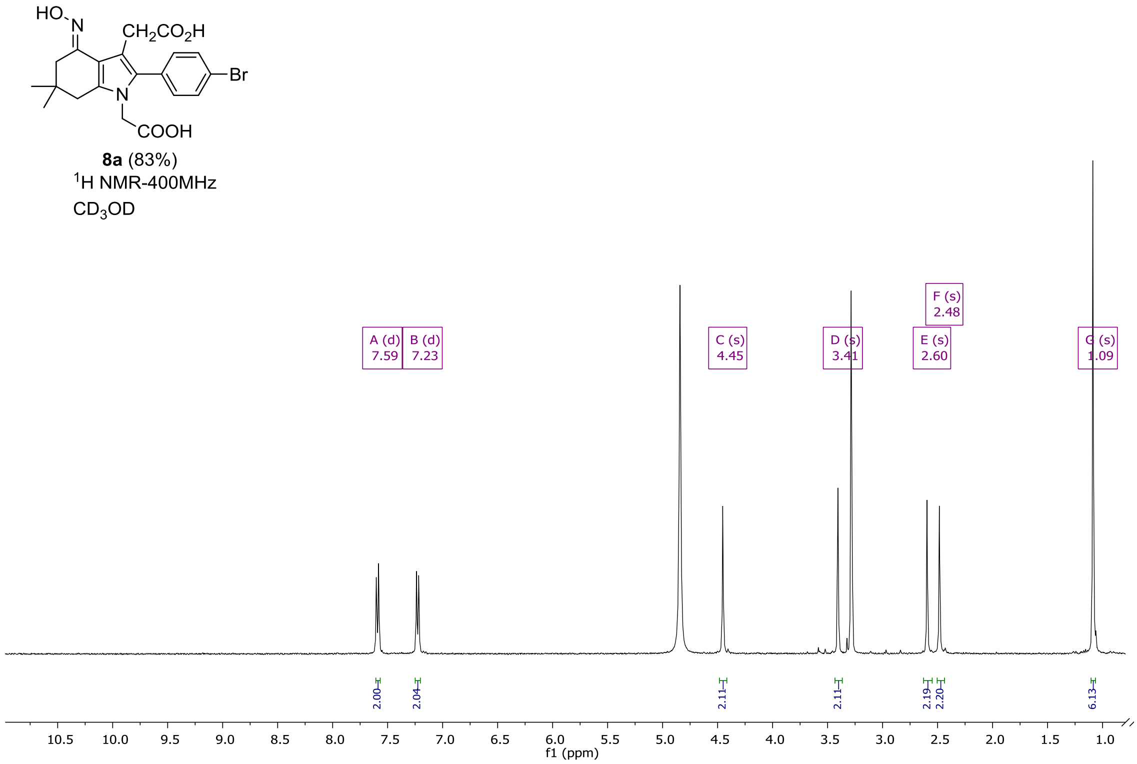

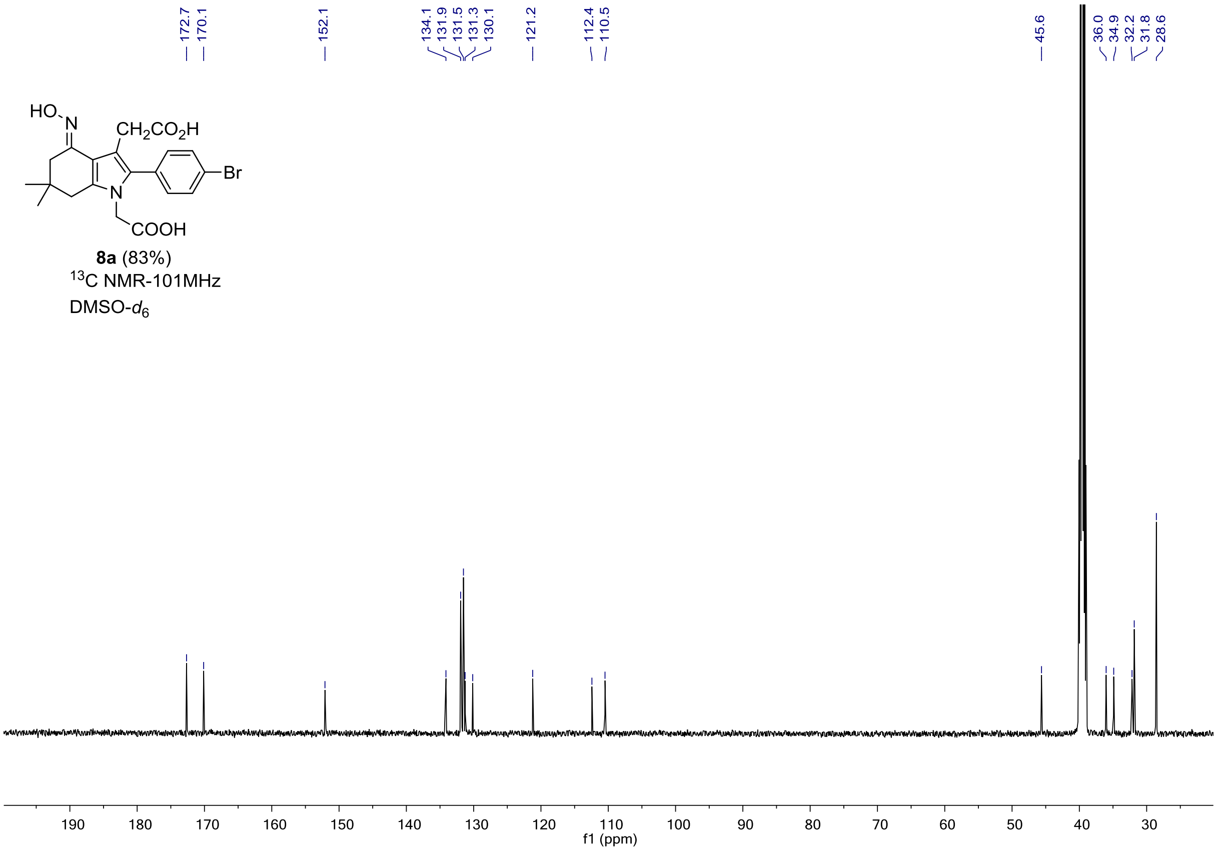

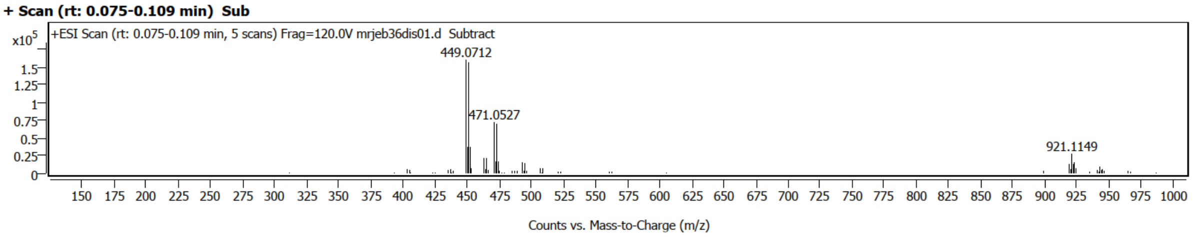

(E)-2-(2-(4-Bromophenyl)-4-(hydroxyimino)-6,6-dimethyl-1-phenyl-4,5,6,7-tetrahydro-1H-indol-3-yl)acetic acid (**8b**)

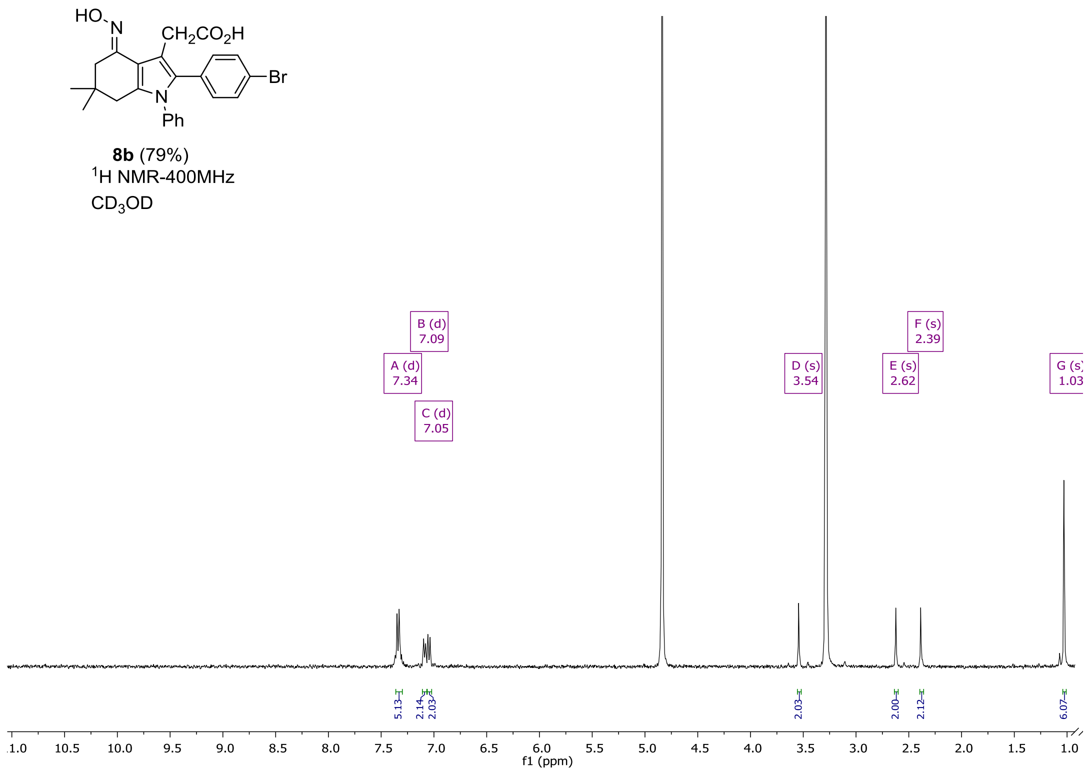

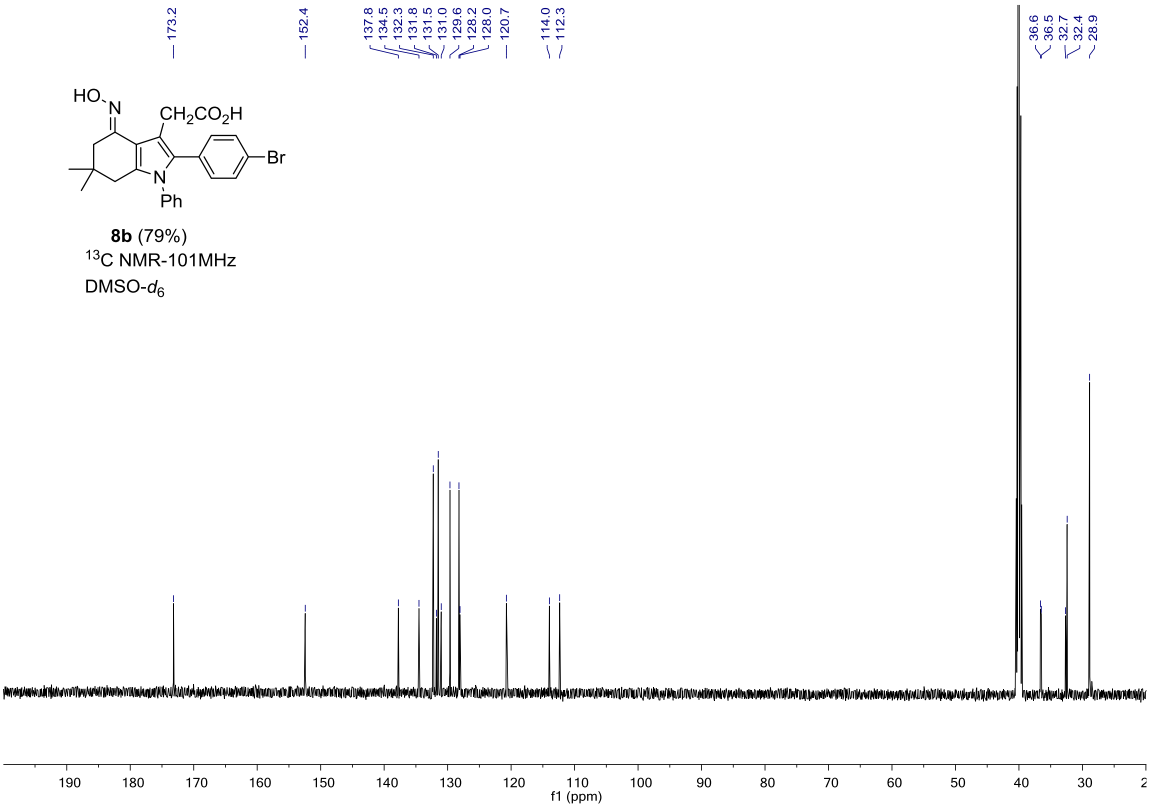

8b (79%)
^{13}C NMR-101MHz
DMSO-d_6

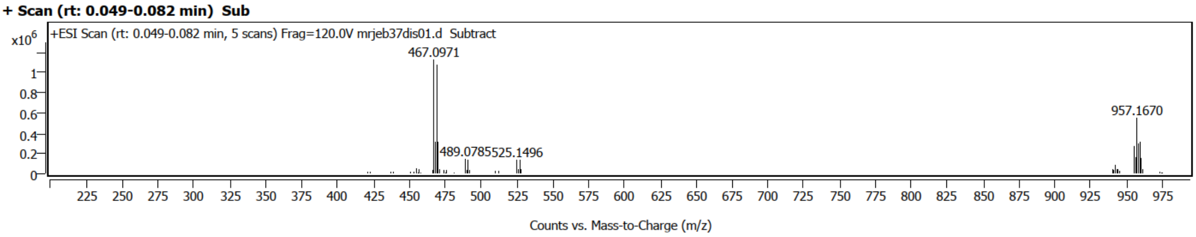

2,2'-(2-(4-Bromophenyl)-7,7-dimethyl-5-oxo-5,6,7,8-tetrahydropyrrolo[3,2-b]azepine-1,3(4H)-diyl)diacetic acid (**9**)

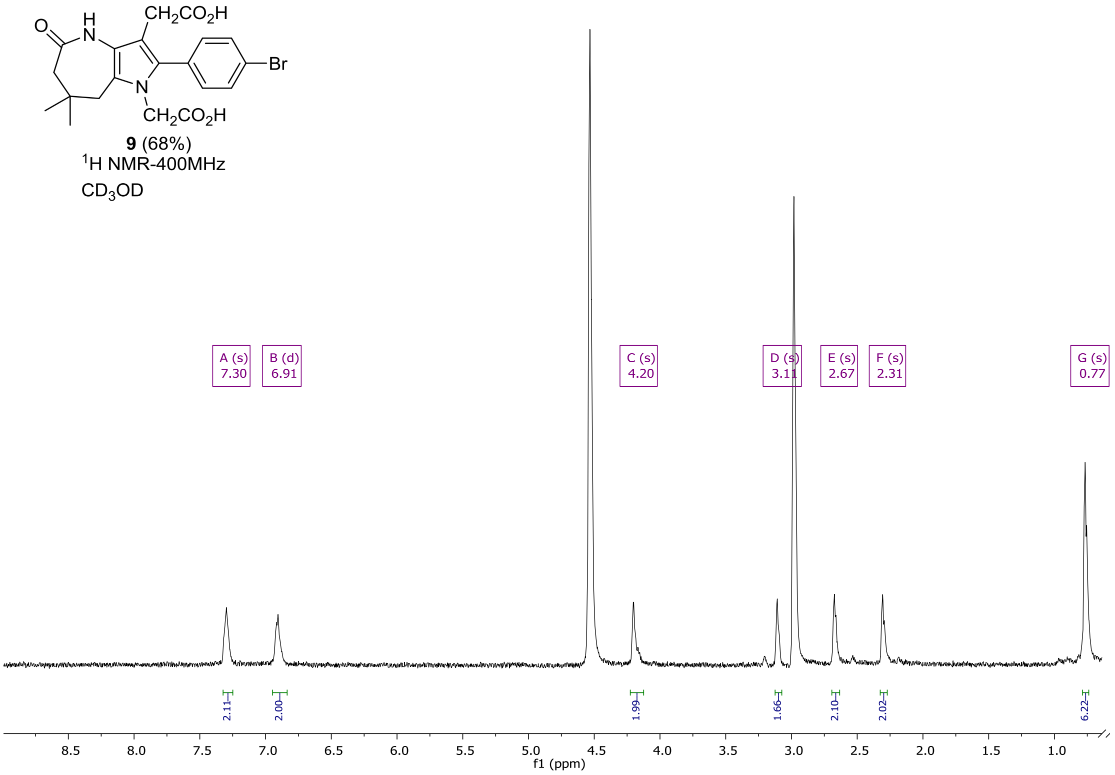

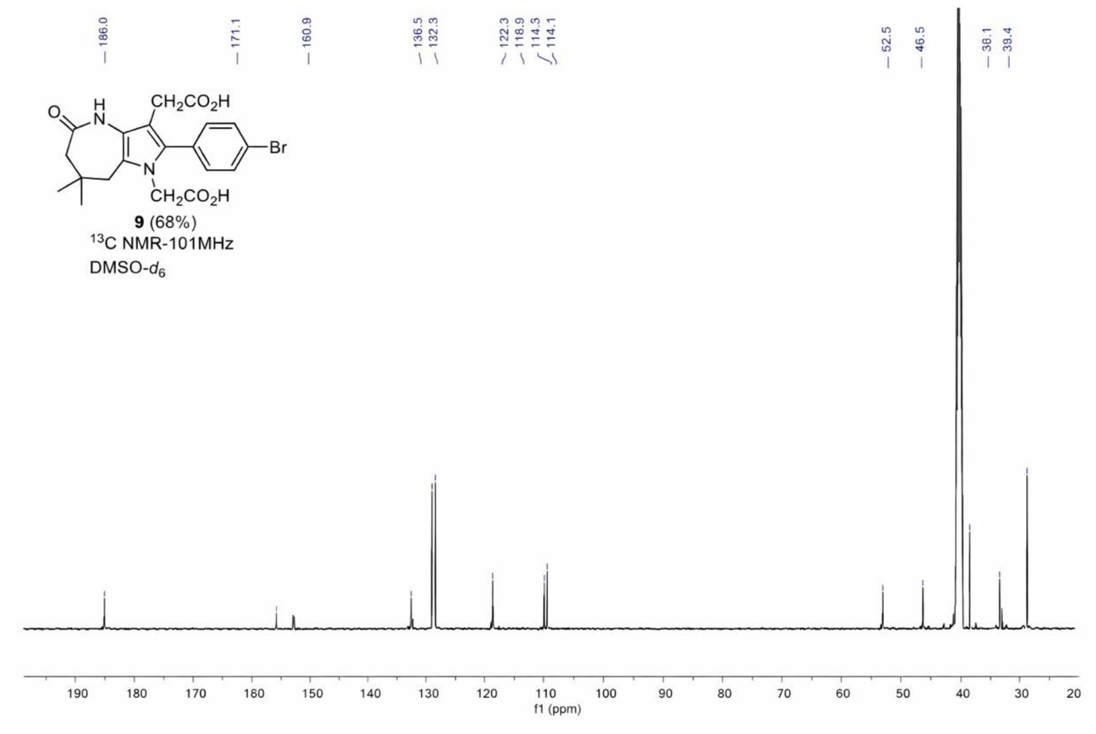

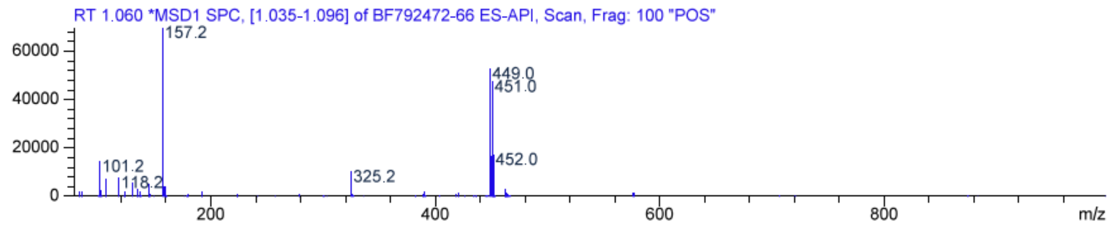

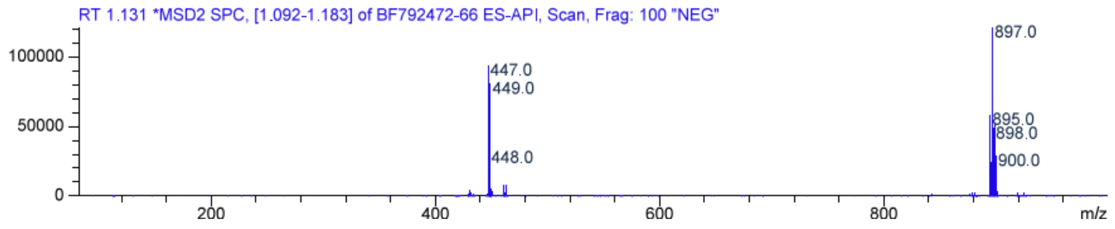

1-((4-chlorophenyl)amino)-3-methyl-1-oxobutan-2-yl 2-(3-(2-amino-1-cyano-2-oxoethyl)-6,6-dimethyl-4-oxo-2-phenyl-4,5,6,7-tetrahydro-1H-indol-1-yl)acetate (**12a**)

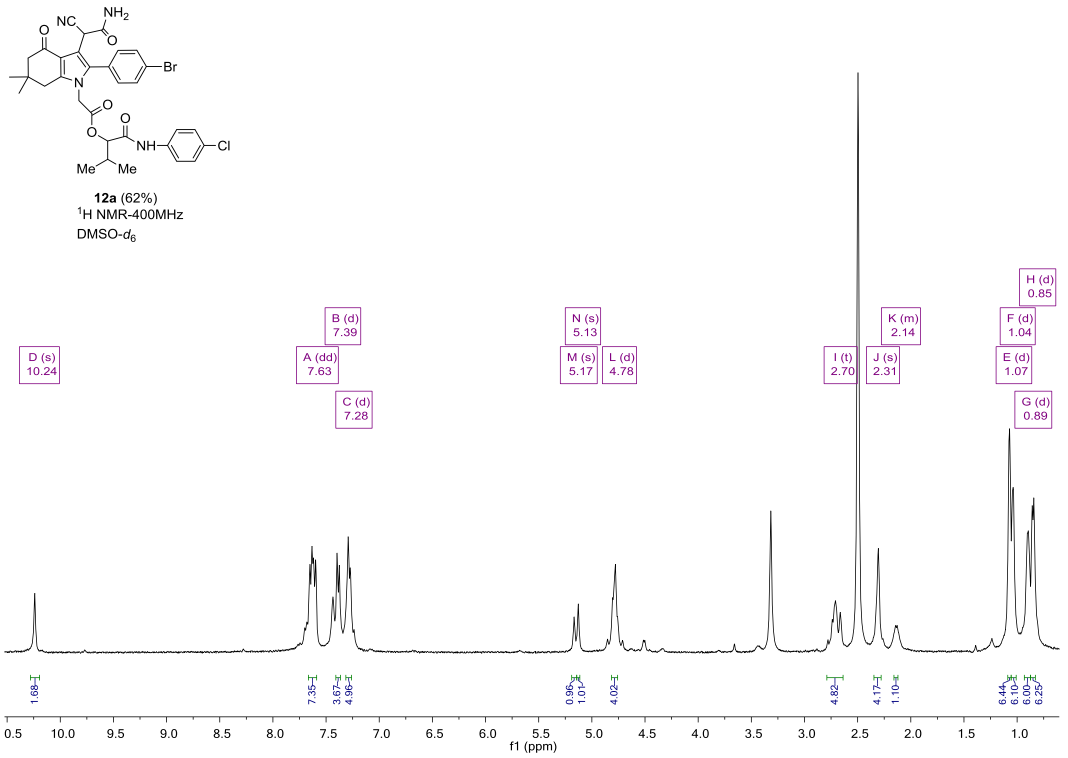

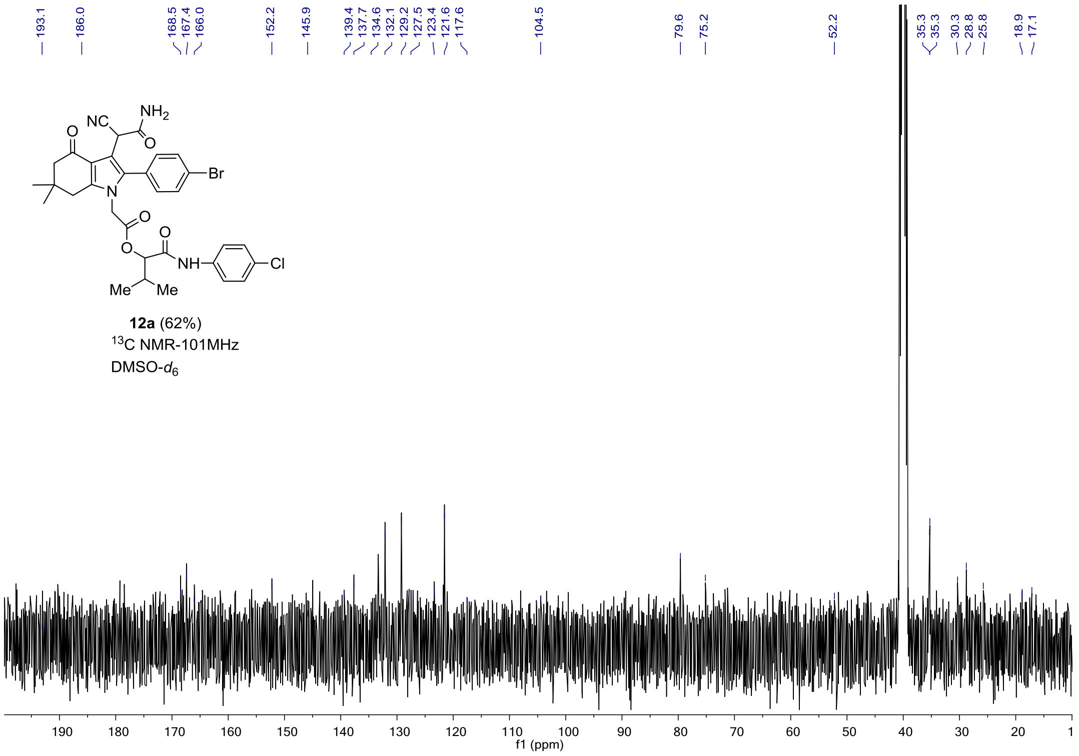

12a (62%)
^{13}C NMR-101MHz
DMSO-d_6

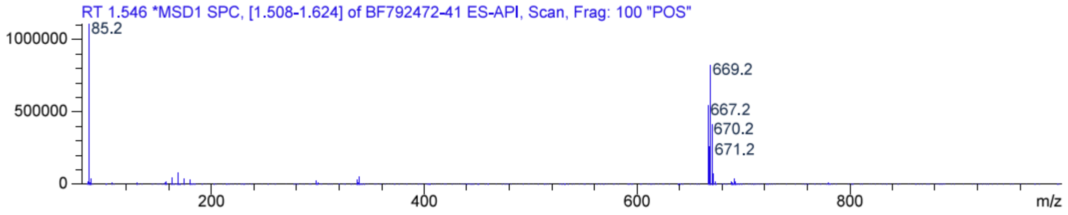

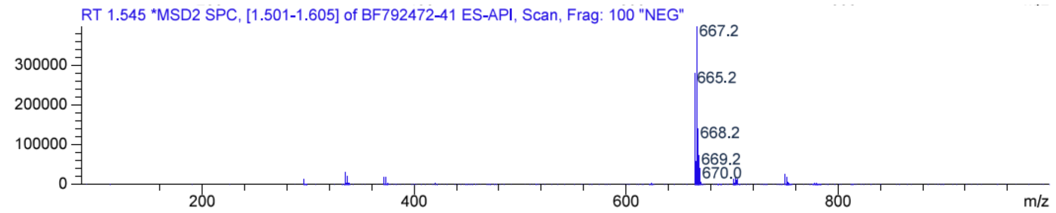

1-(Tert-butylamino)-1-oxopentan-2-yl-2-(3-(1,3-dimethyl-2,4,6-trioxohexahydropyrimidin-5-yl)-6,6-dimethyl-4-oxo-2-phenyl-4,5,6,7-tetrahydro-1H-indol-1-yl)acetate (**12b**)

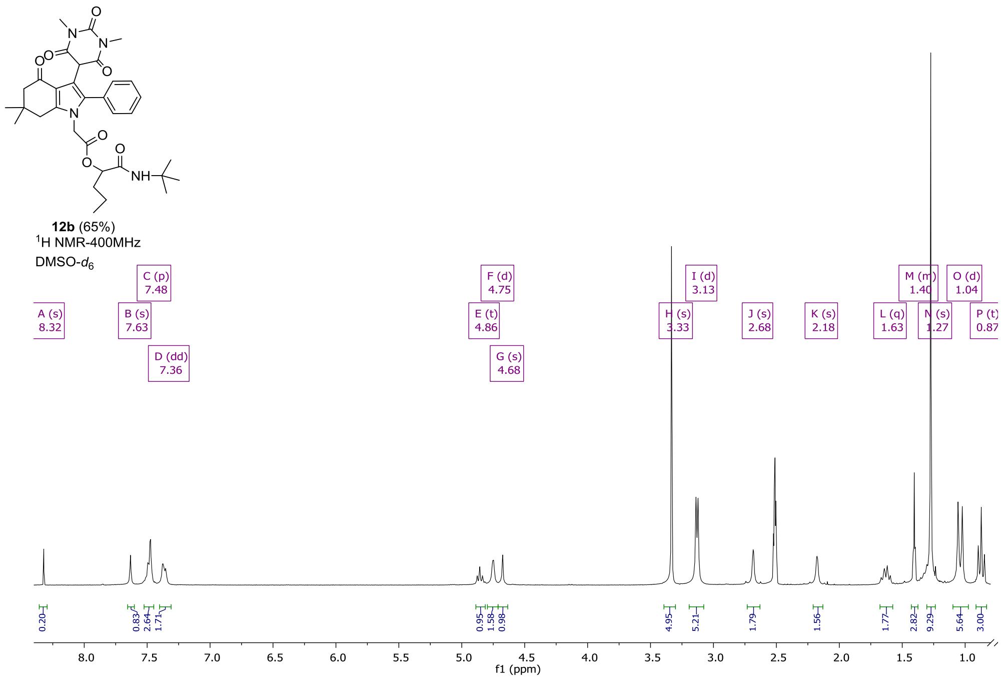

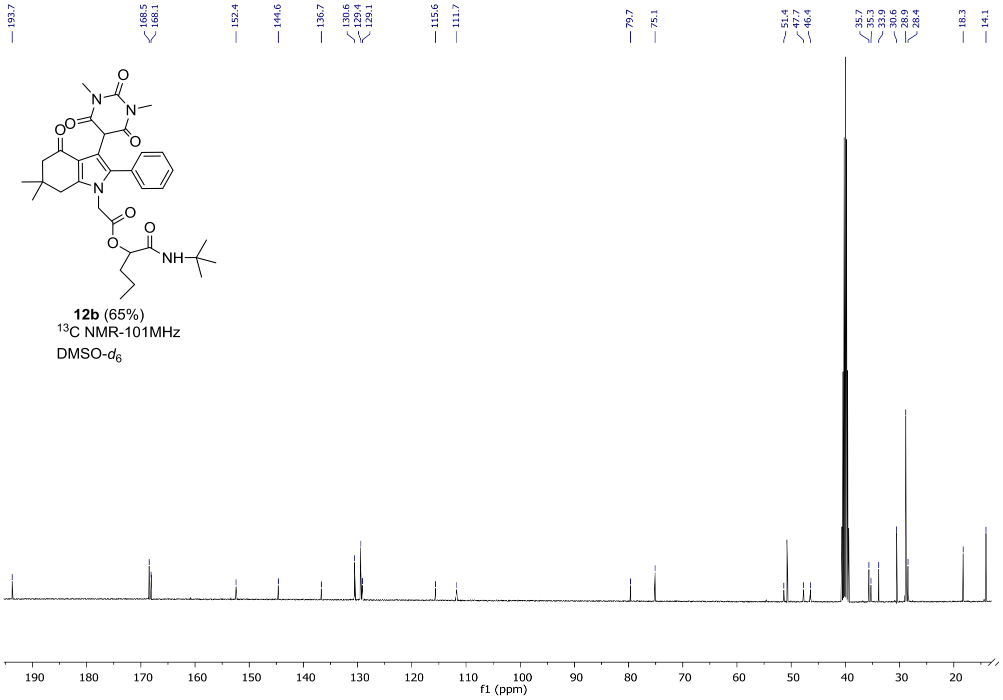

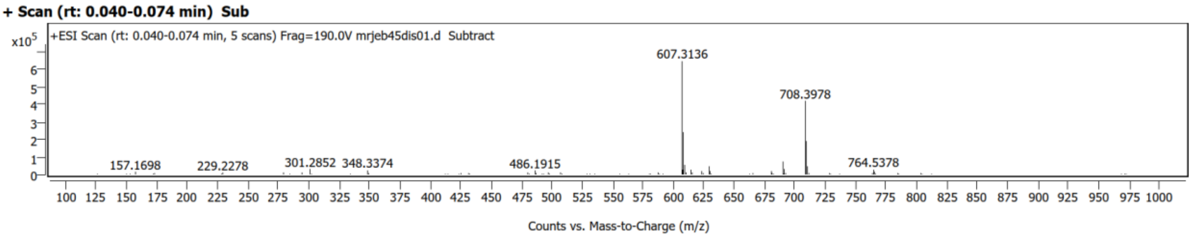

1-(Tert-butylamino)-1-oxopentan-2-yl 2-(2-(4-chlorophenyl)-3-(1,3-dimethyl-2,4,6-trioxohexahydropyrimidin-5-yl)-6,6-dimethyl-4-oxo-4,5,6,7-tetrahydro-1H-indol-1-yl)acetate

(12c)

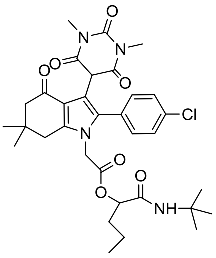

12c(68%)
^{1}H NMR-400MHz
DMSO-d_6

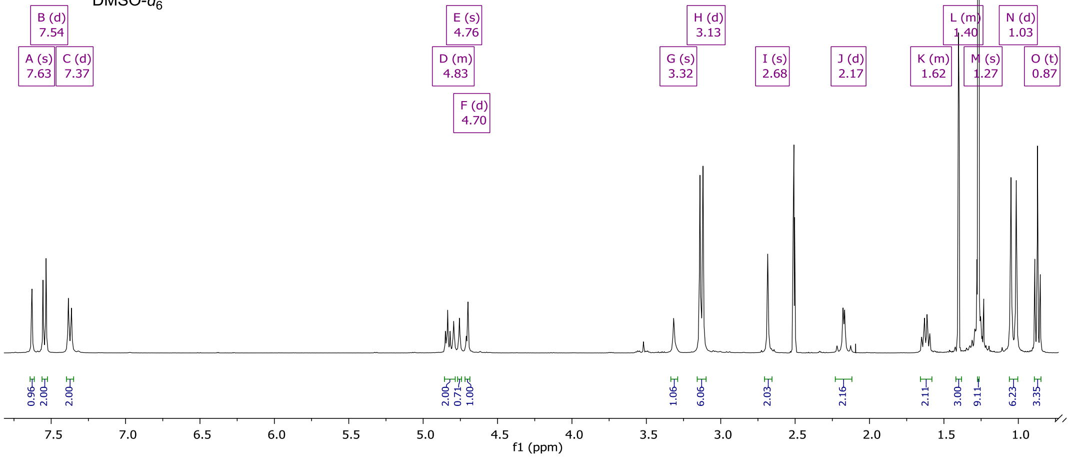

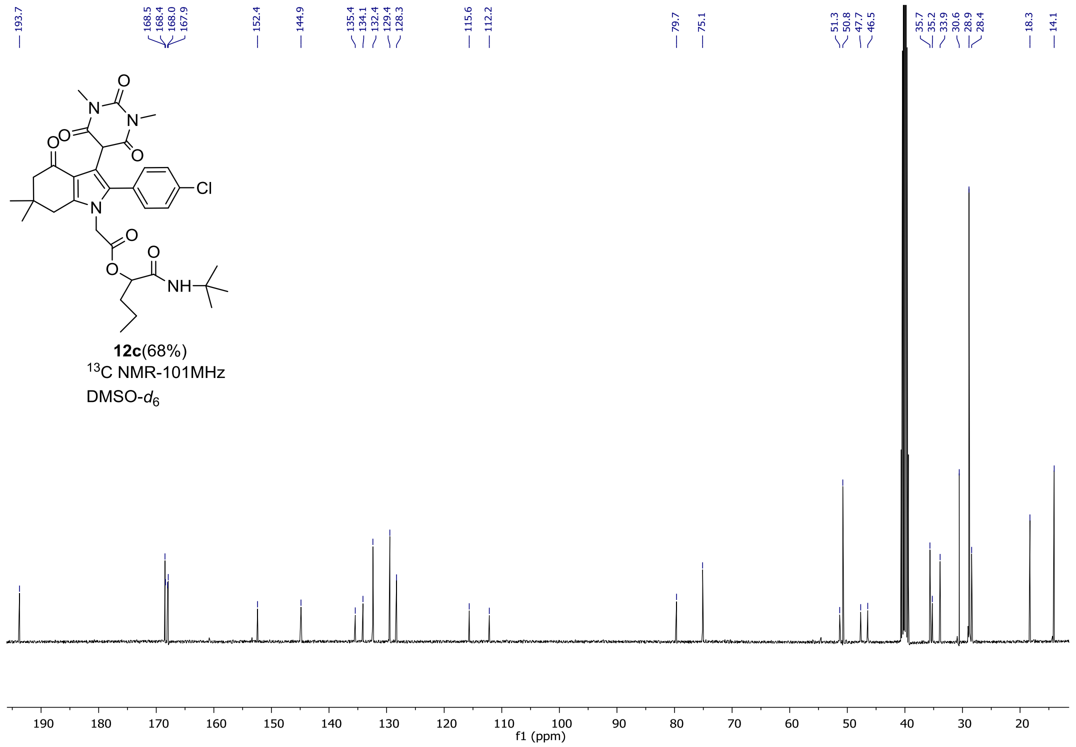
12c(68%)
13C NMR-101MHz
DMSO-d6
193.7
168.5
168.4
168.0
167.9
152.4
144.9
135.4
134.1
132.4
129.4
128.3
115.6
112.2
79.7
75.1
51.3
50.8
47.7
46.5
35.7
35.2
33.9
30.6
28.9
28.4
18.3
14.1
f1 (ppm)

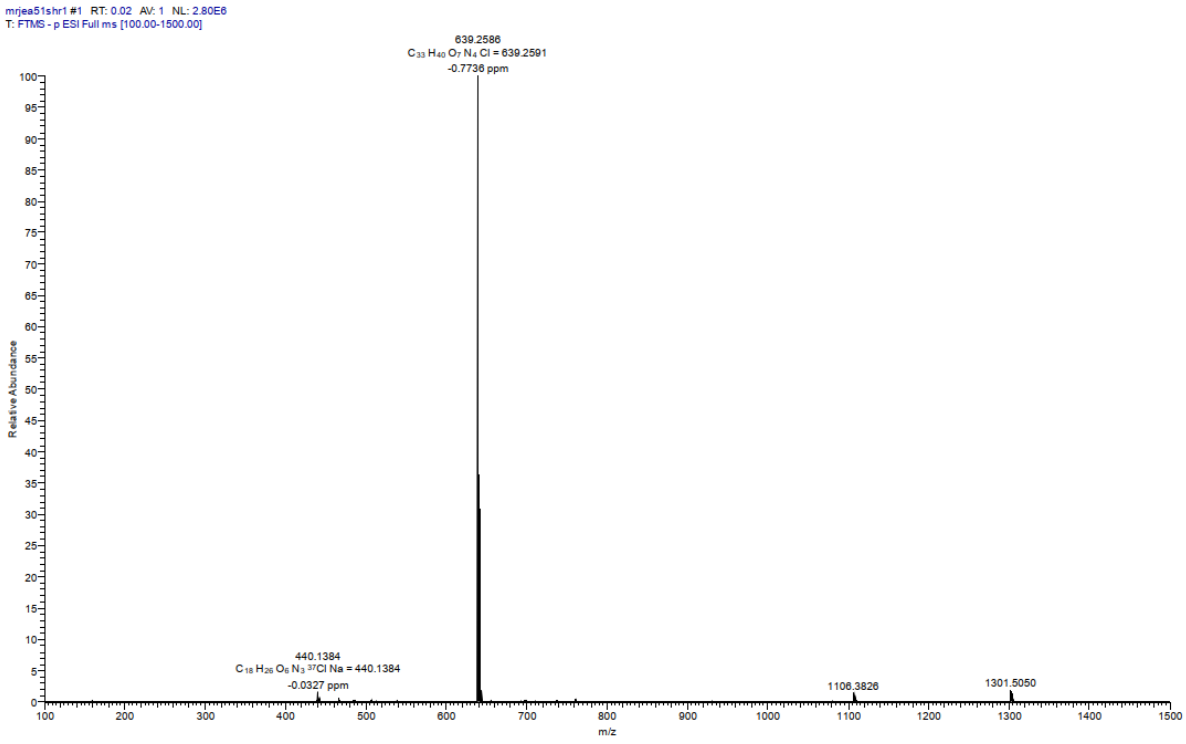
mrjea51shr1 #1 RT: 0.02 AV: 1 NL: 2.80E6
T: FTMS - p ESI Full ms [100.00-1500.00]
639.2586
C33 H40 O7 N4 Cl = 639.2591
-0.7736 ppm
440.1384
C18 H26 O6 N3 37Cl Na = 440.1384
-0.0327 ppm
1106.3826
1301.5050
Relative Abundance
m/z

2-(1-(2-ethoxy-2-oxoethyl)-6,6-dimethyl-4-oxo-2-phenyl-4,5,6,7-tetrahydro-1H-indol-3-yl)acetic acid (**15a**)

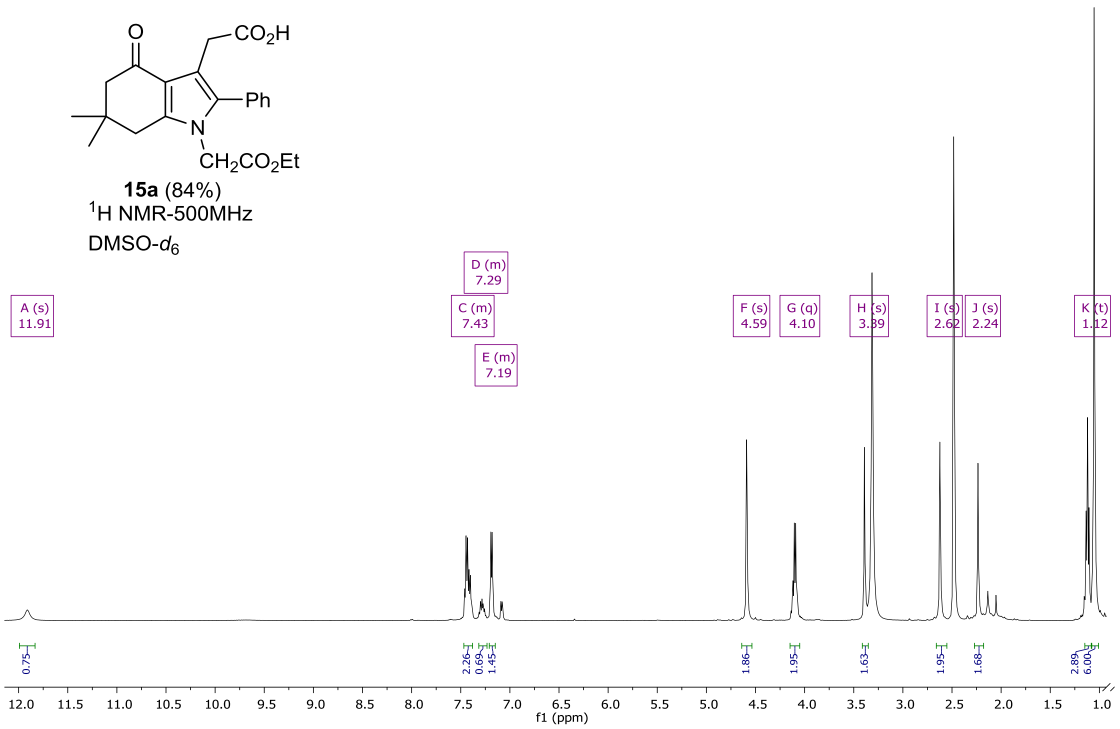

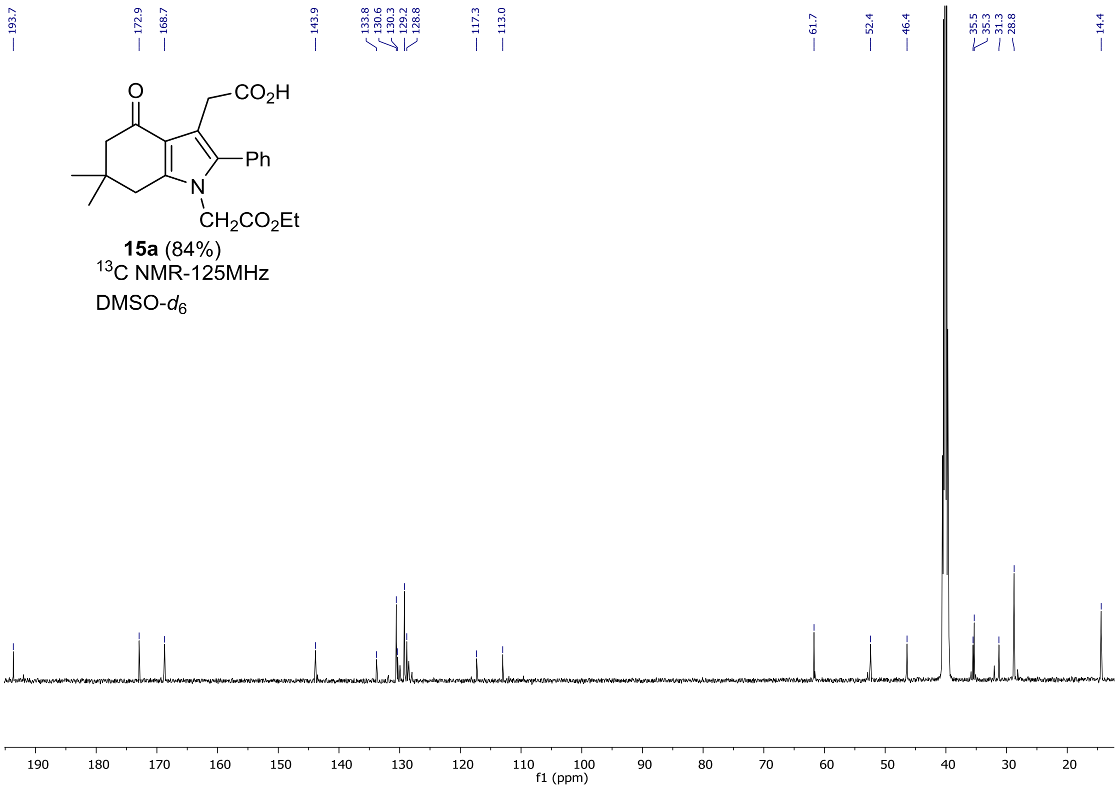

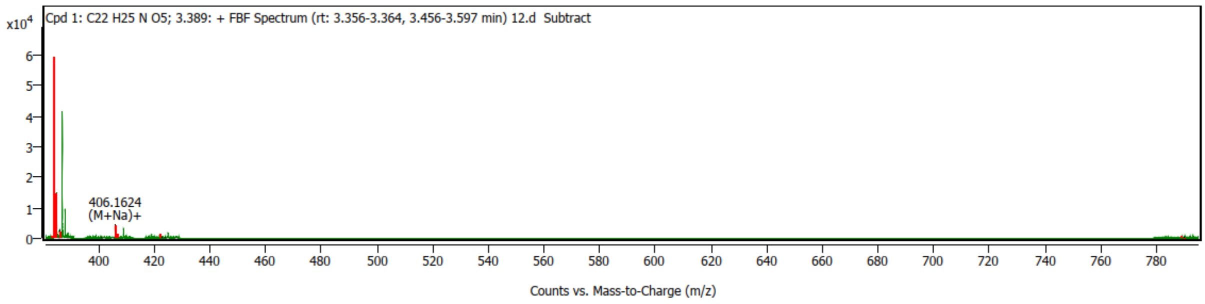

2-(2-(4-Bromophenyl)-1-(2-ethoxy-2-oxoethyl)-6,6-dimethyl-4-oxo-4,5,6,7-tetrahydro-1H-indol-3-yl)acetic acid (**15b**)

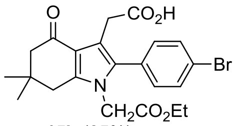

15b (25%)
^{1}H NMR-400MHz
$CDCl_3$

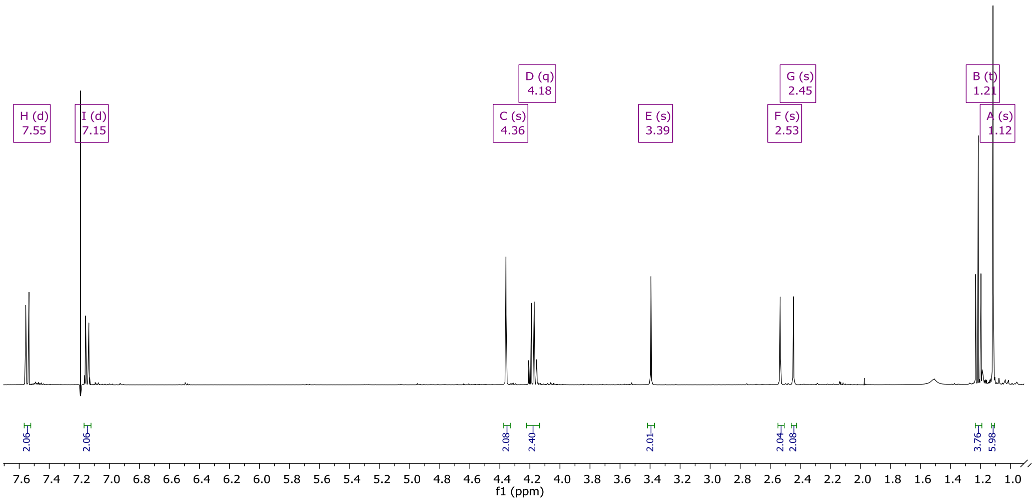

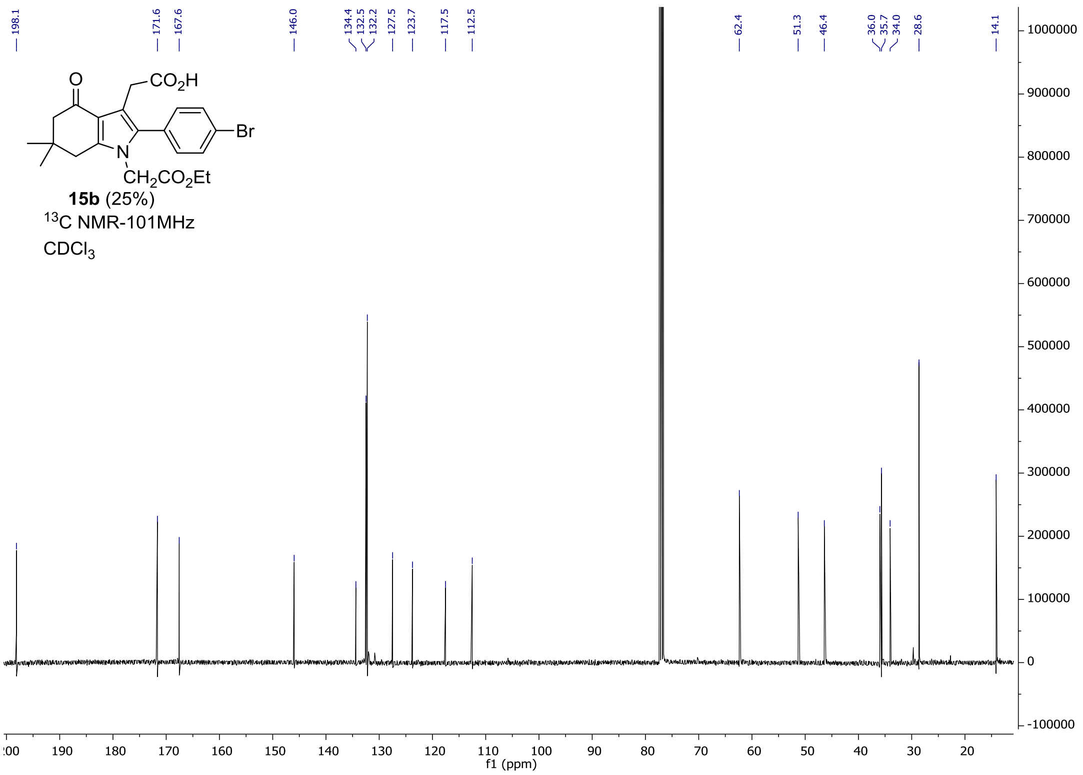
198.1
171.6
167.6
146.0
134.4
132.5
132.2
127.5
123.7
117.5
112.5
62.4
51.3
46.4
36.0
35.7
34.0
28.6
14.1
CO_2H
Br
N
CH_2CO_2Et
15b (25%)
^{13}C NMR-101MHz
$CDCl_3$
f1 (ppm)

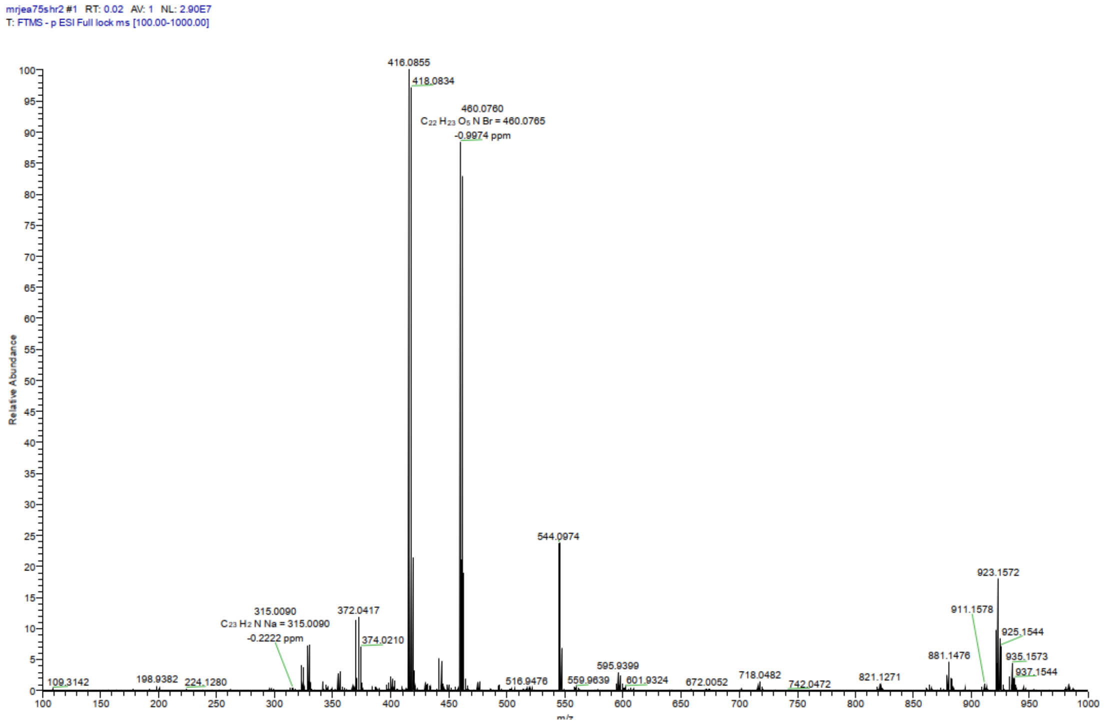
mrjea75shr2 #1 RT: 0.02 AV: 1 NL: 2.90E7
T: FTMS - p ESI Full lock ms [100.00-1000.00]
416.0855
418.0834
460.0760
$C_{22}H_{23}O_5NBr$ = 460.0765
-0.9974 ppm
544.0974
923.1572
911.1578
925.1544
935.1573
937.1544
881.1476
821.1271
742.0472
718.0482
672.0052
601.9324
595.9399
559.9639
516.9476
374.0210
372.0417
315.0090
$C_{23}H_2NNa$ = 315.0090
-0.2222 ppm
224.1280
198.9382
109.3142
Relative Abundance
m/z

2-(1-(2-Ethoxy-2-oxoethyl)-6,6-dimethyl-2-(4-nitrophenyl)-4-oxo-4,5,6,7-tetrahydro-1H-indol-3-yl)acetic acid (**15c**)

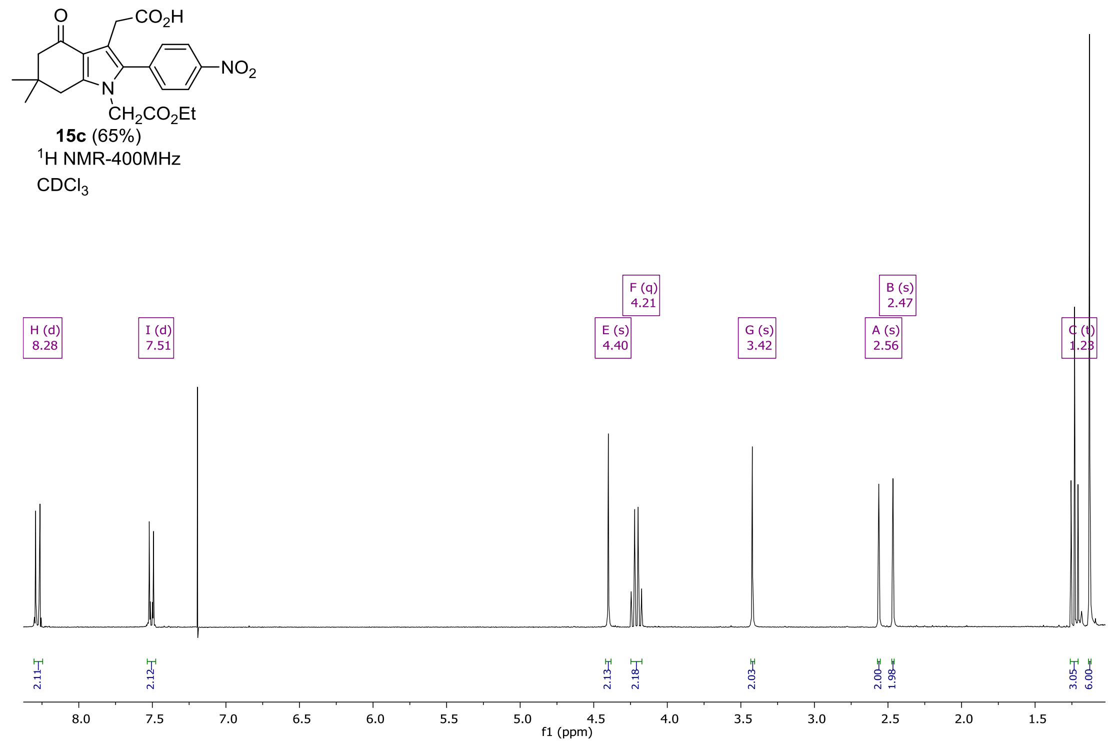

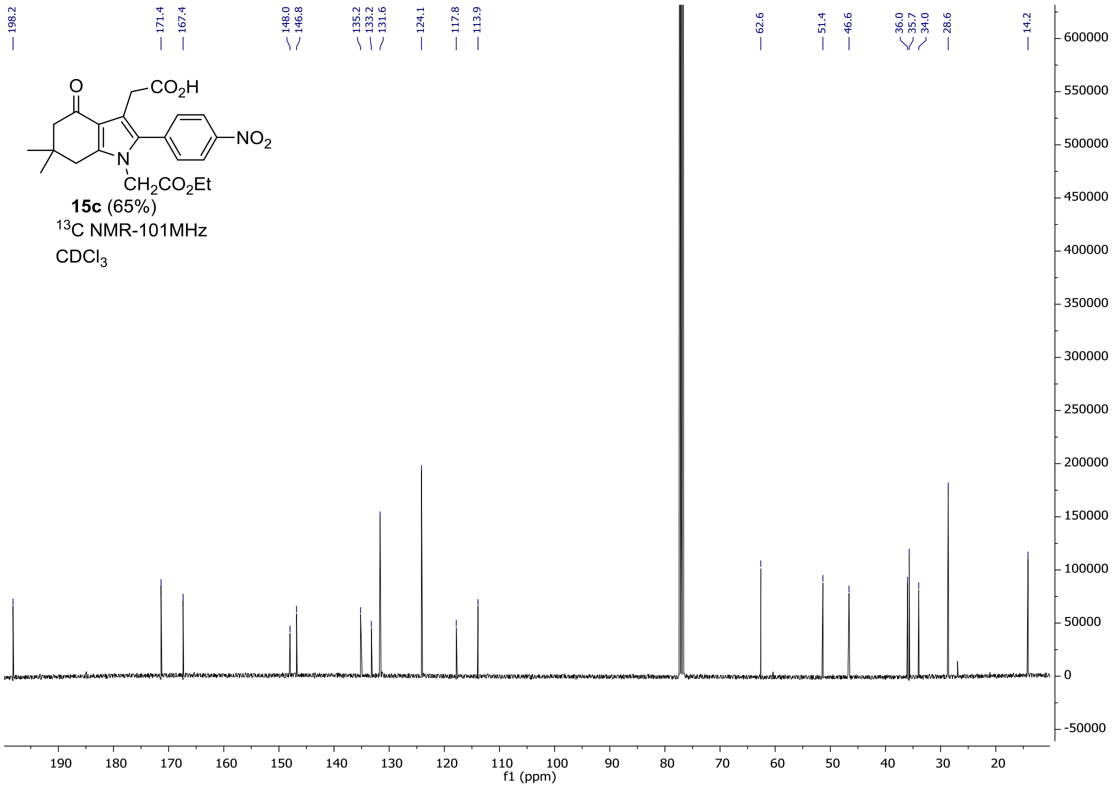

Sample Spectra

+ Scan (rt: -0.001-0.125 min) Sub

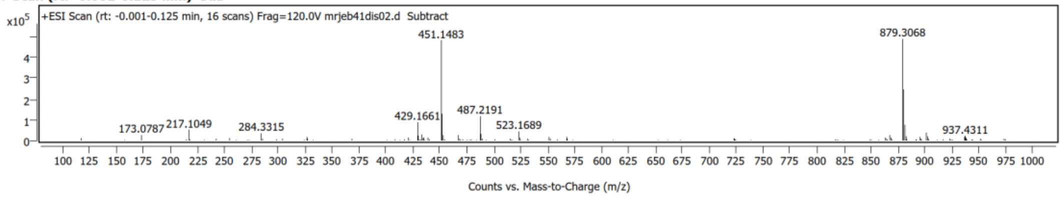

2-(2-(4-Bromophenyl)-1-(2-ethoxy-2-oxoethyl)-4-oxo-4,5,6,7-tetrahydro-1H-indol-3-yl)acetic acid (**15d**)

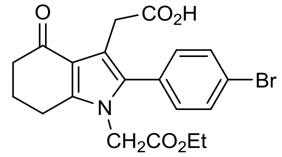

15d (83%)

^{1}H NMR-500MHz

DMSO-d_6

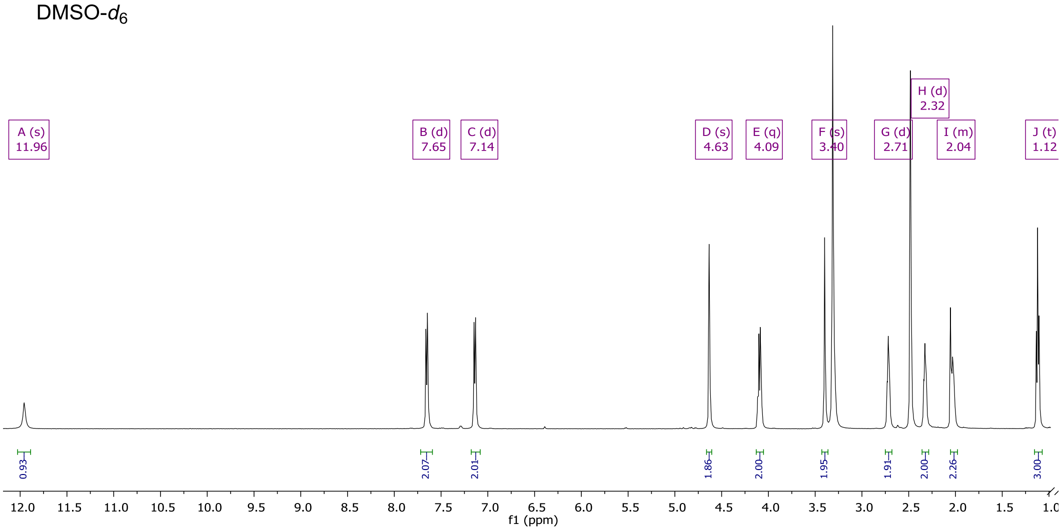

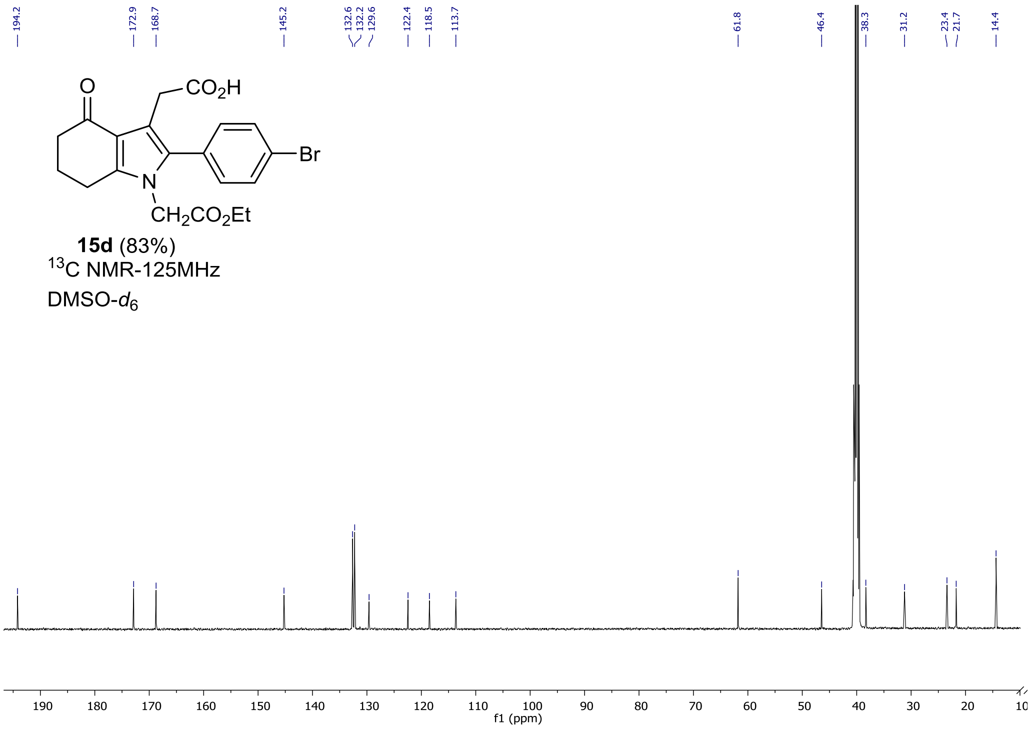
194.2
172.9
168.7
145.2
132.6
132.2
129.6
122.4
118.5
113.7
61.8
46.4
38.3
31.2
23.4
21.7
14.4
CO2H
Br
O
N
CH2CO2Et
15d (83%)
13C NMR-125MHz
DMSO-d6
190 180 170 160 150 140 130 120 110 100 90 80 70 60 50 40 30 20 10
f1 (ppm)

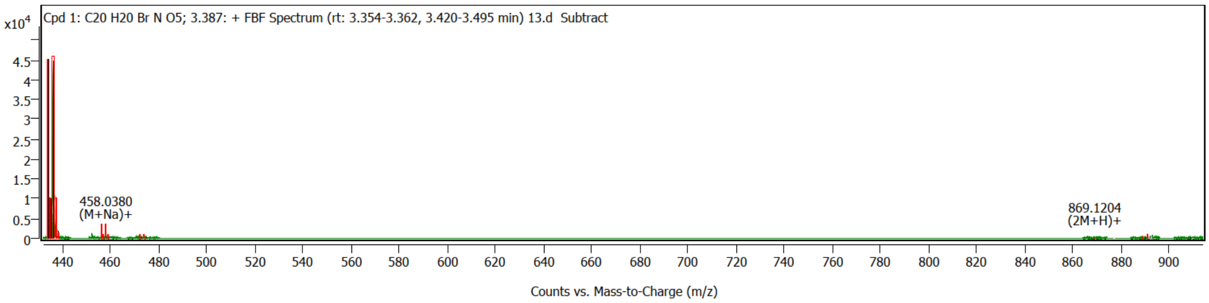
Cpd 1: C20 H20 Br N O5; 3.387: + FBF Spectrum (rt: 3.354-3.362, 3.420-3.495 min) 13.d Subtract
x10^4
458.0380
(M+Na)+
869.1204
(2M+H)+
Counts vs. Mass-to-Charge (m/z)

2-(1-(2-Ethoxy-2-oxoethyl)-4-oxo-2-phenyl-4,5,6,7-tetrahydro-1H-indol-3-yl)acetic acid (**15e**)

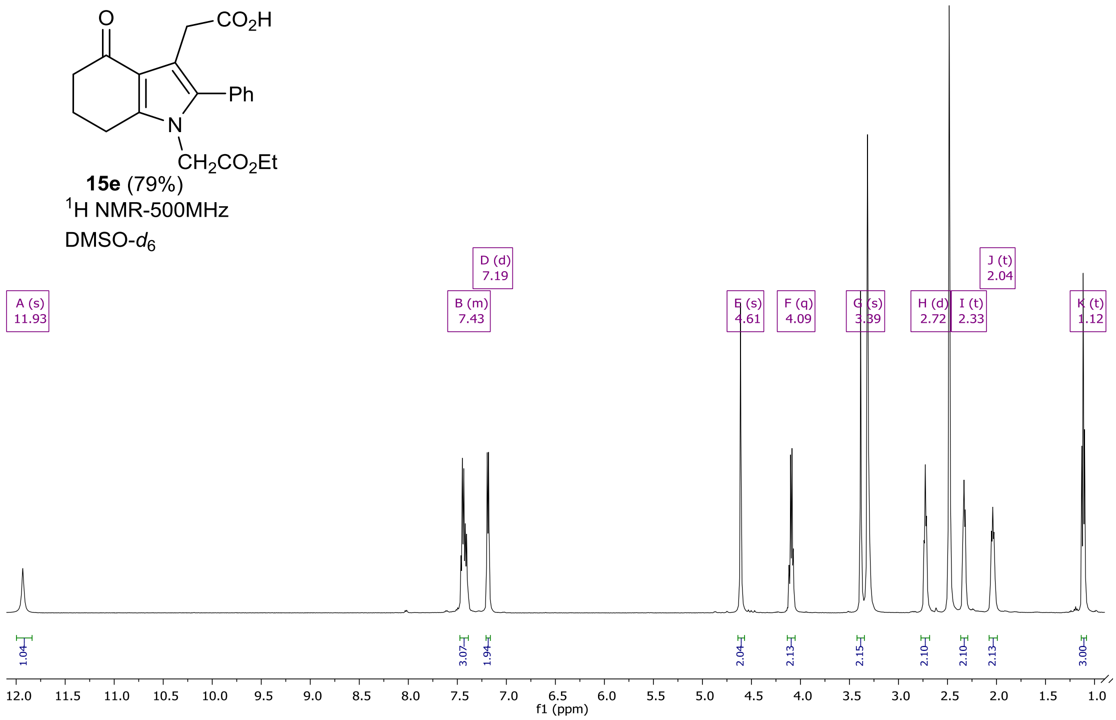

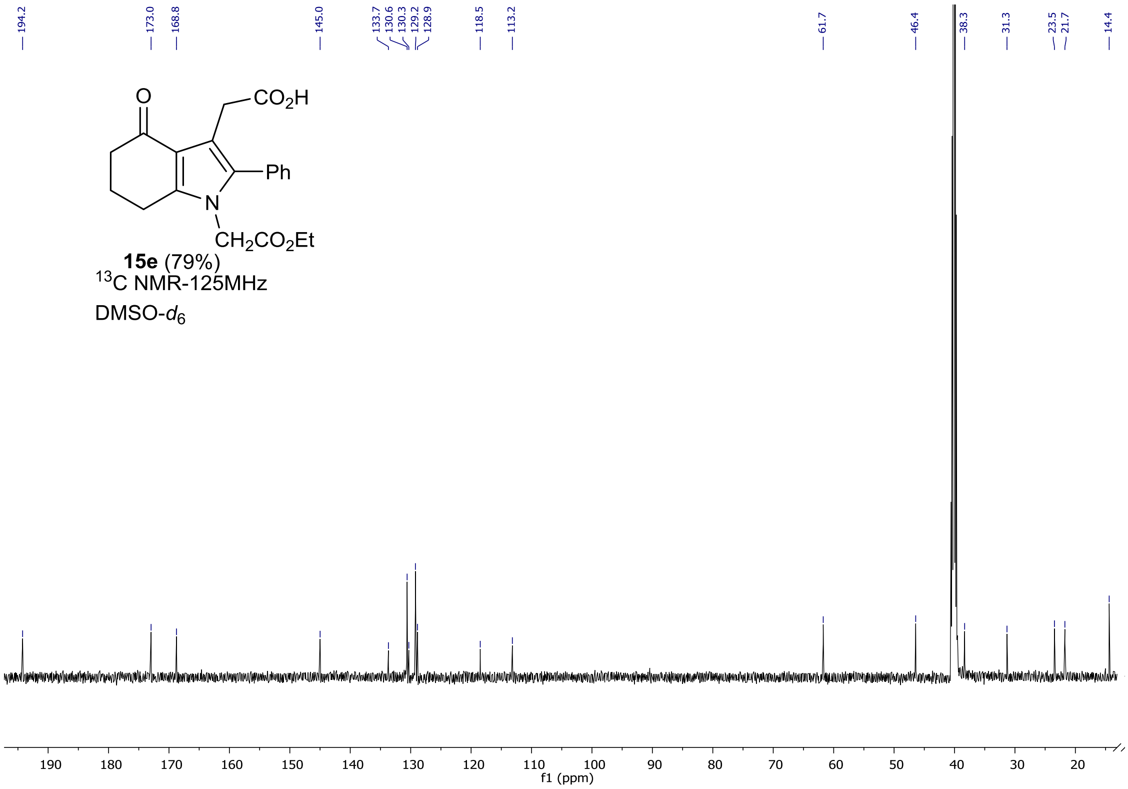

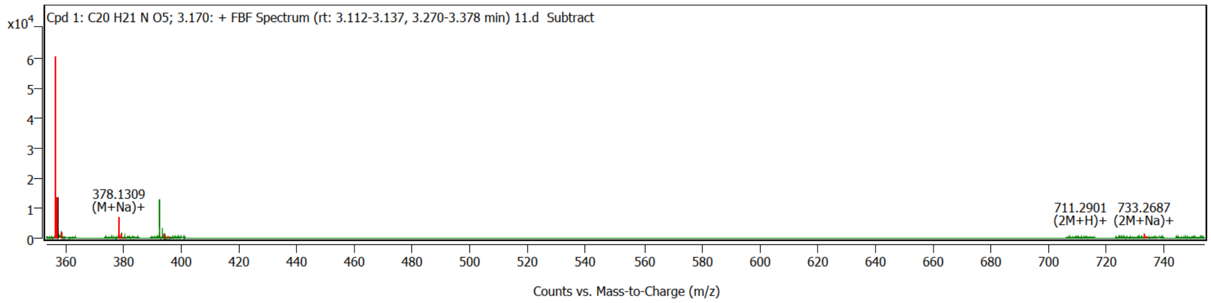

Diethyl 2,2'-(6,6-dimethyl-4-oxo-2-phenyl-4,5,6,7-tetrahydro-1H-indole-1,3-diyl)diacetate

(16a)

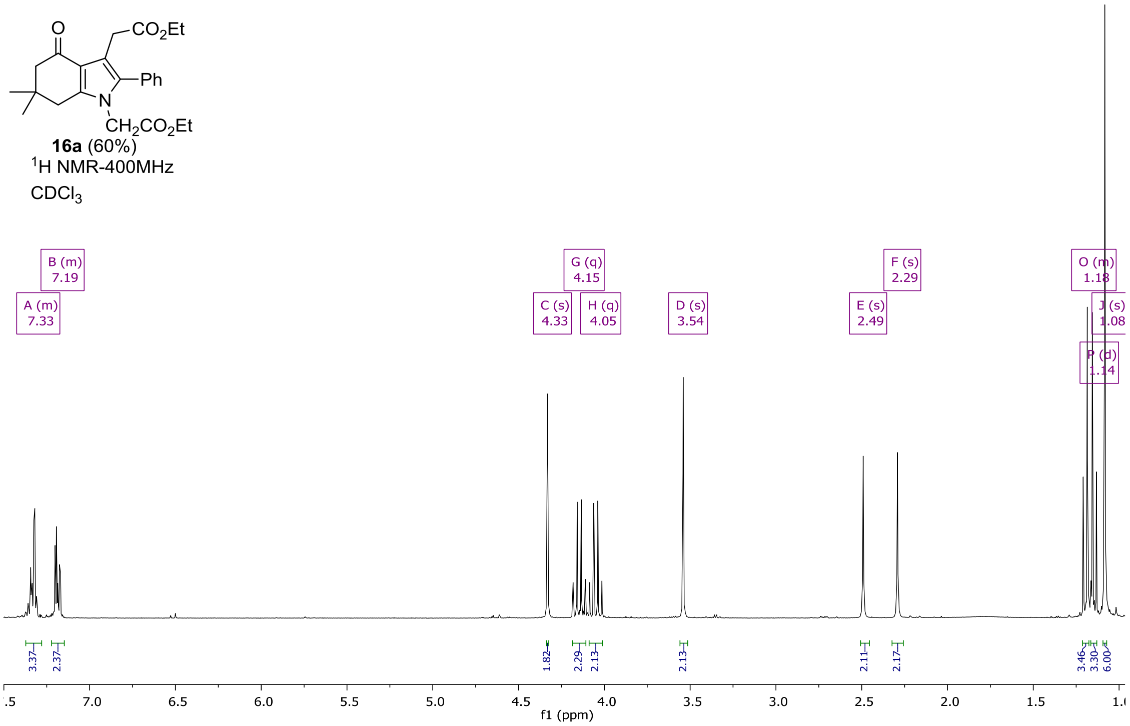

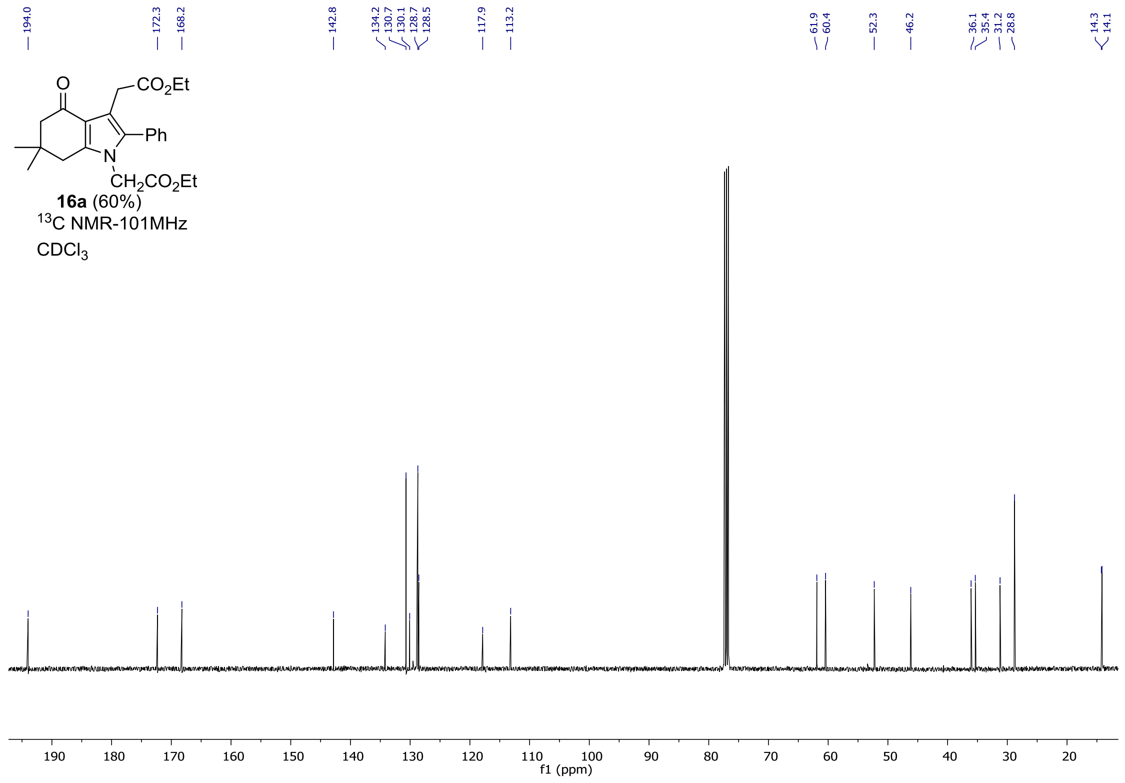
194.0
172.3
168.2
142.8
134.2
130.7
130.1
128.7
128.5
117.9
113.2
61.9
60.4
52.3
46.2
36.1
35.4
31.2
28.8
14.3
14.1
O
CO_2Et
Ph
N
CH_2CO_2Et
16a (60%)
^{13}C NMR-101MHz
$CDCl_3$
f1 (ppm)

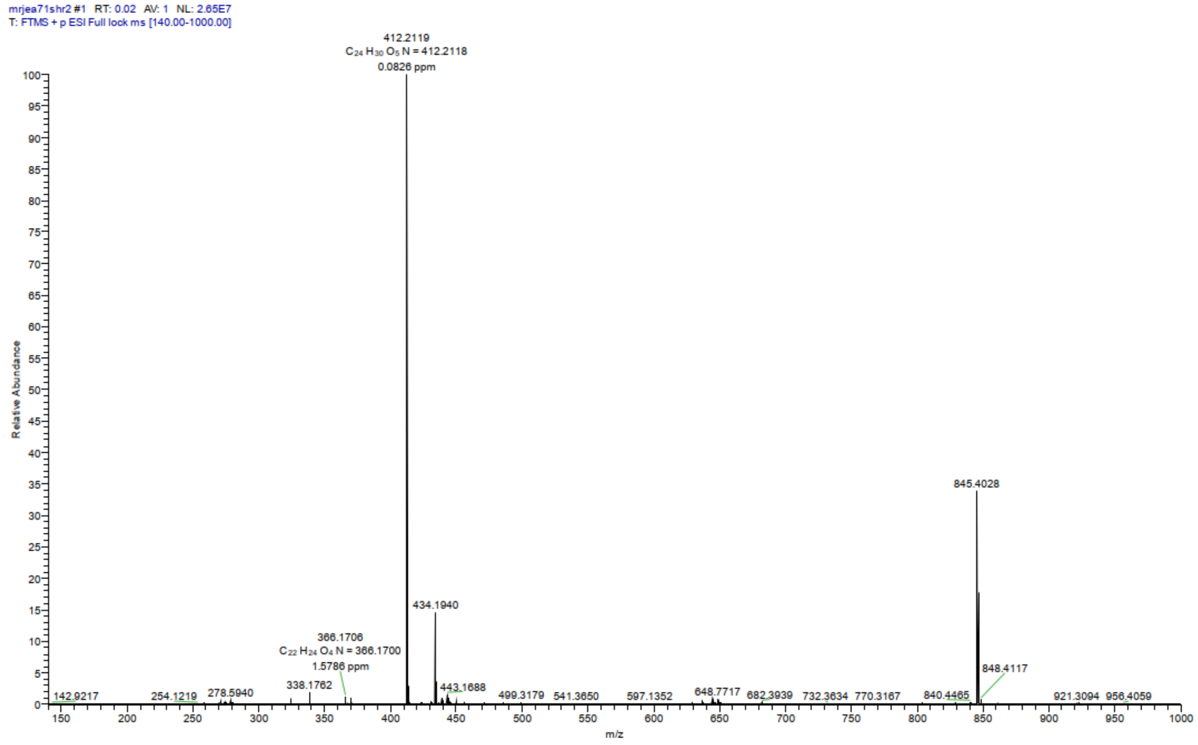
mrjea71shr2 #1 RT: 0.02 AV: 1 NL: 2.65E7
T: FTMS + p ESI Full lock ms [140.00-1000.00]
412.2119
$C_{24}H_{30}O_5N$ = 412.2118
0.0826 ppm
845.4028
434.1940
366.1706
$C_{22}H_{24}O_4N$ = 366.1700
1.5786 ppm
848.4117
338.1762
443.1688
Relative Abundance
m/z

Diethyl 2,2'-(2-(4-bromophenyl)-6,6-dimethyl-4-oxo-4,5,6,7-tetrahydro-1H-indole-1,3-diyl)diacetate (**16b**)

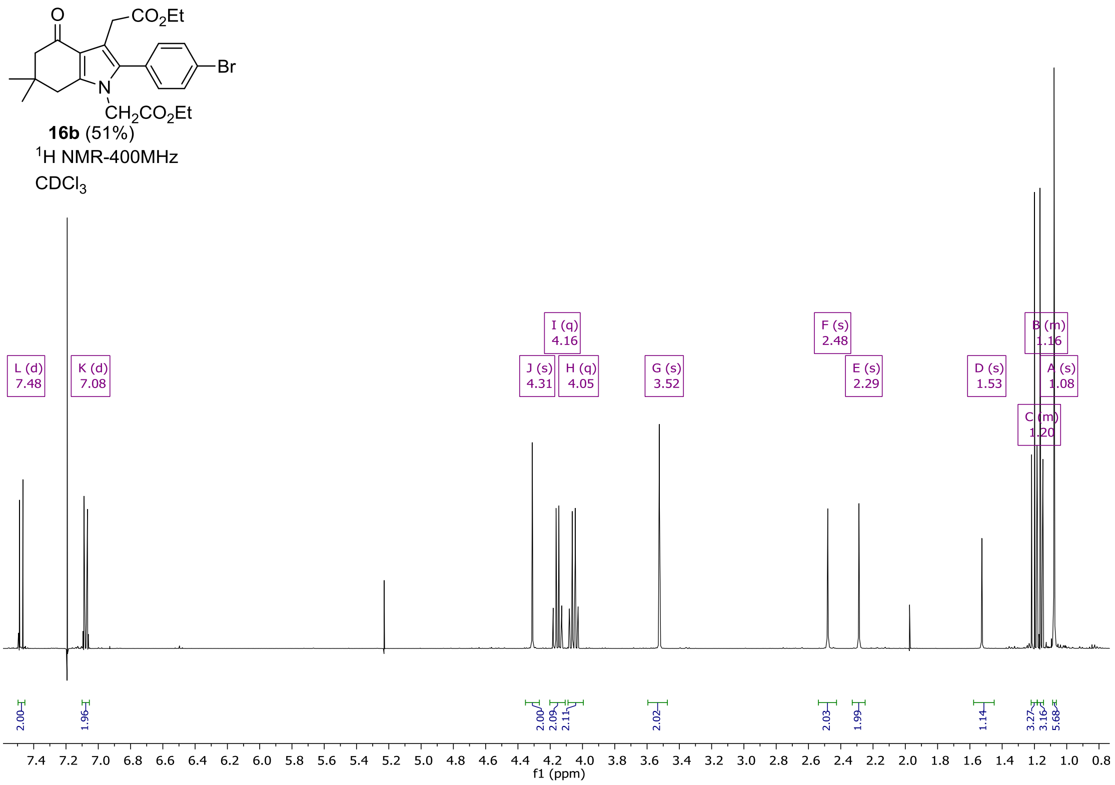

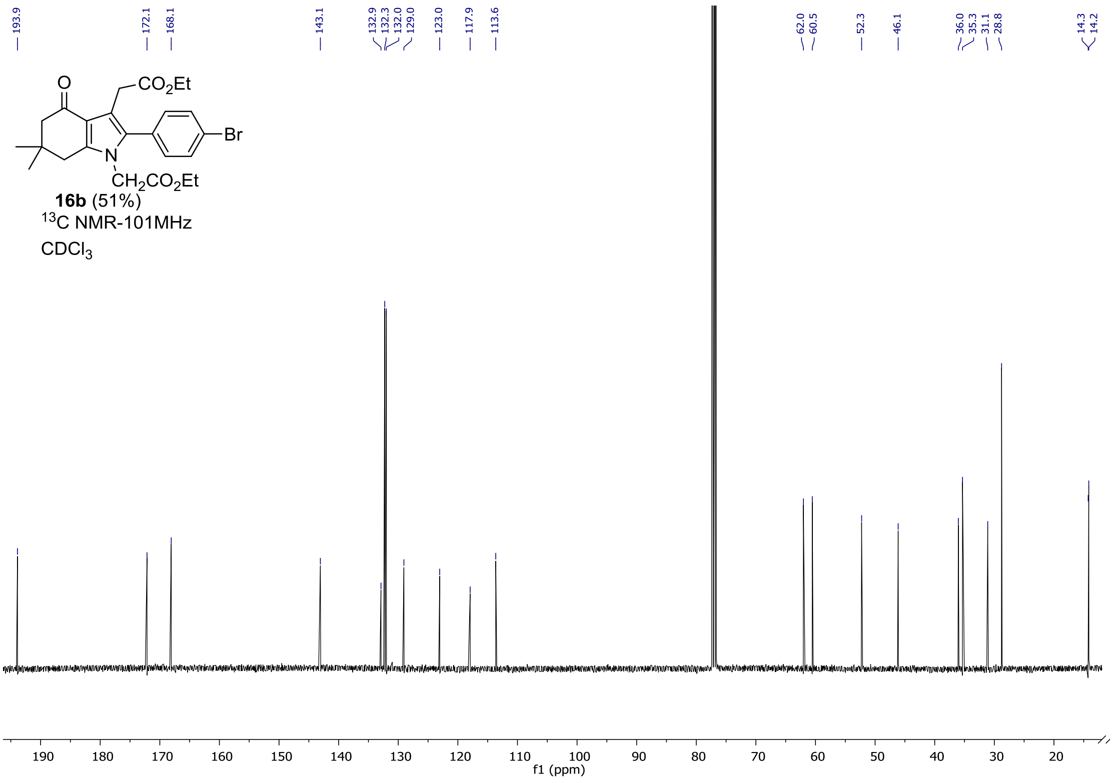

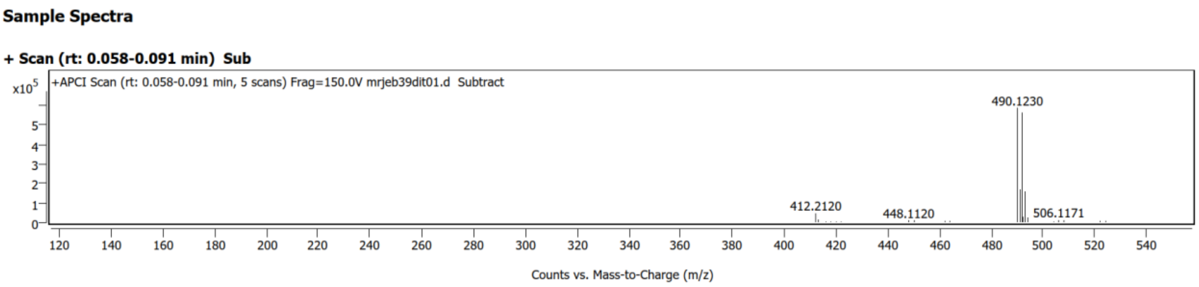

Diethyl 2,2'-(2-(4-chlorophenyl)-6,6-dimethyl-4-oxo-4,5,6,7-tetrahydro-1H-indole-1,3-diyl)diacetate (**16c**)

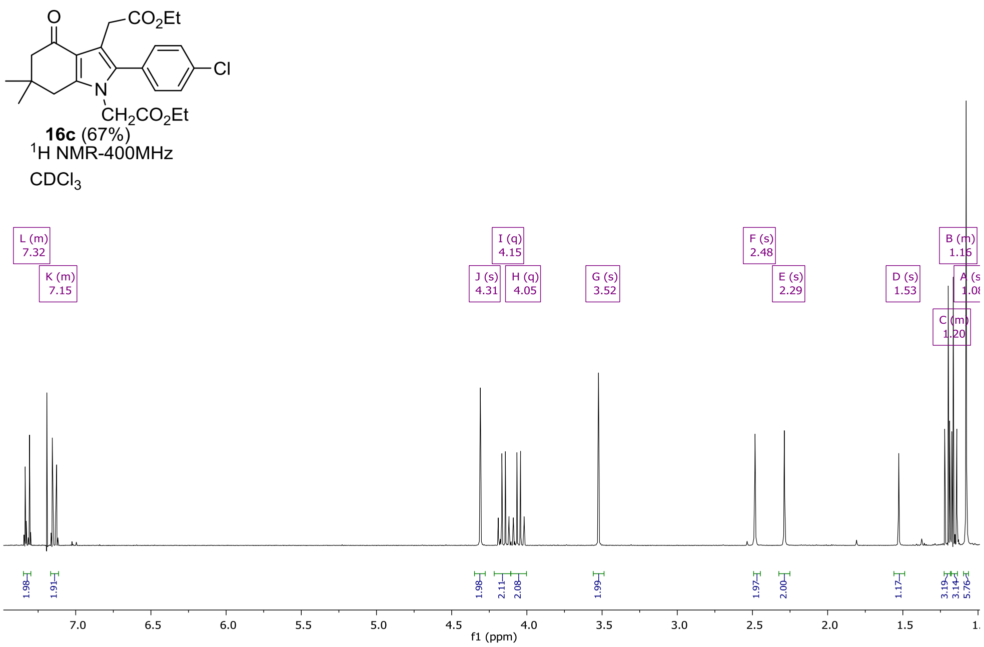

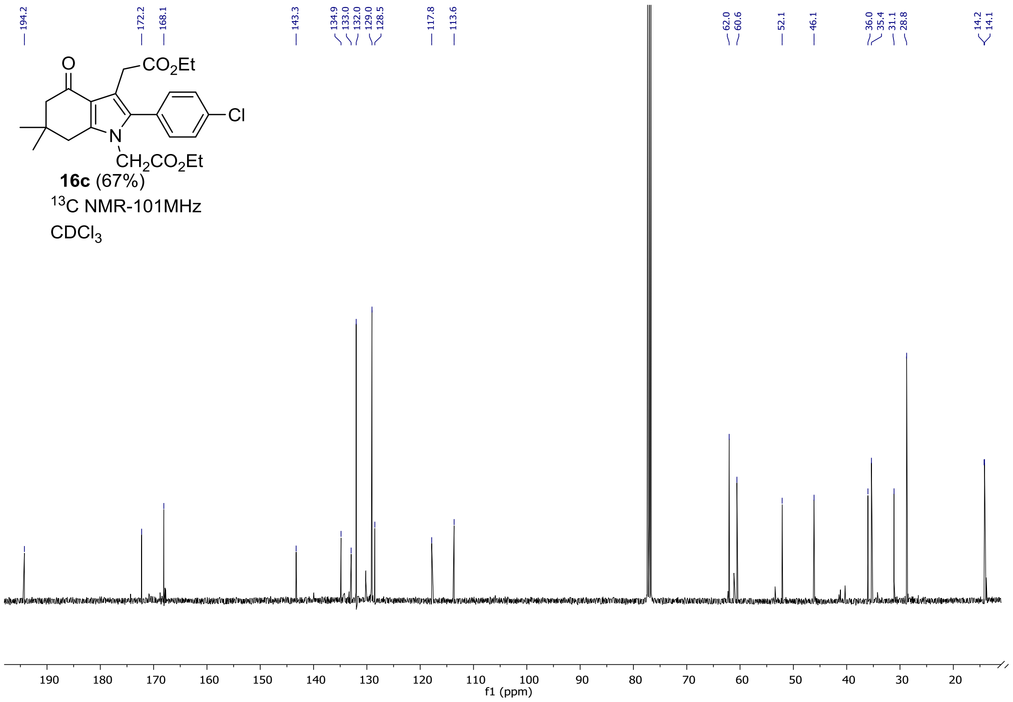

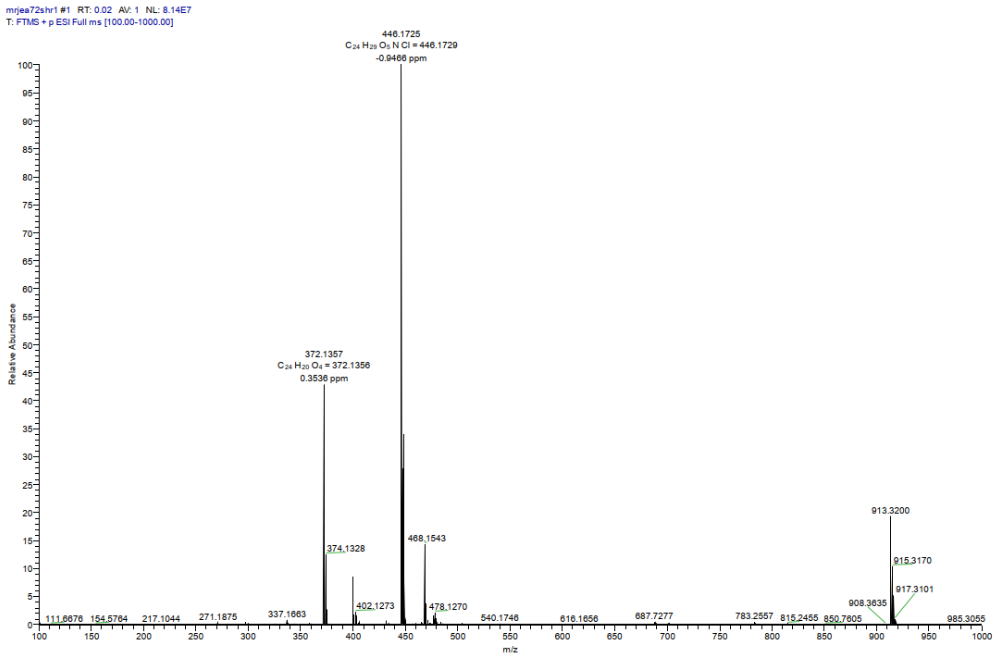

2-(2-(4-chlorophenyl)-7-ethoxy-6,6-dimethyl-4-oxo-4,5,6,7-tetrahydro-1H-indol-1-yl)acetic acid (**17d**)

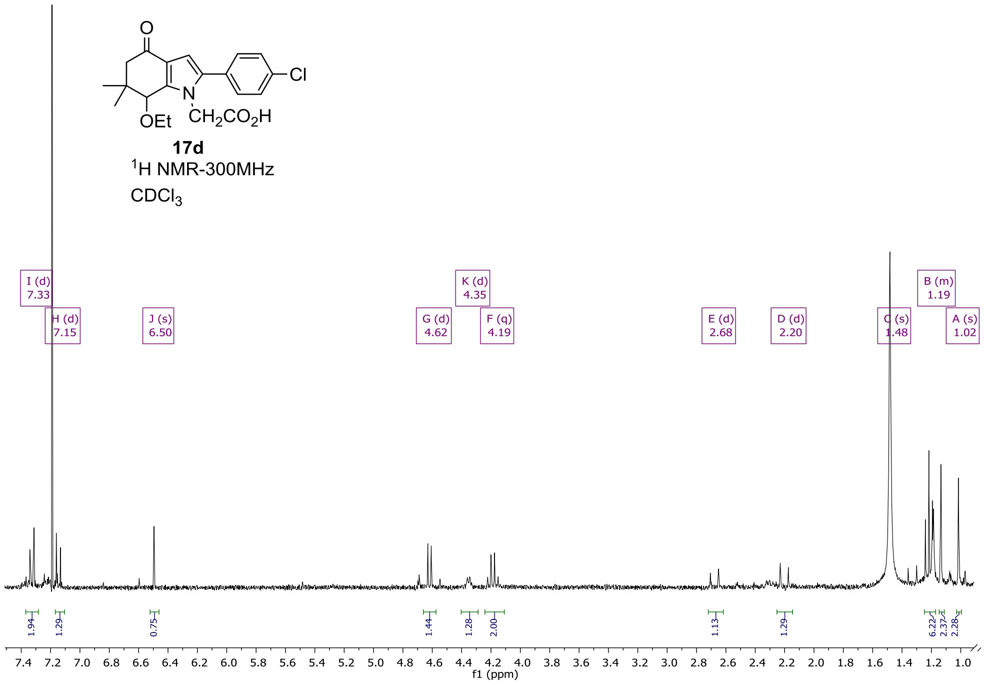

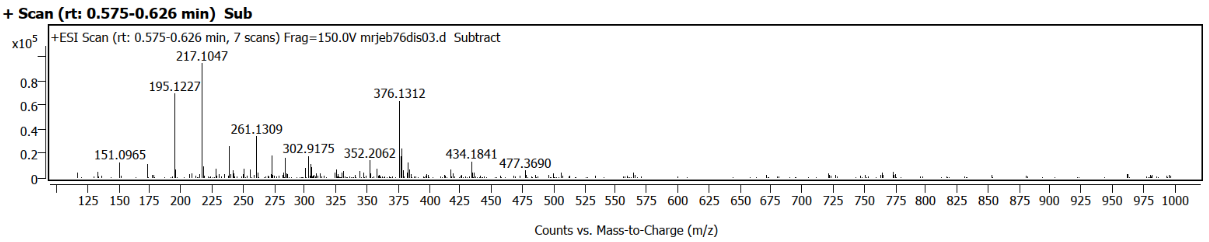

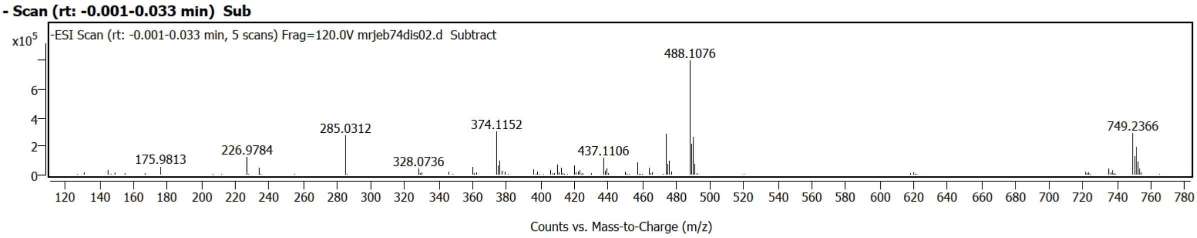

Ethyl 2-(6-(4-chlorophenyl)-1,3-dimethyl-2,4-dioxo-2,3,4,7-tetrahydro-1H-pyrrolo[2,3-d]pyrimidin-5-yl)acetate (**19a**)

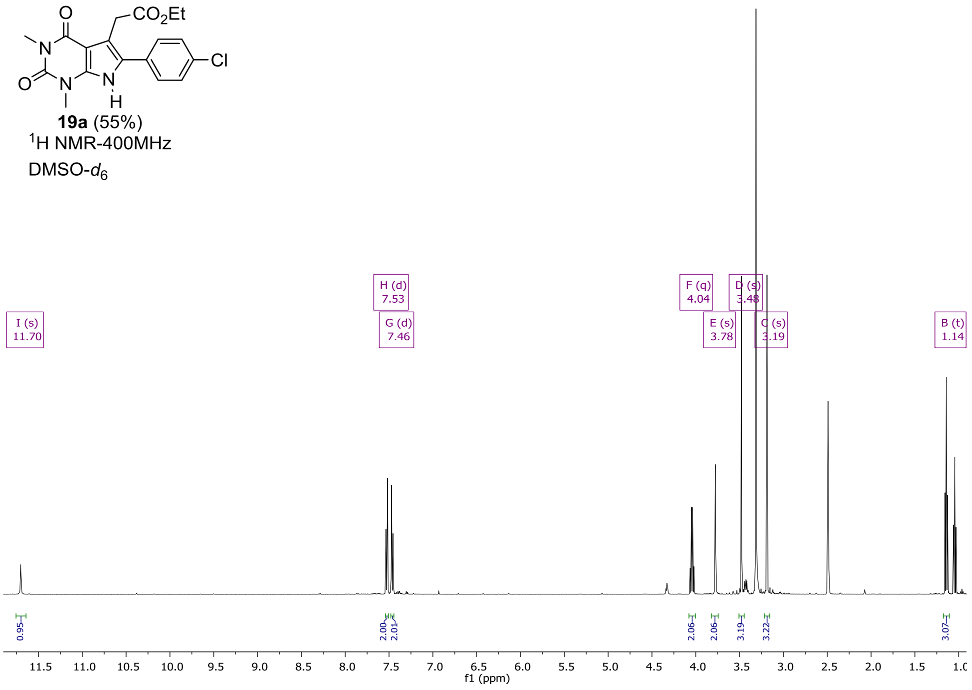

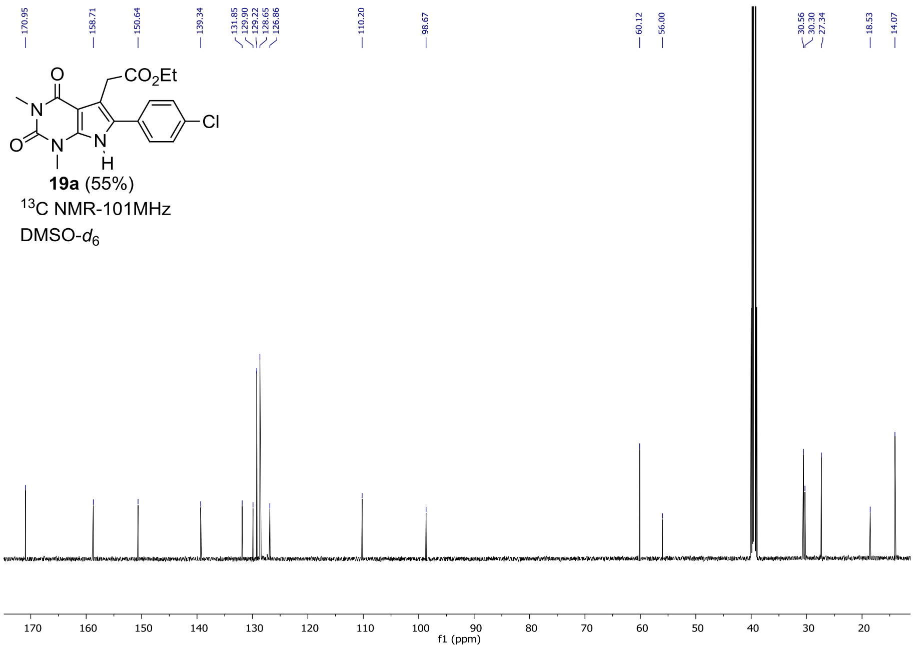

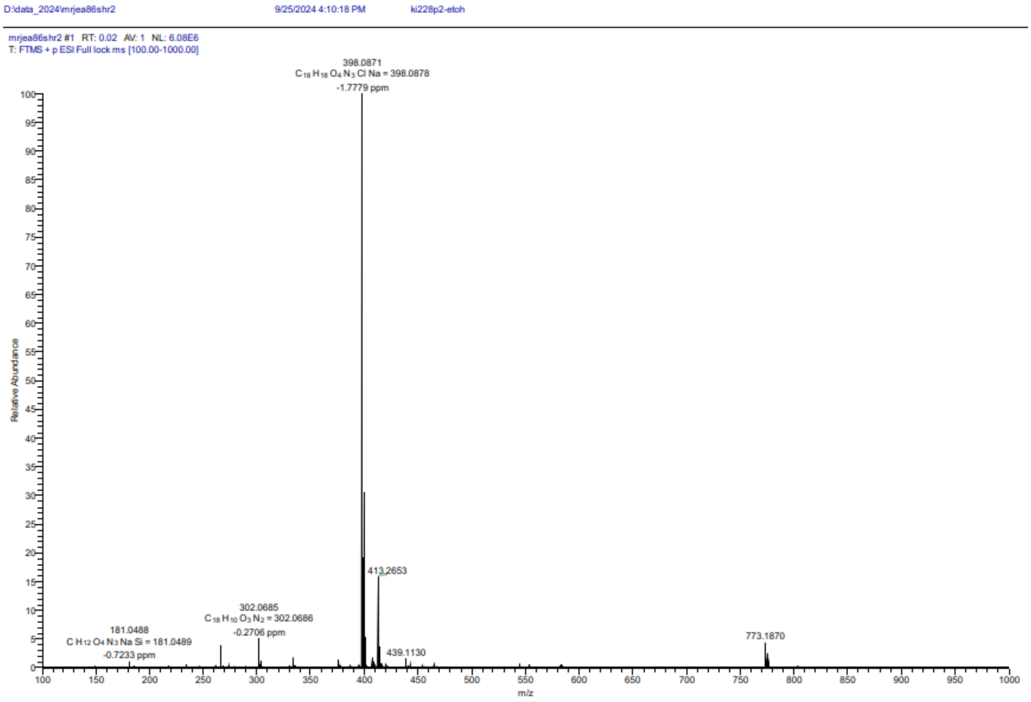

Isopropyl 2-(6-(4-chlorophenyl)-1,3-dimethyl-2,4-dioxo-2,3,4,7-tetrahydro-1H-pyrrolo[2,3-d]pyrimidin-5-yl)acetate (**19b**)

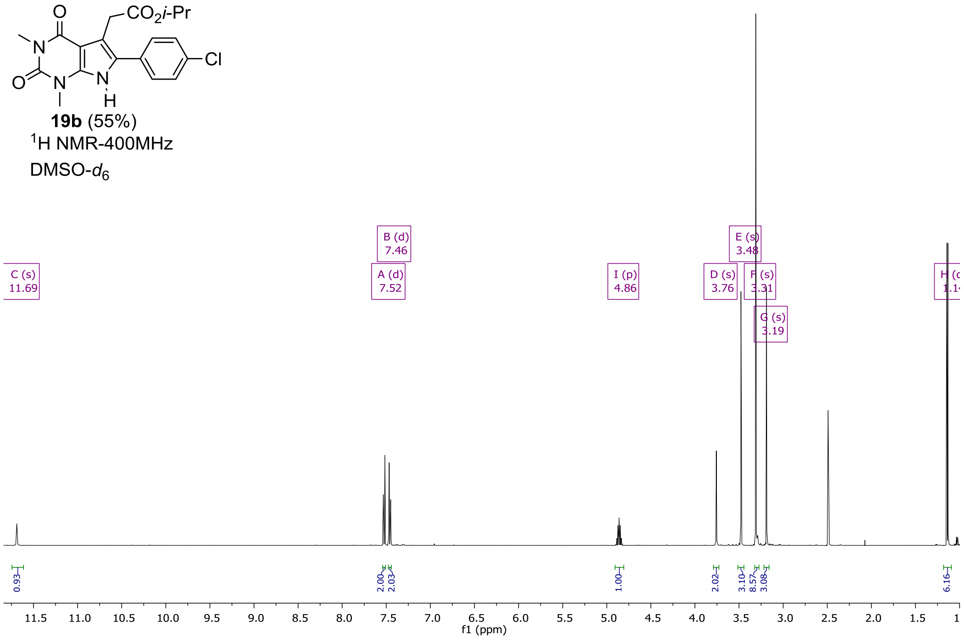

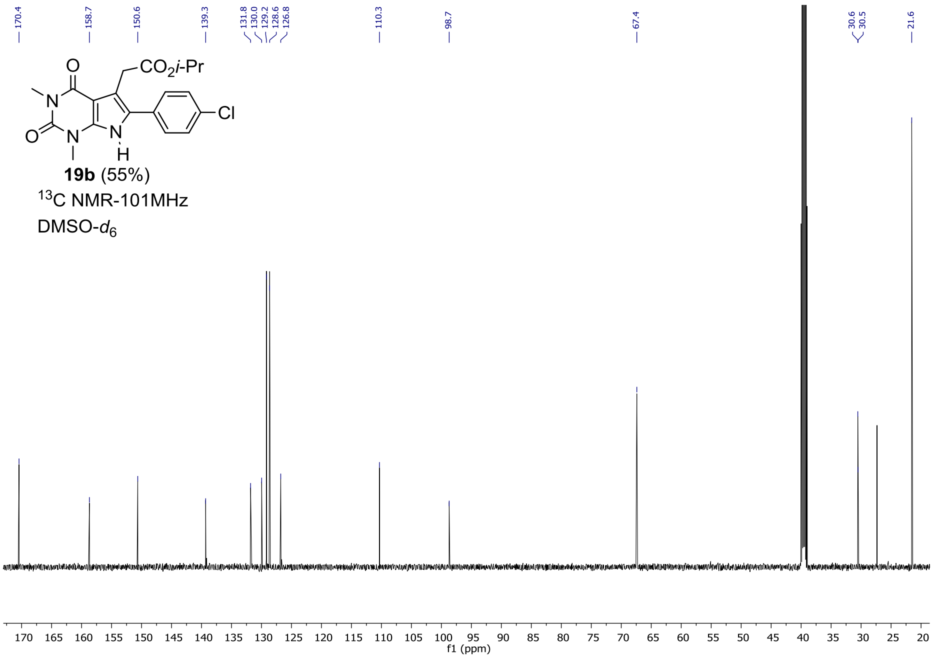
170.4
158.7
150.6
139.3
131.8
130.0
129.2
128.6
126.8
110.3
98.7
67.4
30.6
30.5
21.6
CO_2i-Pr
Cl
19b (55%)
^{13}C NMR-101MHz
DMSO-d_6
f1 (ppm)

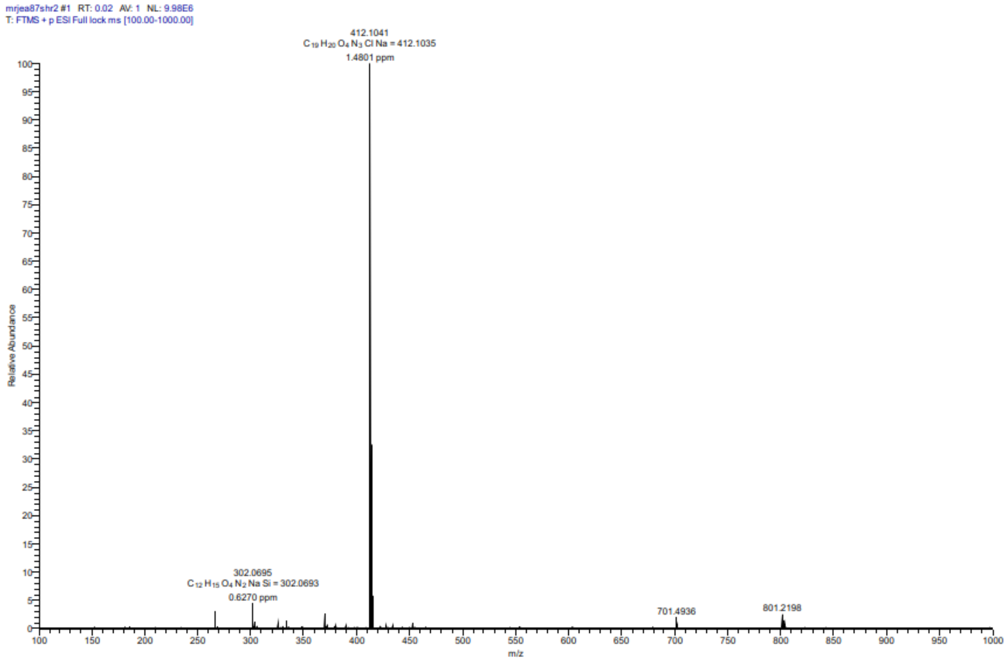
mrjea87shr2 #1 RT: 0.02 AV: 1 NL: 9.98E6
T: FTMS + p ESI Full lock ms [100.00-1000.00]
412.1041
$C_{19}H_{20}O_4N_3ClNa$ = 412.1035
1.4801 ppm
302.0695
$C_{12}H_{15}O_4N_2NaSi$ = 302.0693
0.6270 ppm
701.4936
801.2198
Relative Abundance
m/z

Ethyl 2-(1,3-dimethyl-6-(4-nitrophenyl)-2,4-dioxo-2,3,4,7-tetrahydro-1H-pyrrolo[2,3-d]pyrimidin-5-yl)acetate (**19c**)

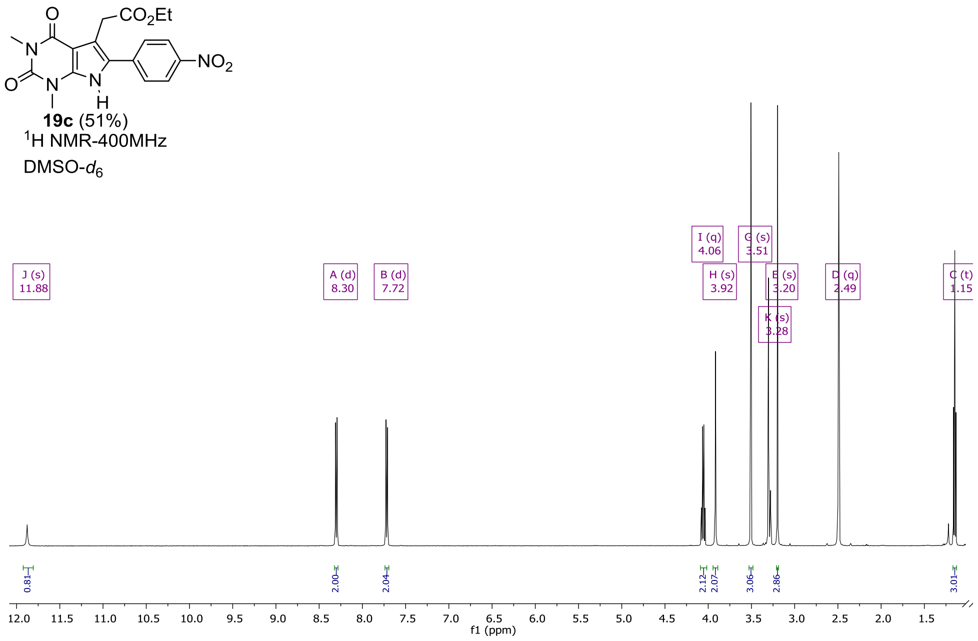

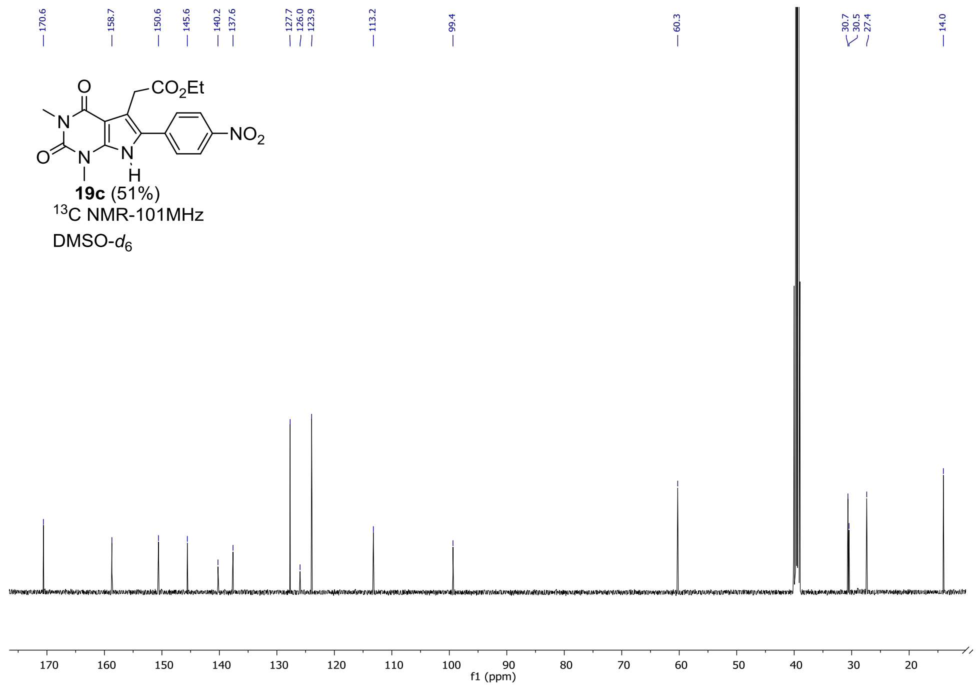

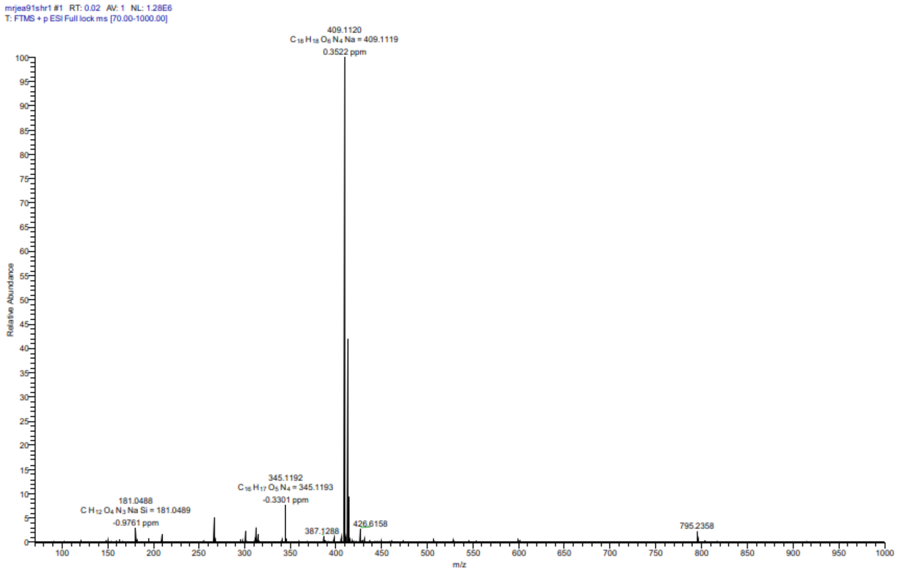

5-(6-Amino-1,3-dimethyl-2,4-dioxo-1,2,3,4-tetrahydropyrimidin-5-yl)-6-(3-chlorophenyl)-1,3-dimethyl-1,7-dihydro-2H-pyrrolo[2,3-d]pyrimidine-2,4(3H)-dione (**20a**)

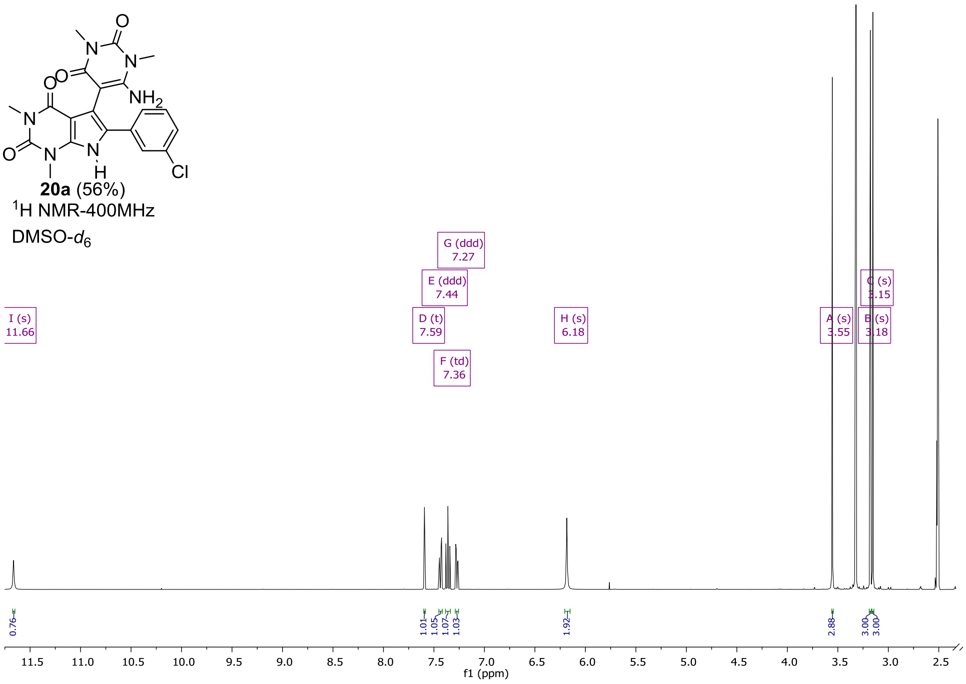

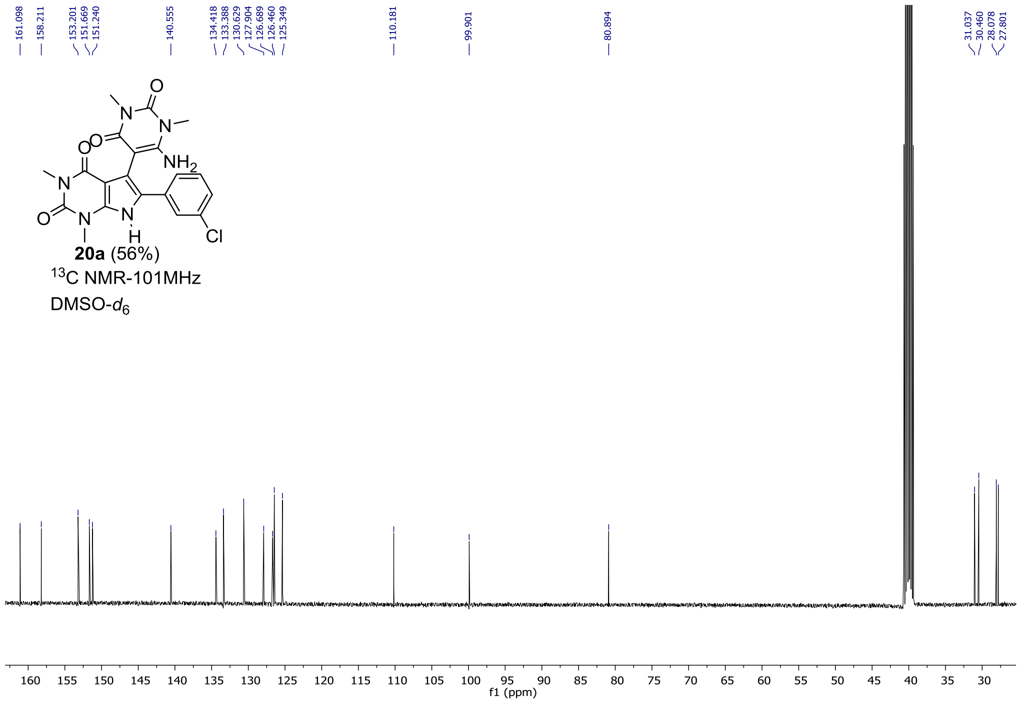

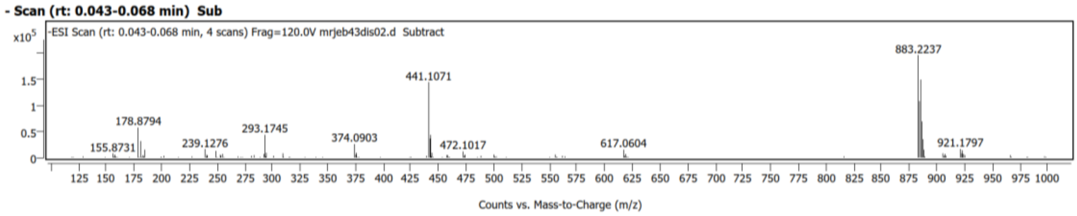

5-(6-Amino-1,3-dimethyl-2,4-dioxo-1,2,3,4-tetrahydropyrimidin-5-yl)-6-(4-fluorophenyl)-1,3-dimethyl-1,7-dihydro-2H-pyrrolo[2,3-d]pyrimidine-2,4(3H)-dione (**20b**)

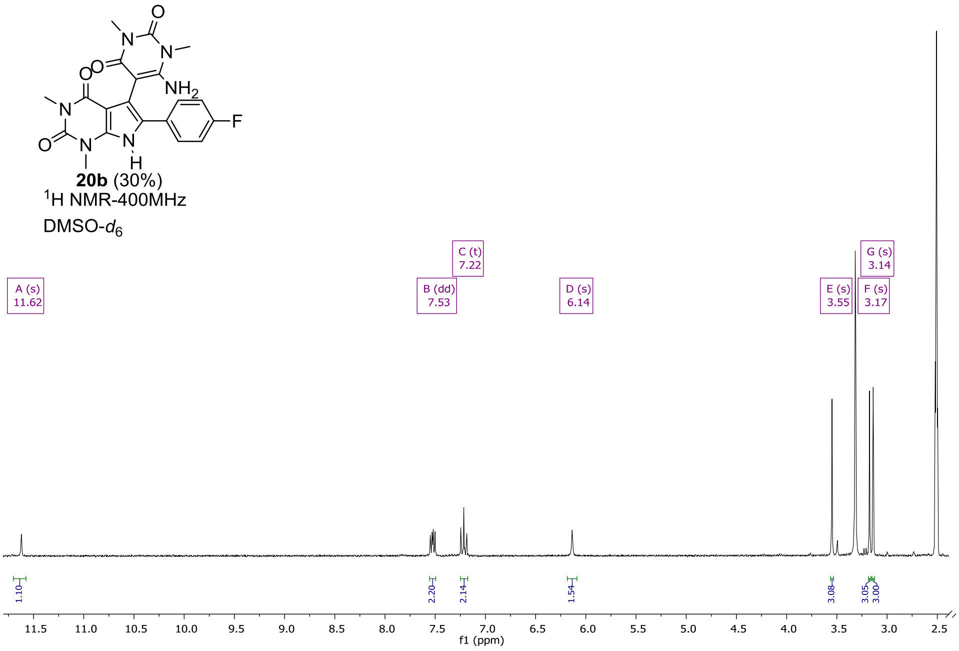

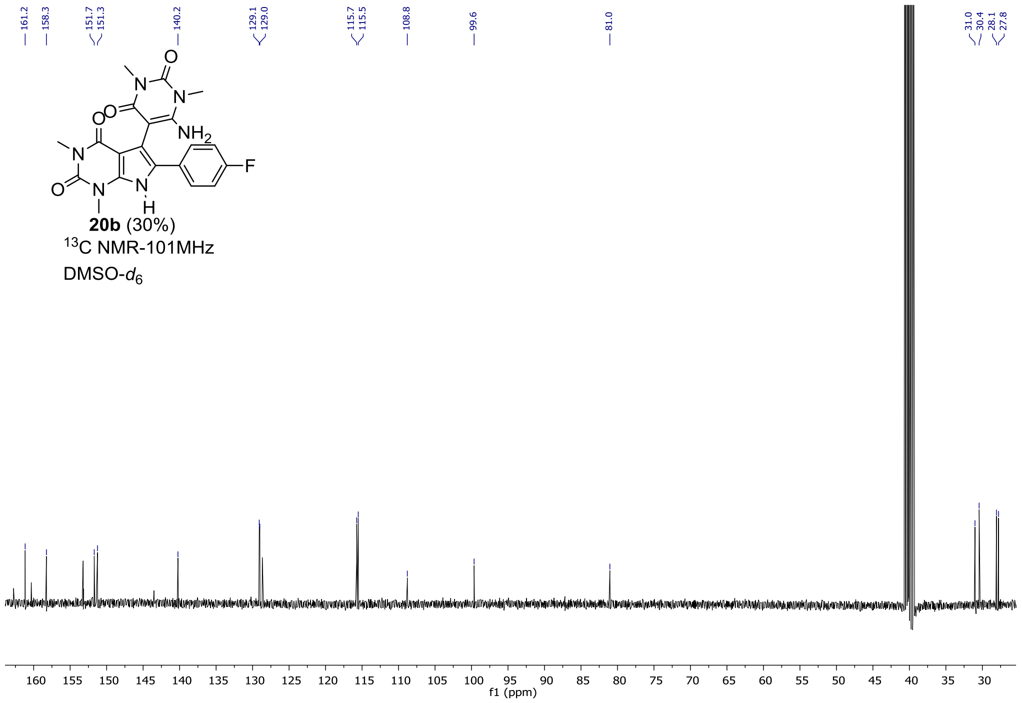
161.2
158.3
151.7
151.3
140.2
129.1
129.0
115.7
115.5
108.8
99.6
81.0
31.0
30.4
28.1
27.8
O
N
N
O
NH2
N
N
O
O
N
H
F
20b (30%)
13C NMR-101MHz
DMSO-d6
f1 (ppm)

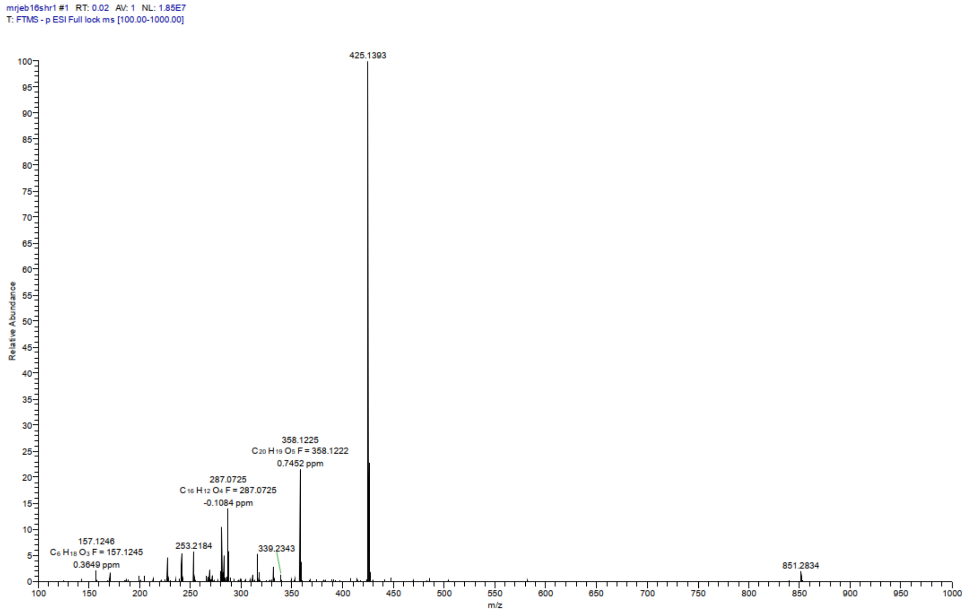
mrjeb16shr1 #1 RT: 0.02 AV: 1 NL: 1.85E7
T: FTMS - p ESI Full lock ms [100.00-1000.00]
425.1393
358.1225
C20 H15 O5 F = 358.1222
0.7452 ppm
287.0725
C16 H12 O4 F = 287.0725
-0.1084 ppm
157.1246
C8 H15 O3 F = 157.1245
0.3649 ppm
253.2184
339.2343
851.2834
Relative Abundance
m/z

5-(6-Amino-1,3-dimethyl-2,4-dioxo-1,2,3,4-tetrahydropyrimidin-5-yl)-6-(4-nitrophenyl)-1,3-dimethyl-1,7-dihydro-2H-pyrrolo[2,3-d]pyrimidine-2,4(3H)-dione (**20c**)

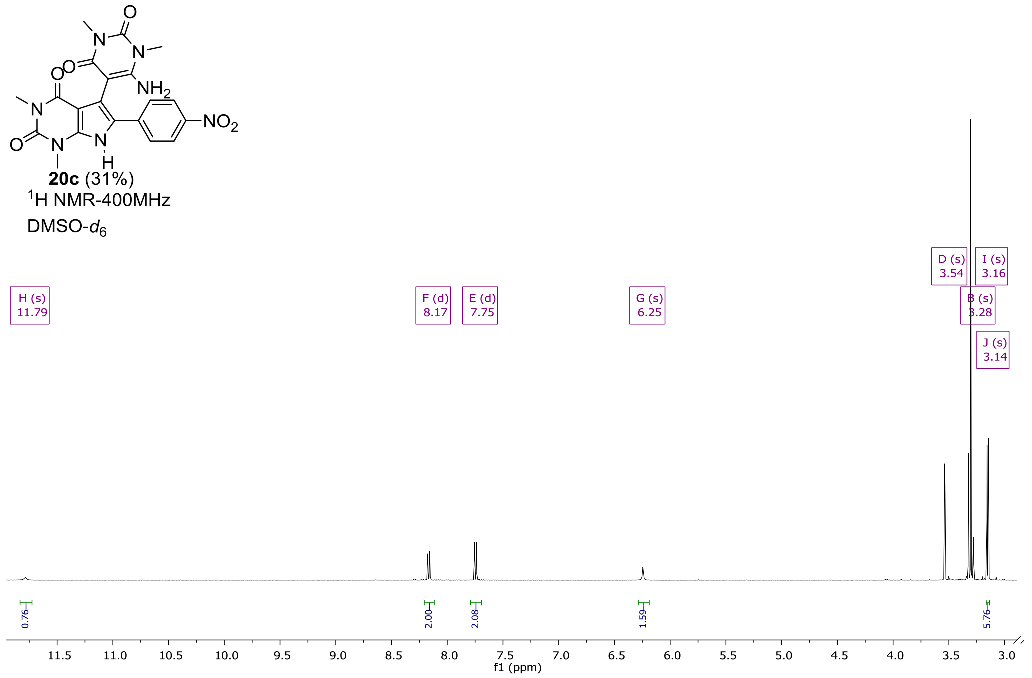

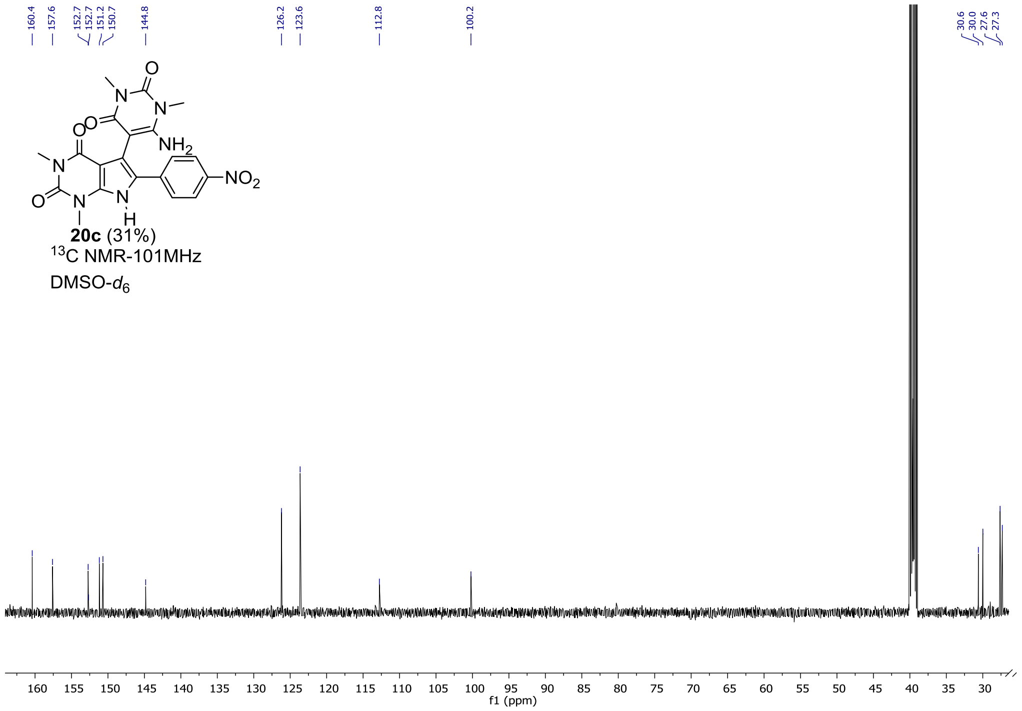

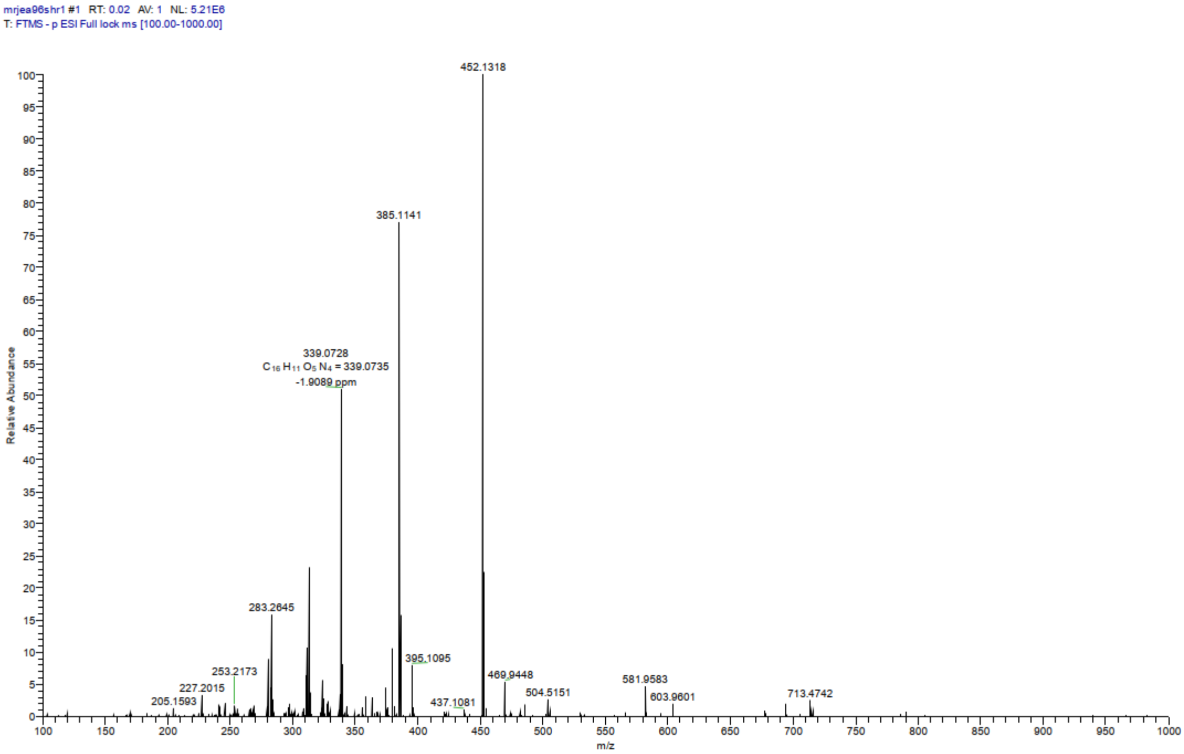
mrjea96shr1 #1 RT: 0.02 AV: 1 NL: 5.21E6
T: FTMS - p ESI Full lock ms [100.00-1000.00]
452.1318
385.1141
339.0728
C 16 H 11 O 5 N 4 = 339.0735
-1.9089 ppm
283.2645
395.1095
253.2173
227.2015
205.1593
469.9448
437.1081
504.5151
581.9583
603.9601
713.4742
Relative Abundance
m/z
